# An educational pathway and teaching materials for first aid training of children in sub-Saharan Africa based on the best available evidence

**DOI:** 10.1186/s12889-020-08857-5

**Published:** 2020-06-03

**Authors:** Emmy De Buck, Jorien Laermans, Anne-Catherine Vanhove, Kim Dockx, Philippe Vandekerckhove, Heike Geduld

**Affiliations:** 1 Centre for Evidence-Based Practice, Belgian Red Cross, Motstraat 40, 2800 Mechelen, Belgium; 2 Cochrane First Aid, Motstraat 40, 2800 Mechelen, Belgium; 3 Department of Public Health and Primary Care, Faculty of Medicine, Catholic University of Leuven, Kapucijnenvoer 35, 3000 Leuven, Belgium; 4 Cochrane Belgium, Center for Evidence-Based Medicine (Cebam), Kapucijnenvoer 33, 3000 Leuven, Belgium; 5 Belgian Red Cross, Motstraat 40, 2800 Mechelen, Belgium; 6 Division of Emergency Medicine, Stellenbosch University, Cape Town, South Africa

**Keywords:** First aid training, Health education, Schools, Children, First aid knowledge, First aid skills, Helping behavior, Evidence-based practice, sub-Saharan Africa, Low- and middle-income countries

## Abstract

**Background:**

First aid training is a cost-effective way to decrease the burden of disease and injury in low- and middle-income countries (LMIC). Since evidence from Western countries has shown that children are able to learn first aid, first aid training of children in LMIC may be a promising way forward. Hence, our project aim was to develop contextualized materials to train sub-Saharan African children in first aid, based on the best available evidence.

**Methods:**

Systematic literature searches were conducted to identify studies on first aid education to children up to 18 years old (research question one), and studies investigating different teaching approaches (broader than first aid) in LMIC (research question two). A multidisciplinary expert panel translated the evidence to the context of sub-Saharan Africa, and evidence and expert input were used to develop teaching materials.

**Results:**

For question one, we identified 58 studies, measuring the effect of training children in resuscitation, first aid for skin wounds, poisoning etc. For question two, two systematic reviews were included from which we selected 36 studies, revealing the effectiveness of several pedagogical methods, such as problem-solving instruction and small-group instruction. However, the certainty of the evidence was low to very low. Hence expert input was necessary to formulate training objectives and age ranges based on “good practice” whenever the quantity or quality of the evidence was limited. The experts also placed the available evidence against the African context.

**Conclusions:**

The above approach resulted in an educational pathway (i.e. a scheme with educational goals concerning first aid for different age groups), a list of recommended educational approaches, and first aid teaching materials for children, based on the best available evidence and adapted to the African context.

## Background

In sub-Saharan Africa, approximately 5·1 million deaths each year are attributable to conditions that could have potentially been addressed by prehospital and emergency care [1], which is however underdeveloped in the majority of low- and middle-income countries (LMIC) [2, 3]. Hence, first aid training is promoted by the World Bank as a very cost-effective way to decrease the burden of disease and injury, with a cost of only 8 USD per disability-adjusted life year averted [1]. Although most studies demonstrating the effectiveness of first aid training programmes for adults were conducted in Western contexts [4–8], some studies from sub-Saharan Africa are also available [9–11], and several African Red Cross National Societies organize first aid trainings for adult laypeople. These trainings were mostly based on materials from Western former colonisers (e.g. the UK, France), which provided a useful basis, but did not take into account the distinct African context. The need for contextualized African materials was expressed by several African Red Cross National Societies in response to a survey sent out by the Belgian Red Cross in 2009, that aimed to collect information on the availability and source of first aid training materials in African Red Cross societies [2]. In an attempt to meet these needs, contextualized and evidence-based African First Aid Materials (AFAM) were developed and released in 2011, providing guidance on up-to-date first aid techniques, as well as injury and disease prevention advice specific for the African context [2].

Since emerging evidence from Western countries has shown that children and adolescents from 5 to 18 years old are able to learn certain first aid techniques and are willing to provide help [12–16], first aid training of children seems a promising way forward in order to maximize impact. However, to our knowledge evidence-based guidelines or teaching materials concerning first aid training to children in LMIC are currently non-existent. As we believe that context is of paramount importance (e.g. to decide on which topics to include, to take into account local habits and beliefs, to take into account local resources and available equipment) we decided to develop materials specifically for the African context.

To support the development of materials to train children in first aid, and to facilitate the integration of first aid training into the school curriculum, an educational pathway may be a useful tool. An educational pathway is an instrument that indicates how children can achieve necessary competences over a certain period of time. It is based on the spiral cognitive theory of learning, stating that “any subject can be taught effectively in some intellectually honest form to any child at any stage of development” [17]. Within an educational pathway (or “spiral curriculum”) basic facts of a topic are taught first, and complexity gradually increases as the child’s age and learning progresses. Reemphasis (“repeated exposure” or “scaffolding”) helps to reinforce and solidify the learning content, so that it can enter the learner’s long-term memory. In the field of first aid, such repetition could help to automate actions, so that learners no longer have to think about each step during stressful first aid situations.

In addition to the importance of the child’s age, the teaching strategy used during the first aid training also affects the child’s knowledge, skills, and attitude. Evidence-based education research, based on many meta-analyses, has shown that classroom methods such as cooperative learning, and feedback or problem-solving methods are effective educational methods that improve learning [18, 19]. However, the evidence base mainly consists of Western studies, and less is known about effective educational methods in LMIC.

The aims of this project were: (1) to develop an educational pathway for first aid training to children (5–18 years) in sub-Saharan Africa making use of the best available evidence, and (2) to create an overview of effective educational methods in the sub-Saharan African context, with the overall aim of developing teaching materials for first aid training of sub-Saharan African children.

## Methods

To provide a basis for the educational pathway and teaching materials, several systematic literature searches were performed. The reporting of the systematic literature reviews was done according to the PRISMA (Preferred Reporting Items for Systematic Reviews and Meta-Analyses) statements (Additional File 1) [20]. No protocol for the systematic literature searches was published beforehand.

### Selection of first aid topics

The following topics were included in the educational pathway: ‘general principles’, ‘four main steps in first aid’ (including the assessment of consciousness, and knowledge about fainting), ‘resuscitation’, ‘choking’, ‘skin wounds’, ‘burns’, ‘bleeding’, ‘poisoning’, ‘injuries to bones, muscles or joints’, ‘stings and bites’, ‘fever’, ‘diarrhoea’, and ‘epilepsy’. This choice was based on the content of the previously developed AFAM [2], epidemiological data on disease burden for sub-Saharan Africa, and expert input. The topic ‘disaster principles’ was added after the consensus meeting (see 2.5), since the expert panel considered it a highly relevant topic in the sub-Saharan African context.

### Systematic literature searches and study selection

Our first research question concerned the effectiveness of first aid education to children of different age groups, on first aid knowledge, skills, and attitude. A similar and previously published systematic review (search date: 2012) was used as a basis for the current searches [12]. New first aid topics were added and selection criteria were adapted to the African context (e.g. training on the use of an automated external defibrillator was not included due to limited resource availability).

Several parallel literature searches were performed for each of the different first aid topics mentioned above, either by updating the existing literature searches (publication date between previous search dates (January 2012, 12) and the current search date (March 232,017)) or by developing new search strategies for new topics. All searches were run in two databases (MEDLINE and Embase). Search strategies and selection criteria can be found in Additional files 2 and 3, respectively.

Our second research question concerned the effectiveness of different educational approaches on children’s knowledge, skills, and attitude in LMIC. Instead of focusing on first aid education, we broadened this research question to education in general. We searched for existing systematic reviews published between 2012 and 2017 (The Campbell Library, MEDLINE, Embase, ERIC and the 3ie Database of Systematic Reviews), since we only wanted to include the most recent educational approaches. Search strategies and selection criteria can be found in Additional files 4 and 5, respectively. The scope of the educational interventions of interest was narrowed to three categories: (1) the provision of traditional hardware instructional materials (e.g. text books, flip-charts), (2) use of ‘structured pedagogy interventions’ (i.e. a combination of newly developed structured lesson content and teacher training in delivering such materials, whether or not in combination with teacher and/or student materials), and (3) use of alternative pedagogical methods (e.g. cooperative teaching, constructivist-based teaching, problem-solving method of teaching).

Study selection was done by 1 reviewer for both research questions, based on title and abstract, and subsequently based on full text.

### Data extraction, data synthesis and quality assessment

For both research questions, the following data were extracted by a single reviewer: study design; characteristics of the population (number of participants, age range); characteristics of the specific programmes (content, duration); methods of outcome measurement; means, mean differences (MDs)/standardized mean differences (SMDs), and confidence intervals (CIs) for continuous data, and risks/odds, risk ratios (RRs)/odds ratios (ORs), and CIs for dichotomous data. In addition, a risk of bias assessment of all individual studies, and an assessment of the overall certainty of evidence (per outcome for question one, and per intervention for question two) was performed using the GRADE (Grading of Recommendations Assessment, Development and Evaluation) approach [21]. For research question one, the evidence was synthesized in a narrative way, because meta-analysis was not possible due to heterogeneity at population, intervention and outcome level. For research question two, effect sizes were extracted from the meta-analyses performed in the identified systematic reviews.

### Making a first draft of the educational pathway

Based on the best available evidence collected under research question one, a first version of the educational pathway was drafted for 2-year age groups (5–6 years, 7–8 years etc.). When evidence was scarce or of low quality, we only formulated training objectives without a specific proposal for the age ranges. For each of the first aid topics, different training objectives were listed and categorized as competences at one of the three following levels (based on the 3 learning domains of Bloom’s Taxonomy of Educational Objectives [22]): (1) Knowledge: defined as the acquired information stored in the memory in an organized manner, (2) Skill: defined as the ability to practically apply the acquired information into a certain action, or (3) Attitude: defined as the willingness/self-efficacy to show a particular behavior. In line with the idea of gradually increasing complexity (according to Bruner’s spiral cognitive theory of learning [17]), we indicated at what age each of the objectives should be: (1) encouraged: the trainer actively pays attention to a certain goal and encourages the children to achieve the goal, (2) known: the trainer makes explicit efforts so that all the children can acquire a certain knowledge item, skill or attitude, or (3): repeated: the trainer repeats and emphasizes the purpose consciously for the children who have already reached the objective and strives to reach the learners who have not yet achieved the objective.

### Consensus meeting and finalizing the educational pathway, based on the best available evidence

In a next step, a panel of experts, consisting of 4 first aid practitioners of several African Red Cross National Societies, as well as 6 academic educational experts and clinicians from sub-Saharan origin (French and English speaking countries), and chaired by HG, was gathered for consultation in Johannesburg, South Africa (17-18 November, 2017). Before the start of the meeting, the draft educational pathway and specific preparatory questions were sent to the panel members. The goal of the meeting was two-fold: (1) to discuss the draft educational pathway (age ranges and training objectives), especially where evidence was scarce or of low quality, to adapt the educational pathway to the local context, and to reach consensus on the final pathway; and (2) to compile a list of effective educational approaches for children, tailored to the sub-Saharan context. More details on the preparatory questions to the panel and on how consensus was reached to achieve these goals can be found in Additional file 6. In short, for each question/item asked to the panel, opinions were collected and a discussion was held in case of disagreement. Next, a proposal was made by the chair, and consensus was sought by hand-raising. If consensus was not reached (i.e. no unanimous agreement), concerns were further addressed in a second discussion and the proposal was further adapted until full consensus was reached.

During the consensus meeting, the expert panel argued that ‘disaster principles’ would be very relevant to add as a first aid topic in the pathway. As none of the identified studies looked at disaster principles as part of the first aid training, it was decided to only formulate knowledge objectives and to agree on appropriate age ranges based on expert opinion only (without searching for additional evidence).

### Development of teaching materials

In a last phase, teaching materials were developed, taking into account: (1) the educational pathway, showing which first aid competences can be achieved at a certain age, (2) the list of effective educational methods, and (3) the content of the evidence-based AFAM, which was updated in 2016 (i.e. the specific first aid interventions) [2]. These teaching materials were circulated electronically for feedback from the expert panel, and a final conference call was organized to discuss this feedback and finalise the materials in November 2018.

## Results

### Study identification and study characteristics

**Fig. 1:**
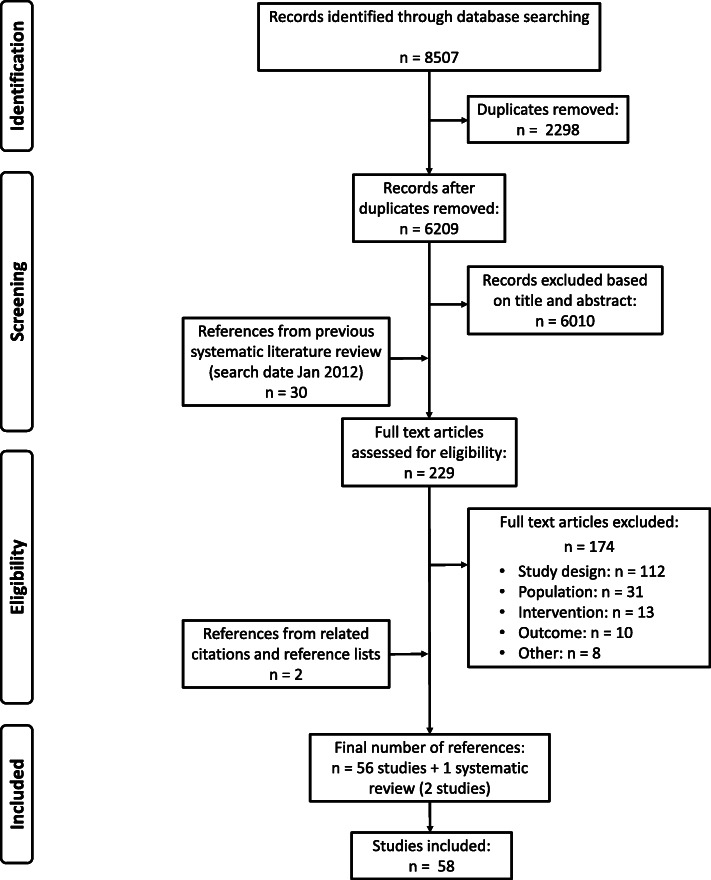
PRISMA study selection flowchart for research question one

For research question one, 58 studies were identified (see Fig. 1). Of these, 43% (25 studies) was performed in Europe, whereas only 4% (two studies) were conducted in Africa. For the first aid topics other than ‘resuscitation’, evidence was scarce or even non-existent. Detailed characteristics of the 58 included studies are listed in Additional file 7.

**Fig. 2:**
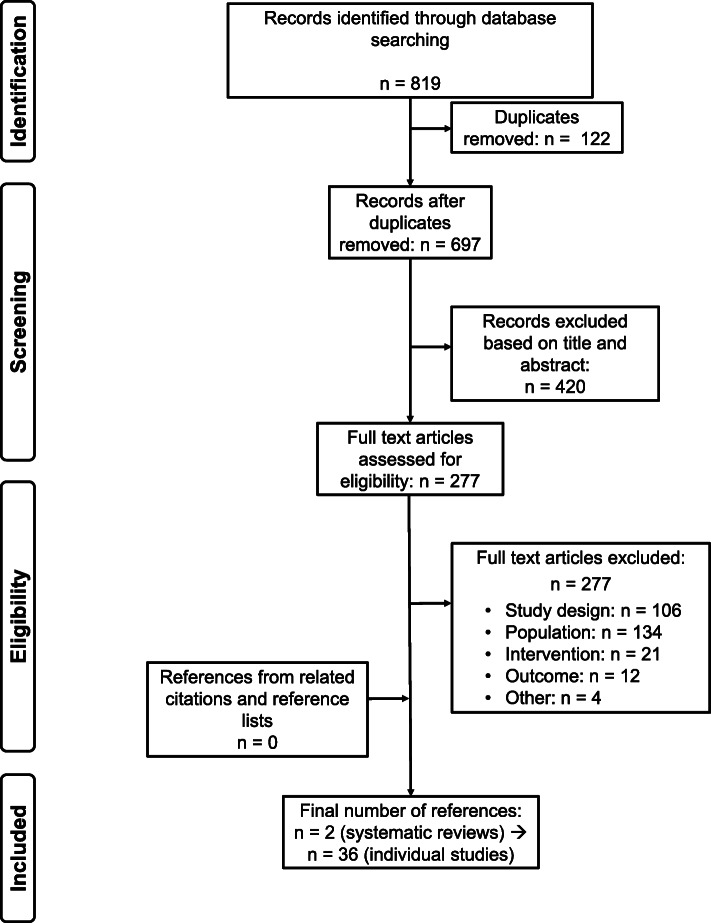
PRISMA study selection flowchart for research question two

For research question two, 819 references were screened and finally two systematic reviews were included (see Fig. 2). The first systematic review included 238 (quasi-)experimental studies, conducted in LMIC, studying a large range of interventions aimed at improving school enrolment, attendance, drop-out, completion and learning as primary outcomes [23]. Of interest to our research question were four studies looking at the provision of instructional materials (e.g. textbooks, flip-charts), and 19 studies investigating structured pedagogy interventions (as defined above). The second systematic review was a PhD thesis of 66 (quasi-)experimental studies, conducted in sub-Saharan Africa, that looked at the same interventions as listed above, as well as interventions focused on improving repetition and retention rates [24]. Of these, we included 16 studies on the use of alternative pedagogical methods, of which three were also included in the category of structured pedagogy interventions. Detailed study characteristics are presented in Additional file 8.

### Best available evidence on the effectiveness of first aid training to children

In the paragraphs below, the findings concerning the effectiveness of first aid training interventions for ‘burns’, ‘bleeding’, and ‘skin wounds’ are discussed in detail. The detailed findings on the other first aid topics, as well as the risk of bias and certainty of evidence, can be consulted in Additional file 7.

In total, we identified six studies that included burns in their training programme (see Table 1 for study characteristics, and Table 2 for study findings; detailed study characteristics can be found in Additional file 7) [25–30]. A statistically significant increase in first aid knowledge was shown after attending a first aid training course, compared to the baseline situation or control group without training, in children of 6–7 years old (MD(%): 27, 95% CI [11;40]) [27], 10–11 years old (MD: 0.2, 95% CI [0.08;0.32]) [26], 10–15 years old (OR: 1.83, *p* = 0.026) [27], and 11–15 years old (RR: 19.80, *p* < 0·001, immediately after the course; RR: 20.00, *p* < 0.001, after 2 months follow-up) [30]. Two studies also measured the children’s skills, by providing an audio-recorded scenario with a severe burn injury in a toddler or a description of a situation requiring first aid for burns [25, 30]. In a pre-post study with 11- to 15-year-olds, a significant increase in first aid skills was found (RR: 189.00, *p* < 0·001, immediately after training; RR: 149.00, *p* < 0·001, after 2 months follow-up) [30]. However, in a study with 11- to 16-year-olds that used a placebo training (on tobacco and alcohol prevention) as a control, no increase in skills concerning the order of first aid responses and listing the correct procedures, could be demonstrated (RR: 7.52, 95% CI [0.89;63.69] and RR: 1.05, 95% CI [0.91;1.21], respectively) [25].

**Table 1:** Characteristics of included studies on the effectiveness of first aid education for burns, bleeding and skin wounds

Author, year, Country	Study design	Population	Comparison	Outcomes
Campbell, 2001, USA [25]	Experimental: randomised controlled trial	*Nr. of participants:* 660 students (51% males, 49% females) *Age range:* 11–16 years	**Intervention:** *Programme:* First aid and home safety training **Control:** *Programme:* Tobacco and alcohol prevention programme	Measured before, immediately after and 1 year after training: *Knowledge:* Emergency response procedures (check-call-care); First aid kit *Skills:* Responses to two audio-recorded scenarios (glass wound, burn injury). Type and order of responses were scored: Check the scene and victim; Call 911; Care for the victim *Attitude:* First aid confidence
Frederick, 2000, UK [26]	Experimental: controlled before after study	*Nr. of participants:* 1096 students (gender not reported) *Age range:* 10–11 years	**Intervention:** *Programme:* Injury Minimization Program for Schools (IMPS) **Control:** No intervention	Outcomes were measured before and 5 months after training: *Knowledge:* Self-developed quiz *Skills:* Performance on a basic life support scenario. [only measured post intervention] *Attitude:* Record observations of dangerous behavior in a video
Heard, 2013, USA [27]	Observational: cohort study	*Nr. of participants:* 2747 students (gender not reported) *Age range:* 10–15 years	**Intervention:** *Programme:* Burn care and prevention	Outcomes were measured before (2012) and 11 months after training (2012 resurvey): *Knowledge:* 10-item survey on burn first aid
Uray, 2003, Austria [28]	Observational: before after study	*Nr. of participants:* 47 students (20 males, 27 females) *Age range:* 6–7 years	**Intervention:** *Programme:* First aid training	Outcomes were measured before and immediately after training: *Knowledge:* Questionnaire in which students had to place three cartoon-like illustrations in the correct sequence
Wafik, 2014, Egypt [30]	Observational: before after study	*Nr. of participants:* 100 students (gender not reported) *Age range:* 11–16 years	**Intervention:** *Programme:* First aid training	Outcomes were measured before, immediately after and 2 months after training: *Knowledge*: 32 item questionnaire. *Skills*: Performance on five first aid scenarios (choking, burns, poisoning, and fractures)
Wilks, 2016, Australia [29]	Observational: before after study	*Nr. of participants*: 107 students (51 males, 56 females) *Age range*: 11–12 years	**Intervention:** *Programme*: First aid, CPR and beach safety training	Outcomes were measured before, 1 week after, and 8 weeks after training: *Knowledge:* 50-item quiz on emergency services and life-supporting first aid

**Table 2:** Study findings of the studies investigating the effectiveness of first aid education for burns, bleeding and skin wounds. 2A. Burns, 2B. Bleeding, 2C. Skin wounds

Outcome	Comparison	Effect Size	#studies, # participants, age	Reference
**A. Burns**				
Knowledge: Burns FA	FA training vs no intervention	Statistically significant: 1.5±1.0 vs 1.3±1.0 MD: 0.2, 95% CI [0.08;0.32] (*p* < 0.001)^a^ *in favour of first aid training*	1, 542 vs 554, 10-11 years	Frederick, 2000 [26]
Knowledge: what to do when clothes catch fire	Pre vs 11 month follow-up	Statistically significant: 1873 (85%) vs 179 (57%) OR: 0·41 ^b,d^ (*p* < 0.01) *in favour of first aid training*	1, 2197 vs 312, 10-15 years	Heard, 2013 [27]
Knowledge: Burns FA	Pre vs 11 month follow-up	Statistically significant: 1820 (83%) vs 258 (83%) OR: 1.83 ^b,d^ (*p* = 0.03) *in favour of first aid training*	1, 2197 vs 312, 10-15 years	Heard, 2013 [27]
Knowledge: Burns FA (score >60%)	Pre vs post	Statistically significant: 5 (5%) vs 99 (99%) ^e^ RR: 19.80^a b^ (*p* < 0.001) *in favour of first aid training*	1, 100, 11-15 years	Wafik, 2014 [30]
Knowledge: Burns FA (score >60%)	Pre vs 2 month follow-up	Statistically significant: 5 (5%) vs 100 (100%) ^e^ RR: 20.00^a b^ (*p* < 0.001) *in favour of first aid training*	1, 100, 11-15 years	Wafik, 2014 [30]
Skills: Burns practice (score >60%)	Pre vs post	Statistically significant: 0 (0%) vs 94 (94%) ^e^ RR: 189.00^a b^ (*p* < 0.001) *in favour of first aid training*	1, 100, 11-15 years	Wafik, 2014 [30]
Skills: Burns practice (score >60%)	Pre vs 2 month follow-up	Statistically significant: 0 (0%) vs 74 (74%) ^e^ RR: 149.00^a b^ (*p* < 0.001) *in favour of first aid training*	1, 100, 11-15 years	Wafik, 2014 [30]
Skills: Order of FA response	FA training vs sham intervention	Scenario (severe burn injury) Not statistically significant: 5 (3%) vs 1 (0·5%) ^e^ RR: 7.52, 95% CI [0.89;63.69]^a c^ (*p* = 0.06)	1, 147 vs 221, 11-16 years	Campbell, 2001 [25]
Skills: Correct procedures listed	FA training vs sham intervention	Scenario (severe burn injury) Not statistically significant: 104 (73%) vs 149 (69%) ^e^ RR: 1.05, 95% CI [0.91;1.21]^a c^ (*p* = 0.49)	1, 147 vs 221, 11-16 years	Campbell, 2001 [25]
**B. Bleeding**				
Knowledge: cuts and bleeding FA	Pre vs 1 week post	Statistically significant: 74 (69%) vs 94 (90%) ^e^ RR: 1.33^a b^ (*p* < 0.001- overall p) *in favour of first aid training*	1, 107 vs 102, 11-12 years	Wilks, 2015 [29]
Knowledge: cuts and bleeding FA	Pre vs 8 week follow-up	Statistically significant: 74 (69%) vs 93 (82%) ^e^ RR: 1.28^a b^ (*p* < 0.001- overall p) *in favour of first aid training*	1, 107 vs 105, 11-12 years	Wilks, 2015 [29]
Knowledge: Haemorrhage FA (score >60%)	Pre vs post	Statistically significant: 34 (34%) vs 97 (97%) ^e^ RR: 2.85^a b^ (*p* < 0.001) *in favour of first aid training*	1, 100, 11-15 years	Wafik, 2014 [30]
Knowledge: Haemorrhage FA (score >60%)	Pre vs 2 month follow-up	Statistically significant: 34 (34%) vs 92 (92%) ^e^ RR: 2.71^a b^ (*p* < 0.001) *in favour of first aid training*	1, 100, 11-15 years	Wafik, 2014 [30]
Skills: Order of FA response	FA training vs sham intervention	Scenario (severe glass wound) Statistically significant: 21 (14%) vs 10 (5%) ^e^ RR: 3.16, 95% CI [1.53;6.51]^a^ (*p* < 0.001) *in favour of first aid training*	1, 147 vs 221, 11-15 years	Campbell, 2001 [25]
Skills: Correct procedures listed	FA training vs sham intervention	Scenario (severe glass wound) Not statistically significant: 75 (52%) vs 125 (57%) ^e^ RR: 0.90, 95% CI [0.74;1.10]^a c^ (*p* = 0.41)	1, 147 vs 221, 11-15 years	Campbell, 2001 [25]
**C. Skin wounds**				
Knowledge: cuts and bleeding FA	Pre vs 1 week post	Statistically significant: 74 (69%) vs 94 (90%) ^e^ RR: 1·33^a b^ (*p* < 0.001- overall p) *in favour of first aid training*	1, 107 vs 102, 11-12 years	Wilks, 2016 [29]
Knowledge: cuts and bleeding FA	Pre vs 8 week follow-up	Statistically significant: 74 (69%) vs 93 (82%) ^e^ RR: 1.28^a b^ (*p* < 0.001- overall p) *in favour of first aid training*	1, 107 vs 105, 11-12 years	Wilks, 2016 [29]
Knowledge: Wounds FA (score >60%)	Pre vs post	Statistically significant: 47 (47%) vs 96 (96%) ^e^ RR: 2.04^a b^ (*p* < 0.001) *in favour of first aid training*	1, 100, 11-15 years	Wafik, 2014 [30]
Knowledge: Wounds FA (score >60%)	Pre vs 2 month follow-up	Statistically significant: 47 (47%) vs 90 (90%) ^e^ RR: 1.91^a b^ (*p* < 0.001) *in favour of first aid training*	1, 100, 11-15 years	Wafik, 2014 [30]
Skills: Order of FA response	FA training vs sham intervention	Scenario (severe glass wound) Statistically significant: 21 (14%) vs 10 (5%) ^e^ RR: 3.16, 95% CI [1.53;6.51]^a^ (*p* < 0.001) *in favour of first aid training*	1, 147 vs 221, 11-16 years	Campbell, 2001 [25]
Skills: Correct procedures listed	FA training vs sham intervention	Scenario (severe glass wound) Not statistically significant: 75 (52%) vs 125 (57%) ^e^ RR: 0.90, 95% CI [0.74;1.10]^a c^ (*p* = 0.41)	1, 147 vs 221, 11-16 years	Campbell, 2001 [25]

Mean ± SD (unless otherwise indicated), *MD* mean difference, *RR* risk ratio, *OR* odds ratio, *RD* risk difference

^a^Calculations done by the reviewer using Revman, R software, or Excel

^b^No raw data available and CI cannot be calculated

^c^Imprecision (large variability of results)

^d^Imprecision (lack of data)

^e^Imprecision (limited sample size or low number of events)

Three studies reported on the effectiveness of first aid training concerning bleeding and skin wounds [25, 29, 30]. A statistically significant increase of knowledge concerning first aid for bleeding or skin wounds was found in children of 11 years onwards. The study by Campbell et al. in 11- to 16-year-olds showed a significant improvement in skills concerning the order of first aid responses in case of bleeding or skin wounds, but not in listing the correct procedures [25]. The certainty of the evidence was very low for the three topics.

### Best available evidence on the effectiveness of educational interventions in LMIC

We identified four studies on the provision of instructional materials to primary schools or their individual students in three different LMIC (India, Kenya and Sierra Leone), from the meta-analyses by Snilstveit et al. (2015) [23]. It could not be demonstrated that the provision of textbooks, flip-charts, or grants used directly for the purchase of materials, results in a statistically significant increase in composite test scores (SMD: 0.01 ± 0.01, 95% CI [− 0.01;0.02], *p* = 0.23), language arts test scores (SMD: 0.00 ± 0.01, 95% CI [− 0.02;0.02], *p* = 0.78) or mathematics test scores (SMD: − 0.02 ± 0.02, 95% CI [− 0.06;0.02], *p* = 0.26). The final certainty of evidence was low.

In total, 17 (quasi-)experimental studies provided 41 effect sizes on using alternative pedagogical methods on learning or testing outcomes of students attending primary or secondary schools across seven African countries (Nigeria, Kenya, Ghana, South Africa, Uganda, Liberia and Mali) [24]. Compared to the use of conventional teaching methods (mostly lecturing), the use of alternative pedagogical methods, such as problem-solving instruction, small-group instruction, guided-inquiry instruction, cooperative instruction and constructivist instruction, was shown to significantly increase the students’ learning or testing outcomes (Cohen’s d: 0.918 ± 0.314, 95% CI [0.25;1.59], *p* < 0.05). The certainty of evidence was rated as low.

Concerning structured pedagogy interventions, we obtained evidence from 19 studies in primary and secondary school students in 12 LMIC (Sudan, Kenya, Uganda, South Africa, Liberia, Mali, India, Cambodia, The Philippines, Chile, Brazil and Costa Rica) [23]. When comparing these interventions to no or other small educational interventions, a statistically significant increase in composite test scores (SMD: 0.06 ± 0.01, 95% CI [0.03;0.08], *p* < 0.0001), language arts test scores (SMD: 0.23 ± 0.05, 95% CI [0.13;0.34], *p* < 0.001) and mathematics test scores (SMD: 0.14 ± 0.03, 95% CI [0.08;0.20], *p* < 0.001) was observed. Significant changes in cognitive test scores could not be demonstrated (SMD: 0.01 ± 0.03, 95% CI [− 0.04;0.07], *p* = 0.66). The certainty of evidence was downgraded to low.

Study findings are provided in Table 3, whereas study characteristics, references of the studies included in systematic reviews, and determination of the certainty of evidence can be found in Additional file 8.

**Table 3:** Results of the meta-analyses on the effectiveness of the 3 categories of educational interventions

Outcome	Comparison	Effect Size	# effect sizes, # studies	Reference
Composite test scores (SMD±SE)	Provision of instructional materials vs no intervention (business as usual)	Not statistically significant: SMD: 0.01±0.01, 95% CI [-0.01;0.02] (*p* = 0.23) ^a^	5 effect sizes from 3 studies ^b^	Snilstveit, 2015 [23]
Language arts test scores (SMD±SE)	Provision of instructional materials vs no intervention (business as usual)	Not statistically significant: SMD: 0.00±0.01, 95% CI [-0.02;0.02] (*p* = 0.78) ^a^	5 effect sizes from 4 studies ^b^	Snilstveit, 2015 [23]
Maths test scores (SMD±SE)	Provision of instructional materials vs no intervention (business as usual)	Not statistically significant: SMD: -0.02±0.02, 95% CI [-0.06;0.02] (*p* = 0.26) ^a^	5 effect sizes from 4 studies ^b^	Snilstveit, 2015 [23]
Learning/testing outcomes (Cohen’s *d* ± SE)	Use of alternative pedagogical methods vs conventional teaching methods	Statistically significant: Cohen’s *d*: 0.918±0.314, 95% CI [0.25;1.59] (*p*<0.05)	41 effect sizes from 17 studies ^b^	Conn, 2014 [24]
Cognitive test scores (SMD±SE)	Structured pedagogy interventionsvs no intervention or other small educational intervention	Not statistically significant: SMD: 0.01±0.03, 95% CI [-0.04;0.07] (*p* = 0.66) ^a^	2 effect sizes from 2 studies ^b^	Snilstveit, 2015 [23]
Composite test scores (SMD±SE)	Structured pedagogy interventionsvs no intervention or other small educational intervention	Statistically significant: SMD: 0.06±0.01, 95% CI [0.03;0.08] (*p* < 0.0001)	3 effect sizes from 3 studies ^b^	Snilstveit, 2015 [23]
Composite test scores (SMD±SE)	Structured pedagogy interventionsvs no intervention or other small educational intervention	*Grades 1-3 sub-group:* Statistically significant: SMD: 0.09±0.02, 95% CI [0.05;0.13] (*p* < 0.0001)	2 effect sizes from 2 studies ^b^	Snilstveit, 2015 [23]
Composite test scores (SMD±SE)	Structured pedagogy interventionsvs no intervention or other small educational intervention	*Grades 4-5 sub-group:* Statistically significant: SMD: 0.08±0.02, 95% CI [0.04;0.12] (*p* < 0.0001)	2 effect sizes from 2 studies ^b^	Snilstveit, 2015 [23]
Language arts test scores (SMD±SE)	Structured pedagogy interventionsvs no intervention or other small educational intervention	Statistically significant: SMD: 0.23±0.05, 95% CI [0.13;0.34] (*p* < 0.001)	67 effect sizes from 17 studies ^b^	Snilstveit, 2015 [23]
Language arts test scores (SMD±SE)	Structured pedagogy interventionsvs no intervention or other small educational intervention	*Grades 1-3 sub-group:* Statistically significant: SMD: 0.23±0.06, 95% CI [0.11;0.35] (*p* < 0.01)	63 effect sizes from 14 studies ^b^	Snilstveit, 2015 [23]
Language arts test scores (SMD±SE)	Structured pedagogy interventionsvs no intervention or other small educational intervention	*Grades 4-6 sub-group:* Not statistically significant: SMD: 0.21±0.13, 95% CI [-0.04;0.47] (*p* = 0.10) ^a^	4 effect sizes from 4 studies ^b^	Snilstveit, 2015 [23]
Maths test scores (SMD±SE)	Structured pedagogy interventionsvs no intervention or other small educational intervention	Statistically significant: SMD: 0.14±0.03, 95% CI [0.08;0.20] (*p* < 0.001)	24 effect sizes from 14 studies ^b^	Snilstveit, 2015 [23]
Maths test scores (SMD±SE)	Structured pedagogy interventionsvs no intervention or other small educational intervention	*Grades 1-3 sub-group:* Statistically significant: SMD: 0.08±0.03, 95% CI [0.03;0.13] (*p* < 0.01)	9 effect sizes from 9 studies ^b^	Snilstveit, 2015 [23]
Maths test scores (SMD±SE)	Structured pedagogy interventionsvs no intervention or other small educational intervention	*Grades 4-6 sub-group:* Statistically significant: SMD: 0.21±0.08, 95% CI [0.04;0.37] (*p* < 0.05)	4 effect sizes from 4 studies ^b^	Snilstveit, 2015 [23]

*SMD* standardized mean difference, *SE* standard error

^a^ Imprecision (lack of data): mean of the control group is not reported

^b^ Imprecision (lack of data): total sample size is not reported

### Educational pathway on first aid for sub-Saharan Africa

The best available evidence on the effectiveness of first aid training to children was used to draft the educational pathway, which was then discussed with the expert panel for context adaptation. Since the certainty of evidence was low to very low, the expert panel had a very important role in formulating and approving training objectives and age ranges. Overall, the panel did not often disagree on the specific age ranges at which certain learning objectives should be encouraged, known or repeated. However, for some topics there was more disagreement among the panel members and a more extensive discussion was necessary, e.g. the inclusion of major incidents/disaster management as a topic, the inclusion of psychological first aid as an intervention, the management of fever, the competence of putting on gloves, the objective of willingness to touch a stranger. In each case of disagreement, arguments were listed and discussed, and a proposal was made by the chair until full consensus was achieved.

An example of how the pathway was adapted to the African context deals with seeking help from a medical care provider. In the draft pathway, it was proposed that children should know how to seek help from a medical care provider at the age of 7–8 years. Since medical care is less accessible in Africa than in high income countries, the expert panel decided to postpone this to the age of 9–10 years, and to keep on repeating until the age of 18 years. A second example deals with the general first aid competence of handwashing before and after administering first aid. Because of the higher prevalence of infectious diseases in the African context, the panel proposed to repeatedly train children until the age of 18 years.

For the topic of burns, evidence showed that children of 6–7 years old can be taught how to correctly provide first aid [28]. The expert panel extrapolated the evidence on burns knowledge in a consistent way to the topics of bleeding and skin wounds, since evidence on the latter topics for children under 11 years was lacking, hence concluding that children should have acquired the basic knowledge at the age of 7–8 years. More advanced knowledge should be attained at the age of 11–12 years (e.g. children know the link between tetanus and skin wounds), or at the age of 13–14 years (e.g. children know the different types of burns). Based on the opinion of the experts, only one knowledge item should already be acquired at the age of 5–6 years: the most common causes of burns (i.e. hot water, fire, flames). Skills competences were set in accordance with the knowledge items.

The final version of the pathway can be found in Table 4 (for the topics ‘burns’, ‘bleeding’, and ‘skin wounds’) and Additional file 9 (for all topics).

**Table 4:** Educational pathway on first aid for burns, bleeding and skin wounds, adapted to the sub-Saharan African context

E: Encourage K: Know/Know How R: Repeat	5–6 yrs	7–8 yrs	9–10 yrs	11–12 yrs	13–14 yrs	15–16 yrs	17–18 yrs
**BURNS**							
**Knowledge**							
The children: • recognise a burn	E	K	R	R	R		
• know how to provide first aid for a burn (regardless of the degree of the burn)	E	K	R	R	R		
• know when to seek medical help for a burn			E	K	R	R	R
• know the difference between a superficial, intermediate and deep burn				E	K	R	R
• know what commonly causes burns (hot water, flames, fire)	K	R	R	R			
• know what can cause a burn (heat, chemicals, radiation...)	E	E	E	E	K	R	R
**Skills**							
The children can: • correctly provide first aid for a burn	E	K	R	R	R		
• seek medical help if the burn is serious				K	R	R	R
**Attitudes**							
*See educational pathway General > Attitudes (see Additional file *9*)*							
The children recognise the importance of: • continuously applying water to a burn	E	K	R	R	R		
**BLEEDING**							
**Knowledge**							
The children know: • what they have to do in the event of a nosebleed	E	K	R	R	R		
• when to seek medical help for a nosebleed		E	K	R	R		
• how to correctly stop (severe) bleeding			E	K	R	R	
• that medical help must always be sought in the event of severe bleeding			E	K	R	R	
**Skills**							
The children can: • correctly stop a nosebleed	E	K	R	R	R		
• apply a bandage to stop (severe) bleeding				K	R	R	
**Attitudes**							
*See educational pathway General > Attitudes (see Additional file *9*)*							
The children recognise the importance of • stopping a bleeding as quickly as possible		E	K	R	R		
**SKIN WOUNDS**							
**Knowledge**							
The children: • recognise a skin wound	E	K	R	R			
• know which equipment is needed to provide first aid for a skin wound	E	K	R	R			
• know when the injured person should seek medical help for a skin wound				K	R	R	R
• know the importance of tetanus vaccinations, and know why tetanus is dangerous and linked with skin wounds				K	R	R	R
• know that an injured person with a skin wound in which a foreign object is embedded should always seek medical help				K	R	R	R
**Skills**							
The children can: • correctly provide first aid for a skin wound if clean water is available	E	K	R	R	R		
• stop the bleeding of a wound that does not stop bleeding by itself			E	K	R	R	R
• correctly provide first aid for a skin wound in which a foreign object is embedded					K	R	R
**Attitude**							
*See educational pathway General > Attitudes (see Additional file *9*)*							
The children recognise the importance of: • correctly providing first aid for a skin wound in which a foreign object is embedded				E	K	R	R

### Teaching materials for first aid training to children in sub-Saharan Africa

**Fig. 3:**
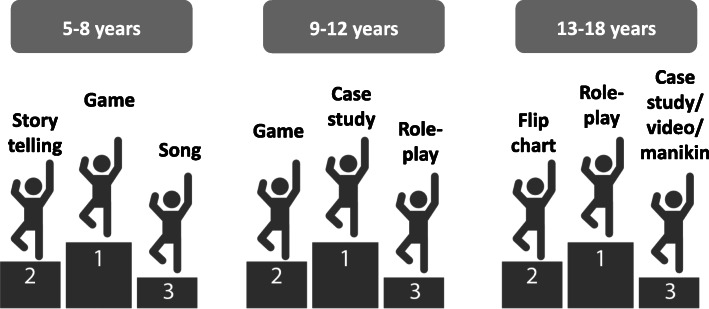
Top three of most appropriate and successful teaching methods for each age group

The panel members agreed to cluster the 2-year age ranges of the educational pathway into 3 broader age groups for the development of separate teaching materials (5–8 years old, 9–12 years old and 13–18 years old). Also, they agreed on a number of recommended educational methods, applicable to the African context. An overview of these methods, the age groups for which they are (most) appropriate, as well as their strengths and weaknesses, is presented in Table 5. The top three of most appropriate and successful teaching methods for each age group is presented in Fig. 3. Text messaging and the use of individual worksheets for children were perceived as non-feasible or non-desirable educational methods.

**Table 5:** Overview of the educational methods that can be used within the sub-Saharan African context, the appropriate age groups, their strengths and weaknesses

Educational method	Appropriate age groups			Strengths	Limitations	Additional panel remarks
	5–8 yrs	9–12 yrs	13–18 yrs			
Song	x	x	x	Easy to motivate children; Enables memorization	Passive way of receiving information; Possible lack of knowledge or understanding of the lyrics	
Quiz	x	**x**	x	Interactive; Engages all children at the same time	Language and comprehension may be a challenge, particularly for non-first language speakers	Should be adapted to the language spoken by and environment of the children; Local contextually appropriate questions should be created
Colouring	x			Enables practical rehearsal of the content; Active learning	Children may not have the necessary hand motor skills	8–12-year-olds could colour, whereas 9–12-year-olds can draw themselves
Poem	**x**	x	x	Allows for repetition, which enables memorization	Difficult wording might interfere with understanding; Can only be used for key messages	5–8-year-olds can recite a poem taught to them, whereas at a later age, children may be able to write their own poems
Puppetry	x			Very visual; Can be engaging, particularly with younger age groups	Difficult to pin content to it; Trainers should be confident with it (time-consuming)	
Drawing	x				Some children might not succeed in drawing; Can be time-consuming	The act of drawing should not interfere with learning
Case study	x	x	x	Can come from the learners themselves; Easy to adapt according to age	Not easy to find a case study that fits the content and the context; When analyzing a case study from a learner, must include and manage children’s emotional reactions to the case; Trainers should be taught to run a case study	5–8-year-olds: incorporate visual elements such as photos 9–12-year-olds: work with a story
Group work	x	x	x	Peer to peer narration and active involvement; Children can speak in their own language	Some children are quiet when in groups and might not say anything	The social aspect of group work can be initiated at 5–8 years, but group work can only really occur from 9 years of age onwards
Storytelling	**x**	x	x	Engaging and entertaining; A story can be adapted to the content and age of the child	If the content is not adjusted to the age group, children may listen without understanding	
Self-discovery	x	x	x	Experiential learning; May include take-home exercises	Need for clear guidelines before and after activity to ensure that the right lessons are learnt	
Drama and simulation	x	**x**	x	Engaging and entertaining	Challenges with group work and holding attention	
Demonstration	x	x	x	Focus on practical skills (key for First Aid training)	Depending on the size of the group, teachers may not be able to engage everyone; Demonstration needs to be done properly, so teachers need training; Skills may need to be broken down into components, to ensure that learners are able to understand and gain the practical skill	
Quotes		x	x	Can be used for big groups; − Children have to think independently whether or not they agree with the quote, and will have to take a position		

x marks an appropriate age range, **x** marks the most appropriate age range

Following the panel meeting, teaching materials were developed for the age group of 9- to 12-year-olds, incorporating the recommended teaching methods for this age group. The competencies to be achieved when following a training using these materials were based on the content of the educational pathway, again for this specific age group.

## Discussion

Our educational pathway on first aid, based on the best evidence available, links key learning objectives concerning first aid to a child’s specific age range (see Additional file 9). It denominates which topics can be covered when training children in first aid, visualizes the time points at which certain items should receive attention, and it is adapted to the African context. The final educational pathway has been laid out in an easy-to-use version, including explanations on how to use it (both the pathway and AFAM are freely downloadable from the Belgian Red Cross website after registration) [31]. One of the successes of this project is that the educational pathway, together with the list of effective educational methods, was immediately used to develop teaching materials for first aid training to African children. The final training materials were piloted in Zimbabwe, where 6 trainers were trained (Train-the-Trainer model) and then went to 6 different schools in 2 provinces (a rural, urban and semi-urban school in each province) to provide 2-h trainings to 12- to 16-year-old children. Those trainings have been observed and the trainers and children were asked to give feedback on the manual and the used educational methods (in terms of satisfaction, feasibility, appropriateness etc.). Based on this feedback, some final changes were made to the training manual. The project is currently being implemented in Zimbabwe, Malawi, Zambia and Lesotho, and further roll-out in other countries is being planned.

Our project has some important limitations. First, the systematic literature searches we performed are not systematic reviews, which is translated into the fact that only 1 reviewer conducted the study selection and data extraction, only two databases were searched for evidence on first aid training of children, and a very focused set of selection criteria was used. The reason behind this choice, which is made by many guideline developers because of feasibility reasons, is that these reviews were part of a larger guideline project in which the evidence conclusions were translated into recommendations and teaching materials. Although this can be considered a limitation of the used review method, we believe that including an expert panel can serve as a back-up for this limitation.

Second, for several first aid topics, including ‘skin wounds’, ‘burns’, ‘bleeding’, ‘injuries to bones, muscles or joints’, and ‘poisoning’, a very limited amount of evidence or no evidence was found. Therefore, many gaps had to be filled by the expert panel, based on their expertise and consensus. In addition, almost half of the studies were European studies, and only four African studies are currently included in the evidence base, which can be seen as a source of indirectness. For the educational studies, the majority of the studies looked at mathematics and language courses, which again is a source of indirectness. Third, the quality/certainty of the obtained evidence was in most cases low to very low. This is mainly due to the less rigorous study designs that are typically used to study educational programme effectiveness, including many uncontrolled before-after studies. There was also a high degree of heterogeneity between the studies, especially at the intervention level (with many differences in the content, delivery and duration of the training programme), and outcome level (measured in many different ways and at different time points). Again, this led to a very prominent role for the expert panel, and many recommendations (learning objectives/age ranges) had to be based on expert opinion.

Nevertheless, this project has several important implications for practice. First, the educational pathway on first aid can be used by first aid trainers to help them decide which content to teach to children of certain age ranges. Second, the educational pathway may be a useful tool for advocating the importance of first aid in health education with the Ministry of Health/Ministry of Education of sub-Saharan African countries. In Zimbabwe, it was recently decided by law to integrate first aid education in the school curriculum. Third, the evidence on the effectiveness of first aid training to children (Additional file 7), which is independent of any geographical region, can be used to develop similar first aid educational pathways, and accompanying training materials, for other contexts or countries, but appropriateness and feasibility for the specific context or target group should be discussed, and adaptations to the context should be made with local experts.

Since we identified several gaps in research and the certainty of the available evidence was low to very low, we also want to advocate for higher quality future research, that uses appropriate control groups, on the effectiveness of first aid training to children in different geographical regions on several learning outcomes. This will allow further development of improved first aid materials.

## Conclusions

The available evidence we identified, together with input from a multidisciplinary expert panel, was used as a basis to (1) develop an educational pathway for first aid training of sub-Saharan African children (5–18 years), and to (2) create an overview of effective educational methods in the sub-Saharan African context. The educational pathway shows which educational goals can be achieved within specific age groups, and represents a useful tool to design a first aid curriculum for children. Both the pathway and the overview of educational methods were used to develop teaching materials for first aid training of children in sub-Saharan Africa. The quantity and certainty of the evidence was low but provided a framework for the pathway and teaching materials, and highlights the clear need for higher quality research in the future.

## Supplementary information

**Additional file 1.** PRISMA checklist2

**Additional file 2.** Search strategies research question 1

**Additional file 3.** Selection criteria research question 1

**Additional file 4.** Search strategies research question 2

**Additional file 5.** Selection criteria research question 2

**Additional file 6.** Description of consensus methods used during expert panel meeting

**Additional file 7.** Characteristics of included studies, synthesis of findings, risk of bias and certainty of evidence for research question 1

**Additional file 8.** Characteristics of included studies, synthesis of findings, risk of bias and certainty of evidence for research question 2

**Additional file 9.** Evidence-based educational pathway on first aid for sub-Saharan Africa 221

## Data Availability

All data generated or analysed during this study are included in this published article and its supplementary information files.

## Abbreviations

LMIC: low- and middle income countries
AFAM: African First Aid Materials
PRISMA: Preferred Reporting Items for Systematic Reviews and Meta-Analyses
MD: mean difference
SMD: standardized mean difference
CI: confidence interval
RR: risk ratio
OR: odds ratio
GRADE: Grading of Recommendations Assessment, Development and Evaluation
