# Supplementary material for: An educational pathway and teaching materials for first aid training of children in sub-Saharan Africa based on the best available evidence
Source: BMC Public Health. 2020 Jun 3;20:836. doi: 10.1186/s12889-020-08857-5 (PMC7268765; doi:10.1186/s12889-020-08857-5)
Supplement: Supplementary file 2 — Additional file 2. Search strategies research question 1 [file 12889_2020_8857_MOESM2_ESM.docx]

# Additional file 2: Search strategies research question 1

Topics: ‘general principles of first aid’, ‘resuscitation’, ‘choking’, ‘skin wounds’, ‘burns’, ‘bleeding’, ‘injuries to bones, muscles or joints’, ‘poisoning’ and ‘stings and bites’

**Publication date:** 01/01/2012-23/03/2017

MEDLINE (via PubMed interface):

1. "Education"[Mesh] OR "Education"[Subheading] OR educat*[TIAB] OR curricul*[TIAB] OR train*[TIAB] OR teach*[TIAB] OR instruct*[TIAB] OR learn*[TIAB] OR course*[TIAB] OR lesson*[TIAB]
2. “Child”[Mesh] OR “Adolescent”[Mesh] OR child*[TIAB] OR adolescent*[TIAB]
3. "Resuscitation"[Mesh] OR "First Aid"[Mesh] OR "Emergencies"[Mesh] OR "Emergency Treatment"[Mesh:NoExp] OR ”Early Medical Intervention”[Mesh] OR resuscitation[TIAB] OR “first aid”[TIAB] OR emergency evacuation*[TIAB] OR moving victim*[TIAB]
4. ("Airway Obstruction"[Mesh] OR "airway obstruction"[TIAB] OR chok*[TIAB]) AND (“back slap”[TIAB] OR “abdominal thrust”[TIAB] OR “Heimlich Maneuver”[Mesh] OR heimlich[TIAB])
5. ”Helping Behavior”[Mesh] OR helping behaviour*[TIAB] OR helping behavior*[TIAB]
6. 3 OR 4 OR 5
7. 1 AND 2 AND 6

Embase (via Embase.com interface):

1. 'education'/exp OR educat*:ab,ti OR curricul*:ab,ti OR train*:ab,ti OR teach*:ab,ti OR instruct*:ab,ti OR learn*:ab,ti OR course*:ab,ti OR lesson*:ab,ti
2. 'child'/exp OR 'adolescent'/exp OR child*:ab,ti OR adolescent*:ab,ti
3. 'resuscitation'/exp OR 'first aid'/exp OR ‘emergency’/exp OR ‘emergency treatment’/de OR ‘early intervention’/exp OR resuscitation:ab,ti OR ‘first aid’:ab,ti OR (emergency NEXT/1 evacuation*):ab,ti OR (moving NEXT/1 victim*):ab,ti
4. (‘airway obstruction'/exp OR ‘airway obstruction':ab,ti OR chok*:ab,ti) AND (‘back slap’:ab,ti OR ‘abdominal thrust’:ab,ti OR 'heimlich maneuver'/exp OR heimlich:ab,ti)
5. ‘helping behavior’:ab,ti OR ‘helping behaviour’:ab,ti
6. 3 OR 4 OR 5
7. 1 AND 2 AND 6

**Topics: ‘**fever’, ‘diarrhoea’, ‘fits’

**Search date:** 31/01/2018

**The Cochrane Library**: ([mh “diarrhea”] OR diarrhea:ti,ab,kw OR diarrhoea:ti,ab,kw) AND ([mh “education”] OR education:ti,ab,kw)

**The Campbell Library**: diarrhoea OR diarrhea

**MEDLINE** (via PubMed interface); systematic reviews:

1. “child”[Mesh] OR “adolescent”[Mesh] OR child*[TIAB] OR adolescent*[TIAB]
2. “education”[Mesh:NoExp] OR “curriculum”[Mesh] OR curricul*[TIAB] OR educat*[TIAB] OR train*[TIAB] OR teach*[TIAB] OR instruct*[TIAB] OR learn*[TIAB] OR course*[TIAB] OR lesson*[TIAB] OR school*[TIAB]
3. diarrhoea[TIAB] OR diarrhea[TIAB] OR "Diarrhea"[Mesh]
4. knowledge[TIAB] OR "Knowledge"[Mesh]
5. (("Meta-Analysis as Topic"[Mesh] OR meta analy*[TIAB] OR metaanaly*[TIAB] OR "Meta-Analysis"[PT] OR systematic review*[TIAB] OR systematic overview*[TIAB] OR "Review Literature as Topic"[Mesh]) OR (cochrane[TIAB] OR embase[TIAB] OR psychlit[TIAB] OR psyclit[TIAB] OR psychinfo[TIAB] OR psycinfo[TIAB] OR cinahl[TIAB] OR cinhal[TIAB] OR “science citation index”[TIAB] OR bids[TIAB] OR cancerlit[TIAB]) OR (reference list*[TIAB] OR bibliograph*[TIAB] OR hand-search*[TIAB] OR “relevant journals”[TIAB] OR manual search*[TIAB]) OR ((“selection criteria”[TIAB] OR “data extraction”[TIAB]) AND "Review"[PT])) NOT ("Comment"[PT] OR "Letter"[PT] OR "Editorial"[PT] OR ("Animals"[Mesh] NOT ("Animals"[Mesh] AND "Humans"[Mesh])))
6. 1-5 AND

**Embase** (via Embase.com interface); systematic reviews:

1. 'child'/exp OR 'adolescent'/exp OR child*:ti,ab OR adolescent*:ti,ab
2. 'education'/exp OR educat*:ab,ti OR curricul*:ab,ti OR train*:ab,ti OR teach*:ab,ti OR instruct*:ab,ti OR learn*:ab,ti OR course*:ab,ti OR lesson*:ab,ti OR school*:ab,ti
3. diarrhoea:ti,ab OR diarrhea:ti,ab OR 'diarrhea'/exp
4. knowledge:ti,ab OR 'Knowledge'/exp
5. (('meta analysis (topic)'/exp OR 'meta analysis'/exp OR (meta NEXT/1 analy*):ab,ti OR metaanalys*:ab,ti OR ‘systematic review (topic)’/exp OR ‘systematic review’/exp OR (systematic NEXT/1 review*):ab,ti OR (systematic NEXT/1 overview*):ab,ti) OR (cancerlit:ab,ti OR cochrane:ab,ti OR embase:ab,ti OR psychlit:ab,ti OR psyclit:ab,ti OR psychinfo:ab,ti OR psycinfo:ab,ti OR cinahl:ab,ti OR cinhal:ab,ti OR 'science citation index':ab,ti OR bids:ab,ti) OR (‘reference list*’:ab,ti OR bibliograph*:ab,ti OR hand-search*:ab,ti OR (manual NEXT/1 search*):ab,ti OR ‘relevant journals’:ab,ti) OR ((‘data extraction’:ab,ti OR ‘selection criteria’:ab,ti) AND review/it)) NOT (letter/it OR editorial/it OR (‘animal’/exp NOT (‘animal’/exp AND 'human'/exp)))
6. 1-5 AND

**MEDLINE** (via PubMed interface); experimental studies:

1. “child”[Mesh] OR “adolescent”[Mesh] OR child*[TIAB] OR adolescent*[TIAB]
2. “education”[Mesh:NoExp] OR “curriculum”[Mesh] OR curricul*[TIAB] OR educat*[TIAB] OR train*[TIAB] OR teach*[TIAB] OR instruct*[TIAB] OR learn*[TIAB] OR course*[TIAB] OR lesson*[TIAB] OR school*[TIAB]
3. diarrhoea[TIAB] OR diarrhea[TIAB] OR "Diarrhea"[Mesh] OR fever[TIAB] OR "Fever"[Mesh] OR fits[TIAB] OR seizures[TIAB] OR epilepsy[TIAB] OR "Epilepsy"[Mesh] OR "Seizures"[Mesh]
4. knowledge[TIAB] OR "Knowledge"[Mesh]
5. ((“Clinical Trial”[PT] OR “Comparative Study”[PT] OR “Cross-Over Studies”[Mesh] OR “Clinical Trials as Topic”[Mesh] OR "Non-Randomized Controlled Trials as Topic"[Mesh] OR random*[TIAB] OR controll*[TIAB] OR “intervention study”[TIAB] OR “experimental study”[TIAB] OR “comparative study”[TIAB] OR trial[TIAB] OR evaluat*[TIAB] OR “before and after”[TIAB] OR “interrupted time series”[TIAB] OR "Controlled Before-After Studies"[Mesh] OR "Interrupted Time Series Analysis"[Mesh]) NOT ("Animals"[Mesh] NOT (Animals[Mesh] AND "Humans"[Mesh])))
6. 1-5 AND

**Embase** (via Embase.com interface); experimental studies:

1. 'child'/exp OR 'adolescent'/exp OR child*:ti,ab OR adolescent*:ti,ab
2. 'education'/exp OR educat*:ab,ti OR curricul*:ab,ti OR train*:ab,ti OR teach*:ab,ti OR instruct*:ab,ti OR learn*:ab,ti OR course*:ab,ti OR lesson*:ab,ti OR school*:ab,ti
3. diarrhoea:ti,ab OR diarrhea:ti,ab OR 'diarrhea'/exp OR fever:ti,ab OR 'Fever'/exp OR fits:ti,ab OR seizures:ti,ab OR epilepsy:ti,ab OR 'Epilepsy'/exp OR 'Seizures'/exp
4. knowledge:ti,ab OR 'Knowledge'/exp
5. ('randomized controlled trial'/exp OR 'clinical trial'/exp OR 'comparative study'/exp OR random*:ab,ti OR control*:ab,ti OR ‘intervention study’:ab,ti OR ‘experimental study’:ab,ti OR ‘comparative study’:ab,ti OR trial:ab,ti OR evaluat*:ab,ti OR ‘before and after’:ab,ti OR ‘interrupted time series’:ab,ti) NOT ('animal'/exp NOT 'human'/exp)
6. 1-5 AND
