# Supplementary material for: An educational pathway and teaching materials for first aid training of children in sub-Saharan Africa based on the best available evidence
Source: BMC Public Health. 2020 Jun 3;20:836. doi: 10.1186/s12889-020-08857-5 (PMC7268765; doi:10.1186/s12889-020-08857-5)
Supplement: Supplementary file 3 — Additional file 3. Selection criteria research question 1 [file 12889_2020_8857_MOESM3_ESM.docx]

# Additional file 3: Selection criteria research question 1

**PICO:** ‘Is first aid education (related to one of the topics listed above) (Intervention) an effective intervention in children (5-18 years) (Population) to improve first aid knowledge, skills and/or attitudes (Outcome) compared to no first aid training (Comparison)?

Population: *Include*: children or adolescents between 5 to 18 years old. *Exclude:* children with special educational needs (e.g. due to intellectual or physical disabilities).

Intervention: *Include*: studies on first aid education concerning one of the topics listed above. A general first aid training was only considered if outcomes for the at least one of the different first aid topics were measured separately. *Exclude*: studies evaluating online training only, studies in which first aid is only a very small part of a larger curriculum.

Comparison: *Include*: studies with a passive control group (no training), or baseline measurement, or comparing different age groups. *Exclude*: studies comparing different training methods without a passive control group or a baseline measurement control condition.

Outcome: *Include*: studies that measured first aid knowledge, skills, attitudes regarding the different first aid topics. For diarrhoea, fever and fits, the panel agreed that only competences on knowledge had to be included in the pathway. *Exclude*: studies containing only a general first aid score for knowledge, skills or attitudes.

Study design: *Include*: a systematic review: inclusion of the studies of the systematic review if the search strategy and selection criteria are clearly described and if the systematic review has critically appraised the methodological quality of the included individual studies. An experimental study: inclusion in case of one of the following study types: (quasi or non-) randomised controlled trial, (controlled) before and after study. An observational study: inclusion in case of one of the following study types: cohort and case-control study, (controlled) before and after study. *Exclude*: animal studies, ex vivo or in vitro studies, letter to the editor, conference abstracts, studies reporting no quantitative data, and studies reporting only means but no SDs, effect sizes or p-values.

Language: *Include*: English.
