# Supplementary material for: An educational pathway and teaching materials for first aid training of children in sub-Saharan Africa based on the best available evidence
Source: BMC Public Health. 2020 Jun 3;20:836. doi: 10.1186/s12889-020-08857-5 (PMC7268765; doi:10.1186/s12889-020-08857-5)
Supplement: Supplementary file 4 — Additional file 4. Search strategies research question 2 [file 12889_2020_8857_MOESM4_ESM.docx]

# Additional file 4: Search strategies research question 2

**Publication date:** 01/01/2012-24/04/2017

The Campbell Library: 'education'

MEDLINE (via PubMed interface):

1. "Education"[Mesh] OR educat*[TIAB] OR “Learning”[Mesh] OR learn*[TIAB] OR teach*[TIAB]
2. “Child”[Mesh] OR “Adolescent”[Mesh] OR child*[TIAB] OR youth[TIAB] OR teenage*[TIAB] OR adolescent*[TIAB] OR “Students”[Mesh] OR student*[TIAB] OR “Schools”[Mesh] OR school*[TIAB]
3. “Developing Countries”[Mesh] OR “developing countr*”[TIAB] OR “low income countries”[TIAB] OR “middle income countries”[TIAB]
4. ((((((((((((Meta-Analysis as Topic[Mesh])) OR ((meta analy*[TIAB]))) OR ((metaanaly*[TIAB]))) OR ((Meta-Analysis[Publication Type]))) OR ((systematic review*[TIAB] OR systematic overview*[TIAB]))) OR ((Review Literature as Topic[Mesh])))) OR ((cochrane[TIAB] OR embase[TIAB] OR psychlit[TIAB] OR psyclit[TIAB] OR psychinfo[TIAB] OR psycinfo[TIAB] OR cinahl[TIAB] OR cinhal[TIAB] OR science citation index[TIAB] OR bids[TIAB] OR cancerlit[TIAB]))) OR ((reference list*[TIAB] OR bibliograph*[TIAB] OR hand-search*[TIAB] OR relevant journals[TIAB] OR manual search*[TIAB]))) OR ((((selection criteria[TIAB] OR data extraction[TIAB])) AND ((Review[PT])))))) NOT ((Comment[PT] OR Letter[PT] OR Editorial[PT] OR animal[Mesh] NOT (animal[Mesh] AND human[Mesh])))
5. 1-4 AND

Embase (via Embase.com interface):

1. ‘Education’/exp OR educat*:ab,ti OR ‘Learning’/exp OR learn*:ab,ti OR teach*:ab,ti
2. ‘Child’/exp OR ‘Adolescent’/exp OR child*:ab,ti OR youth:ab,ti OR teenage*:ab,ti OR adolescent*:ab,ti OR ‘Student’/exp OR student*:ab,ti OR ‘School’/exp OR school*:ab,ti
3. 'Developing country'/exp OR ‘developing countr*’:ab,ti OR ‘Low income country’/exp OR ‘Middle income country’/exp OR ‘low income countr*’:ab,ti OR ‘middle income countr*’:ab,ti
4. ‘meta analysis (topic)’/exp OR ‘meta analysis’/exp OR ‘meta analysis’:ab,ti OR ‘meta-analysis’:ab,ti OR ‘systematic review (topic)’/exp OR ‘systematic review’/exp OR ‘cochrane’:ab,ti OR ‘embase’:ab,ti OR ‘pubmed’:ab,ti OR ‘medline’:ab,ti OR ‘reference list’:ab,ti OR ‘reference lists’:ab,ti OR ‘bibliography’:ab,ti OR ‘bibliographies’:ab,ti OR ‘hand-search’:ab,ti OR ‘manual search’:ab,ti OR ‘relevant journals’:ab,ti OR ‘selection criteria’:ab,ti OR ‘data extraction’:ab,ti
5. 1-4 AND

**ERIC** (via Ovid interface):

1. Exp education/ OR educat*.ti,ab. OR exp learning/ OR learn*.ti,ab. OR teach*.ti,ab.
2. Exp developing nations/ OR developing countr*.ti,ab. OR low income countr*.ti,ab. OR middle income countr*.ti,ab.
3. Exp Meta Analysis/ OR meta analysis.ti,ab. OR meta-analysis.ti,ab. OR systematic review.ti,ab.
4. 1-3 AND

**3ie Database of Systematic Reviews** (via <http://www.3ieimpact.org/en/evidence/systematic-reviews/>) using the Advanced Search mode, entering the following search term in the Title field: education
