# Supplementary material for: An educational pathway and teaching materials for first aid training of children in sub-Saharan Africa based on the best available evidence
Source: BMC Public Health. 2020 Jun 3;20:836. doi: 10.1186/s12889-020-08857-5 (PMC7268765; doi:10.1186/s12889-020-08857-5)
Supplement: Supplementary file 5 — Additional file 5. Selection criteria research question 2 [file 12889_2020_8857_MOESM5_ESM.docx]

# Additional file 5: Selection criteria research question 2

**PICO:** ‘In primary and secondary school children (5-18 years) in low- and middle-income countries (Population), is the provision of instructional materials and/or the use of alternative pedagogical methods (Intervention) effective for improving knowledge, skills and attitude (Outcome) compared to not providing these materials and/or using an alternative form of these methods (Comparison)?’

Population: *Include*: children between the age of 5 and 18 years attending mainstream primary and secondary schools in low- and middle-income countries, as defined by the World Bank at the point in time that an intervention was carried out. *Exclude*: children attending vocational or postsecondary education (e.g. universities), children with special educational needs (e.g. due to learning difficulties or intellectual or physical disabilities), children with autism, children affected by armed conflicts, refugees, migrants, orphans, children living in high-income countries.

Intervention: as the evidence concerning the effectiveness of educational interventions was gathered in the view of developing teaching materials for first aid training to children in sub-Saharan Africa, the scope of the educational interventions of interest was narrowed to 3 categories: the provision of instructional materials, the use of alternative pedagogical methods, and structured pedagogy interventions. *Include*: provision of traditional hardware instructional materials (e.g. text books, flip-charts), use of ‘structured pedagogy interventions’ (i.e. a combination of newly developed structured lesson content and teacher training in delivering such materials whether or not in combination with materials for both teachers and students), use of alternative pedagogical methods (e.g. cooperative teaching, constructivist-based teaching, problem-solving method of teaching). Interventions delivered by teachers in a school setting or by teachers or volunteers during extracurricular classes or activities. *Exclude*: computer-based interventions, interventions delivered by parents or community members.

Comparison: *Include*: business as usual, including traditional lecturing method of teaching, other educational intervention.

Outcome: *Include*: knowledge, skills and/or attitude (willingness to show a certain behaviour) as a primary outcome. *Exclude*: behaviour as a primary or secondary outcome.

Study design: *Include*: a quantitative or mixed-methods systematic review that clearly describes its search strategy and selection criteria, that has critically appraised the methodological quality of the included individual studies, that uses clear statistical methods to analyze the quantitative data, and that clearly reports effect sizes
