# Supplementary material for: An educational pathway and teaching materials for first aid training of children in sub-Saharan Africa based on the best available evidence
Source: BMC Public Health. 2020 Jun 3;20:836. doi: 10.1186/s12889-020-08857-5 (PMC7268765; doi:10.1186/s12889-020-08857-5)

# Additional file 6: Description of consensus methods used during expert panel meeting

Reaching consensus concerning the educational pathway in first aid involved a multistep process.

Two weeks before the start of the meeting, 4 general preparatory questions (see below) were sent to the expert panel members together with the draft educational pathway. This allowed us to collect the opinion of every panel member independently of the opinions of the other panel members, in order to avoid a bandwagon effect or social desirability bias. In addition to these 4 more general questions, the preparatory document contained a ticking box exercise (see below) in which the panel members were asked to use their own experience to indicate the age at which children would be able to learn specific knowledge items, skills and attitudes. Only those learning objectives for which no or only a limited amount of studies was found were included in this exercise, and on the draft educational pathway no proposal was made for these objectives, not to influence the panel. The expert feedback was bundled, summarized and discussed with the chair in preparation of the meeting. The different answers per question, and different choices for the age ranges based on the ticking exercise, were presented on several PowerPoint slides for discussion during the meeting.

During the actual panel meeting, a formal discussion was held on the educational pathway (day 1) and effective teaching methods (day 2). On day 1, the project methods, the evidence that supported the pathway and the structure of the pathway itself were presented by the researchers. Following these presentations, the **4 general preparatory questions** were discussed. The first 2 questions (question 1 and 2 below) were formally discussed and consensus was obtained following discussion of disagreements (panel members who disagreed with the majority were specifically asked to provide their rationale). The other two questions (question 3 and 4 below) asked for the experts’ field experience and provided some context and background which could be useful for the following discussions. Following this discussion, **the age ranges for the different learning objectives** in the educational pathway were discussed one by one, with a specific focus on those learning objectives for which no or only limited evidence was available (based on the results of the ticking box exercise). For theses objectives, the expert feedback was compared with the proposal made in the draft version, and in case of large discrepancies between both, a discussion was held (again with specific explanation by panel members who disagreed with the proposal in the draft pathway). Next, a modified proposal was made by the chair, and consensus was sought by hand-raising. If consensus was not reached (i.e. no unanimous agreement), concerns were further addressed in a second discussion and the proposal was further adapted until full consensus was reached. The chair of the panel specifically paid attention to everyone in the group being heard.

On day 2 the evidence on **effective educational methods** was presented. Next, several educational methods (e.g. song, role-play, poem, puppetry, storytelling, lecturing, video) were discussed with the panel to determine their suitability and feasibility in the African context. In addition, the panel was asked to determine for which age groups these educational methods could be used, using a post-it exercise. Each panel member received two post-its, each with one specific educational method written down on it. For each of these two methods, the panel members were asked to write down the strengths and weaknesses and suitability/feasibility for the African context on the corresponding post-it note. Afterwards, the members shared their thoughts with the entire panel, and other panel members were given the chance to add extra strengths, weaknesses or suitability/feasibility issues. Next, the panel members were asked to stick their 2 post-its in one specific “age column” (5-8, 9-12, 13-18 years) on a flipchart, and their proposals were discussed with the full panel until consensus was reached. Finally, each panel member was asked to name his/her top three of most appropriate and successful teaching methods for each age group on a post-it note, independently from the other panel members. Post-it notes were collected by the researchers, who composed the overall top three of the full expert panel through vote-counting..

**Preparatory questions sent to the expert panel before the meeting:**

1. Content of the educational pathway and curriculum (General, Four main steps in first aid, Resuscitation and defibrillation, etc.): are there any topics that should be in- or excluded from the current proposal?
2. Age ranges: The current proposal of the educational pathway is based on age ranges per 2 years (5-6 yrs, 7-8 yrs, etc.): what would be an appropriate and feasible level of differentiation for the educational materials (= first aid manual for children)?
3. Field experience on first aid education in your context: How is first aid education (school context) and extracurricular activities (e.g. Red Cross club) organized in your context?
4. Field experience on teaching methods in your context: What works in education in your context (rural vs. urban context, teaching methods feasible in your context,…)?

**Extract ticking box exercise:**


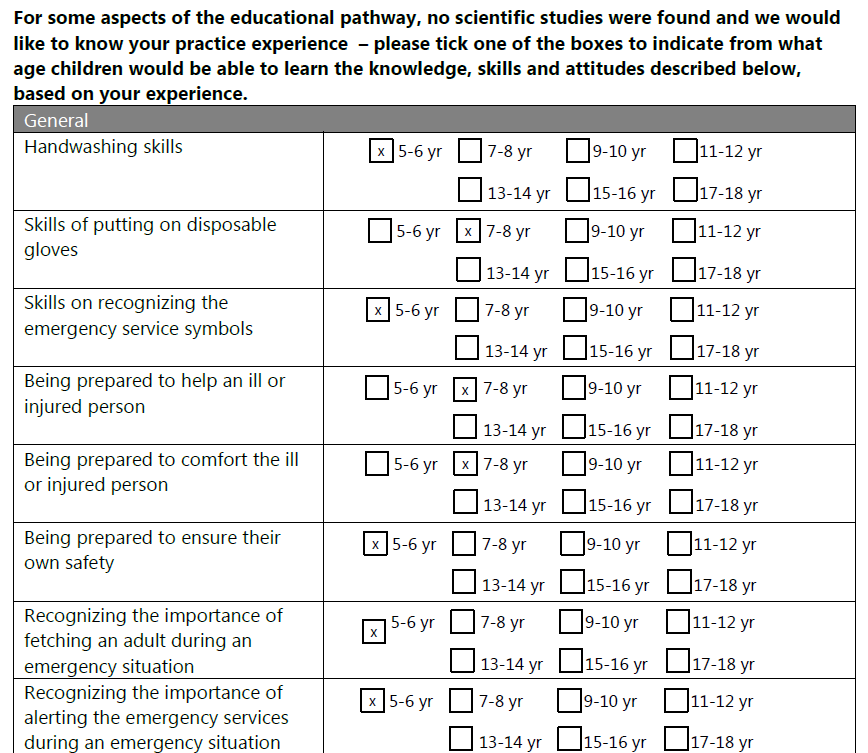

Supplement: Supplementary file 6 — Additional file 6. Description of consensus methods used during expert panel meeting [file 12889_2020_8857_MOESM6_ESM.docx]
