# Supplementary material for: An educational pathway and teaching materials for first aid training of children in sub-Saharan Africa based on the best available evidence
Source: BMC Public Health. 2020 Jun 3;20:836. doi: 10.1186/s12889-020-08857-5 (PMC7268765; doi:10.1186/s12889-020-08857-5)
Supplement: Supplementary file 7 — Additional file 7. Characteristics of included studies, synthesis of findings, risk of bias and certainty of evidence for research question 1 [file 12889_2020_8857_MOESM7_ESM.docx]

# Additional file 7: Characteristics of included studies, synthesis of findings, risk of bias and certainty of evidence for research question 1

*This overview includes all 58 available studies (including 25 of the 30 previously identified and published studies (Resuscitation 2015, 94: 8–22, but excluding 5 studies in the current analysis since it was decided not to include AED use in the African materials), and the 33 newly identified studies)*

## General principles

Characteristics of included studies

| **Author, year, Country** | **Study design** | **Population** | **Comparison/Risk factor** | **Remarks** |
| --- | --- | --- | --- | --- |
| Akca, 2016, Turkey | Observational: before after study | *Nr. of participants:*  100 students:   - Gender not reported   *Age range:*  Not reported  Mean ± SD:  17.1 ± 0.73 years | **Intervention:**  *Program:* Foreign body aspiration (ear, nose and respiratory tract) training  *Content:*  Frequency, causes, symptoms and first aid of foreign body aspiration.  Lecture + demonstration + hands-on training of first aid interventions.  *Duration:* 45 min | Outcomes were measured before and 1 month after training.  *Knowledge*: Self-developed questionnaire on foreign body aspiration knowledge |
| Ammirati, 2014, France | Experimental: non-randomized controlled trial | *Nr. of participants*: 285 students:   - 138 males - 147 females   Two arms:  Intervention n=140 Control n=145  *Age range:*  <6 years  Mean: 5.4 years  (SD not reported) | **Intervention:**  *Program*: First aid training  *Content*: Recognise unusual situation + call emergency number + describe situation.  *Duration:* Not reported.  **Control:**  No intervention. | Outcomes were measured two months after completion of FA training.  *Knowledge:* Children had to observe pictures and decide when it would be appropriate to make an emergency call  *Skills:* Ability to perform an emergency call   - Use telephone - Introduce yourself - Describe situation |
| Beskind, 2016, USA | Experimental: cluster-randomized controlled trial | *Nr. of participants:*  179 students:   - Gender not reported   Three arms:  Intervention1 n=69  Intervention2 n=56  Control n=54  *Age range:*  14-18 years | **Intervention:**  *Program:* chest compression only CPR (CCO-CPR) training  *Content:*  Intervention1: Brief video illustrating the steps to perform CCO-CPR.  Intervention2: Classroom training including a power-point presentation + demonstration + hands-on manikin training on CCO-CPR.  *Duration:*  Intervention1: 1.5 min  Intervention2: 20 min  **Control:**  *Program:* Sham video  *Content:* University of Arizona recruiting video  *Duration:* 1 minute | Outcomes were measured before, immediately after and 2 months after training.  *Skills*: Responsiveness + CPR quality (depth, compression rate) on manikin measured during 2 minutes  [only post-training and follow-up data were extracted] |
| Bollig, 2009, Norway | Experimental: randomized controlled trial | *Nr. of participants:* 228 students   - 126 males - 102 females   Two arms:  Intervention n=117 Control n=111  *Age range:*  6-7 years | **Intervention:**  *Program*: CPR training  *Content*: Assessing consciousness and breathing, wound treatment, bleeding, recovery position, behavior in emergency situations, emergency call, first aid scenarios. Lectures provided by instructor using a glove puppet.  *Duration*: 5 lessons (45 min each, one lesson per week)  **Control:**  No intervention. | Outcomes were measured immediately after and 6 months after training.  *Knowledge:* Emergency number  *Skills*: Test scenario with unconscious victim. Ability to:   - Assess consciousness - Assess breathing - Give emergency information - Recovery position - Airway management |
| Campbell, 2001, USA | Experimental: randomized controlled trial | *Nr. of participants:* 660 students:   - 51% males - 49% females   Two arms:  Intervention n=293 Control n=367  *Age range:*  11-16 years  Mean ± SD:  13 ± 1.11 years | **Intervention:**  *Program:* First aid and home safety training  *Content:* Emergency care, fever and first aid kit, bleeding, burns, fractures, dislocations and sudden illness, sports injury and prevention, poisoning, bites, stings and allergies, first aid review and household safety.  **Control:**  *Program:* Tobacco and alcohol prevention program  *Content:* Health effects of smoking and alcohol, peer pressure, decision making, societal influences and refusal skills.  *Duration (intervention + control):* 8 sessions (2 hours/session) spread over a 7-to 10-week period | Outcomes were measured before, immediately after and 1 year after training.  *Knowledge:*   - Emergency response procedures (check-call-care) - First aid kit   [only knowledge data for FA procedure were extracted]  *Skills:* Responses to two audio-recorded scenarios: scenario 1. severe wound from glass; scenario 2. severe burn injury from a toddler. Type and order of responses were scored:   - Check the scene and victim - Call 911 - Care for the victim   *Attitude:* First aid confidence |
| Celik, 2013, Turkey | Observational: before after study | *Nr. of participants:*  231 students:   - 170 high school - 61 university - Gender not reported   [Only data from high school students were extracted]  *Age range:*  15-18 years (n=170)  Grade 10-12 | **Intervention:**  *Program:* Foreign body aspiration (ear, nose and airway) training  *Content:* Frequency, causes, symptoms and first aid of foreign body aspiration.  Lecture (power-point) + 3 videos + hands-on dummy training for at least 5 students of each group while others watched.  *Duration:* 60 minute lecture + 5 minute video + 45 minute practice | Outcomes were measured before and 1 month after training.  *Knowledge*: Questionnaire on foreign body aspiration knowledge  *Attitude*: Self-efficacy |
| Connolly, 2007, UK | Experimental: controlled before after study  [extracted data are from experimental before after design without control] | *Nr. of participants:* 79 students:   - 47 males - 32 females   Two arms:  Intervention n=46  Control n=33  *Age range:*  10-12 years  Mean intervention:  11.8 years  Mean control:  12.7 years  (SD not reported) | **Intervention:**  *Program*: CPR training (‘ABC for life’ program)  *Content*: How to approach a patient, CPR and recovery position.  Video + small group lecture + demonstration + hands-on manikin training.  *Duration*: < 2 hours  **Control:**  No intervention. | Outcomes were measured before, immediately after (only intervention group) and 6 months after training.  *Knowledge:* 22 point multiple choice questionnaire (patient approach, basic life support and CPR) |
| Engeland, 2002, Norway | Experimental: randomized controlled trial | *Nr. of participants:* 893 students:   - 50% males - 50% females   Two arms:  Intervention n=741 Control n=152  *Age range:*  13-14 years | **Intervention:**  *Program:* first aid training  *Content:* Chest pain, cardiac arrest, epileptic seizure, injuries to the head or neck, burn injuries, internal bleeding, obstruction of the airways and drowning first aid.  Textbook + video.  *Duration:* Sessions spread over 6 months  **Control:**  No intervention. | Outcomes were measured before and 6 months after training.  *Knowledge:* 17- item first aid questionnaire  [knowledge data were not extracted, since no data on separate first aid topics were not available]  *Attitude:* Self-efficacy, willingness to provide and learn first aid |
| Frederick,  2000, UK | Experimental: controlled before after study | *Nr. of participants:* 1096 students   - Gender not reported   Two arms:  Intervention n=542  Control n=554  *Age range:*  10-11 years | **Intervention:**  *Program:* Injury Minimization Program for Schools (IMPS)  *Content:* Road safety, accidents in the home, fire, electricity, poisons, waterways, basic life support and resuscitation skills.  Lecture + video + hands-on manikin training + hospital/emergency department visit.  *Duration:* Not reported. Spread over 1 academic year  **Control:**  No intervention. | Outcomes were measured before and 5 months after training.  *Knowledge:* Self-developed quiz  *Skills:* Performance on a basic life support scenario. [only measured post intervention]  *Attitude:* Record observations of dangerous behavior in a video  [attitude data were not extracted]  [only post-training data were extracted] |
| Frederick, 2002, UK | Experimental: non-randomized controlled trial | *Nr. of participants:* 1096 students   - Gender not reported   Two arms:  Intervention n=542  Control n=554  *Age range:*  10-11 years | **Intervention**:  *Program:* Basic life support training (part of IMPS; see Frederick, 2000)  *Content:* Basic life support, call for help, recovery position, resuscitation.  Lecture + hands-on manikin training.  *Duration:* 70 minutes  **Control**:  No intervention. | Outcomes were measured 5 months after training.  *Skills*: Performance of basic life support and CPR on manikin |
| Lorem, 2008, Norway | Observational: case - control study | *Nr. of participants:* 102 students  + data available on family and friends [ only student data were extracted]  Two arms:  Intervention n=52:   - 43% males - 57% females   Control n=50:   - 34% males - 66% females   *Age range:*  12 years  Mean ± SD intervention:  12 ± 0.02 years  Mean ± SD control:  11.9 ± 0.05 years | **Intervention**:  *Program*: CPR training  *Content*: CPR video + information folder + hands-on manikin training.  *Duration*: 45 min (30 min DVD)  **Control**:  No intervention before testing. | Intervention: Outcomes were measured 1 week after training.  Control: Outcomes were measured before training.  *Skills:* Video based performance in scenario + CPR quality (depth, tidal volume) on manikin measured for 2 minutes |
| Luria, 2000, USA | Experimental: randomized controlled trial | *Nr. of participants:* 246 students:   - Gender not reported   Two arms:  Intervention   - n=122 (pre) - n=90 (post)   Control   - n=124 (pre) - n=91 (post)   *Age range*:  5-6 years | **Intervention:**  *Program*: Safety education program  *Content*: How to cross the street, call 911, avoid strangers.  Lecture + practical part + coloring book + rock concert reinforcing the teaching objectives.  *Duration*: half-day –  20 minutes per topic  **Control**:  No intervention. | Outcomes were measured before and 6 months after training.  *Knowledge:* Questionnaire consisting of 3 sections: how to cross the street, how to recognize an emergency and dial 911, and how to avoid strangers.  [only 911 data were extracted] |
| Meissner, 2012, Germany | Observational: before after study | *Nr. of participants*: 129 students:   - 57 males - 72 females   *Age range*:  14-15 years  Mean ± SD:  14.6 ± 1.4 years | **Intervention:**  *Program*: CPR, AED and healthy lifestyle training  *Content*: Sudden cardiac arrest, recovery position, CPR, AED use.  Lecture + demonstration + hands-on manikin training + workshops on healthy lifestyle and physical activity.  *Duration:* 45 minute lecture + 30 minute practice | Outcomes were measured before, immediately after (same day), and four months after training.  *Skills:* Checklist + CPR quality (frequency, depth, time until start of chest compressions) on manikin  *Attitude:* FA, health issues, and self-confidence assessment  [only skills + self-confidence data were extracted] |
| Plotnikoff, 1989, Australia | Experimental: controlled before after study | *Nr. of participants:*  45 students:   - Gender not reported   Two arms: Intervention n=20 Control n=25  *Age range:*  11-12 years | **Intervention**:  *Program:* CPR training (see Moore, 1992)  **Control**:  No intervention. | Outcomes were measured before, immediately after and 5 months after training.  *Knowledge:* 22-item CPR questionnaire  *Skills*: CPR performance on manikin  [only post-training and follow-up data were extracted] |
| Wilks, 2016, Australia | Observational: before after study | *Nr. of participants*: 107 students:   - 51 males - 56 females   *Age range*:  11-12 years  Mean ± SD:  11.4 ± 0.5 years | **Intervention:**  *Program*: First aid, CPR and beach safety training  *Content*:   - Beach safety - Leadership and team bonding exercises - First aid: sprains, bleeding, choking, heart attack, allergic reactions - Hands-on manikin training   *Duration:* 1 day | Outcomes were measured before, 1 week after, and 8 weeks after training.  *Knowledge:* 50-item quiz on emergency services and life-supporting first aid |
| Younas, 2006, UK | Experimental: non-randomized controlled trial | *Nr. of participants:*  59 students:   - Gender not reported   Two arms:  Intervention n=34  Control n=25  *Age range:*  13-16 years | **Intervention:**  *Program*: CPR/AED training  *Content*: CPR and AED use. Hands-on manikin training.  *Duration*: Not reported.  **Control:**  No intervention | Outcomes were measured 6 months after training.  *Skills*: 10-item checklist during cardiac arrest scenario |

**Synthesis of findings**

| **General** | | | | | |
| --- | --- | --- | --- | --- | --- |
| **Outcome** | | **Comparison** | **Effect Size** | **#studies, # participants** | **Reference** |
| *Knowledge* | | | | | |
| ***Call for help*** | | | | | |
| - 10-12 years | | | | | |
| Knowledge: seeking help | Pre vs post | | Statistically significant:  7 (15.2%) vs 36 (78.3%) §  RR:5.14 *£  (p<0.001)  *in favour of first aid training* | 1, 46 vs 46  (within subject) | Connolly, 2007 |
| - 11-12 years | | | | | |
| Knowledge: FA unconsciousness – send for help | Pre vs 1 week post | | Not statistically significant:  63 (59%) vs 64 (61%) §  RR: 1.07* £†  (p=0.382- overall p) | 1, 107 vs 102 (within subject) | Wilks, 2016 |
|  | Pre vs 8 weeks post | | Not statistically significant:  63 (59%) vs 54 (51%) §  RR: 0.87* £†  (p=0.382- overall p) | 1, 107 vs 105 (within subject) |  |
|  | 1 week post vs 8 weeks post | | Not statistically significant:  64 (61%) vs 54 (51%) §  RR: 0.82* £†  (p=0.382- overall p) | 1, 102 vs 105 (within subject) |  |
| ***Emergency call*** | | | | | |
| - 5-6 years | | | | | |
| Knowledge: emergency number (at least 2-point increase in test scores) | FA training vs no intervention | | Not statistically significant:  60 (67%) vs 57 (63%) §  RR: 1.06, 95%CI [0.86;1.32] ¥  (p=0.57) * | 1, 90 vs 91 | Luria, 2000 |
| Knowledge: How to dial emergency number (/16) |  |  | Not statistically significant:  Mean pre-post change, [range]: 4.15, [-13;-16] vs 3.66, [-8;-13]  MD: 0.49* £†  (p=0.41) |  |  |
| Knowledge: know when to call emergency number (3 situations) |  |  | Emergency situation1:  Statistically significant:  87 (62.1%) vs 12 (8.3%) §  RR: 7.51, 95%CI [4.30;13.11]*  (p<0.0001)  *In favour of first aid training*  Emergency situation 3:  Statistically significant:  93 (66.4%) vs 20 (13.8%) §  RR: 4.82, 95%CI [3.15;7.36]*  (p<0.0001)  *In favour of first aid training*  Emergency situation 2 (correct response = don’t call):  Not statistically significant:  105 (75%) vs 110 (75.9%)  RR: 0.99, 95%CI [0.87;1.13]*  (p=0.24) | 1, 140 vs 145 | Ammirati, 2014 |
| Knowledge: Describe the situation from photograph |  |  | Emergency situation 1:  Statistically significant:  95 (67.9%) vs 66 (45.5%) §  RR: 1.49, 95%CI [1.21;1.84]*  (p<0.001)  *In favour of first aid training*  Emergency situation 2:  Statistically significant:  100 (71.4%) vs 60 (41.4%) §  RR: 1.73, 95%CI [1.38;2.15]*  (p<0.0001)  *In favour of first aid training*  Emergency situation 3:  Statistically significant:  106 (75.7%) vs 87 (60.0%) §  RR: 1.26, 95%CI [1.07;1.48]*  (p=0.01)  *In favour of first aid training* |  |  |
| - 6-7 years | | | | | |
| Knowledge: Emergency number | FA training vs no intervention | | Statistically significant:  90 (77%) vs 18 (16%) §  RR: 4.74, 95%CI [3.07;7.32]*  (p<0.00001)  *In favour of first aid training* | 1, 117 vs 111  (immediately post) | Bollig, 2009 |
|  |  |  | Statistically significant:  58 (46%) vs 18 (16%) §  RR: 2.86, 95%CI [1.80;4.55]*  (p<0.00001)  *In favour of first aid training* | 1, 125 vs 111  (6 month follow-up) |  |
| - 10-12 years | | | | | |
| Knowledge: Emergency number | FA training vs no intervention | | Statistically significant:  3.0±1.0 vs 2.8±1.1  MD: 0.2, 95%CI [0.08;0.32]  (p=0.002)*  *in favour of first aid training* | 1, 542 vs 554 § (power analysis) | Frederick, 2000 |
|  | Pre vs immediately post | | Not statistically significant:  45 (97.8%) vs 46 (100%) §  RR: 1.02* £†  (p>0.05) | 1, 46 vs 46  (within subject) | Connolly, 2007 |
| - 11-12 years | | | | | |
| Knowledge: Emergency number | Pre vs 1 week post | | Not statistically significant:  0.88 ± 0.33 vs 0.95 ± 0.21  MD: 0.07* £†  (p=0.142- overall p) | 1, 107 vs 102 § (within subject) | Wilks, 2016 |
|  | Pre vs 8 weeks post | | Not statistically significant:  0.88 ± 0.33 vs 0.92 ± 0.27  MD: 0.04* £†  (p=0.142- overall p) | 1, 107 vs 105 § (within subject) |  |
|  | 1 week post vs 8 weeks post | | Not statistically significant:  0.95 ± 0.21 vs 0.92 ± 0.27  MD: -0.03* £†  (p=0.142 – overall p) | 1, 102 vs 105 § (within subject) |  |
| Knowledge: call emergency number + sit patient in comfortable position | Pre vs 1 week post | | Not statistically significant:  82 (77%) vs 91 (87%) §  RR: 1.16* £†  (p=0.718- overall p) | 1, 107 vs 102 (within subject) |  |
|  | Pre vs 8 weeks post | | Not statistically significant:  82 (77%) vs 85 (81%) §  RR: 1.06* £†  (p=0.718- overall p) | 1, 107 vs 105 (within subject) |  |
|  | 1 week post vs 8 weeks post | | Not statistically significant:  91 (87%) vs 85 (81%) §  RR: 0.91* £†  (p=0.718- overall p) | 1, 102 vs 105 (within subject) |  |
| - 15-18 years | | | | | |
| Knowledge: Emergency call | Pre vs post | | Not statistically significant:  168 (98.8%) vs 170 (100%) §  OR: ∞, 95%CI [0.19;∞] ¥  (p=0.5)* | 1, 170 vs 170  (within subject) | Celik, 2013 |
| - 16-18 years | | | | | |
| Knowledge: Emergency number | Pre vs post | | Not statistically significant:  98 (98%) vs 100 (100%) §  OR: ∞, 95%CI [0.19;∞] ¥  (p=0.5)* | 1, 100 vs 100  (within subject) | Akca, 2016 |
| *Skills* | | | | | |
| ***Call for help*** | | | | | |
| - 10-11 years | | | | | |
| Skills: Call for help | FA training vs no intervention | | Statistically significant:  140 (26%) vs 54 (9%) §  RR: 2.82, 95%CI [2.11;3.77]  (p<0.00001)*  *in favour of first aid training* | 1, 534 vs 581 §(power analysis) | Frederick, 2000  Frederick, 2002 |
| - 11-12 years | | | | | |
| Skills: Call for help | FA training vs no intervention | | 0 (0%) vs 0 (0%) §  RR: not estimable  statistical analysis not possible | 1, 20 vs 25 (post) | Plotnikoff, 1989 |
|  |  |  | 0 (0%) vs 0 (0%) §  RR: not estimable  statistical analysis not possible | 1, 20 vs 25 (5 month follow-up) |  |
| - 13-16 years | | | | | |
| Skills: Call for help | FA training vs no intervention | | Not statistically significant:  12 (79%) vs 4 (16%) §  RR: 2.21, 95%CI [0.81;6.04] ¥  (p=0.12)* | 1, 34 vs 25 | Younas, 2006 |
| - 14-15 years | | | | | |
| Skills: Call for help | Pre vs post | | Statistically significant:  22 (17%) vs 3 (2%) §  RR: 0.14* £  (p<0.001)  *in favour of first aid training* | 1, 129 vs 129 (within subject) | Meissner, 2012 |
|  | Post vs 4 month follow-up | | Statistically significant:  3 (2%) vs 11 (8.2%) §  RR: 3.67* £  (p=0.016)  *decline over time* |  |  |
| ***Emergency call*** | | | | | |
| - 5-6 years | | | | | |
| Skills: Use the telephone | FA training vs no intervention | | Statistically significant:  78 (55.7%) vs 12 (17.7%) §  RR: 3.16, 95%CI [1.85;5.39]*  (p<0.0001)  *In favour of first aid training* | 1, 140 vs 68 | Ammirati, 2014 |
| Skills: Introduce yourself, explain location |  |  | Statistically significant:  115 (82.1%) vs 23 (33.8%) §  RR: 2.43, 95%CI [1.73;3.42]*  (p<0.0001)  *In favour of first aid training* |  |  |
| Skills: Describe the situation during simulation exercise |  |  | Statistically significant:  125 (89.3%) vs 51 (75%) §  RR: 1.19, 95%CI [1.03;1.38]*  (p=0.01)  *In favour of first aid training* |  |  |
| - 6-7 years | | | | | |
| Skills: Provide correct information | FA training vs no intervention | | Statistically significant:  59 (50%) vs 7 (6%) §  RR: 8.00, 95%CI [3.82;16.75]*  (p<0.00001)  *in favour of first aid training* | 1, 117 vs 111  (immediately post) | Bollig, 2009 |
|  |  |  | Statistically significant:  86 (69%) vs 7 (6%) §  RR: 10.91, 95%CI [5.27;22.56]*  (p<0.00001)  *in favour of first aid training* | 1, 125 vs 111  (6 month follow-up) |  |
| - 10-12 years | | | | | |
| Skills: Call emergency number | *FA training vs no intervention* | | *Statistically significant:*  *36 (69%) vs 19 (38%) §*  *RR: 1.82, 95%CI [1.22;2.71]**  *(p=0.004)*  *in favour of first aid training* | 1, 52 vs 50 | Lorem, 2008 |
| - 11-16 years | | | | | |
| Skills: Call emergency number  (2 situations) | FA training vs sham intervention | | Scenario 1 (severe glass wound)  Statistically significant:  95 (65%) vs 119 (54%) §  RR: 1.20, 95%CI [1.01;1.42]*  (p=0.04)  *in favour of first aid training*  Scenario 2 (severe burn injury)  Not statistically significant:  50 (34%) vs 63 (29%) §  RR: 1.19, 95%CI [0.88;1.62]*¥  (p=0.244) | 1, 147 vs 221 | Campbell, 2001 |
| - 13-16 years | | | | | |
| Skills: Call emergency number + provide information | FA training vs no intervention | | Not statistically significant:  28 (82.4%) vs 22 (88%) §  RR: 094, 95%CI [0.76;1.16]  (p=0.54)* | 1, 34 vs 25 | Younas, 2006 |
| - 14-15 years | | | | | |
| Skills: Call emergency number | Pre vs post | | Statistically significant:  86 (67%) vs 95 (92%) §  RR: 1.37* £  (p<0.001)  *in favour of first aid training* | 1, 129 vs 103 (within subject) | Meissner, 2012 |
|  | Post vs 4 month follow-up | | Not statistically significant:  95 (92%) vs 94 (95.9%) §  RR: 1.04* £†  (p=0.75) | 1, 103 vs 98  (within subject) |  |
| - 14-18 years | | | | | |
| Skills: Call emergency number | Brief video vs no intervention | | Statistically significant:  49 (84.5%) vs 17 (37.0%) §  RR: 2.34, 95%CI [1.57;3.47]  (p<0.00001, still significant after Bonferroni correction p=0.0056)*  *in favour of first aid training* | 1, 58 vs 47 (post) | Beskind, 2016 |
|  | Classroom training vs no intervention | | Statistically significant:  40 (75.0%) vs 17 (37.0%) §  RR: 2.05, 95%CI [1.36;3.09]  (p<0.00001, still significant after Bonferroni correction p=0.0056)*  *in favour of first aid training* | 1, 54 vs 47 (post) |  |
|  | Brief video vs no intervention | | Not statistically significant:  45 (78.3%) vs 26 (56.3%) §  RR: 1.40, 95%CI [1.05;1.88]  (p=0.02, but no longer significant after Bonferroni correction p=0.0056)* | 1, 58 vs 47 (2 month follow-up) |  |
|  | Classroom training vs no intervention | | Not statistically significant:  40 (74.5%) vs 26 (56.3%) §  RR: 1.34, 95%CI [0.99,1.81]  (p=0.06)* | 1, 54 vs 47 (2 month follow-up) |  |
| Skills: Time to calling 911 (sec.) | Brief video vs no intervention | | Not statistically significant:  8.5±7.2 vs 9.5±10.99  MD: -1.0, 95%CI [-4.65;2.65]  (p=0.59)* | 1, 58 vs 47 (post) § |  |
|  | Classroom training vs no intervention | | Not statistically significant:  10.9±19.4 vs 9.5±10.99  MD: 1.40, 95%CI [-4.65;7.45] ¥  (p=0.65)* | 1, 54 vs 47 (post) § |  |
|  | Brief video vs no intervention | | Not statistically significant:  7.3±6.5 vs 8.4±9.1  MD -1.10, 95%CI [-4.19;1.99] ¥  (p=0.49) | 1, 58 vs 47 (2 month follow-up) § |  |
|  | Classroom training vs no intervention | | Statistically significant:  5.5±5.5 vs 8.4±9.1  MD: -2.90, 95%CI [-5.89,0.09] ¥  (p=0·06, but significant after Bonferroni correction)  *in favour of first aid training* | 1, 54 vs 47 (2 month follow-up) § |  |
| *Attitude* | | | | | |
| - 13-14 years | | | | | |
| Attitude: Willingness to help  (change after vs before intervention) | FA training vs no intervention | | Girls  Not statistically significant:  Mean pre-post change: 3.9 vs -0.4  MD: 4.3, 95%CI [-1.6;10.2] ¥  (p>0.05)  Boys  Statistically significant:  Mean pre-post change: 2.4 vs -5.0  MD: 7.4, 95%CI [1.4;13.5]  (p<0.05)  *in favour of first aid training* | 1, 741 vs 152 | Engeland, 2002 |

Mean ± SD (unless otherwise indicated), MD: mean difference, RR: risk ratio, OR: odds ratio, RD: risk difference

* calculations done by the reviewer using Revman, R software, or Excel

£ No raw data available and CI cannot be calculated.

¥ Imprecision (large variability of results)

† Imprecision (lack of data)

§ Imprecision (limited sample size or low number of events)

λ data extracted from graph

Quality of evidence

***Experimental studies***

| **Author, Year** | **Lack of allocation concealment** | **Lack of blinding** | **Incomplete accounting of outcome events** | **Selective outcome reporting** | **Other limitations** |
| --- | --- | --- | --- | --- | --- |
| Ammirati, 2014 | Randomization: yes, no randomization  Allocation concealment: not applicable | Unclear | No | No | No pre-test/baseline assessment |
| Beskind, 2016 | Randomization:no, a cluster-randomization was performed  Allocation concealment: no, allocation was concealed | No, statistician was blinded, outcomes were recorded on manikin or simple yes/no action checks | Yes, larger loss to follow-up in brief video compared to others | No | / |
| Bollig, 2009 | Randomization and allocation concealment: unclear, not specified in the article | Unclear if those recording and analyzing outcomes were aware of the arm to which participants were allocated | No | No | No pre-test/baseline assessment |
| Campbell, 2001 | Randomization and allocation concealment: unclear, not specified in the article | No | Yes, considerable loss to follow-up | Yes, data from some time-points not reported | No pre-test/baseline assessment |
| Connolly, 2007 | Randomization: yes, no randomization  Allocation concealment: not applicable | No | No (loss to follow-up similarly in both groups) | No | Within subject design (no control available for extracted data); possible incorrect statistical analysis (data analyzed using Student’s t-test, incorrect for within subject design) |
| Engeland, 2002 | Randomization and allocation concealment: unclear, not specified in the article | No (no information in paper about blinding of those analyzing outcomes, but only questionnaires were used, which is an objective outcome measurement) | No, intention-to-treat analysis was performed | No | Pre-post change scores were used in the statistical analysis, but control and intervention group were not well matched (very different pre-training scores and similar post-training scores) |
| Frederick, 2000 | Randomization: yes, no randomization  Allocation concealment: not applicable | Yes, assessors were un-blinded | No | No | / |
| Frederick, 2002 | Randomization: yes, no randomization  Allocation concealment: not applicable | Yes, assessors were un-blinded | No | No | No pre-test/baseline assessment |
| Luria, 2000 | Randomization and allocation concealment: unclear, not specified in the article | No (no information about blinding of those analyzing outcomes, but only questionnaires were used, which is an objective outcome measurement) | Yes, considerable loss to follow-up, although similarly in both groups | No |  |
| Plotnikoff, 1989 | Randomization: yes, no randomization  Allocation concealment: not applicable | Unclear if those recording and analyzing outcomes were aware of the arm to which participants were allocated | No | No | / |
| Younas, 2006 | Randomization: yes, no randomization  Allocation concealment: not applicable | Unclear if those recording and analyzing outcomes were aware of the arm to which participants were allocated | No | No | No pre-test/baseline assessment |

***Observational studies***

| **Author, Year** | **Inappropriate eligibility criteria** | **Inappropriate methods for exposure and outcome variables** | **Not controlled for confounding** | **Incomplete or inadequate follow-up** | **Other limitations** |
| --- | --- | --- | --- | --- | --- |
| Akca, 2016 | Yes, no inclusion of control population | No | Yes | No | / |
| Celik, 2013 | Yes, no inclusion of control population | No, a questionnaire was used | Yes, not controlled for confounding factors (i.e. previous history with choking infants/children) | No | / |
| Lorem, 2008 | No | No (not applicable for exposure variables; no flawed measurement of outcome variables) | Yes | No | No pre-test/baseline assessment |
| Meissner, 2012 | Yes, no inclusion of passive control population | No, a questionnaire and checklist were used | No, controlled for age, height, weight, BMI and gender | Yes, considerable drop-out | Yes, questionable number of participants: 72 vs 59 (girls vs. boys) is more than total of 103 tested post intervention |
| Wilks, 2016 | Yes, no inclusion of control population | No, a questionnaire was used | Unclear, statistics not reported | No | / |

**Certainty of the body of evidence**

KNOWLEDGE

|  | **Initial grading Low [C]** | Downgrading due to |
| --- | --- | --- |
| **Limitations of study design** | -1 | See table ‘Quality of evidence’ |
| **Imprecision** | -1 | Limited sample sizes/low number of events + lack of data + large variability of results |
| **Inconsistency** | 0 |  |
| **Indirectness** | 0 |  |
| **Publication bias** | 0 |  |
| **QUALITY (GRADE)** | **Final grading Very low [D]** |  |

SKILLS

|  | **Initial grading Low [C]** | Downgrading due to |
| --- | --- | --- |
| **Limitations of study design** | -1 | See table ‘Quality of evidence’ |
| **Imprecision** | -1 | Limited sample sizes/low number of events + lack of data + large variability of results |
| **Inconsistency** | 0 |  |
| **Indirectness** | 0 |  |
| **Publication bias** | 0 |  |
| **QUALITY (GRADE)** | **Final grading Very low [D]** |  |

ATTITUDE

|  | **Initial grading High [A]** | Downgrading due to |
| --- | --- | --- |
| **Limitations of study design** | -1 | See table ‘Quality of evidence’ |
| **Imprecision** | -1 | Large variability of results |
| **Inconsistency** | 0 |  |
| **Indirectness** | 0 |  |
| **Publication bias** | 0 |  |
| **QUALITY (GRADE)** | **Final grading Low [C]** |  |

| **Conclusion** | *KNOWLEDGE*  ***Call for help***  There is limited evidence neither in favour of FA training nor the control. A statistically significant increase of call for help knowledge using FA training could not be demonstrated in   - 11-12 year olds: pre to 1w post, and pre to 8w follow-up (Wilks, 2016)   Note: call for help knowledge was shown not to decline over time in   - 11-12 year olds: 1w post to 8w post (Wilks, 2016)   There is limited evidence in favour of FA training. It was shown that FA training resulted in a statistically significant increase of call for help knowledge in   - 10-12 year olds: pre to post (Connolly, 2007)   Evidence is of very low quality and results of these studies are imprecise due to limited sample sizes, low number of events and lack of data.  ***Emergency call***  There is limited evidence neither in favour of FA training nor the control. A statistically significant increase of emergency call knowledge using FA training could not be demonstrated in   - 5-6 year olds: compared to no intervention (Luria, 2000) - 10-12 year olds: pre to post (Connolly, 2007) - 11-12 year olds: pre to post, and pre to 8w follow-up (Wilks, 2016) - 15-18 year olds: pre to post (Celik, 2013) - 16-18 year olds: pre to post (Akca, 2016)   Note: knowledge scores were already very high in all pre to post studies, thus leaving little room for improvement  There is limited evidence in favour of FA training. It was shown that FA training resulted in a statistically significant increase of emergency call knowledge in   - 5-6 year olds: compared to no intervention (Ammirati, 2014) - 6-7 year olds: compared to no intervention (Bollig, 2009) - 10-12 year olds: compared to no intervention (Frederick, 2000)   Note: emergency call knowledge was shown not to decline over time in   - 11-12 year olds: 1w post to 8w post (Wilks, 2016)   Evidence is of very low quality and results of these studies are imprecise due to limited sample sizes, low number of events, lack of data and large variability of results.  *SKILLS*  ***Call for help***  There is limited evidence neither in favour of FA training nor the control. A statistically significant increase of call for help skills could not be demonstrated in   - 11-12 year olds: compared to no intervention (Plotnikoff, 1989) - 13-16 year olds: compared to no intervention (Younas, 2006)   There is limited evidence in favour of FA training. It was shown that FA training resulted in a statistically significant increase of call for help skills in   - 10-11 year olds: compared to no intervention (Frederick, 2000) - 14-15 year olds: pre to post (Meissner, 2012)   Note: call for help skills were shown to decline over time in   - 14-15 year olds: post to 4m follow-up (Meissner, 2012)   ***Emergency call***  There is limited evidence neither in favour of FA training nor the control. A statistically significant increase of emergency call skills could not be demonstrated in   - 11-16 year olds: compared to sham intervention (Campbell, 2001) – scenario 2 - 13-16 year olds: compared to no intervention (Younas, 2006) - 14-18 year olds: compared to no intervention (Beskind, 2016) – time to calling 911   There is limited evidence in favour of FA training. It was shown that FA training resulted in a statistically significant increase of emergency call skills in   - 5-6 year olds: compared to no intervention (Ammirati, 2014) - 6-7 year olds: compared to no intervention (Bollig, 2009) - 10-12 year olds: compared to no intervention (Lorem, 2008) - 11-16 year olds: compared to sham intervention (Campbell, 2001) – scenario 1 - 14-15 year olds: pre to post (Meissner, 2012) - 14-18 year olds: compared to no intervention (Beskind, 2016) – call emergency number   Note: emergency call skills were shown not to decline over time in   - 14-15 year olds: post to 4m follow-up (Meissner, 2012)   Evidence is of very low quality and results of these studies are imprecise due to limited sample sizes, low number of events, lack of data and large variability of results.  *ATTITUDES*  There is limited evidence neither in favour of FA training nor the control. A statistically significant increase of willingness to help using FA training could not be demonstrated in   - 13-14 year old girls: compared to no intervention (Engeland, 2002)   There is limited evidence in favour of FA training. It was shown that FA training resulted in a statistically significant increase of willingness to help in   - 13-14 year old boys: compared to no intervention (Engeland, 2002)   Evidence is of low quality and results of this study are imprecise due to large variability of results. |
| --- | --- |
| **Reference(s)** | **Articles**  Akca SO. *The effect of foreign body aspiration training on the knowledge level of pupils.* Braz J Otorhinolaryngology 2016, 82(4): 408-415.  Ammirati C, Gagnayre R, Amsallem C, Nemitz B, Gignon M. *Are schoolteachers able to teach first aid to children younger than 6 years?* A comparative study. BMJ Open 2014, 4**,** e005848.  Beskind DL, Stolz U, Thiede R, Hoyer R, Burns W, Brown J, Ludgate M, Tiutan T, Shane R, McMorrow D, Pleasants M, Panchal AR. *Viewing a bried chest-compression-only CPR video improves bystander CPR performance and responsiveness in high school students: a cluster randomized trial.* Resuscitation 2016, 104: 28-33.  Bollig G, Wahl HA, Svendsen MV. *Primary school children are able to perform basic life-savind first aid measures.* Resuscitation 2009, 80: 689-692.  Campbell NR, Ayala GX, Litrownik AJ, Slymen DJ, Zavala F, Elder JP. *Evaluation of a first aid and home safety program for hispanic migrant adolescents.* American Journal of Preventive Medicine 2001, 20(4): 258-265.  Çelik N, Arikan D. *The effect of the training given to the child development students about foreign body aspiration upon their knowledge levels.* International Journal of Pediatric Otorhinolaryngology 2013, 77: 1811-1817.  Connolly M, Toner P, Connolly D, McCluskey DR. *The ‘ABC for life’ programme – Teaching basic life support in schools.* Resuscitation 2007, 72: 270-279.  Engeland A, Reysamb E, Smedslund G, Sogaard AJ. *Effects of first-aid training in junior high schools.* Injury control and safety promotion 2002, 9(2): 99-106.  Frederick K, Bixby E, Orzel MN, Stewart-Brown S, Willett K. *An evaluation of the effectiveness of the injury minimization programme for schools (IMPS).* Injury prevention 2000, 6: 92-95.  Frederick K, Bixby E, Orzel MN, Stewart-Brown S, Willet K. *Will changing the emphasis from ‘pulseless’ to ‘no signs of circulation’ improve the recall scores for effective life support skills in children?* Resuscitation 2002, 55: 255-261.  Lorem T, Palm A, Wik L. *Impact of a self-instruction CPR kit on 7^th^ graders’ and adults’ skills and CPR performance.* Resuscitation 2008, 79: 103-108.  Luria JW, Smith GA, Chapman JI. *An evaluation of a safety education program for kindergarten and elementary school children.* Arch pediatr adolesc med 2000, 154: 227-231.  Meissner TM, Kloppe C, Hanefeld C. *Basic life support skills of high school students before and after cardiopulmonary resuscitation training: a longitudinal investigation.* Scand J Trauma Resusc Emerg Med 2012, 20**,** 31.  Plotnikoff R, Moore PJ. *Retention of cardiopulmonary resuscitation knowledge and skills by 11- and 12-year-old children.* The medical Journal of Asutralia 1989, 150: 297-302.  Wilks J, Kanasa H, Pendergast D, Clark K. *Emergency response readiness for primary school children.* Aust Health Rev 2016.  Younas S, Raynes A, Morton S, Mackway-Jones K. *An evaluation of the effectiveness of the opportunities for resuscitation and citizen safety (ORCS) defibrillator training programme designed for older school children.* Resuscitation 2006, 71: 222-228.  **Systematic reviews**  De Buck E, Van Remoortel H, Dieltjens T, Verstraeten H, Clarysse M, Moens O, Vandekerckhove P. *Evidence-based educational pathway for the integration of first aid training in school curricula.* Resuscitation 2015, 94**,** 8-22.  Dieltjens T, De Buck E, Verstraeten H, Adriaenssens L, Clarysse M, Moens O, Devreker A, Bastiaen M, Claessens C, Verhelst K. *Evidence-based recommendations on automated external defibrillator training for children and young people in Flanders-Belgium.* Resuscitation 2013, 84**,** 1304-9.  He Z, Wynn P, Kendrick D. *Non-resuscitative first-aid training for children and laypeople: a systematic review.* Emerg Med J 2014, 31(9):763-8.  Lenson S, Mills J. *First aid knowledge retention in school children: A review of the literature.* Australasian Journal of Paramedicine 2016, 13.  Plant N, Taylor K. *How best to teach CPR to schoolchildren: a systematic review.* Resuscitation 2013, 84(4):415-21.  Reveruzzi B, Buckley L, Sheehan M. *School-Based First Aid Training Programs: A Systematic Review.* J Sch Health 2016, 86(4):266-72. |

## Four steps in first aid

Characteristics of included studies

| **Author, year, Country** | **Study design** | **Population** | **Comparison/Risk factor** | **Remarks** |
| --- | --- | --- | --- | --- |
| Akca, 2016, Turkey | Observational: before after study | *Nr. of participants:*  100 students:   - Gender not reported   *Age range:*  Not reported  Mean ± SD:  17.1 ± 0.73 years | **Intervention:**  *Program:* Foreign body aspiration (ear, nose and respiratory tract) training  *Content:*  Frequency, causes, symptoms and first aid of foreign body aspiration.  Lecture + demonstration + hands-on training of first aid interventions.  *Duration:* 45 min | Outcomes were measured before and 1 month after training.  *Knowledge*: Self-developed questionnaire on foreign body aspiration knowledge |
| Ammirati, 2014, France | Experimental: non-randomized controlled trial | *Nr. of participants*: 285 students:   - 138 males - 147 females   Two arms:  Intervention n=140 Control n=145  *Age range:*  <6 years  Mean: 5.4 years  (SD not reported) | **Intervention:**  *Program*: First aid training  *Content*: Recognise unusual situation + call emergency number + describe situation.  *Duration:* Not reported.  **Control:**  No intervention. | Outcomes were measured two months after completion of FA training.  *Knowledge:* Children had to observe pictures and decide when it would be appropriate to make an emergency call  *Skills:* Ability to perform an emergency call   - Use telephone - Introduce yourself - Describe situation |
| Beskind, 2016, USA | Experimental: cluster-randomized controlled trial | *Nr. of participants:*  179 students:   - Gender not reported   Three arms:  Intervention1 n=69  Intervention2 n=56  Control n=54  *Age range:*  14-18 years | **Intervention:**  *Program:* chest compression only CPR (CCO-CPR) training  *Content:*  Intervention1: Brief video illustrating the steps to perform CCO-CPR.  Intervention2: Classroom training including a power-point presentation + demonstration + hands-on manikin training on CCO-CPR.  *Duration:*  Intervention1: 1.5 min  Intervention2: 20 min  **Control:**  *Program:* Sham video  *Content:* University of Arizona recruiting video  *Duration:* 1 minute | Outcomes were measured before, immediately after and 2 months after training.  *Skills*: Responsiveness + CPR quality (depth, compression rate) on manikin measured during 2 minutes  [only post-training and follow-up data were extracted] |
| Bohn, 2012, Germany | Experimental: controlled before after study  [extracted data are from experimental before after design without control] | *Nr. of participants:*  433 students   - 223 males - 210 females   Three arms:  Intervention 1+2 n=251  Control n=182  *Age range:*  10-13 years  Two age groups:  10 year n=214  13 year n=219 | **Intervention:**  *Program:* CPR training  Intervention1: Annual training  Intervention2: Biannual training  *Content:* CPR lecture + hands-on manikin training. Focus on chest compression over ventilation.  *Duration:* 1 hour lecture + 2 hours practice  **Control:**  No intervention. | Outcomes were measured before, 1 year after (only intervention group 1+2), and two years after training.  [control group data were not extracted]  *Knowledge:* 11-item CPR questionnaire  *Skills*: CPR quality (depth, rate, tidal volume, ventilation frequency) on manikin measured during 5 minutes  [only pre-post (1 year after training) data were extracted for knowledge and skills]  *Attitude:* CPR self-efficacy, willingness, and confidence  Remark: This study includes the same data as Lukas, 2016 (see reference list).  [only data from Bohn extracted as it is more relevant for PICO] |
| Bollig, 2009, Norway | Experimental: randomized controlled trial | *Nr. of participants:* 228 students   - 126 males - 102 females   Two arms:  Intervention n=117 Control n=111  *Age range:*  6-7 years | **Intervention:**  *Program*: CPR training  *Content*: Assessing consciousness and breathing, wound treatment, bleeding, recovery position, behavior in emergency situations, emergency call, first aid scenarios. Lectures provided by instructor using a glove puppet.  *Duration*: 5 lessons (45 min each, one lesson per week)  **Control:**  No intervention. | Outcomes were measured immediately after and 6 months after training.  *Knowledge:* Emergency number  *Skills*: Test scenario with unconscious victim. Ability to:   - Assess consciousness - Assess breathing - Give emergency information - Recovery position - Airway management |
| Campbell, 2001, USA | Experimental: randomized controlled trial | *Nr. of participants:* 660 students:   - 51% males - 49% females   Two arms:  Intervention n=293 Control n=367  *Age range:*  11-16 years  Mean ± SD:  13 ± 1.11 years | **Intervention:**  *Program:* First aid and home safety training  *Content:* Emergency care, fever and first aid kit, bleeding, burns, fractures, dislocations and sudden illness, sports injury and prevention, poisoning, bites, stings and allergies, first aid review and household safety.  **Control:**  *Program:* Tobacco and alcohol prevention program  *Content:* Health effects of smoking and alcohol, peer pressure, decision making, societal influences and refusal skills.  *Duration (intervention + control):* 8 sessions (2 hours/session) spread over a 7-to 10-week period | Outcomes were measured before, immediately after and 1 year after training.  *Knowledge:*   - Emergency response procedures (check-call-care) - First aid kit   [only knowledge data for FA procedure were extracted]  *Skills:* Responses to two audio-recorded scenarios: scenario 1. severe wound from glass; scenario 2. severe burn injury from a toddler. Type and order of responses were scored:   - Check the scene and victim - Call 911 - Care for the victim   *Attitude:* First aid confidence |
| Celik, 2013, Turkey | Observational: before after study | *Nr. of participants:*  231 students:   - 170 high school - 61 university - Gender not reported   [Only data from high school students were extracted]  *Age range:*  15-18 years (n=170)  Grade 10-12 | **Intervention:**  *Program:* Foreign body aspiration (ear, nose and airway) training  *Content:* Frequency, causes, symptoms and first aid of foreign body aspiration.  Lecture (power-point) + 3 videos + hands-on dummy training for at least 5 students of each group while others watched.  *Duration:* 60 minute lecture + 5 minute video + 45 minute practice | Outcomes were measured before and 1 month after training.  *Knowledge*: Questionnaire on foreign body aspiration knowledge  *Attitude*: Self-efficacy |
| Connolly, 2007, UK | Experimental: controlled before after study  [extracted data are from experimental before after design without control] | *Nr. of participants:* 79 students:   - 47 males - 32 females   Two arms:  Intervention n=46  Control n=33  *Age range:*  10-12 years  Mean intervention:  11.8 years  Mean control:  12.7 years  (SD not reported) | **Intervention:**  *Program*: CPR training (‘ABC for life’ program)  *Content*: How to approach a patient, CPR and recovery position.  Video + small group lecture + demonstration + hands-on manikin training.  *Duration*: < 2 hours  **Control:**  No intervention. | Outcomes were measured before, immediately after (only intervention group) and 6 months after training.  *Knowledge:* 22 point multiple choice questionnaire (patient approach, basic life support and CPR) |
| Creutzfeldt, 2013, Sweden | Experimental: before after study | *Nr. of participants*: 36 students   - 16 males - 20 females - 12 Swedish students - 24 USA students   *Age range:*  15-16 years  Grade 10 n=35 Grade 11 n=1 | **Intervention:**  *Program*: Virtual reality CPR training  *Content*: A training session including 3-4 emergency scenarios in virtual environment + feedback from instructor.  Swedish group: Identical to American group + a second (identical) session 6 months later.  *Duration:* 90-120 min | Outcomes were measured before (only self-efficacy and engagement mode) and immediately after training (all outcomes).  *Attitude:* Self-efficacy, concentration, perceptions regarding feasibility of training, confidence, engagement mode, mental strain, feelings/perceptions about having to act in an emergency situation  [only self-efficacy and confidence were extracted] |
| Engeland, 2002, Norway | Experimental: randomized controlled trial | *Nr. of participants:* 893 students:   - 50% males - 50% females   Two arms:  Intervention n=741 Control n=152  *Age range:*  13-14 years | **Intervention:**  *Program:* first aid training  *Content:* Chest pain, cardiac arrest, epileptic seizure, injuries to the head or neck, burn injuries, internal bleeding, obstruction of the airways and drowning first aid.  Textbook + video.  *Duration:* Sessions spread over 6 months  **Control:**  No intervention. | Outcomes were measured before and 6 months after training.  *Knowledge:* 17- item first aid questionnaire  [knowledge data were not extracted, since no data on separate first aid topics were not available]  *Attitude:* Self-efficacy, willingness to provide and learn first aid |
| Frederick,  2000, UK | Experimental: controlled before after study | *Nr. of participants:* 1096 students   - Gender not reported   Two arms:  Intervention n=542  Control n=554  *Age range:*  10-11 years | **Intervention:**  *Program:* Injury Minimization Program for Schools (IMPS)  *Content:* Road safety, accidents in the home, fire, electricity, poisons, waterways, basic life support and resuscitation skills.  Lecture + video + hands-on manikin training + hospital/emergency department visit.  *Duration:* Not reported. Spread over 1 academic year  **Control:**  No intervention. | Outcomes were measured before and 5 months after training.  *Knowledge:* Self-developed quiz  *Skills:* Performance on a basic life support scenario. [only measured post intervention]  *Attitude:* Record observations of dangerous behavior in a video  [attitude data were not extracted]  [only post-training data were extracted] |
| Frederick, 2002, UK | Experimental: non-randomized controlled trial | *Nr. of participants:* 1096 students   - Gender not reported   Two arms:  Intervention n=542  Control n=554  *Age range:*  10-11 years | **Intervention**:  *Program:* Basic life support training (part of IMPS; see Frederick, 2000)  *Content:* Basic life support, call for help, recovery position, resuscitation.  Lecture + hands-on manikin training.  *Duration:* 70 minutes  **Control**:  No intervention. | Outcomes were measured 5 months after training.  *Skills*: Performance of basic life support and CPR on manikin |
| Joshi, 2012, UK | Experimental: non-randomized controlled trial | *Nr. of participants:*  292 students:   - 148 males - 144 females   Two arms:  Intervention n=198   - 104 males - 94 females   Control n=94   - 44 males - 50 females   *Age range:*  10-11 years | **Intervention:**  *Program:* Recovery position training  *Content:* A range of skills, with a small focus on the recovery position.  *Duration:* 2 hours (10 minutes recovery position  **Control:**  No intervention. | Outcomes were measured before and 3 months after training.  *Knowledge:* 10-item questionnaire  [knowledge data were not extracted – not relevant]  *Skills*: Performance checklist + video on recovery position (only measured post) |
| Lester, 1996, UK | Observational: case series study | *Nr. of participants:* 41 students:   - 17 males - 24 females   *Age range:*  11-12 years | **Intervention:**  *Program*: CPR training  *Content*: CPR + ethics.  Lecture + video + hands-on manikin training.  *Duration*: 3 sessions  (1 hour per session) | Outcomes were measured immediately after and 9 days (only skills) after training.  *Knowledge:* Multiple choice CPR questionnaire  *Skills*: Performance checklist first aid + CPR on manikin  *Attitude:* CPR attitudes  [only attitude data were extracted – case series] |
| Lester, 1997, UK | Experimental: non-randomized controlled trial  [extracted data are from experimental case series design without control as no passive control group was present] | *Nr. of participants:* 243 students:   - 128 males - 115 females   Two arms:  Intervention1 n=106  Intervention2 n=137  [Intervention2 data were not included in the analysis]  *Age range:*  11-12 years | **Intervention:**  *Program*: CPR training  Intervention1:  *Content*: CPR + ethics.  Lecture + video + hands-on manikin training.  *Duration*: 3 sessions  (1 hour per session)  Intervention2:  *Program*: CPR training (teacher + pupils). | Outcomes were measured immediately after training.  *Knowledge:* 10-point CPR multiple choice questionnaire  *Skills*: Performance checklist first aid + CPR on manikin  *Attitude:* CPR confidence, willingness, reasons not to  [only attitude data were extracted – case series] |
| Lorem, 2008, Norway | Observational: case - control study | *Nr. of participants:* 102 students  + data available on family and friends [ only student data were extracted]  Two arms:  Intervention n=52:   - 43% males - 57% females   Control n=50:   - 34% males - 66% females   *Age range:*  12 years  Mean ± SD intervention:  12 ± 0.02 years  Mean ± SD control:  11.9 ± 0.05 years | **Intervention**:  *Program*: CPR training  *Content*: CPR video + information folder + hands-on manikin training.  *Duration*: 45 min (30 min DVD)  **Control**:  No intervention before testing. | Intervention: Outcomes were measured 1 week after training.  Control: Outcomes were measured before training.  *Skills:* Video based performance in scenario + CPR quality (depth, tidal volume) on manikin measured for 2 minutes |
| Luria, 2000, USA | Experimental: randomized controlled trial | *Nr. of participants:* 246 students:   - Gender not reported   Two arms:  Intervention   - n=122 (pre) - n=90 (post)   Control   - n=124 (pre) - n=91 (post)   *Age range*:  5-6 years | **Intervention:**  *Program*: Safety education program  *Content*: How to cross the street, call 911, avoid strangers.  Lecture + practical part + coloring book + rock concert reinforcing the teaching objectives.  *Duration*: half-day –  20 minutes per topic  **Control**:  No intervention. | Outcomes were measured before and 6 months after training.  *Knowledge:* Questionnaire consisting of 3 sections: how to cross the street, how to recognize an emergency and dial 911, and how to avoid strangers.  [only 911 data were extracted] |
| Meissner, 2012, Germany | Observational: before after study | *Nr. of participants*: 129 students:   - 57 males - 72 females   *Age range*:  14-15 years  Mean ± SD:  14.6 ± 1.4 years | **Intervention:**  *Program*: CPR, AED and healthy lifestyle training  *Content*: Sudden cardiac arrest, recovery position, CPR, AED use.  Lecture + demonstration + hands-on manikin training + workshops on healthy lifestyle and physical activity.  *Duration:* 45 minute lecture + 30 minute practice | Outcomes were measured before, immediately after (same day), and four months after training.  *Skills:* Checklist + CPR quality (frequency, depth, time until start of chest compressions) on manikin  *Attitude:* FA, health issues, and self-confidence assessment  [only skills + self-confidence data were extracted] |
| Moore, 1992, Australia | Experimental: non-randomized controlled trial | *Nr. of participants:* 102 students:   - Gender not reported   Two arms:  Intervention n=51  Control n=51  *Age range:*  11-12 years | **Intervention**:  *Program*: CPR training  *Content*: CPR video + demonstration + hands-on manikin training.  *Duration*: 140 min  **Control**:  No intervention. | Outcomes were measured 5 years after training.  *Knowledge:* 14-item multiple choice questionnaire  *Skills*: Performance checklist on manikin CPR + CPR quality (ventilations) measured for 2 minutes |
| Petric, 2013, Croatia | Observational: cohort study  [extracted data are from observational cross-sectional design] | *Nr. of participants:*  301 students   - 143 males - 158 females   +361 parents [only student data were extracted]  *Age range:*  12-15 years  Median: 13 years | **No intervention.** | The following outcomes were measured:  *Attitude*: Basic life support training + willingness and fear to apply BLS  [only willingness and fear to apply BLS data were extracted] |
| Plotnikoff, 1989, Australia | Experimental: controlled before after study | *Nr. of participants:*  45 students:   - Gender not reported   Two arms: Intervention n=20 Control n=25  *Age range:*  11-12 years | **Intervention**:  *Program:* CPR training (see Moore, 1992)  **Control**:  No intervention. | Outcomes were measured before, immediately after and 5 months after training.  *Knowledge:* 22-item CPR questionnaire  *Skills*: CPR performance on manikin  [only post-training and follow-up data were extracted] |
| Reder, 2006, USA | Experimental: cluster randomized controlled trial | *Nr. of participants:* 779 students   - 424 males - 355 females   Four arms:  Intervention1 n=213   - 128 males - 85 females   Intervention2 n=170   - 79 males - 91 females   Intervention3 n=206   - 107 males - 99 females   Control n=190   - 110 males - 80 females   *Age range:*  14-18 years | **Intervention:**  *Program:* CPR and AED training  Intervention1:  *Content:* Interactive computer training.  *Duration:* 45 minutes  Intervention2:  *Content:* Interactive computer training + instructor-led hands-on manikin training.  *Duration:* 90 minutes  Intervention3:  *Content:* Traditional classroom instruction (video + demonstration) + instructor-led hands-on manikin training.  *Duration:* 90 minutes  **Control**:  No intervention. | Outcomes were measured 2 days after and 2 months after training.  *Knowledge:* 10-item CPR and AED questionnaire  *Skills*: CPR (ventilations + chest compressions) and AED performance on a manikin  [only skills data were extracted, since no separate knowledge scores for CPR and AED were available]  [only post-training data were extracted] |
| Sherif, 2005, Austria | Observational: cohort study | *Nr. of participants:*  57 students:   - 30 males - 27 females   *Age range:*  10 years n=27  14 years n=30 | **Intervention**:  *Program*: CPR training  *Content*: CPR lecture + demonstration + hands-on manikin training.  *Duration*: 30 minutes | Outcomes were measured immediately after training.  *Skills*: Check consciousness, call for help, start CPR. CPR quality (ventilations) on manikin |
| Uray,  2003, Austria | Observational:  before after study | *Nr. of participants:*  47 students:   - 20 males - 27 females     *Age range:*  6-7 years | **Intervention:**  *Program:* First aid training  *Content*: Emergency call, CPR, AED, recovery position, bleeding and burns.  Demonstration + hands-on manikin training.  *Duration:* 1 week | Outcomes were measured before and immediately after training.  *Knowledge:* Questionnaire in which students had to place three cartoon-like illustrations in the correct sequence |
| Wilks, 2016, Australia | Observational: before after study | *Nr. of participants*: 107 students:   - 51 males - 56 females   *Age range*:  11-12 years  Mean ± SD:  11.4 ± 0.5 years | **Intervention:**  *Program*: First aid, CPR and beach safety training  *Content*:   - Beach safety - Leadership and team bonding exercises - First aid: sprains, bleeding, choking, heart attack, allergic reactions - Hands-on manikin training   *Duration:* 1 day | Outcomes were measured before, 1 week after, and 8 weeks after training.  *Knowledge:* 50-item quiz on emergency services and life-supporting first aid |
| Wilks, 2017, Australia | Observational:  before after study | *Nr. of participants*: 107 students:   - 51 males - 56 females   *Age range*:  11-12 years  Mean ± SD:  11.4 ± 0.5 years | **Intervention:**  *Program*: First aid, CPR and beach safety training  *Content*:   - Beach safety - Leadership and team bonding exercises - First aid: sprains, bleeding, choking, heart attack, allergic reactions - Hands- on manikin training   *Duration:* 1 day | Outcomes were measured before, 1 week after, and 8 weeks after training.  *Knowledge:* Self-developed quiz on beach safety. (not relevant for ES)  *Attitude:* Confidence in providing first aid to family member/ friend/ same age stranger/ adult stranger  [Only attitude data were extracted] |
| Younas, 2006, UK | Experimental: non-randomized controlled trial | *Nr. of participants:*  59 students:   - Gender not reported   Two arms:  Intervention n=34  Control n=25  *Age range:*  13-16 years | **Intervention:**  *Program*: CPR/AED training  *Content*: CPR and AED use. Hands-on manikin training.  *Duration*: Not reported.  **Control:**  No intervention | Outcomes were measured 6 months after training.  *Skills*: 10-item checklist during cardiac arrest scenario |

**Synthesis of findings**

| **Four steps in first aid** | | | | | |
| --- | --- | --- | --- | --- | --- |
| **Outcome** | | **Comparison** | **Effect Size** | **#studies, # participants** | **Reference** |
| *Knowledge* | | | | | |
| ***Care for safety*** | | | | | |
| - 10-12 years | | | | | |
| Knowledge: Personal safety | Pre vs post | | Statistically significant:  4 (8.7%) vs 43 (93.5%) §  RR: 10.75* £  (p<0.001)  *in favour of first aid training* | 1, 46 vs 46  (within subject) | Connolly, 2007 |
| ***Call for help*** | | | | | |
| - 10-12 years | | | | | |
| Knowledge: seeking help | Pre vs post | | Statistically significant:  7 (15.2%) vs 36 (78.3%) §  RR:5.14 *£  (p<0.001)  *in favour of first aid training* | 1, 46 vs 46  (within subject) | Connolly, 2007 |
| - 11-12 years | | | | | |
| Knowledge: FA unconsciousness – send for help | Pre vs 1 week post | | Not statistically significant:  63 (59%) vs 64 (61%) §  RR: 1.07* £†  (p=0.382- overall p) | 1, 107 vs 102 (within subject) | Wilks, 2016 |
|  | Pre vs 8 weeks post | | Not statistically significant:  63 (59%) vs 54 (51%) §  RR: 0.87* £†  (p=0.382- overall p) | 1, 107 vs 105 (within subject) |  |
|  | 1 week post vs 8 weeks post | | Not statistically significant:  64 (61%) vs 54 (51%) §  RR: 0.82* £†  (p=0.382- overall p) | 1, 102 vs 105 (within subject) |  |
| ***Emergency call*** | | | | | |
| - 5-6 years | | | | | |
| Knowledge: emergency number (at least 2-point increase in test scores) | FA training vs no intervention | | Not statistically significant:  60 (67%) vs 57 (63%) §  RR: 1.06, 95%CI [0.86;1.32] ¥  (p=0.57) * | 1, 90 vs 91 | Luria, 2000 |
| Knowledge: How to dial emergency number (/16) |  |  | Not statistically significant:  Mean pre-post change, [range]: 4.15, [-13;-16] vs 3.66, [-8;-13]  MD: 0.49* £†  (p=0.41) |  |  |
| Knowledge: know when to call emergency number (3 situations) |  |  | Emergency situation1:  Statistically significant:  87 (62.1%) vs 12 (8.3%) §  RR: 7.51, 95%CI [4.30;13.11]*  (p<0.0001)  *In favour of first aid training*  Emergency situation 3:  Statistically significant:  93 (66.4%) vs 20 (13.8%) §  RR: 4.82, 95%CI [3.15;7.36]*  (p<0.0001)  *In favour of first aid training*  Emergency situation 2 (correct response = don’t call):  Not statistically significant:  105 (75%) vs 110 (75.9%)  RR: 0.99, 95%CI [0.87;1.13]*  (p=0.24) | 1, 140 vs 145 | Ammirati, 2014 |
| Knowledge: Describe the situation from photograph |  |  | Emergency situation 1:  Statistically significant:  95 (67.9%) vs 66 (45.5%) §  RR: 1.49, 95%CI [1.21;1.84]*  (p<0.001)  *In favour of first aid training*  Emergency situation 2:  Statistically significant:  100 (71.4%) vs 60 (41.4%) §  RR: 1.73, 95%CI [1.38;2.15]*  (p<0.0001)  *In favour of first aid training*  Emergency situation 3:  Statistically significant:  106 (75.7%) vs 87 (60.0%) §  RR: 1.26, 95%CI [1.07;1.48]*  (p=0.01)  *In favour of first aid training* |  |  |
| - 6-7 years | | | | | |
| Knowledge: Emergency number | FA training vs no intervention | | Statistically significant:  90 (77%) vs 18 (16%) §  RR: 4.74, 95%CI [3.07;7.32]*  (p<0.00001)  *In favour of first aid training* | 1, 117 vs 111  (immediately post) | Bollig, 2009 |
|  |  |  | Statistically significant:  58 (46%) vs 18 (16%) §  RR: 2.86, 95%CI [1.80;4.55]*  (p<0.00001)  *In favour of first aid training* | 1, 125 vs 111  (6 month follow-up) |  |
| - 10-12 years | | | | | |
| Knowledge: Emergency number | FA training vs no intervention | | Statistically significant:  3.0±1.0 vs 2.8±1.1  MD: 0.2, 95%CI [0.08;0.32]  (p=0.002)*  *in favour of first aid training* | 1, 542 vs 554 § (power analysis) | Frederick, 2000 |
|  | Pre vs immediately post | | Not statistically significant:  45 (97.8%) vs 46 (100%) §  RR: 1.02* £†  (p>0.05) | 1, 46 vs 46  (within subject) | Connolly, 2007 |
| - 11-12 years | | | | | |
| Knowledge: Emergency number | Pre vs 1 week post | | Not statistically significant:  0.88 ± 0.33 vs 0.95 ± 0.21  MD: 0.07* £†  (p=0.142- overall p) | 1, 107 vs 102 § (within subject) | Wilks, 2016 |
|  | Pre vs 8 weeks post | | Not statistically significant:  0.88 ± 0.33 vs 0.92 ± 0.27  MD: 0.04* £†  (p=0.142- overall p) | 1, 107 vs 105 § (within subject) |  |
|  | 1 week post vs 8 weeks post | | Not statistically significant:  0.95 ± 0.21 vs 0.92 ± 0.27  MD: -0.03* £†  (p=0.142 – overall p) | 1, 102 vs 105 § (within subject) |  |
| Knowledge: call emergency number + sit patient in comfortable position | Pre vs 1 week post | | Not statistically significant:  82 (77%) vs 91 (87%) §  RR: 1.16* £†  (p=0.718- overall p) | 1, 107 vs 102 (within subject) |  |
|  | Pre vs 8 weeks post | | Not statistically significant:  82 (77%) vs 85 (81%) §  RR: 1.06* £†  (p=0.718- overall p) | 1, 107 vs 105 (within subject) |  |
|  | 1 week post vs 8 weeks post | | Not statistically significant:  91 (87%) vs 85 (81%) §  RR: 0.91* £†  (p=0.718- overall p) | 1, 102 vs 105 (within subject) |  |
| - 15-18 years | | | | | |
| Knowledge: Emergency call | Pre vs post | | Not statistically significant:  168 (98.8%) vs 170 (100%) §  OR: ∞, 95%CI [0.19;∞] ¥  (p=0.5)* | 1, 170 vs 170  (within subject) | Celik, 2013 |
| - 16-18 years | | | | | |
| Knowledge: Emergency number | Pre vs post | | Not statistically significant:  98 (98%) vs 100 (100%) §  OR: ∞, 95%CI [0.19;∞] ¥  (p=0.5)* | 1, 100 vs 100  (within subject) | Akca, 2016 |
| ***Check consciousness*** | | | | | |
| - 10-12 years | | | | | |
| Knowledge: Assess conscious level | Pre vs post | | Not statistically significant:  34 (73.9%) vs 39 (84.8%) §  RR: 1.15* £†  (p>0.05) | 1, 46 vs 46  (within subject) | Connolly, 2007 |
| Knowledge: Assess consciousness |  |  | Statistically significant:  24 (52.2%) vs 39 (84.8%) §  RR: 1.63* £  (p<0.001)  *in favour of first aid training* |  |  |
| ***Open the airway*** | | | | | |
| - 10-12 years | | | | | |
| Knowledge: opening airway | Pre vs post | | Statistically significant:  13 (28.3%) vs 37 (80.4%) §  RR:2.85 * £  (p<0.001) | 1, 46 vs 46  (within subject) | Connolly, 2007 |
| Knowledge: Assess breathing |  |  | Question 1:  Not statistically significant:  38 (82.6%) vs 44 (95.7%) §  RR: 1.16* £†  (p>0.05)  Question 2:  Statistically significant:  20 (43.5%) vs 40 (87.0%) §  RR: 2* £  (p<0.001)  *in favour of first aid training*  Question 3:  Statistically significant:  21 (45.7%) vs 42 (91.3%) §  RR: 2* £  (p<0.001)  *in favour of first aid training* |  |  |
| ***Recovery position*** | | | | | |
| - 6-7 years | | | | | |
| Knowledge: Recovery position | Pre vs post | | Statistically significant:  MD: 18%, 95%CI [6%;28%] λ  (p<0.05)  In favour of first aid training | 1, 47 vs 47 §  (within subject) | Uray, 2003 |
| - 10-12 years | | | | | |
| Knowledge: Recovery position | Pre vs immediately post | | Statistically significant:  26 (56.5%) vs 41 (89.1%) §  RR: 1.58* £  (p<0.001)  *in favour of first aid training* | 1, 46 vs 46  (within subject) | Connolly, 2007 |
| *Skills* | | | | | |
| ***Care for safety*** | | | | | |
| - 10-11 years | | | | | |
| Skills: danger assessment | FA training vs no intervention | | Statistically significant:  191 (36%) vs 16 (3%) §  RR: 12.99, 95%CI [7.91;21.34]  (p<0.00001)*  *in favour of first aid training* | 1, 534 vs 581 §(power analysis) | Frederick, 2000  Frederick, 2002 |
| - 11-16 years | | | | | |
| Skills: Checking environment  (2 situations) | FA training vs no intervention | | Scenario 1:  Not statistically significant:  46 (31%) vs 56 (25%) §  RR: 1.23, 95%CI [0.89;1.72] ¥  (p=0.21)*  Scenario 2:  Not statistically significant:  14 (10%) vs 17 (8%) §  RR: 1.24, 95%CI [0.63;2.43] ¥  (p=0.54)* | 1, 147 vs 221 | Campbell, 2001 |
| - 13-16 years | | | | | |
| Skills: Check for danger | FA training vs no intervention | | Statistically significant:  17 (50%) vs 6 (24%) §  RR: 2.08, 95%CI [0.96;4.52] ¥  (p=0.06)*  *in favour of first aid training* | 1, 34 vs 25 | Younas, 2006 |
| ***Call for help*** | | | | | |
| - 10-11 years | | | | | |
| Skills: Call for help | FA training vs no intervention | | Statistically significant:  140 (26%) vs 54 (9%) §  RR: 2.82, 95%CI [2.11;3.77]  (p<0.00001)*  *in favour of first aid training* | 1, 534 vs 581 §(power analysis) | Frederick, 2000  Frederick, 2002 |
| - 11-12 years | | | | | |
| Skills: Call for help | FA training vs no intervention | | 0 (0%) vs 0 (0%) §  RR: not estimable  statistical analysis not possible | 1, 20 vs 25 (post) | Plotnikoff, 1989 |
|  |  |  | 0 (0%) vs 0 (0%) §  RR: not estimable  statistical analysis not possible | 1, 20 vs 25 (5 month follow-up) |  |
| - 13-16 years | | | | | |
| Skills: Call for help | FA training vs no intervention | | Not statistically significant:  12 (79%) vs 4 (16%) §  RR: 2.21, 95%CI [0.81;6.04] ¥  (p=0.12)* | 1, 34 vs 25 | Younas, 2006 |
| - 14-15 years | | | | | |
| Skills: Call for help | Pre vs post | | Statistically significant:  22 (17%) vs 3 (2%) §  RR: 0.14* £  (p<0.001)  *in favour of first aid training* | 1, 129 vs 129 (within subject) | Meissner, 2012 |
|  | Post vs 4 month follow-up | | Statistically significant:  3 (2%) vs 11 (8.2%) §  RR: 3.67* £  (p=0.016)  *decline over time* |  |  |
| ***Emergency call*** | | | | | |
| - 5-6 years | | | | | |
| Skills: Use the telephone | FA training vs no intervention | | Statistically significant:  78 (55.7%) vs 12 (17.7%) §  RR: 3.16, 95%CI [1.85;5.39]*  (p<0.0001)  *In favour of first aid training* | 1, 140 vs 68 | Ammirati, 2014 |
| Skills: Introduce yourself, explain location |  |  | Statistically significant:  115 (82.1%) vs 23 (33.8%) §  RR: 2.43, 95%CI [1.73;3.42]*  (p<0.0001)  *In favour of first aid training* |  |  |
| Skills: Describe the situation during simulation exercise |  |  | Statistically significant:  125 (89.3%) vs 51 (75%) §  RR: 1.19, 95%CI [1.03;1.38]*  (p=0.01)  *In favour of first aid training* |  |  |
| - 6-7 years | | | | | |
| Skills: Provide correct information | FA training vs no intervention | | Statistically significant:  59 (50%) vs 7 (6%) §  RR: 8.00, 95%CI [3.82;16.75]*  (p<0.00001)  *in favour of first aid training* | 1, 117 vs 111  (immediately post) | Bollig, 2009 |
|  |  |  | Statistically significant:  86 (69%) vs 7 (6%) §  RR: 10.91, 95%CI [5.27;22.56]*  (p<0.00001)  *in favour of first aid training* | 1, 125 vs 111  (6 month follow-up) |  |
| - 10-12 years | | | | | |
| Skills: Call emergency number | *FA training vs no intervention* | | *Statistically significant:*  *36 (69%) vs 19 (38%) §*  *RR: 1.82, 95%CI [1.22;2.71]**  *(p=0.004)*  *in favour of first aid training* | 1, 52 vs 50 | Lorem, 2008 |
| - 11-16 years | | | | | |
| Skills: Call emergency number  (2 situations) | FA training vs sham intervention | | Scenario 1 (severe glass wound)  Statistically significant:  95 (65%) vs 119 (54%) §  RR: 1.20, 95%CI [1.01;1.42]*  (p=0.04)  *in favour of first aid training*  Scenario 2 (severe burn injury)  Not statistically significant:  50 (34%) vs 63 (29%) §  RR: 1.19, 95%CI [0.88;1.62]*¥  (p=0.244) | 1, 147 vs 221 | Campbell, 2001 |
| - 13-16 years | | | | | |
| Skills: Call emergency number + provide information | FA training vs no intervention | | Not statistically significant:  28 (82.4%) vs 22 (88%) §  RR: 094, 95%CI [0.76;1.16]  (p=0.54)* | 1, 34 vs 25 | Younas, 2006 |
| - 14-15 years | | | | | |
| Skills: Call emergency number | Pre vs post | | Statistically significant:  86 (67%) vs 95 (92%) §  RR: 1.37* £  (p<0.001)  *in favour of first aid training* | 1, 129 vs 103 (within subject) | Meissner, 2012 |
|  | Post vs 4 month follow-up | | Not statistically significant:  95 (92%) vs 94 (95.9%) §  RR: 1.04* £†  (p=0.75) | 1, 103 vs 98  (within subject) |  |
| - 14-18 years | | | | | |
| Skills: Call emergency number | Brief video vs no intervention | | Statistically significant:  49 (84.5%) vs 17 (37.0%) §  RR: 2.34, 95%CI [1.57;3.47]  (p<0.00001, still significant after Bonferroni correction p=0.0056)*  *in favour of first aid training* | 1, 58 vs 47 (post) | Beskind, 2016 |
|  | Classroom training vs no intervention | | Statistically significant:  40 (75.0%) vs 17 (37.0%) §  RR: 2.05, 95%CI [1.36;3.09]  (p<0.00001, still significant after Bonferroni correction p=0.0056)*  *in favour of first aid training* | 1, 54 vs 47 (post) |  |
|  | Brief video vs no intervention | | Not statistically significant:  45 (78.3%) vs 26 (56.3%) §  RR: 1.40, 95%CI [1.05;1.88]  (p=0.02, but no longer significant after Bonferroni correction p=0.0056)* | 1, 58 vs 47 (2 month follow-up) |  |
|  | Classroom training vs no intervention | | Not statistically significant:  40 (74.5%) vs 26 (56.3%) §  RR: 1.34, 95%CI [0.99,1.81]  (p=0.06)* | 1, 54 vs 47 (2 month follow-up) |  |
| Skills: Time to calling 911 (sec.) | Brief video vs no intervention | | Not statistically significant:  8.5±7.2 vs 9.5±10.99  MD: -1.0, 95%CI [-4.65;2.65]  (p=0.59)* | 1, 58 vs 47 (post) § |  |
|  | Classroom training vs no intervention | | Not statistically significant:  10.9±19.4 vs 9.5±10.99  MD: 1.40, 95%CI [-4.65;7.45] ¥  (p=0.65)* | 1, 54 vs 47 (post) § |  |
|  | Brief video vs no intervention | | Not statistically significant:  7.3±6.5 vs 8.4±9.1  MD -1.10, 95%CI [-4.19;1.99] ¥  (p=0.49) | 1, 58 vs 47 (2 month follow-up) § |  |
|  | Classroom training vs no intervention | | Statistically significant:  5.5±5.5 vs 8.4±9.1  MD: -2.90, 95%CI [-5.89,0.09] ¥  (p=0.06, but significant after Bonferroni correction)  *in favour of first aid training* | 1, 54 vs 47 (2 month follow-up) § |  |
| ***Check consciousness*** | | | | | |
| - 6-7 years | | | | | |
| Skills: Assess consciousness | FA training vs no intervention | | Statistically significant:  57 (49%) vs 8 (7%) §  RR: 6.76, 95%CI [3.38;13.52]  (p<0.00001)*  *in favour of first aid training* | 1, 117 vs 111  (post) | Bollig, 2009 |
|  |  |  | Not statistically significant:  15 (12%) vs 8 (7%) §  RR: 1.67, 95%CI [0.73;3.78] ¥  (p=0.22)* | 1, 125 vs 111  (6 month follow-up) |  |
| - 10-11 years | | | | | |
| Skills: Check responsiveness | FA training vs no intervention | | Statistically significant:  312 (58%) vs 68 (12%) §  RR: 4.99, 95%CI [3.95;6.31]*  (p<0.0005)  *in favour of first aid training* | 1, 534 vs 581 §(power analysis) | Frederick, 2000  Frederick, 2002 |
| - 10-14 years | | | | | |
| Skills: Time to check oral cavity (seconds) | 10 year vs 14 year | | Not statistically significant:  8.3±3.3 vs 8.9±2.6  MD: -0.60, 95%CI [-2.15;0.95]  (p=0.45)* | 1, 27 vs 30 § | Sherif, 2005 |
| - 11-12 years | | | | | |
| Skills: establish unresponsiveness | FA training vs no intervention | | Statistically significant:  8 (40%) vs 1 (4%) §  RR: 10.0, 95%CI [1.36;73.45]  (p=0.02)*  *in favour of first aid training* | 1, 20 vs 25 (post) | Plotnikoff, 1989 |
|  |  |  | Statistically significant:  4 (20%) vs 0 (0%) §  RR: 11.14, 95%CI [0.64;195.47]  (p=0.10)*  *in favour of first aid training* | 1, 20 vs 25 (5 month follow-up) |  |
| Skills: Check consciousness – physical |  |  | Not statistically significant:  8 (15.7%) vs 13 (25.5%) §  RR: 0.62, 95%CI [0.28;1.36] ¥  (p=0.23)* | 1, 51 vs 51 | Moore, 1992 |
| Skills: Check consciousness - verbal |  |  | Not statistically significant:  5 (9.8%) vs 5 (9.8%) §  RR: 1.00, 95%CI [0.31;3.25] ¥  (p=1.00)* |  |  |
| - 12 years | | | | | |
| Skills: Check responsiveness (shaking) | FA training vs no intervention | | Statistically significant:  32 (61%) vs 6 (12%) §  RR: 5.13, 95%CI [2.35;11.20]*  (p<0.05)  *in favour of first aid training* | 1, 52 vs 50 | Lorem, 2008 |
| - 13-16 years | | | | | |
| Skills: Check responsiveness | FA training vs no intervention | | Statistically significant:  21 (61.8%) vs 6 (24%) §  RR: 2.57, 95%CI [1.22;5.43]  (p=0.01)*  *in favour of first aid training* | 1, 34 vs 25 | Younas, 2006 |
| - 14-15 years | | | | | |
| Skills: Check responsiveness (verbal) | Pre vs post | | Statistically significant:  31 (24%) vs 95 (92%) §  RR: 3.84* £  (p<0.001)  *in favour of first aid training* | 1, 129 vs 103  (within subject) | Meissner, 2012 |
|  | Post vs 4 month follow-up | | Statistically significant:  95 (92%) vs 80 (81.6%) §  RR: 0.89* £  (p=0.06)  *decline over time* | 1, 103 vs 98  (within subject) |  |
| Skills: Check responsiveness (shaking) | Pre vs post | | Statistically significant:  19 (15%) vs 88 (85%) §  RR: 5.80* £  (p<0.001)  *in favour of first aid training* | 1, 129 vs 103  (within subject) |  |
|  | Post vs 4 month follow-up | | Not statistically significant:  88 (85%) vs 84 (85.7%) §  RR: 1.00* £†  (p=0.57) | 1, 103 vs 98  (within subject) |  |
| ***Open the airway*** | | | | | |
| - 6-7 years | | | | | |
| Skills: Assess breathing | FA training vs no intervention | | Statistically significant:  92 (79%) vs 3 (3%) §  RR: 29.09, 95%CI [9.49;89.18]  (p<0.00001)*  *in favour of first aid training* | 1, 117 vs 111  (post) | Bollig, 2009 |
|  |  |  | Statistically significant:  46 (37%) vs 3 (3%) §  RR: 13.62, 95%CI [4.36;42.56]  (p<0.00001)*  *in favour of first aid training* | 1, 125 vs 111  (6 month follow-up) |  |
| Skills: Airway management |  |  | Statistically significant:  80 (68%) vs 1 (1%) §  RR: 75.90, 95%CI [10.74;536.13]  (p<0.0001)*  *in favour of first aid training* | 1, 117 vs 111  (post) |  |
|  |  |  | Statistically significant:  30 (24%) vs 1 (1%) §  RR: 26.64, 95%CI [3.69;192.16]  (p=0.001)*  *in favour of first aid training* | 1, 125 vs 111  (6 month follow-up) |  |
| - 10-11 years | | | | | |
| Skills: Assess airway | FA training vs no intervention | | Statistically significant:  90 (17%) vs 20 (3%) §  RR: 4.90, 95%CI [3.06;7.83]  (p<0.00001)*  *in favour of first aid training* | 1, 534 vs 581 §(power analysis) | Frederick, 2000  Frederick, 2002 |
| Skills: Assess breathing |  |  | Statistically significant:  80 (15%) vs 9 (2%) §  RR: 9.67, 95%CI [4.90;19.07]  (p<0.00001)*  *in favour of first aid training* |  |  |
| - 10-14 years | | | | | |
| Skills: Time to assess breathing (seconds) | 10 year vs 14 year | | Not statistically significant:  21.7±5.3 vs 23.6±5.2  MD: -1.90, 95%CI [-4.63;0.83]  (p=0.17)* | 1, 27 vs 30 § | Sherif, 2005 |
| - 11-12 years | | | | | |
| Skills: Assess breathing (chest movement) | FA training vs no intervention | | Not statistically significant:  26 (51%) vs 18 (35.3%) §  RR: 1.44, 95%CI [0.91;2.29] ¥  (p=0.11)* | 1, 51 vs 51 | Moore, 1992 |
| Skills: Assess breathing (listening) |  |  | Not statistically significant:  12 (23.5%) vs 13 (25.5%) §  RR: 0.92, 95%CI [0.47;1.83] ¥  (p=0.82)* |  |  |
| Skills: Open airway (head tilt) |  |  | Not statistically significant:  36 (70.6%) vs 27 (52.9%) §  RR: 1.33, 95%CI [0.97;1.82] ¥  (p=0.07)* |  |  |
| Skills: Assess breathing |  |  | Statistically significant:  13 (65%) vs 2 (8%) §  RR: 8.13, 95%CI [2.07;31.90]  (p=0.003)*  *in favour of first aid training* | 1, 20 vs 25 (post) | Plotnikoff, 1989 |
|  |  |  | Not statistically significant:  6 (30%) vs 1 (4%) §  RR: 7.50, 95%CI [0.98;57.32] ¥  (p=0.05)  *in favour of first aid training* | 1, 20 vs 25 (5 month follow-up) |  |
| Skills: Open airway |  |  | Statistically significant:  18 (90%) vs 7 (28%) §  RR: 3.21, 95%CI [1.69;6.13]  (p=0.0004)*  *in favour of first aid training* | 1, 20 vs 25 (post) |  |
|  |  |  | Statistically significant:  16 (80%) vs 2 (8%) §  RR: 10.0, 95%CI [2.60;38.47]  (p=0.0008)  *in favour of first aid training* | 1, 20 vs 25 (5 month follow-up) |  |
| - 13-16 years | | | | | |
| Skills: Assess breathing | FA training vs no intervention | | Not statistically significant:  20 (58.8%) vs 9 (36%) §  RR: 1.63, 95%CI [0.90;2.96] ¥  (p=0.10)*  *in favour of first aid training* | 1, 34 vs 25 | Younas, 2006 |
| Skills: Open airway |  |  | Statistically significant:  21 (61.8%) vs 7 (16%) §  RR: 2.21; 95%CI [1.12;4.36]  (p=0.02)*  *in favour of first aid training* |  |  |
| - 14-15 years | | | | | |
| Skills: Check breathing | Pre vs post | | Statistically significant:  35 (27%) vs 95 (92%) §  RR: 3.40* £  (p<0.001)  *in favour of fist aid training* | 1, 129 vs 103  (within subject) | Meissner, 2012 |
|  | Post vs 4 month follow-up | | Not statistically significant:  95 (92%) vs 91 (92.9%) §  RR: 1.01* £  (p=1.0) | 1, 103 vs 98  (within subject) |  |
| - 14-18 years | | | | | |
| Skills: Open airway | Computer vs no intervention | | Statistically significant:  104 (50%) vs 23 (12%) §  RR: 4.09, 95%CI [2.72;6.14]  (p<0.00001)*  *in favour of first aid training* | 1, 208 vs 188  (post) | Reder, 2006 |
|  | Computer + demo vs no intervention | | Statistically significant:  117 (73%) vs 23 (12 %) §  RR: 5.98, 95%CI [4.03;8.87]  (p<0.00001)*  *in favour of first aid training* | 1, 160 vs 188  (post) |  |
|  | Video + demo vs no intervention | | Statistically significant:  120 (62%) vs 23 (12%) §  RR: 5.08, 95%CI [3.41;7.57]  (p<0.00001)*  *in favour of first aid training* | 1, 193 vs 188  (post) |  |
|  | Computer vs no intervention | | Statistically significant:  75 (41%) vs 22 (15%) §  RR: 2.79, 95%CI [1.83;4.26]  (p<0.00001)*  *in favour of first aid training* | 1, 182 vs 149 (2 month follow-up) |  |
|  | Computer + demo vs no intervention | | Statistically significant:  80 (52%) vs 22 (15%) §  RR: 3.54, 95%CI [2.34;5.36]  (p<0.00001)*  *in favour of first aid training* | 1, 153 vs 149  (2 month follow-up) |  |
|  | Video + demo vs no intervention | | Statistically significant:  84 (53%) vs 22 (15%) §  RR: 3.60, 95%CI [2.38;5.44]  (p<0.00001)  *in favour of first aid training* | 1, 158 vs 149  (2 month follow-up) |  |
| ***Recovery position*** | | | | | |
| - 6-7 years | | | | | |
| Skills: Recovery position | FA training vs no intervention | | Statistically significant:  102 (87%) vs 1 (1%) §  RR: 96.77, 95%CI [13.74;681.76]  (p<0.00001)*  *in favour of first aid training* | 1, 117 vs 111  (immediately post) | Bollig, 2009 |
|  |  |  | Statistically significant:  70 (56%) vs 1 (1%) §  RR: 62.16, 95%CI [8.78;440.10]  (p<0.00001)*  *in favour of first aid training* | 1, 125 vs 111  (6 month follow-up) |  |
| - 10-11 years | | | | | |
| Skills: Recovery position | FA training vs no intervention | | Statistically significant:  40 (8%) vs 10 (2%) §  RR: 4.35, 95%CI [2.20;8.62]*  (p<0.0005)  *in favour of first aid training* | 1, 534 vs 581 §(power analysis) | Frederick, 2002 |
| Skills: Successful recovery position |  |  | Statistically significant:  62 (31.3%) vs 18 (19.1%) §  RR: 1.64, 95%CI [1.03;2.60]  (p=0.04)*  *in favour of first aid training* | 1, 198 vs 94 | Joshi, 2012 |
| Skills: Turned on side in final recovery position |  |  | Statistically significant:  144 (73%) vs 45 (48%) §  RR: 1.52, 95%CI [1.21, 1.91]  (p=0.0003)*  *in favour of first aid training* |  |  |
| Skills: Airway secured in final recovery position |  |  | Statistically significant:  92 (46%) vs 30 (32%) §  RR: 1.46, 95%CI [1.05;2.03]  (p=0.03)*  *in favour of first aid training* |  |  |
| Skills: Stable in final recovery position |  |  | Statistically significant:  77 (39%) vs 21 (22%) §  RR: 1.74, 95%CI [1.15;2.64]  (p=0.009)*  *in favour of first aid training* |  |  |
| Skills: Move arm away from body (move used during routine) |  |  | Not statistically significant:  72 (36%) vs 22 (23%) §  RR: 1.55, 95%CI [1.03;2.34]* £†  (p=0.026, no longer significant after Bonferroni correction p<0.007) |  |  |
| Skills: Raise leg to flexed position (move used during routine) |  |  | Statistically significant:  143 (72%) vs 30 (32%) §  RR: 2.26, 95%CI [1.66;3.08]* £  (p<0.001, still significant after Bonferroni correction p<0.007)  *in favour of first aid training* |  |  |
| Skills: Move hand to other arm/cheek (move used during routine) |  |  | Statistically significant:  91 (46%) vs 13 (14%) §  RR: 3.32, 95%CI [1.96; 5.63]* £  (p<0.001, still significant after Bonferroni correction p<0.007)  *in favour of first aid training* |  |  |
| Skills: Hold hand in position while rolling (move used during routine) |  |  | Not statistically significant:  24 (12%) vs 8 (9%) §  RR: 1.42, 95%CI [0.67;3.05]* ¥ £†  (p=0.356) |  |  |
| Skills: Use knee as pivot (move used during routine) |  |  | Statistically significant:  45 (23%) vs 9 (10%) §  RR: 2.37, 95%CI [1.21;4.65]* £  (p=0.007, still significant after Bonferroni correction p<0.007)  *in favour of first aid training* |  |  |
| Skills: Roll on side (move used during routine) |  |  | Statistically significant:  169 (85%) vs 58 (62%) §  RR: 1.38, 95%CI [1.17;1.64]* £  (p<0.001, still significant after Bonferroni correction p<0.007)  *in favour of first aid training* |  |  |
| Skills: Attend airway (move used during routine) |  |  | Not statistically significant:  43 (22%) vs 11 (12%) §  RR: 1.86, 95%CI [1.00;3.43]* £†  (p=0.039, no longer significant after Bonferroni correction p<0.007) |  |  |
| - 11-12 years | | | | | |
| Skills: Recovery position | FA training vs no intervention | | Statistically significant:  12 (60%) vs 2 (8%) §  RR: 7.50, 95%CI [1.89;29.71]  (p=0.004)*  *in favour of first aid training* | 1, 20 vs 25 (post) | Plotnikoff, 1989 |
|  |  |  | Statistically significant:  10 (50%) vs 0 (0%) §  RR: 26.00, 95%CI [1.62;418.32]  (p=0.02)*  *in favour of first aid training* | 1, 20 vs 25 (5 month follow-up) |  |
| *Attitude* | | | | | |
| - 10-13 years | | | | | |
| Attitude: Self-confidence | | 4 year follow-up  (no pre or control) | Feel capable to intervene in an emergency: 83.1% £†  Too afraid to intervene: 25.3% £† | 1, 144 vs 144§  (within subject) | Bohn, 2012 |
| - 11-12 years | | | | | |
| Attitude: self-efficacy using acquired skills | | Post training | boys: 13 (77%)  girls: 9 (38%) | 1, 41 total: 17 boys and 24 girls § £†  (case series) | Lester, 1996 |
| Attitude: Self-efficacy | | Boys vs girls | Statistically significant:  66 (55%) vs 79 (75%) §  RR: 0.75, 95%CI [0.62;0.92]*  (p<0.01)  *In favour of girls* | 1, 120 vs 108  (case series) | Lester, 1997 |
| Attitude: FA confidence | | Pre vs 1 week post vs 8 week follow-up | Percentage of respondents who are ‘confident’ or ‘very confident’   - On a family member:   71 vs 78 vs 87 § £†   - On a friend:   47 vs 58 vs 60 § £†   - On a same age stranger:   14 vs 21 vs 20 § £†   - On an adult stranger:   14 vs 20 vs 17 § £† | 1, 107 vs 102 vs 105 | Wilks, 2017 |
| - 11-16 years | | | | | |
| Attitude: FA confidence | | FA training vs no intervention | Statistically significant:  Regression: -0.070, 95%CI [--0.130;-0.010] £  (p≤0.05)  *in favour of first aid training* | 1, 147 vs 221 § | Campbell, 2001 |
|  |  |  | immediately after:  3.15 vs 3.05  MD: 0.1* £ |  |  |
|  |  |  | 1 year follow-up:  3.23 vs 3.21  MD: 0.02* £ |  |  |
| - 12-15 years | | | | | |
| Attitude: Self-confidence would increase with training | | Cross-sectional | Strongly agree: 74.1%  Agree: 12.1%  Neither agree nor disagree: 7.4%  Disagree: 2.7%  Strongly disagree: 3.7% | 1, 301 § £† | Petric, 2013 |
| - 13-14 years | | | | | |
| Attitude: Feelings towards having to provide FA if necessary  (change after vs before intervention) | FA training vs no intervention | | Girls  Statistically significant:  Mean pre-post change: 2.8 vs -4.3  MD: 7.1, 95%CI [0.1;14.1]  (p<0.05)  *in favour of first aid training*  Boys  Statistically significant:  Mean pre-post change: 2.0 vs -7.0  MD: 9.00, 95%CI [1.7;16.3]  (p<0.05)  *in favour of first aid training* | 1, 741 vs 152 | Engeland, 2002 |
| Attitude: Learning FA  (change after vs before intervention) |  |  | Girls  Not statistically significant:  Mean pre-post change: -0.4 vs -1.9  MD: 1.5, 95%CI [-2.6;5.5] ¥  (p>0.05)  Boys  Statistically significant:  Mean pre-post change: 0.2 vs -5.1  MD: 5.3, 95%CI [0.1;10.3]  (p<0.05)  *in favour of first aid training* |  |  |
| Attitude: Self-efficacy |  |  | Girls  Statistically significant:  Mean pre-post change: 10.8 vs -4.5  MD: 15.3, 95%CI [8.7;21.6]  (p<0.05)  *in favour of first aid training*  Boys  Statistically significant:  Mean pre-post change: 6.7 vs -5.2  MD: 11.9, 95%CI [5.8;18.1]  (p<0.05)  *in favour of first aid training* |  |  |
| - 15-16 years | | | | | |
| Attitude: self-efficacy | Pre vs post | | Statistically significant:  Median (IQR): 6.0 (6.4-5.5) vs 6.8 (7.0-6.3) λ  (p<0.05)  *in favour of first aid training* | 1, 12 vs 12 (Swedish group) + 24 vs 24 (US group) §£†  (within subject) | Creutzfeldt, 2013 |
| ***Recovery position*** | | | | | |
| - 14-15 years | | | | | |
| Attitude: Sufficient knowledge about … | Post intervention | | Stable recovery position: 93.8% | 1, 103 § £† | Meissner, 2012 |
| Attitude: would have dared to apply … before training vs would dare to apply … following training |  |  | Stable recovery position:  123 (95%) vs 47 (46%) §  RR: 0.48* £† | 1, 129 vs 103  (within subject) |  |

Mean ± SD (unless otherwise indicated), MD: mean difference, RR: risk ratio, OR: odds ratio, RD: risk difference

* calculations done by the reviewer using Revman, R software, or Excel

£ No raw data available and CI cannot be calculated.

¥ Imprecision (large variability of results)

† Imprecision (lack of data)

§ Imprecision (limited sample size or low number of events)

λ data extracted from graph

Quality of evidence

***Experimental studies***

| **Author, Year** | **Lack of allocation concealment** | **Lack of blinding** | **Incomplete accounting of outcome events** | **Selective outcome reporting** | **Other limitations** |
| --- | --- | --- | --- | --- | --- |
| Ammirati, 2014 | Randomization: yes, no randomization  Allocation concealment: not applicable | Unclear | No | No | No pre-test/baseline assessment |
| Beskind, 2016 | Randomization:  no, a cluster-randomization was performed  Allocation concealment: no, allocation was concealed | No, statistician was blinded, outcomes were recorded on manikin or simple yes/no action checks | Yes, larger loss to follow-up in brief video compared to others | No | / |
| Bohn, 2012 | Randomization: yes, no randomization  Allocation concealment: not applicable | No, outcomes were recorded on manikin or knowledge questionnaire | Yes, unequal losses to follow-up. No ITT performed | No | Within subject design as no relevant data from control group could be extracted |
| Bollig, 2009 | Randomization and allocation concealment: unclear, not specified in the article | Unclear if those recording and analyzing outcomes were aware of the arm to which participants were allocated | No | No | No pre-test/baseline assessment |
| Campbell, 2001 | Randomization and allocation concealment: unclear, not specified in the article | No | Yes, considerable loss to follow-up | Yes, data from some time-points not reported | No pre-test/baseline assessment |
| Connolly, 2007 | Randomization: yes, no randomization  Allocation concealment: not applicable | No | No (loss to follow-up similarly in both groups) | No | Within subject design (no control available for extracted data); possible incorrect statistical analysis (data analyzed using Student’s t-test, incorrect for within subject design) |
| Creutzfeldt, 2013 | Randomization: yes, no randomization  Allocation concealment: not applicable | No | No | Yes, issue with n (%) from the feelings/perceptions scores | No passive control group  Pre and post self-confidence data were measured through one post-intervention exit questionnaire (not independent) |
| Engeland, 2002 | Randomization and allocation concealment: unclear, not specified in the article | No (no information in paper about blinding of those analyzing outcomes, but only questionnaires were used, which is an objective outcome measurement) | No, intention-to-treat analysis was performed | No | Pre-post change scores were used in the statistical analysis, but control and intervention group were not well matched (very different pre-training scores and similar post-training scores) |
| Frederick, 2000 | Randomization: yes, no randomization  Allocation concealment: not applicable | Yes, assessors were un-blinded | No | No | / |
| Frederick, 2002 | Randomization: yes, no randomization  Allocation concealment: not applicable | Yes, assessors were un-blinded | No | No | No pre-test/baseline assessment |
| Joshi, 2012 | Randomization: yes, no randomization  Allocation concealment: not applicable | No, independently judged by 2 observers | No | No | No pre-test/baseline assessment |
| Luria, 2000 | Randomization and allocation concealment: unclear, not specified in the article | No (no information about blinding of those analyzing outcomes, but only questionnaires were used, which is an objective outcome measurement) | Yes, considerable loss to follow-up, although similarly in both groups | No |  |
| Moore, 1992 | Randomization: yes, no randomization  Allocation concealment: not applicable | Unclear if those recording and analyzing outcomes were aware of the arm to which participants were allocated | Unclear, not specified in the article | No | No pre-test/baseline assessment |
| Plotnikoff, 1989 | Randomization: yes, no randomization  Allocation concealment: not applicable | Unclear if those recording and analyzing outcomes were aware of the arm to which participants were allocated | No | No | / |
| Reder, 2006 | Randomization: no, cluster randomization performed  Allocation concealment: unclear | No | No (loss to follow-up similarly in all groups) | No | No pre-test/baseline assessment |
| Younas, 2006 | Randomization: yes, no randomization  Allocation concealment: not applicable | Unclear if those recording and analyzing outcomes were aware of the arm to which participants were allocated | No | No | No pre-test/baseline assessment |

***Observational studies***

| **Author, Year** | **Inappropriate eligibility criteria** | **Inappropriate methods for exposure and outcome variables** | **Not controlled for confounding** | **Incomplete or inadequate follow-up** | **Other limitations** |
| --- | --- | --- | --- | --- | --- |
| Akca, 2016 | Yes, no inclusion of control population | No | Yes | No | / |
| Celik, 2013 | Yes, no inclusion of control population | No, a questionnaire was used | Yes, not controlled for confounding factors (i.e. previous history with choking infants/children) | No | / |
| Lester, 1996 | Not applicable | No (not applicable for exposure variables; no flawed measurement of outcome variables) | Yes | No | Case series |
| Lester, 1997 | Yes, no inclusion of passive control population | No (not applicable for exposure variables; no flawed measurement of outcome variables) | Yes | No | No pre-test/baseline assessment |
| Lorem, 2008 | No | No (not applicable for exposure variables; no flawed measurement of outcome variables) | Yes | No | No pre-test/baseline assessment |
| Meissner, 2012 | Yes, no inclusion of passive control population | No, a questionnaire and checklist were used | No, controlled for age, height, weight, BMI and gender | Yes, considerable drop-out | Yes, questionable number of participants: 72 vs 59 (girls vs. boys) is more than total of 103 tested post intervention |
| Petric, 2013 | Not applicable | No, a questionnaire was used | Yes, not controlled for confounding factors such as experience with cardiac arrest or social and cultural differences | No | Cohort study, sampling was not randomized |
| Sherif, 2005 | Not applicable | No (not applicable for exposure variables; no flawed measurement of outcome variables) | Yes (only sub-analyses for gender, no controlling for age,…) | No | Cohort study |
| Uray, 2003 | Yes, no inclusion of control population | No (not applicable for exposure variables; no flawed measurement of outcome variables) | Yes | No | Incorrect statistical analysis likely (2x2 table next to graph from which we extracted MD values adds up to 92 persons although a 47 person within subject design was used) |
| Wilks, 2016 | Yes, no inclusion of control population | No, a questionnaire was used | Unclear, statistics not reported | No | / |
| Wilks, 2017 | Yes, no inclusion of control population | No, a questionnaire was used | Unclear, statistics not reported | No | / |

**Certainty of the body of evidence**

KNOWLEDGE

|  | **Initial grading Low [C]** | Downgrading due to |
| --- | --- | --- |
| **Limitations of study design** | -1 | See table ‘Quality of evidence’ |
| **Imprecision** | -1 | Limited sample sizes/low number of events + lack of data |
| **Inconsistency** | 0 |  |
| **Indirectness** | 0 |  |
| **Publication bias** | 0 |  |
| **QUALITY (GRADE)** | **Final grading Very low [D]** |  |

SKILLS

|  | **Initial grading Low [C]** | Downgrading due to |
| --- | --- | --- |
| **Limitations of study design** | -1 | See table ‘Quality of evidence’ |
| **Imprecision** | -1 | Limited sample sizes/low number of events + lack of data + large variability of results |
| **Inconsistency** | 0 |  |
| **Indirectness** | 0 |  |
| **Publication bias** | 0 |  |
| **QUALITY (GRADE)** | **Final grading Very low [D]** |  |

ATTITUDE

|  | **Initial grading Low [C]** | Downgrading due to |
| --- | --- | --- |
| **Limitations of study design** | -1 | See table ‘Quality of evidence’ |
| **Imprecision** | -1 | Limited sample sizes/low number of events + lack of data + large variability of results |
| **Inconsistency** | 0 |  |
| **Indirectness** | 0 |  |
| **Publication bias** | 0 |  |
| **QUALITY (GRADE)** | **Final grading Very low [D]** |  |

| **Conclusion** | *KNOWLEDGE*  ***Care for safety***  There is limited evidence in favour of FA training. It was shown that FA training resulted in a statistically significant increase of care for safety knowledge in   - 10-12 year olds: pre to post (Connolly, 2007)   ***Call for help***  There is limited evidence neither in favour of FA training nor the control. A statistically significant increase of call for help knowledge using FA training could not be demonstrated in   - 11-12 year olds: pre to 1w post, and pre to 8w follow-up (Wilks, 2016)   There is limited evidence in favour of FA training. It was shown that FA training resulted in a statistically significant increase of call for help knowledge in   - 10-12 year olds: pre to post (Connolly, 2007)   ***Emergency call***  There is limited evidence neither in favour of FA training nor the control. A statistically significant increase of emergency call knowledge using FA training could not be demonstrated in   - 5-6 year olds: compared to no intervention (Luria, 2000) - 10-12 year olds: pre to post (Connolly, 2007) - 11-12 year olds: pre to post, and pre to 8w follow-up (Wilks, 2016) - 15-18 year olds: pre to post (Celik, 2013) - 16-18 year olds: pre to post (Akca, 2016)   Note: knowledge scores were already very high in all pre to post studies, thus leaving little room for improvement  There is limited evidence in favour of FA training. It was shown that FA training resulted in a statistically significant increase of emergency call knowledge in   - 5-6 year olds: compared to no intervention (Ammirati, 2014) - 6-7 year olds: compared to no intervention (Bollig, 2009) - 10-12 year olds: compared to no intervention (Frederick, 2000)   Note: emergency call knowledge was shown not to decline over time in   - 11-12 year olds: 1w post to 8w post (Wilks, 2016)   ***Check consciousness***  There is limited evidence neither in favour of FA training nor the control. A statistically significant increase of check consciousness knowledge using FA training could not be demonstrated in   - 10-12 year olds: pre to post – assess conscious level (Connolly, 2007)   There is limited evidence in favour of FA training. It was shown that FA training resulted in a statistically significant increase of check consciousness knowledge in   - 10-12 year olds: pre to post – assess consciousness (Connolly, 2007)   ***Open the airway***  There is limited evidence neither in favour of FA training nor the control. A statistically significant increase of open the airway knowledge using FA training could not be demonstrated in   - 10-12 year olds: pre to post – assess breathing Q1 (Connolly, 2007)   There is limited evidence in favour of FA training. It was shown that FA training resulted in a statistically significant increase of open the airway knowledge in   - 10-12 year olds: pre to post – opening the airway, and assess breathing Q2 and Q3 (Connolly, 2007)   ***Recovery position***  There is limited evidence in favour of FA training. It was shown that FA training resulted in a statistically significant increase of recovery position knowledge in   - 6-7 year olds: pre to post (Uray, 2003) - 10-12 year olds: pre to post (Connolly, 2007)   Evidence is of very low quality and results of these studies are imprecise due to limited sample sizes, low number of events and lack of data.  *SKILLS*  ***Care for safety***  There is limited evidence neither in favour of FA training nor the control. A statistically significant increase of care for safety skills could not be demonstrated in   - 11-16 year olds: compared to no intervention (Campbell, 2001)   There is limited evidence in favour of FA training. It was shown that FA training resulted in a statistically significant increase of care for safety skills in   - 10-11 year olds: compared to no intervention (Frederick, 2000) - 13-16 year olds: compared to no intervention (Younas, 2006)   ***Call for help***  There is limited evidence neither in favour of FA training nor the control. A statistically significant increase of call for help skills could not be demonstrated in   - 11-12 year olds: compared to no intervention (Plotnikoff, 1989) - 13-16 year olds: compared to no intervention (Younas, 2006)   There is limited evidence in favour of FA training. It was shown that FA training resulted in a statistically significant increase of call for help skills in   - 10-11 year olds: compared to no intervention (Frederick, 2000) - 14-15 year olds: pre to post (Meissner, 2012)   Note: call for help skills were shown to decline over time in   - 14-15 year olds: post to 4m follow-up (Meissner, 2012)   ***Emergency call***  There is limited evidence neither in favour of FA training nor the control. A statistically significant increase of emergency call skills could not be demonstrated in   - 11-16 year olds: compared to sham intervention (Campbell, 2001) – scenario 2 - 13-16 year olds: compared to no intervention (Younas, 2006) - 14-18 year olds: compared to no intervention (Beskind, 2016) – time to calling 911   There is limited evidence in favour of FA training. It was shown that FA training resulted in a statistically significant increase of emergency call skills in   - 5-6 year olds: compared to no intervention (Ammirati, 2014) - 6-7 year olds: compared to no intervention (Bollig, 2009) - 10-12 year olds: compared to no intervention (Lorem, 2008) - 11-16 year olds: compared to sham intervention (Campbell, 2001) – scenario 1 - 14-15 year olds: pre to post (Meissner, 2012) - 14-18 year olds: compared to no intervention (Beskind, 2016) – call emergency number   Note: emergency call skills were shown not to decline over time in   - 14-15 year olds: post to 4m follow-up (Meissner, 2012)   ***Check consciousness***  There is limited evidence neither in favour of FA training nor the control. A statistically significant increase of check consciousness skills could not be demonstrated in   - 11-12 year olds: compared to no intervention (Moore, 1992)   There is limited evidence in favour of FA training. It was shown that FA training resulted in a statistically significant increase of check consciousness skills in   - 6-7 year olds: compared to no intervention (Bollig, 2009) - 10-11 year olds: compared to no intervention (Frederick, 2000) - 11-12 year olds: compared to no intervention (Plotnikoff, 1989) - 12 year olds: compared to no intervention (Lorem, 2008) - 13-16 year olds: compared to no intervention (Younas, 2006) - 14-15 year olds: pre to post (Meissner, 2012)   Note: check consciousness skills were shown to decline over time in   - 14-15 year olds: post to 4m follow-up (Meissner, 2012) – check responsiveness verbally   Note: check consciousness skills were shown not to decline over time in   - 14-15 year olds: post to 4m follow-up (Meissner, 2012) – check responsiveness by shaking   There is limited evidence neither in favour of older age nor younger age. A statistically significant increase in check consciousness skills could not be demonstrated with increasing age in   - 10-14 year olds (Sherif, 2005)   ***Open the airway***  There is limited evidence neither in favour of FA training nor the control. A statistically significant increase of open the airway skills could not be demonstrated in   - 11-12 year olds: compared to no intervention (Moore, 1992)   There is limited evidence in favour of FA training. It was shown that FA training resulted in a statistically significant increase of open the airway skills in   - 6-7 year olds: compared to no intervention (Bollig, 2009) - 10-11 year olds: compared to no intervention (Frederick, 2000) - 11-12 year olds: compared to no intervention (Plotnikoff, 1989) - 13-16 year olds: compared to no intervention (Younas, 2006) - 14-15 year olds: pre to post (Meissner, 2012) - 14-18 year olds: compared to no intervention (Reder, 2006)   Note: open the airway skills were shown not to decline over time in   - 14-15 year olds: post to 4m follow-up (Meissner, 2012)   There is limited evidence neither in favour of older age nor younger age. A statistically significant increase in open the airway skills could not be demonstrated with increasing age in   - 10-14 year olds (Sherif, 2005)   ***Recovery position***  There is limited evidence neither in favour of FA training nor the control. A statistically significant increase of recovery position skills could not be demonstrated in   - 10-11 year olds: compared to no intervention (Joshi, 2012) – move arms away from body, hold hand in position while rolling, attend airway   There is limited evidence in favour of FA training. It was shown that FA training resulted in a statistically significant increase of recovery position skills in   - 6-7 year olds: compared to no intervention (Bollig, 2009) - 10-11 year olds: compared to no intervention (Frederick, 2002; Joshi, 2012) – recovery position, turned on side in final position, stable final position, raise leg, move hand to cheek, use knee as pivot, roll on side - 11-12 year olds: compared to no intervention (Plotnikoff, 1989)   Evidence is of very low quality and results of these studies are imprecise due to limited sample sizes, low number of events, lack of data and large variability of results.  *ATTITUDE*  There is limited evidence in favour of FA training. It was shown that FA training resulted in a significant increase of FA self-efficacy in   - 11-16 year olds: compared to no intervention (Campbell, 2001) - 12-15 year olds (Petric, 2013) - 13-14 year olds: compared to no intervention (Engeland, 2002) - 15-16 year olds: pre to post (Creutzfeldt, 2013)   The majority of participants is confident to intervene in an emergency post FA training in   - 10-13 year olds (Bohn, 2012) - 11-12 year old boys (Lester, 1996) - 11-12 year olds (Wilks, 2017) – on a family member, on a friend - 13-14 year olds (Engeland, 2002)   The majority of participants is not confident to intervene in an emergency post FA training in   - 11-12 year old girls (Lester, 1996) - 11-12 year old (Wilks, 2017) – on a stranger   ***Recovery position***  The majority of participants is confident to apply the recovery position post FA training in   - 14-15 year olds (Meissner, 2012)   Evidence is of very low quality and results of these studies are imprecise due to limited sample sizes, low number of events, lack of data and large variability of results. |
| --- | --- |
| **Reference(s)** | **Articles**  Akca SO. *The effect of foreign body aspiration training on the knowledge level of pupils.* Braz J Otorhinolaryngology 2016, 82(4): 408-415.  Ammirati C, Gagnayre R, Amsallem C, Nemitz B, Gignon M. *Are schoolteachers able to teach first aid to children younger than 6 years?* A comparative study. BMJ Open 2014, 4**,** e005848.  Beskind DL, Stolz U, Thiede R, Hoyer R, Burns W, Brown J, Ludgate M, Tiutan T, Shane R, McMorrow D, Pleasants M, Panchal AR. *Viewing a bried chest-compression-only CPR video improves bystander CPR performance and responsiveness in high school students: a cluster randomized trial.* Resuscitation 2016, 104: 28-33.  Bohn A, Van Aken HK, Möllhoff T, Wienzek H, Kimmeyer P, Wild E, Döpker S, Lukas RP, Weber TP. *Teaching resuscitation in schools: annual tuition by trained teachers is effective starting at age 10. A four-year prospective cohort study.* Resuscitation 2012, 83: 619-625.  Bollig G, Wahl HA, Svendsen MV. *Primary school children are able to perform basic life-savind first aid measures.* Resuscitation 2009, 80: 689-692.  Campbell NR, Ayala GX, Litrownik AJ, Slymen DJ, Zavala F, Elder JP. *Evaluation of a first aid and home safety program for hispanic migrant adolescents.* American Journal of Preventive Medicine 2001, 20(4): 258-265.  Çelik N, Arikan D. *The effect of the training given to the child development students about foreign body aspiration upon their knowledge levels.* International Journal of Pediatric Otorhinolaryngology 2013, 77: 1811-1817.  Connolly M, Toner P, Connolly D, McCluskey DR. *The ‘ABC for life’ programme – Teaching basic life support in schools.* Resuscitation 2007, 72: 270-279.  Creutzfeldt J, Hedman L, Heinrichs L, Youngblood P, Felländer-Tsai L. *Cardiopulmonary resuscitation training in high school using avatars in virtual worlds: an international feasibility study.* Journal of medical Internet research 2013, 15**,** e9.  Engeland A, Reysamb E, Smedslund G, Sogaard AJ. *Effects of first-aid training in junior high schools.* Injury control and safety promotion 2002, 9(2): 99-106.  Frederick K, Bixby E, Orzel MN, Stewart-Brown S, Willett K. *An evaluation of the effectiveness of the injury minimization programme for schools (IMPS).* Injury prevention 2000, 6: 92-95.  Frederick K, Bixby E, Orzel MN, Stewart-Brown S, Willet K. *Will changing the emphasis from ‘pulseless’ to ‘no signs of circulation’ improve the recall scores for effective life support skills in children?* Resuscitation 2002, 55: 255-261.  Joshi MS, Lamb R. *Less is more. Possible ways to improve tuition of the recovery position.* Emerg Med J 2012, 29: 679-682.  Lester C, Donnelly P, Weston C, Morgan M. *Teaching schoolchildren cardiomulmonary resuscitation.* Resuscitation 1996, 31: 33-38.  Lester C, Donnelly P, Weston C. *Is peer tutoring beneficial in the context of school resuscitation training?* Health edication research theory & practice 1997, 12(3): 347-354.  Lorem T, Palm A, Wik L. *Impact of a self-instruction CPR kit on 7^th^ graders’ and adults’ skills and CPR performance.* Resuscitation 2008, 79: 103-108.  Lukas RP, Van Aken H, MölhoffT, Weber T, Rammert M, Wild E, Bohn A. *Kids save lives: a six-year longitudinal study of schoolchildren learning cardiopulmonary resuscitation: Who should do the teaching and will the effects last?* Resuscitation 2016, 101: 35-40.  Luria JW, Smith GA, Chapman JI. *An evaluation of a safety education program for kindergarten and elementary school children.* Arch pediatr adolesc med 2000, 154: 227-231.  Meissner TM, Kloppe C, Hanefeld C. *Basic life support skills of high school students before and after cardiopulmonary resuscitation training: a longitudinal investigation.* Scand J Trauma Resusc Emerg Med 2012, 20**,** 31.  Moore JP, Plotnikoff RC, Preston GD. *A study of school students’ long term retention of expired air resuscitation knowledge and skills.* Resuscitation 1992, 24: 17-25.  Petric J, Malicki M, Markovic D, Mestrovic J. *Students’ and parents’ attitudes toward basic life suppot training in primary schools.* Croat Med J 2013, 54: 376-380.  Plotnikoff R, Moore PJ. *Retention of cardiopulmonary resuscitation knowledge and skills by 11- and 12-year-old children.* The medical Journal of Asutralia 1989, 150: 297-302.  Reder S, Cummings P, Quan L. *Comparison of the three instructional methods for teaching cardiopulmonary resuscitation and use of an automatic external defibrillator to high school students.* Resuscitation 2006, 69: 443-453.  Sherif C, Erdös J, Sohm M, Schönbauer R, Rabitsch W, Schuster E, Frass M. *Effectiveness of mouth-to-mouth resuscitation performed by young adolescents on a mannequin.* American Journal of Emergency Medicine 2005, 23: 51-54.  Uray T, Lunzer A, Ochsenhofer A, Thanikkel L, Zingerle R, Lillie P, Brandl E, Sterz F, LSFA school study group. *Feasibility of life-supporting first-aid (LSFA) training as a mandatory subject in primary schools.* Resuscitation 2003, 59: 211-220.  Wilks J, Kanasa H, Pendergast D, Clark K. *Emergency response readiness for primary school children.* Aust Health Rev 2016.  Wilks J, Kanasa H, Pendergast D, Clark K. *Beach safety education for primary school children.* Int J Inj Contr Saf Promot 2017**,** 1-10.  Younas S, Raynes A, Morton S, Mackway-Jones K. *An evaluation of the effectiveness of the opportunities for resuscitation and citizen safety (ORCS) defibrillator training programme designed for older school children.* Resuscitation 2006, 71: 222-228.  **Systematic reviews**  De Buck E, Van Remoortel H, Dieltjens T, Verstraeten H, Clarysse M, Moens O, Vandekerckhove P. *Evidence-based educational pathway for the integration of first aid training in school curricula.* Resuscitation 2015, 94**,** 8-22.  Dieltjens T, De Buck E, Verstraeten H, Adriaenssens L, Clarysse M, Moens O, Devreker A, Bastiaen M, Claessens C, Verhelst K. *Evidence-based recommendations on automated external defibrillator training for children and young people in Flanders-Belgium.* Resuscitation 2013, 84**,** 1304-9.  He Z, Wynn P, Kendrick D. *Non-resuscitative first-aid training for children and laypeople: a systematic review.* Emerg Med J 2014, 31(9):763-8.  Lenson S, Mills J. *First aid knowledge retention in school children: A review of the literature.* Australasian Journal of Paramedicine 2016, 13.  Plant N, Taylor K. *How best to teach CPR to schoolchildren: a systematic review.* Resuscitation 2013, 84(4):415-21.  Reveruzzi B, Buckley L, Sheehan M. *School-Based First Aid Training Programs: A Systematic Review.* J Sch Health 2016, 86(4):266-72. |

## Resuscitation

Characteristics of included studies

| **Author, year, Country** | **Study design** | **Population** | **Comparison/Risk factor** | **Remarks** |
| --- | --- | --- | --- | --- |
| Abelairas-Gómez, 2014, Spain | Observational: cohort study | *Nr. of participants:*  721 students:   - 361 males - 360 females   *Age range:*  10-15 years  Two age groups:  10-12 years: n=370 13-15 years: n=351 | **Intervention:**  *Program:* CPR training  *Content:* Importance of CPR, recognition of cardiac arrest and basic life support manoeuvres.  Lecture + short videos + hands-on manikin training with immediate verbal feedback.  *Duration:* 1 hour | Outcomes were measured 1 hour after training.  *Skills*: CPR quality (depth, rate, leaning and hand position) on manikin measured during 2 minutes  [data were extracted per age group] |
| Alanazi, 2013, Saudi Arabia | Observational: cross-sectional study | *Nr. of participants*: 575 students:   - 70% males - 30% females   *Age range:*  Not reported  Mean ± SD:  16.5 ± 2 years | **No intervention.** | A questionnaire was used to measure the following outcomes:  *Knowledge:* CPR awareness and knowledge  *Attitude:* Willingness to perform CPR on a family member or stranger  [Only attitude data were extracted – cross-sectional design] |
| Beskind, 2016, USA | Experimental: cluster-randomized controlled trial | *Nr. of participants:*  179 students:   - Gender not reported   Three arms:  Intervention1 n=69  Intervention2 n=56  Control n=54  *Age range:*  14-18 years | **Intervention:**  *Program:* chest compression only CPR (CCO-CPR) training  *Content:*  Intervention1: Brief video illustrating the steps to perform CCO-CPR.  Intervention2: Classroom training including a power-point presentation + demonstration + hands-on manikin training on CCO-CPR.  *Duration:*  Intervention1: 1.5 min  Intervention2: 20 min  **Control:**  *Program:* Sham video  *Content:* University of Arizona recruiting video  *Duration:* 1 minute | Outcomes were measured before, immediately after and 2 months after training.  *Skills*: Responsiveness + CPR quality (depth, compression rate) on manikin measured during 2 minutes  [only post-training and follow-up data were extracted] |
| Bohn, 2012, Germany | Experimental: controlled before after study  [extracted data are from experimental before after design without control] | *Nr. of participants:*  433 students   - 223 males - 210 females   Three arms:  Intervention 1+2 n=251  Control n=182  *Age range:*  10-13 years  Two age groups:  10 year n=214  13 year n=219 | **Intervention:**  *Program:* CPR training  Intervention1: Annual training  Intervention2: Biannual training  *Content:* CPR lecture + hands-on manikin training. Focus on chest compression over ventilation.  *Duration:* 1 hour lecture + 2 hours practice  **Control:**  No intervention. | Outcomes were measured before, 1 year after (only intervention group 1+2), and two years after training.  [control group data were not extracted]  *Knowledge:* 11-item CPR questionnaire  *Skills*: CPR quality (depth, rate, tidal volume, ventilation frequency) on manikin measured during 5 minutes  [only pre-post (1 year after training) data were extracted for knowledge and skills]  *Attitude:* CPR self-efficacy, willingness, and confidence  Remark: This study includes the same data as Lukas, 2016 (see reference list).  [only data from Bohn extracted as it is more relevant for PICO] |
| Choi, 2015, Republic of Korea | Experimental: non-randomized controlled trial  [extracted data are from experimental case series design without control as no passive control group was present] | *Nr. of participants:*  187 students   - 0 males - 187 females   Two arms:  Intervention1 n=68  Intervention2 n=119  *Age:* 15-16 years | **Intervention:**  *Program:* CPR training  Intervention1: Training by nurse  Intervention2: Training by peers  *Content:* Hands-on basic life support mannequin training.  *Duration:* 1 hour | Outcomes were measured 3 months after training.  *Knowledge*: survey on CPR knowledge  [knowledge data were not extracted – case series design]  *Attitude:* willingness to perform CPR |
| Connolly, 2007, UK | Experimental: controlled before after study  [extracted data are from experimental before after design without control] | *Nr. of participants:* 79 students:   - 47 males - 32 females   Two arms:  Intervention n=46  Control n=33  *Age range:*  10-12 years  Mean intervention:  11.8 years  Mean control:  12.7 years  (SD not reported) | **Intervention:**  *Program*: CPR training (‘ABC for life’ program)  *Content*: How to approach a patient, CPR and recovery position.  Video + small group lecture + demonstration + hands-on manikin training.  *Duration*: < 2 hours  **Control:**  No intervention. | Outcomes were measured before, immediately after (only intervention group) and 6 months after training.  *Knowledge:* 22 point multiple choice questionnaire (patient approach, basic life support and CPR) |
| Creutzfeldt, 2013, Sweden | Experimental: before after study | *Nr. of participants*: 36 students   - 16 males - 20 females - 12 Swedish students - 24 USA students   *Age range:*  15-16 years  Grade 10 n=35 Grade 11 n=1 | **Intervention:**  *Program*: Virtual reality CPR training  *Content*: A training session including 3-4 emergency scenarios in virtual environment + feedback from instructor.  Swedish group: Identical to American group + a second (identical) session 6 months later.  *Duration:* 90-120 min | Outcomes were measured before (only self-efficacy and engagement mode) and immediately after training (all outcomes).  *Attitude:* Self-efficacy, concentration, perceptions regarding feasibility of training, confidence, engagement mode, mental strain, feelings/perceptions about having to act in an emergency situation  [only self-efficacy and confidence were extracted] |
| Fernandes, 2016, Brazil | Observational: before after study | *Nr. of participants:*  60 students:   - 14 males - 46 females - 30 public school - 30 private school   *Age range:*  Not reported  Mean ± SD  public school:  16.5 ± 0.68 years  Mean ± SD  private school:  16.8 ± 0.70 years | **Intervention:**  *Program:* CPR training  *Content:* BLS, chain of survival, CPR with emphasis on chest compressions, AED use  and correct positioning of the victim after recovery.  Lecture + hands-on manikin training.  *Duration:* 180 min | Outcomes were measured before, immediately after (same day) and 6 months after training.  *Knowledge*: 15-item basic life support multiple-choice questionnaire  [only overall knowledge scores were extracted] |
| Frederick, 2002, UK | Experimental: non-randomized controlled trial | *Nr. of participants:* 1096 students   - Gender not reported   Two arms:  Intervention n=542  Control n=554  *Age range:*  10-11 years | **Intervention**:  *Program:* Basic life support training (part of IMPS; see Frederick, 2000)  *Content:* Basic life support, call for help, recovery position, resuscitation.  Lecture + hands-on manikin training.  *Duration:* 70 minutes  **Control**:  No intervention. | Outcomes were measured 5 months after training.  *Skills*: Performance of basic life support and CPR on manikin |
| Hori, 2016, Japan | Observational: cohort study | *Nr. of participants*: 6352 students:   - 4966 males - 1386 females   *Age range:*  10-16 years  10-11 year n=392 12-13 year n=1798 15-16 year n=4162 | **Intervention:**  *Program*: Basic life support  *Content*: BLS lecture + demonstration + hands-on mannequin training.  *Duration:*  Total: 3 hours  Lecture: 30 minutes  Practice: 120 minutes  Break + questionnaire: 30 minutes | Outcomes were measured immediately after training.  *Attitude:* Confidence to perform CPR, appropriateness of the training content  [only confidence data were extracted] |
| Iserbyt, 2016, Belgium | Experimental: before after study | *Nr. of participants:*  313 students:   - 180 males - 133 females   Two arms:  Intervention n=198   - 104 males - 94 females   Control n=94   - 44 males - 50 females   *Age range:*  12-18 years | **Intervention:**  *Program:* BLS training  *Content:* Safe approach, responsiveness, call for help, airway, breathing, call 112, CPR. Peer learning with iPads (doer and helper) + 5 minute lecture.  *Duration:* 45 minutes | Outcomes were measured 1 week before (attitudes) and 1 week after training (skills + attitudes).  *Skills:* BLS performance was assessed individually on manikin. (only measured post intervention)  [skills data were not extracted – case series design]  *Attitudes*: Willingness to perform BLS on a family member/ best friend/ stranger + reasons not to. |
| Jiménez-Fábrega, 2009, Spain | Observational: before after study | *Nr. of participants:* 600 students:   - 53.4% males - 46.6% females   [at 1 year follow-up only n=300 students]  *Age range:*  14-16 years  Mean ± SD:  14.9 ± 0.8 years | **Intervention**:  *Program*: CPR training (“PROCES”)  *Content*: 4 CPR lectures + demonstration + 4 hands-on manikin training sessions.  *Duration*: 8 sessions (45 minutes per session) | Outcomes were measured before, immediately after and 1 year after training.  *Knowledge:* 20 multiple-choice CPR questions |
| Jones, 2007, UK | Observational: cohort study | *Nr. of participants:* 157 students:   - 74 males - 83 females   *Age range:*  9-14 years  Three age groups:  9-10 years n=55   - 27 males - 28 females   11-12 years n=54   - 23 males - 31 females   13-14 years n=48   - 24 males - 24 females | **Intervention**:  *Program*: Basic life support training    *Content*: CPR hands-on manikin training.  *Duration*: 20 minutes | Outcomes were measured immediately after training.  *Skills*: CPR quality (rate, depth, hand position) on manikin measured for 3 minutes |
| Kelley, 2006, USA | Experimental: before after study | *Nr. of participants*: 33 students:   - 17 males - 16 females   *Age range*:  12-14 years  Mean:  13.7 years | **Intervention:**  *Program*: CPR and AED training  *Content*: CPR and AED lectures + hands-on manikin demonstration.  *Duration:* 1 hour | Outcomes were measured before (knowledge, attitude), immediately after (skills) and 4 weeks (knowledge, skills) after training.  *Knowledge:* Modified AHA, CPR/AED test  *Skills*: Standardized performance checklist for CPR and AED scenario on manikin  *Attitude:* Self-developed questionnaire on CPR and AED attitudes |
| Kitamura, 2016, Japan | Observational: before after study | *Nr. of participants*: 1917 students:   - 987 males - 930 females   + data available on parents and teachers [only student data were extracted]  *Age range*:  10-12 years  Mean ± SD:  11.1 ± 0.7 years | **Intervention:**  *Program*: CPR and AED training (PUSH project)  *Content*: CPR and AED lectures + DVD + hands-on manikin training.  *Duration:* 45 min | Outcomes were measured before and immediately after training.  *Attitude:* Self-developed questionnaire on CPR attitudes, CPR and AED confidence + questionnaire on parents’ and teachers’ views about CPR training and AED use  [only student data were extracted] |
| Lester, 1996, UK | Observational: case series study | *Nr. of participants:* 41 students:   - 17 males - 24 females   *Age range:*  11-12 years | **Intervention:**  *Program*: CPR training  *Content*: CPR + ethics.  Lecture + video + hands-on manikin training.  *Duration*: 3 sessions  (1 hour per session) | Outcomes were measured immediately after and 9 days (only skills) after training.  *Knowledge:* Multiple choice CPR questionnaire  *Skills*: Performance checklist first aid + CPR on manikin  *Attitude:* CPR attitudes  [only attitude data were extracted – case series] |
| Lester, 1997, UK | Experimental: non-randomized controlled trial  [extracted data are from experimental case series design without control as no passive control group was present] | *Nr. of participants:* 243 students:   - 128 males - 115 females   Two arms:  Intervention1 n=106  Intervention2 n=137  [Intervention2 data were not included in the analysis]  *Age range:*  11-12 years | **Intervention:**  *Program*: CPR training  Intervention1:  *Content*: CPR + ethics.  Lecture + video + hands-on manikin training.  *Duration*: 3 sessions  (1 hour per session)  Intervention2:  *Program*: CPR training (teacher + pupils). | Outcomes were measured immediately after training.  *Knowledge:* 10-point CPR multiple choice questionnaire  *Skills*: Performance checklist first aid + CPR on manikin  *Attitude:* CPR confidence, willingness, reasons not to  [only attitude data were extracted – case series] |
| Lorem, 2008, Norway | Observational: case - control study | *Nr. of participants:* 102 students  + data available on family and friends [ only student data were extracted]  Two arms:  Intervention n=52:   - 43% males - 57% females   Control n=50:   - 34% males - 66% females   *Age range:*  12 years  Mean ± SD intervention:  12 ± 0.02 years  Mean ± SD control:  11.9 ± 0.05 years | **Intervention**:  *Program*: CPR training  *Content*: CPR video + information folder + hands-on manikin training.  *Duration*: 45 min (30 min DVD)  **Control**:  No intervention before testing. | Intervention: Outcomes were measured 1 week after training.  Control: Outcomes were measured before training.  *Skills:* Video based performance in scenario + CPR quality (depth, tidal volume) on manikin measured for 2 minutes |
| Ma, 2015, Hong Kong | Observational: cross-sectional study | *Nr. of participants*: 383 students:   - 199 males - 184 females   *Age range:*  15-16 years | **No intervention.** | The following outcomes were measured:  *Knowledge:* Self-developed CPR questionnaire  *Attitude:* CPR attitudes and barriers to CPR  [only attitude data were extracted - cross-sectional design] |
| Meissner, 2012, Germany | Observational: before after study | *Nr. of participants*: 129 students:   - 57 males - 72 females   *Age range*:  14-15 years  Mean ± SD:  14.6 ± 1.4 years | **Intervention:**  *Program*: CPR, AED and healthy lifestyle training  *Content*: Sudden cardiac arrest, recovery position, CPR, AED use.  Lecture + demonstration + hands-on manikin training + workshops on healthy lifestyle and physical activity.  *Duration:* 45 minute lecture + 30 minute practice | Outcomes were measured before, immediately after (same day), and four months after training.  *Skills:* Checklist + CPR quality (frequency, depth, time until start of chest compressions) on manikin  *Attitude:* FA, health issues, and self-confidence assessment  [only skills + self-confidence data were extracted] |
| Moore, 1992, Australia | Experimental: non-randomized controlled trial | *Nr. of participants:* 102 students:   - Gender not reported   Two arms:  Intervention n=51  Control n=51  *Age range:*  11-12 years | **Intervention**:  *Program*: CPR training  *Content*: CPR video + demonstration + hands-on manikin training.  *Duration*: 140 min  **Control**:  No intervention. | Outcomes were measured 5 years after training.  *Knowledge:* 14-item multiple choice questionnaire  *Skills*: Performance checklist on manikin CPR + CPR quality (ventilations) measured for 2 minutes |
| Naqvi, 2011, Pakistan | Observational: before after study | *Nr. of participants:*  30 students:   - 11 males - 19 females   *Age range:*  11-15 years  Mean ± SD:  13.4 ± 1.7 years | **Intervention**:  *Program*: CPR training  *Content*: CPR lecture + video + demonstration + hands-on manikin training.  *Duration*: 5 hours | Outcomes were measured before, immediately after and 3 months after training.  *Knowledge*: 25-item questionnaire  *Skills*: Performance checklist on manikin BLS (only measured at post and follow-up)  [skills data not extracted, not measured before - case series design] |
| Omi, 2008, Japan | Observational: cross-sectional study | *Nr. of participants:* 3316 students:   - 51% males - 49% females   *Age range:*  15-17 years  Mean ± SD:  16 ± 1 years | **No intervention**. | The following outcomes were measured:  *Attitude*: CPR willingness within 5 cardiac arrest scenarios (stranger, trauma, child, elderly and relative), reasons not to perform CPR |
| Parashar, 2010, India | Observational: before after study | *Nr. of participants:*  40 students:   - 20 males - 20 females   *Age range:*  14-16 years  Two age groups:  14-15 years n=23  15-16 years n=17 | **Intervention**:  *Program*: CPR training  *Content*: Basic life support.  *Duration*: Not reported. | Outcomes were assessed before and 8 days after training.  *Knowledge*: BLS questionnaire  *Skills*: Observation BLS checklist |
| Parnell, 2006, New Zealand | Observational: cross-sectional study | *Nr. of participants:* 494 students:   - 218 males - 250 females - 26 missing data   *Age range:*  16-17 years | **No intervention**. | The following outcomes were measured:  *Knowledge:* First aid and resuscitation questionnaire  *Attitude*: FA and CPR education, CPR confidence, willingness to perform ventilation on a family member/ stranger  [only attitude data were extracted – cross-sectional design] |
| Petric, 2013, Croatia | Observational: cohort study  [extracted data are from observational cross-sectional design] | *Nr. of participants:*  301 students   - 143 males - 158 females   +361 parents [only student data were extracted]  *Age range:*  12-15 years  Median: 13 years | **No intervention.** | The following outcomes were measured:  *Attitude*: Basic life support training + willingness and fear to apply BLS  [only willingness and fear to apply BLS data were extracted] |
| Plotnikoff, 1989, Australia | Experimental: controlled before after study | *Nr. of participants:*  45 students:   - Gender not reported   Two arms: Intervention n=20 Control n=25  *Age range:*  11-12 years | **Intervention**:  *Program:* CPR training (see Moore, 1992)  **Control**:  No intervention. | Outcomes were measured before, immediately after and 5 months after training.  *Knowledge:* 22-item CPR questionnaire  *Skills*: CPR performance on manikin  [only post-training and follow-up data were extracted] |
| Rahman, 2013, Malaysia | Experimental: cluster randomized controlled trial | *Nr. of participants*: 477 students   - 210 males - 267 females   Two arms:  Intervention n=251   - 73 males - 178 females   Control n=226   - 137 males - 89 females   *Age range:*  Secondary school | **Intervention:**  *Program*: CPR training  *Content*: CPR lecture + video + pamphlet + hands-on manikin training.  *Duration:* 15 minute lecture (other durations not mentioned)  **Control:**  *Program*: Booklet on the hazard of smoking  *Duration:* Not reported. | Outcomes were measured before and two weeks after training.  *Knowledge:* Heart disease, risk factors for cardiac arrest, CPR  *Attitude:* CPR, mouth-to-mouth ventilation, BLS teaching  [only post-training data were extracted] |
| Reder, 2006, USA | Experimental: cluster randomized controlled trial | *Nr. of participants:* 779 students   - 424 males - 355 females   Four arms:  Intervention1 n=213   - 128 males - 85 females   Intervention2 n=170   - 79 males - 91 females   Intervention3 n=206   - 107 males - 99 females   Control n=190   - 110 males - 80 females   *Age range:*  14-18 years | **Intervention:**  *Program:* CPR and AED training  Intervention1:  *Content:* Interactive computer training.  *Duration:* 45 minutes  Intervention2:  *Content:* Interactive computer training + instructor-led hands-on manikin training.  *Duration:* 90 minutes  Intervention3:  *Content:* Traditional classroom instruction (video + demonstration) + instructor-led hands-on manikin training.  *Duration:* 90 minutes  **Control**:  No intervention. | Outcomes were measured 2 days after and 2 months after training.  *Knowledge:* 10-item CPR and AED questionnaire  *Skills*: CPR (ventilations + chest compressions) and AED performance on a manikin  [only skills data were extracted, since no separate knowledge scores for CPR and AED were available]  [only post-training data were extracted] |
| Ribeiro, 2013, Brazil | Experimental: before after study | *Nr. of participants:*  202 students   - Male/female ratio wrongly reported   Two cohorts:  Public school n=81  Private school n=121  *Age range:*  13-15 years | **Intervention:**  *Program:* CPR training  *Content:* Recognition of cardiac arrest, CPR (ventilation + chest compression), AED use.  Video presentation + hands-on manikin training.  *Duration:* 120 minutes | Outcomes were measured before, immediately after, and 6 months after training.  *Knowledge*: 25-item CPR and AED questionnaire |
| Rivera-Tovar, 1990, USA | Experimental: controlled before after study | *Nr. of participants:*  38 students   - 20 males - 18 females   *Age range:*  11-12 years  Mean:  males: 11 years 5 months  females: 11 years 4 months | *Program*: CPR training  **Intervention 1**:  *Content*: CPR lecture + demonstration + memory training (rehearsal) for 3 different scenarios.  **Intervention 2**:  *Content*: CPR lecture + demonstration + memory training (rehearsal + feedback – imprecise elaboration) for 3 different scenarios.  **Intervention 3:**  *Content*: CPR lecture + demonstration + memory training (rehearsal + detailed feedback - precise elaboration) for 3 different scenarios.  *Duration*: 85-120 minutes total  **Control:**  No intervention. | Outcomes were measured before, immediately after, and 1, 3 and 5 months after training  Skills: Correct CPR responses in sequence in 3 different scenarios |
| Sherif, 2005, Austria | Observational: cohort study | *Nr. of participants:*  57 students:   - 30 males - 27 females   *Age range:*  10 years n=27  14 years n=30 | **Intervention**:  *Program*: CPR training  *Content*: CPR lecture + demonstration + hands-on manikin training.  *Duration*: 30 minutes | Outcomes were measured immediately after training.  *Skills*: Check consciousness, call for help, start CPR. CPR quality (ventilations) on manikin |
| Taniguchi, 2012, Japan | Observational: cross-sectional study | *Nr. of participants*:  5312 students:   - 2695 males - 2617 females   Three surveys:  1998 n=479   - 196 males - 283 females   2006 n=3125   - 1594 males - 1531 females     2010 n=1708   - 905 males - 803 females   *Age range:*  15-18 years | **No intervention.** | The following outcomes were measured:  *Attitude:* Willingness to perform chest compressions and/or mouth-to-mouth ventilation + reasons not to |
| Toner, 2007, UK | Observational: before after study | *Nr. of participants:*  82 students   - 29 males - 53 females   *Age range:*  10-12 years  Mean: 10.7 years  (SD not reported) | **Intervention:**  *Program:* CPR training ‘ABC for life’ program (see Connolly, 2007) | Outcomes were measured before and immediately after training.  *Knowledge*: 22-point CPR questionnaire |
| Uray,  2003, Austria | Observational:  before after study | *Nr. of participants:*  47 students:   - 20 males - 27 females     *Age range:*  6-7 years | **Intervention:**  *Program:* First aid training  *Content*: Emergency call, CPR, AED, recovery position, bleeding and burns.  Demonstration + hands-on manikin training.  *Duration:* 1 week | Outcomes were measured before and immediately after training.  *Knowledge:* Questionnaire in which students had to place three cartoon-like illustrations in the correct sequence |
| Vetter, 2016, USA | Experimental:  controlled before after study  [extracted data are from experimental before after design without control as intervention2 is not a passive control] | *Nr. of participants:*  412 students:   - 150 males - 262 females   Two arms:  Intervention1 n=230  Intervention2 n=182  *Age range:*  Not reported  Mean ± SD:  15.9 ± 1.3 years | **Intervention:**  *Program:* CPR and AED training (health class)  Intervention1:  *Content*: Usual CPR and AED training + peer education + participation in CPR/AED Olympics.  Intervention2:  *Content*: Usual CPR and AED training.  *Duration:* Not reported. | Outcomes were measured before, immediately after (but before CPR/AED Olympics), and 12 months after training.  *Knowledge*: CPR and AED questionnaire  *Skills*: CPR test on manikin (call 911, recognition of CPR need, hand placement, AED placement, effectiveness of ventilations and compressions)  *Attitude*: Willingness to perform CPR using mouth to mouth or hands only in different scenarios (relative, child, old, blood, vomit) |
| Wafik,  2014, Egypt | Observational: before after study | *Nr. of participants:* 100 students:   - Gender not reported     *Age range:*  11-16 years  Mean ± SD:  13.2 ± 0.8 years | **Intervention:**  *Program:* First aid training  *Content:* wounds, poisoning, chemicals,  electrocution, hemorrhage, burns, fractures, choking and basic life support.  Lecture + question time + group discussion.  *Duration:* 6 sessions (1 hour/session) | Outcomes were measured before, immediately after and 2 months after training.  *Knowledge*: 32 item questionnaire.  *Skills*: Performance on five first aid scenarios (choking, burns, poisoning, and fractures). |
| Wilks, 2016, Australia | Observational: before after study | *Nr. of participants*: 107 students:   - 51 males - 56 females   *Age range*:  11-12 years  Mean ± SD:  11.4 ± 0.5 years | **Intervention:**  *Program*: First aid, CPR and beach safety training  *Content*:   - Beach safety - Leadership and team bonding exercises - First aid: sprains, bleeding, choking, heart attack, allergic reactions - Hands-on manikin training   *Duration:* 1 day | Outcomes were measured before, 1 week after, and 8 weeks after training.  *Knowledge:* 50-item quiz on emergency services and life-supporting first aid |
| Younas, 2006, UK | Experimental: non-randomized controlled trial | *Nr. of participants:*  59 students:   - Gender not reported   Two arms:  Intervention n=34  Control n=25  *Age range:*  13-16 years | **Intervention:**  *Program*: CPR/AED training  *Content*: CPR and AED use. Hands-on manikin training.  *Duration*: Not reported.  **Control:**  No intervention | Outcomes were measured 6 months after training.  *Skills*: 10-item checklist during cardiac arrest scenario |

**Synthesis of findings**

| **Resuscitation (CPR)** | | | | | |
| --- | --- | --- | --- | --- | --- |
| **Outcome** | | **Comparison** | **Effect Size** | **#studies, # participants** | **Reference** |
| *Knowledge* | | | | | |
| - 6-7 years | | | | | |
| Knowledge: CPR | Pre vs post | | Statistically significant:  MD: 30%, 95%CI [16%,46%] λ  (p<0.05)  *in favour of first aid training* | 1, 47 vs 47 §  (within subject) | Uray, 2003 |
| - 10 years | | | | | |
| Knowledge: CPR (/11) | Pre vs post | | Statistically significant:  9.16±0.12 vs 7.71±0.14  MD: 1.45* £  (p<0.05)  *in favour of first aid training* | 1, 90 vs 90 §  (within subject) | Bohn, 2012 |
| - 10-12 years | | | | | |
| Knowledge: CPR (/22) | Pre vs post | | Statistically significant:  12.5 (57.2%) vs 17 (77.7%) §  MD: 4.5* £  (p<0.001)  *in favour of first aid training* | 1, 190 vs 190  (within subject) | Toner, 2007 |
| Knowledge: Chest compression |  |  | Question 1  Statistically significant:  31 (67.4%) vs 39 (84.8%) §  RR: 1.26 * £  (p<0.05)  *in favour of first aid training* | 1, 46 vs 46  (within subject) | Connolly, 2007 |
|  |  |  | Question 2  Statistically significant:  6 (13.0%) vs 36 (78.3%) §  RR: 6.00* £  (p<0.001)  *in favour of first aid training* |  |  |
| Knowledge: Compression rate |  |  | Statistically significant:  30 (6.5%) vs 45 (97.8%) §  RR: 1.50* £  (p<0.001)  *in favour of first aid training* |  |  |
| Knowledge: Ventilation |  |  | Statistically significant:  22 (47.8%) vs 36 (78.3%) §  RR: 1.64 *£  (p<0.01)  *in favour of first aid training* |  |  |
| Knowledge: Ventilation ratio |  |  | Statistically significant:  11 (23.9%) vs 44 (95.7%) §  RR: 4.00* £  (p<0.001)  *in favour of first aid training* |  |  |
| - 11-12 years | | | | | |
| Knowledge: CPR  (/14) | FA training vs no intervention | | Not statistically significant:  10.84±1.97 vs 10.59±1.22  MD: 0.25, 95%CI [-0.39;0.89]*  (p=0.44) | 1, 51 vs 51 § | Moore, 1992 |
| Knowledge: CPR (/22) |  |  | Statistically significant:  15.05±3.54 vs 10.60±2.47  MD: 4.45, 95%CI [2.62;6.28]  (p<0.00001)*  *in favour of first aid training* | 1, 20 vs 25 §  (post) | Plotnikoff, 1989 |
|  |  |  | Statistically significant:  12.40±3.70 vs 9.32±2.34  MD: 3.08, 95%CI [1.22;4.94]  (p=0.001)*  *in favour of first aid training* | 1, 20 vs 25 §  (5 month follow-up) |  |
| Knowledge: Ratio 30:2 | Pre vs 1 week post | | Statistically significant:  0.03±0.17 vs 0.90±0.31  MD: 0.87* £†  (p<0.001)  *in favour of first aid training* | 1, 107 vs 102 §  (within subject) | Wilks, 2016 |
|  | Pre vs 8 week follow-up | | Statistically significant:  0.03±0.17 vs 0.82±0.39µ  MD: 0.79* £†  (p<0.001)  *in favour of first aid training* | 1, 107 vs 105 §  (within subject) |  |
|  | 1 week post vs 8 week follow-up | | Not statistically significant:  0.90±0.31 vs 0.82±0.39  MD: -0.08* £†  (p=0.159) | 1, 102 vs 105 §  (within subject) |  |
| - 11-15 years | | | | | |
| Knowledge: CPR (/25) | Pre vs post | | Statistically significant:  10.17±4.17 vs 18.10±3.78  MD: 7.93* £  (p<0.0001)  *in favour of first aid training* | 1, 30 vs 30 §  (within subject) | Naqvi, 2011 |
|  | Post vs 3 month follow-up | | Not statistically significant:  18.10±3.78 vs 17.67±4.12  MD: -0.43* £†  (p=0.314) |  |  |
| Knowledge: CPR (score >60%) | Pre vs post | | Statistically significant:  0 (0%) vs 94 (94%) §  RR: 189.00* £  (p<0.001)  *in favour of first aid training* | 1, 100 vs 100  (within subject) | Wafik, 2014 |
|  | Pre vs 2 month follow-up | | Statistically significant:  0 (0%) vs 85 (85%) §  RR: 171.00* £  (p<0.001)  *in favour of first aid training* |  |  |
| - 13 years | | | | | |
| Knowledge: CPR (/11) | Pre vs post | | Statistically significant:  10.09±0.16 vs 9.10±0.19  MD: 0.99* £  p<0.05  *in favour of first aid training* | 1, 54 vs 54 §  (within subject) | Bohn, 2012 |
| - 13-15 years | | | | | |
| Knowledge: CPR | Pre vs post | | Statistically significant:  5.82±1.15 vs 3.04±1.11  MD: 2.78* £  (p<0.05)  *in favour of first aid training* | Public school  1, 81 vs 81 §  (within subject) | Ribeiro, 2013 |
|  |  |  | Statistically significant:  6.06±1.13 vs 3.46±1.15  MD: 2.60* £  (p<0.05)  *in favour of first aid training* | Private school    1, 121 vs 121 §  (within subject) |  |
| Knowledge: CPR sequence |  |  | Statistically significant:  5.47±1.59 vs 2.20±0,97  MD: 3.27* £  (p<0.05)  *in favour of first aid training* | Public school  1, 81 vs 81 §  (within subject) |  |
|  |  |  | Statistically significant:  5.86±1.43 vs 2.39±1.12  MD: 3.47* £  (p<0.05)  *in favour of first aid training* | Private school    1, 121 vs 121 §  (within subject) |  |
| Knowledge: Chest compression |  |  | Statistically significant:  1.14±0.66 vs 2.78±0.56  MD: 1.64±0.56 £†  (p<0.05)*  *in favour of first aid training* | Public school  1, 81 vs 81 §  (within subject) |  |
|  |  |  | Statistically significant:  1.13±0.80 vs 2.83±0.48  MD: 1.72±0.92 £†  (p<0.05)*  *in favour of first aid training* | Private school    1, 121 vs 121 §  (within subject) |  |
| Knowledge: Ventilation |  |  | Statistically significant:  1.51±0.99 vs 3.86±0.38  MD: 2.35* £†  (p<0.05)  *in favour of first aid training* | Public school  1, 81 vs 81 §  (within subject) |  |
|  |  |  | Statistically significant:  1.63±0.82 vs 3.81±0.61  MD: 2.18* £†  (p<0.05)  *in favour of first aid training* | Private school    1, 121 vs 121 §  (within subject) |  |
| - 14-16 years | | | | | |
| Knowledge: CPR (/10) | Pre vs post | | Statistically significant:  4.25 vs 5.4 λ  MD: 1.15* £  (p<0.001)  *in favour of first aid training* | 1, 600 vs 600  (within subject) | Jiménez-Fabrega, 2009 |
|  | Post vs 1 year follow-up | | Statistically significant:  5.4 vs 5 λ  MD: 0.4* £  (p<0.001)  *decline over time* | 1, 300 vs 300 §  (within subject) |  |
| Knowledge: CPR score | Pre vs post | | Statistically significant:  27.03% vs 74.92%  % difference: 47.89%* £  (p<0.05)  *in favour of first aid training* | 1, 40 vs 40 §  (within subject) | Parashar, 2010 |
| - 14-17 years | | | | | |
| Knowledge: CPR | FA training vs no intervention | | Statistically significant:  79.99±13.59 vs 63.28±13.50  MD: 16.71, 95%CI [14.28;19.14]*  (p<0.001)  *in favour of first aid training* | 1, 251 vs 226 | Rahman, 2013 |
| - 14-18 years | | | | | |
| Knowledge: CPR and AED | Pre vs post | | Statistically significant:  54.9±16.9 vs 77.0±15.0  MD: 22.1* £  (p<0.001)  *in favour of first aid training* | CPR/AED + olympics  1, 230 vs 230 §  (within subject) | Vetter, 2016 |
|  |  |  | Statistically significant:  54.2±16.5 vs 74.8±16.0  MD: 20.6* £  (p<0.001)  *in favour of first aid training* | CPR/AED  1, 182 vs 182 §  (within subject) |  |
|  | Post vs 12 month follow-up | | Statistically significant:  79.1±11.1 vs 73.7±13.2  MD: -5.4* £  (p=0.02)  *decline over time* | CPR/AED + olympics  1, 230 vs 230 §  (within subject) |  |
|  |  |  | Not statistically significant:  79.6±11.4 vs 75.5±12.1  MD: -4.1* £†  (p=0.09) | CPR/AED  1, 182 vs 182 §  (within subject) |  |
| - 16-17 years | | | | | |
| Knowledge: CPR (%) | Pre vs post | | Statistically significant:  30.2±12.2 vs 62.4±19.6  MD: 32.2* £  (p<0.05)  *in favour of first aid training* | Public school  1, 30 vs 30 §  (within subject) | Fernandes, 2016 |
|  |  |  | Statistically significant:  42±14 vs 86±7.8  MD: 44* £  (p<0.05)  *in favour of first aid training* | Private school    1, 30 vs 30 §  (within subject) |  |
|  | Pre vs 6 month follow-up | | Statistically significant:  30.2±12.2 vs 45.6±16  MD: 15.4* £  (p<0.05)  *in favour of first aid training* | Public school  1, 30 vs 30 §  (within subject) |  |
|  |  |  | Statistically significant:  42±14 vs 65±12.4  MD: 23* £  (p<0.05)  *in favour of first aid training* | Private school    1, 30 vs 30 §  (within subject) |  |
| *Skills* | | | | | |
| ***Overall resuscitation (CPR) skills*** | | | | | |
| - 10-11 years | | | | | |
| Skills: Ratio 15:2 | | FA training vs no intervention | Statistically significant:  128 (24%) vs 9 (2%) §  RR: 15.47, 95%CI [7.95;30.11]  (p<0.00001)*  *in favour of first aid training* | 1, 534 vs 581 §(power analysis) | Frederick, 2002 |
| - 11-12 years | | | | | |
| Skills: CPR in correct sequence | FA training vs no intervention | | Statistically significant  80% (intervention 1), 61% (intervention 2) and 63% (intervention 3) vs control condition (0.8%) £  p<0.05  *in favour of first aid training* | 1, 42 total § £  (post through follow-up periods) | Rivera-Tovar, 1990 |
| - 12 years | | | | | |
| Skills: Ratio 30:2 | FA training vs no intervention | | Statistically significant:  47 (90.5%) vs 4 (11%) §  RR: 11.30, 95%CI[4.40;29.04]*  (p<0.05)  *in favour of first aid training* | 1, 52 vs 50 | Lorem, 2008 |
| - 12-14 years | | | | | |
| Skills: CPR and AED use | Immediately after training | | 7/7 actions correct: 25/33 (75.7%) £†  6/7 actions correct: 29/33 (87.8%) £† | 1, 33 vs 33 §  (within subject) | Kelley, 2006 |
|  | 4 weeks after training | | 7/7 actions correct: 25/33 (75.7%) £†  6/7 actions correct: 28/33 (84.8%) £† |  |  |
| - 13-16 years | | | | | |
| Skills: Ratio 15:2 | FA training vs no intervention | | Statistically significant:  17 (50%) vs 3 (12%) §  RR: 4.17, 95%CI [1.37;12.69]  (p=0.01)*  *in favour of first aid training* | 1, 34 vs 25 | Younas, 2006 |
| - 14-15 years | | | | | |
| Skills: overall CPR score | Pre vs post | | Statistically significant:  Median (IQR): 4 (3-6) vs 10 (9-10)  Median difference: 6* £  (p<0.001)  *in favour of first aid training* | 1, 129 vs 103 §  (within subject) | Meissner, 2012 |
|  | Post vs 4 month follow-up | | Not statistically significant:  Median (IQR): 10 (9-10) vs 10 (9-10)  Median difference: 0* £†  (p=0.89) | 1, 103 vs 98 §  (within subject) |  |
| - 14-16 years | | | | | |
| Skills: CPR  (/10) | Pre vs post | | Statistically significant:  4.25 vs 7 λ  MD: 2.75* £  (p<0.001)  *in favour of first aid training* | 1, 600 vs 600  (within subject) | Jiménez-Fabrega, 2009 |
|  | Post vs 1 year follow-up | | Statistically significant:  7 vs 6.5 λ  MD: 0.5* £  (p<0.001)  *decline over time* | 1, 300 vs 300 §  (within subject) |  |
| Skills: CPR score | Pre vs post | | Statistically significant:  18.11% vs 69.50%  % difference: 51.39%* £  (p<0.05)  *in favour of first aid training* | 1, 40 vs 40 §  (within subject) | Parashar, 2010 |
| - 14-18 years | | | | | |
| Skills: CPR and AED | Pre vs post | | Statistically significant:  15.1±22.9 vs 76.3±20.8  MD: 61.2* £  (p<0.001)  *in favour of first aid training* | CPR/AED + olympics  1, 230 vs 230 §  (within subject) | Vetter, 2016 |
|  |  |  | Statistically significant:  16.9±22.6 vs 77.4±23.2  MD: 60.5* £  (p<0.001)  *in favour of first aid training* | CPR/AED  1, 182 vs 182 §  (within subject) |  |
|  | Post vs 12 month follow-up | | Not statistically significant:  88.6±12.1 vs 87.9±15.4  MD: -0.7* £†  (p=0.77) | CPR/AED + olympics  1, 230 vs 230 §  (within subject) |  |
|  |  |  | Statistically significant:  90.5±11.7 vs 78.9±21.9  MD: -11.6* £  (p<0.01)  *decline over time* | CPR/AED  1, 182 vs 182 §  (within subject) |  |
| ***Resuscitation (CPR) skills - chest compressions*** | | | | | |
| - 9-14 years | | | | | |
| Skills: Compression rate (rate/min) | 9-10 years vs  11-12 years | | Not statistically significant:  108±31 vs 109±26  MD: -1.0, 95%CI [-11.73;9.73]  (p=0.86)* | 1, 55 vs 54 § | Jones, 2007 |
|  | 9-10 years vs  13-14 years | | Not statistically significant:  108±31 vs 116±25  MD: -8.0, 95%CI [-18.82;2.82]  (p=0.16)* | 1, 55 vs 48 § |  |
|  | 11-12 years vs  13-14 years | | Not statistically significant:  109±26 vs 116±25  MD: -7.0, 95%CI [-16.9;2.9]  (p=0.17)* | 1, 54 vs 48 § |  |
| Skills: Compression rate (Number correct) | 9-10 years vs  11-12 years | | Not statistically significant:  10 (18%) vs 14 (26%) §  RR: 0.70, 95% CI [0.34;1.44] ¥  (p=0.33)* | 1, 55 vs 54 |  |
|  | 9-10 years vs  13-14 years | | Not statistically significant:  10 (18%) vs 14 (30%) §  RR: 0.62, 95%CI [0.31;1.27] ¥  (p=0.19)* | 1, 55 vs 48 |  |
|  | 11-12 years vs  13-14 years | | Not statistically significant:  14 (26%) vs 14 (30%) §  RR: 0.89, 95%CI [0.47;1.67] ¥  (p=0.71)* | 1, 54 vs 48 |  |
| Skills: Compression depth (mm) | 9-10 years vs  11-12 years | | Statistically significant:  23±6.9 vs 28±9.0  MD: -5.0, 95%CI [-8.01;-1.99]  (p=0.002)*  *in favour of older age* | 1, 55 vs 54 § |  |
|  | 9-10 years vs  13-14 years | | Statistically significant:  23±6.9 vs 35±7.8  MD: -12.0, 95%CI [-14.86;-9.14]  (p<0.00001)*  *in favour of older age* | 1, 55 vs 48 § |  |
|  | 11-12 years vs  13-14 years | | Statistically significant:  28±9.0 vs 35±7.8  MD: -7.0, 95%CI [-10.26;-3.74]  (p<0.0001)*  *in favour of older age* | 1, 54 vs 48 § |  |
| Skills: Compression depth (Number correct) | 9-10 years vs  11-12 years | | Statistically significant:  0 (0%) vs 10 (19%) §  RR: 0.05, 95%CI [0.0;0.78]  (p=0.03)*  *in favour of older age* | 1, 55 vs 54 |  |
|  | 9-10 years vs  13-14 years | | Statistically significant:  0 (0%) vs 21 (45%) §  RR: 0.02, 95%CI [0.0;0.33]  (p=0.006)*  *in favour of older age* | 1, 55 vs 48 |  |
|  | 11-12 years vs  13-14 years | | Statistically significant:  10 (19%) vs 21 (45%) §  RR: 0.42, 95%CI [0.22;0.81]  (p=0.009)*  *in favour of older age* | 1, 54 vs 48 |  |
| Skills: 100% correct hand placement | 9-10 years vs  11-12 years | | Not statistically significant:  22 (40%) vs 12 (22%) §  RR: 1.80, 95%CI [0.99;3.26] ¥  (p=0.05)*  *in favour of younger age* | 1, 55 vs 54 |  |
|  | 9-10 years vs  13-14 years | | Not statistically significant:  22 (40%) vs 15 (31%) §  RR: 1.28, 95%CI [0.75;2.17] ¥  (p=0.36)* | 1, 55 vs 48 |  |
|  | 11-12 years vs  13-14 years | | Not statistically significant:  12 (22%) vs 15 (31%) §  RR: 0.71, 95%CI [0.37;1.36] ¥  (p=0.31)* | 1, 54 vs 48 |  |
| Skills: 80-100% correct hand placement | 9-10 years vs  11-12 years | | Not statistically significant:  32 (58%) vs 25 (46%) §  RR: 1.26, 95%CI [0.87;1.81] ¥  (p=0.22)* | 1, 55 vs 54 |  |
|  | 9-10 years vs  13-14 years | | Not statistically significant:  32 (58%) vs 25 (52%) §  RR: 1.12, 95%CI [0.79;1.59] ¥  (p=0.54)* | 1, 55 vs 48 |  |
|  | 11-12 years vs  13-14 years | | Not statistically significant:  25 (46%) vs 25 (52%) §  RR: 0.89, 95%CI [0.60;1.32] ¥  (p=0.56)* | 1, 54 vs 48 |  |
| - 10 years | | | | | |
| Skills: Compression frequency (rate/min) | Pre vs post | | Statistically significant:  23.95±3.62 vs 59.81±2.22  MD: 35.86* £  (p<0.05)  *in favour of first aid training* | 1, 90 vs 90 §  (within subject) | Bohn, 2012 |
| Skills: Compression depth (mm) |  |  | Statistically significant:  11.92±1.56 vs 34.75±1.17  MD: 22.83* £  (p<0.05)  *in favour of first aid training* |  |  |
| - 10-11 years | | | | | |
| Skills: Hand placement | FA training vs no intervention | | Statistically significant:  182 (34%) vs 22 (4%) §  RR: 9.0, 95%CI [5.88;13.79]  (p<0.00001)*  *in favour of first aid training* | 1, 534 vs 581 §(power analysis) | Frederick, 2002 |
| Skills: Compression rate (100/min) |  |  | Statistically significant:  135 (25%) vs 7 (1%) §  RR: 20.98, 95%CI [9.91;44.45]  (p<0.00001)*  *in favour of first aid training* |  |  |
| - 10-15 years | | | | | |
| *Skills:* compression depth (mm) | 10-12 years vs  13-15 years | | Statistically significant:  32.6±8.6 vs 41.3±8.0  MD: -8.7, 95%CI [-9.91;-7.49] *  (p<0.001)  *in favour of older age* | 1, 370 vs 351 | Abelairas-Gómez, 2014 |
| *Skills:* >70% correct compression depth |  |  | Statistically significant:  7 (1.9%) vs 39 (11.1%) §  RR: 0.17, 95%CI [0.08;0.38]  (p<0.0001)*  *in favour of older age* |  |  |
| *Skills:* >50% correct compression depth |  |  | Statistically significant:  10 (2.7%) vs 58 (16.5%) §  RR: 0.16, 95%CI [0.08;0.31]  (p<0.00001)*  *in favour of older age* |  |  |
| *Skills:* compression rate |  |  | Statistically significant:  131.6±16.7 vs 124.5±16.3  MD: 7.10, 95%CI [4.69;9.51]*  (p<0.001)  *in favour of older age* |  |  |
| *Skills:* correct compression fraction |  |  | Statistically significant:  5.0±13.1 vs 16.3±24.2  MD: -11.30; 95%CI [-14.16;-8.44]*  (p<0.001)  *in favour of older age* |  |  |
| *Skills:* >70% correct chest compressions |  |  | Statistically significant:  4 (1.1%) vs 24 (6.8%) §  RR: 0.16, 95%CI [0.06;0.45]  (p=0.0006)*  *in favour of older age* |  |  |
| *Skills:* >50% correct chest compressions |  |  | Statistically significant:  9 (2.4%) vs 36 (10.3%) §  RR: 0.24, 95%CI [0.12;0.49]  (p<0.0001)*  *in favour of older age* |  |  |
| *Skills:* insufficient chest compression |  |  | Statistically significant:  247.8±53.3 vs 196.7±80.3  MD: 51.10, 95%CI [41.1;61.1]*  (p<0.001)  *in favour of older age* |  |  |
| *Skills:* incorrect chest recoil (leaning) |  |  | Statistically significant:  25.5±55.3 vs 46.3±73.9  MD: -20.80, 95%CI [-30.37;-11.23]*  (p<0.001)  *in favour of older age* |  |  |
| *Skills:* incorrect hand position |  |  | Statistically significant:  11.2±44.5 vs 3.7±22.2  MD: 7.50, 95%CI [2.41;12.59]*  (p=0.004)  *in favour of older age* |  |  |
| *Skills*: arbitrary cardiac output index |  |  | Statistically significant:  4270.9±1166.0 vs 5112.9±1092.2  MD: -842.0, 95%CI [-1006.84;-677.16]*  (p<0.001)  *in favour of older age* |  |  |
| - 11-12 years | | | | | |
| Skills: Effective compressions | FA training vs no intervention | | Statistically significant:  40.05±27.61 vs 0.48±2.40  MD: 39.57, 95%CI [27.43;51.71]  (p<0.00001)*  *in favour of first aid training* | 1, 20 vs 25 § (post) | Plotnikoff, 1989 |
|  |  |  | Statistically significant:  13.20±16.88 vs 1.24±6.00  MD: 11.96, 95%CI [4.20;19.72]  (p=0.003)  *in favour of first aid training* | 1, 20 vs 25 § (5 month follow-up) |  |
| Skills: Incorrect hand positions |  |  | Not statistically significant:  2.10±6.6 vs 0.32±1.6  MD: 1.78, 95%CI [--1.18;4.74] ¥  (p=0.24) | 1, 20 vs 25 § (post) |  |
|  |  |  | Five month follow-up:  1.9±8.27 vs 0±0  MD: 1.9 £†  statistical analysis not possible | 1, 20 vs 25 § (5 month follow-up) |  |
| - 12 years | | | | | |
| Skills: Compression rate (rate/min) | FA training vs no intervention | | Statistically significant:  Median [95%CI]: 84 [79;107] vs 113 [107;118]  Median difference: 29* £†  (p<0.05)  *in favour of first aid training* | 1, 52 vs 50 § | Lorem, 2008 |
| Skills: Compression depth (mm) |  |  | Statistically significant:  Median [95%CI]: 23 [20;27] vs 36 [34;38]  Median difference: 13* £†  (p<0.05)  *in favour of first aid training* |  |  |
| - 13 years | | | | | |
| Skills: Compression frequency (rate/min) | Pre vs post | | Statistically significant:  21.77±4.83 vs 73.01±2.96  MD: 51.24* £  (p<0.05)  *in favour of first aid training* | 1, 54 vs 54 §  (within subject) | Bohn, 2012 |
| Skills: Compression depth (mm) |  |  | Statistically significant:  12.64±2.09 vs 38.91±1.56  MD: 26.27* £  (p<0.05)  *in favour of first aid training* |  |  |
| - 14-15 years | | | | | |
| Skills: Chest compression | Pre vs post | | Statistically significant:  37 (29%) vs 102 (99%) §  RR: 3.45* £  (p<0.001)  *in favour of first aid training* | 1, 129 vs 103  (within subject) | Meissner, 2012 |
|  | Post vs 4 month follow-up | | Not statistically significant:  102 (99%) vs 97 (99%) §  RR: 1.00* £†  (p=1.0) | 1, 103 vs 98  (within subject) |  |
| Skills: Compression frequency | Boys vs girls | | Not statistically significant:  100.1±23.5 vs 97.9±21.4  MD: 2.20, 95%CI [-5.65;10.05]  (p=0.58)* | 1, 57 vs 72 §  (post) |  |
|  |  |  | Not statistically significant:  105.3±19.7 vs 100.5±23.3  MD: 4.80, 95%CI [-2.62;12.22]  (p=0.22)* | 1, 57 vs 72 §  (4 month follow-up) |  |
| Skills: Compression depth |  |  | Statistically significant:  5±0.6 vs 4.6±0.5  MD: 0.40, 95%CI [0.21;0.59]  (p<0.0001)*  *in favour of boys* | 1, 57 vs 72 §  (post) |  |
|  |  |  | Statistically significant:  5.2±0.6 vs 4.7±0.6  MD: 0.50, 95%CI [0.29;0.71]  (p<0.00001)*  *in favour of boys* | 1, 57 vs 72 §  (4 month follow-up) |  |
| Skills: sufficient depth at ≥50% of compressions |  |  | Not statistically significant:  33 (57.4%) vs 36 (50.3%) §  RR: 1.16, 95%CI [0.84;1.59] ¥  (p=0.37)* | 1, 57 vs 72  (post) |  |
|  |  |  | Not statistically significant:  29 (50.9%) vs 30 (41.8%) §  RR: 1.22, 95%CI [0.84;1.77] ¥  (p=0.30)* | 1, 57 vs 72  (4 month follow-up) |  |
| Skills: Time until start of chest compression |  |  | Not statistically significant:  35.4±17.9 vs 33.1±15.8  MD: 2.30, 95%CI [-3.61;8.21] ¥  (p=0.44) | 1, 57 vs 72 §  (post) |  |
|  |  |  | Not statistically significant:  42.2±21.1 vs 42.0±25.2  MD: 0.20, 95%CI [-7.79;8.19] ¥  (p=0.96) | 1, 57 vs 72 §  (4 month follow-up) |  |
| - 14-18 years | | | | | |
| Skills: Start compression within 2 minutes | Brief video vs no intervention | | Statistically significant:  58 (100%) vs 35 (74.1%) §  RR: 1.34, 95%CI [1.13;1.59]  (p=0.0007, still significant after Bonferroni correction p=0.0056)*  *in favour of first aid training* | 1, 58 vs 47  (post) | Beskind, 2016 |
|  | Classroom training vs no intervention | | Statistically significant:  53 (98.2%) vs 35 (74.1%) §  RR: 1.32, 95%CI [1.11;1.56]  (p=0.002, still significant after Bonferroni correction p=0.0056)*  *in favour of first aid training* | 1, 54 vs 47  (post) |  |
|  | Brief video vs no intervention | | Not statistically significant:  57 (98.3%) vs 44 (93.8%) §  RR: 1.05, 95%CI [0.97;1.14]  (p=0.25)* | 1, 58 vs 47  (2 month follow-up) |  |
|  | Classroom training vs no intervention | | Not statistically significant:  54 (100%) vs 44 (93.8%) §  RR: 1.07; 95%CI[0.98;1.16]  (p=0.12) | 1, 54 vs 47  (2 month follow-up) |  |
| Skills: Compression frequency (rate/min) | Brief video vs no intervention | | Statistically significant:  104±26.6 vs 82±33.0  MD: 22.0, 95%CI [10.43;33.57]  (p=0.0002, still significant after Bonferroni correction p=0.0056)*  *in favour of first aid training* | 1, 58 vs 47 §  (post) |  |
|  | Classroom training vs no intervention | | Statistically significant:  93±25.6 vs 82±33.0  MD: 11.0, 95%CI [-0.65;22.65]  (p=0.065 but significant after Bonferroni correction)*  *in favour of first aid training* | 1, 54 vs 47 §  (post) |  |
|  | Brief video vs no intervention | | Not statistically significant:  99±26.6 vs 103±27.2  MD: -4.00, 95%CI [-14.36;6.36] ¥  (p=0.45)* | 1, 58 vs 47 §  (2 month follow-up) |  |
|  | Classroom training vs no intervention | | Not statistically significant:  97±25.6 vs 103±27.2  MD: -6.00, 95%CI [-16.35;4.35] ¥  (p=0.26)* | 1, 54 vs 47 §  (2 month follow-up) |  |
| Skills: Compression depth (mm) | Brief video vs no intervention | | Not statistically significant:  32.3±11.0 vs 28.3±13.6  MD: 4.0, 95%CI [-0.81;8.81]  (p=0.10) | 1, 58 vs 47 §  (post) |  |
|  | Classroom training vs no intervention | | Statistically significant:  34.0±11.7 vs 28.3±13.6  MD: 5.70, 95%CI [0.71;10.69]  (p=0.03, still significant after Bonferroni correction)*  *In favour of first aid training* | 1, 54 vs 47 §  (post) |  |
|  | Brief video vs no intervention | | Statistically significant:  31.1±11.0 vs 35.7±11.9  MD: -4.60; 95%CI [-9.03;-0.17]  (p=0.04, still significant after Bonferroni correction p=0.0056)* | 1, 58 vs 47 §  (2 month follow-up) |  |
|  | Classroom training vs no intervention | | Not statistically significant  34.5±12.0 vs 35.7±11.9  MD: -1.20; 95%CI [-5.87;3.47] ¥  (p=0.61)* | 1, 54 vs 47 §  (2 month follow-up) |  |
| Skills: hands-off time | Brief video vs no intervention | | Not statistically significant  16.6±18.6 vs 27.5±33.7  MD: -10.9, 95%CI [-21.66;-0.14]  (p=0.05)* | 1, 58 vs 47 §  (post) |  |
|  | Classroom training vs no intervention | | Not statistically significant:  8.1±12.5 vs 27.5±33.7  MD: -19.4, 95%CI [-29.60;-9.20]  (p=0.0002, but no longer significant after Bonferroni correction)* | 1, 54 vs 47 §  (post) |  |
|  | Brief video vs no intervention | | Not statistically significant:  6.7±10.3 vs 12.5±17.4  MD: -5.8; 95%CI [-11.44;-0.16]  (p=0.04, but no longer significant after Bonferroni correction)* | 1, 58 vs 47 §  (2 month follow-up) |  |
|  | Classroom training vs no intervention | | Statistically significant:  9.0±11.7 vs 12.5±17.4  MD: -3.50; 95%CI [-9.37;2.37]  (p=0.24, but significant after Bonferroni correction)  *In favour of first aid training* | 1, 54 vs 47 §  (2 month follow-up) |  |
| Skills: Hand position | Computer vs no intervention | | Statistically significant:  112 (54%) vs 36 (19%) §  RR: 2.81, 95%CI [2.04;3.87]  (p<0.00001)*  *in favour of first aid training* | 1, 207 vs 187  (post) | Reder, 2006 |
|  | Computer + demo vs no intervention | | Statistically significant:  118 (74%) vs 36 (19%) §  RR: 3.83, 95%CI [2.82;5.21]  (p<0.00001)*  *in favour of first aid training* | 1, 160 vs 187  (post) |  |
|  | Video + demo vs no intervention | | Statistically significant:  133 (68%) vs 36 (19%) §  RR: 3.52, 95%CI [2.59;4.80]  (p<0.00001)*  *in favour of first aid training* | 1, 196 vs 187  (post) |  |
|  | Computer vs no intervention | | Statistically significant:  73 (40%) vs 41 (27%) §  RR: 1.48, 95%CI [1.08;2.03]  (p=0.02)*  *in favour of first aid training* | 1, 182 vs 151  (2 month follow-up) |  |
|  | Computer + demo vs no intervention | | Statistically significant:  93 (61%) vs 41 (27%) §  RR: 2.24, 95%CI [1.67;2.99]  (p<0.00001)*  *in favour of first aid training* | 1, 153 vs 151  (2 month follow-up) |  |
|  | Video + demo vs no intervention | | Statistically significant:  84 (53%) vs 41 (27%) §  RR: 1.96, 95%CI [1.45;2.64]  (p<0.0001)*  *in favour of first aid training* | 1, 158 vs 151  (2 month follow-up) |  |
| Skills: Compression successful | Computer vs no intervention | | Statistically significant:  36 (21%) vs 14 (9%) §  RR: 2.36, 95%CI [1.32;4.21]  (p=0.004)*  *in favour of first aid training* | 1, 170 vs 156  (post) |  |
|  | Computer + demo vs no intervention | | Statistically significant:  38 (28%) vs 14 (9%) §  RR: 3.09, 95%CI [1.75;5.46]  (p<0.0001)*  *in favour of first aid training* | 1, 137 vs 156  (post) |  |
|  | Video + demo vs no intervention | | Statistically significant:  46 (29%) vs 14 (9%) §  RR: 3.20, 95%CI [1.84;5.59]  (p<0.0001)*  *in favour of first aid training* | 1, 160 vs 156  (post) |  |
|  | Computer vs no intervention | | Statistically significant:  32 (19%) vs 12 (10%) §  RR: 1.87, 95%CI [1.00;3.47] ¥  (p=0.05)*  *in favour of first aid training* | 1, 170 vs 119  (2 month follow-up) |  |
|  | Computer + demo vs no intervention | | Not statistically significant:  27 (19%) vs 12 (10%) §  RR: 1.87, 95%CI [0.99;3.53] ¥  (p=0.05)*  *in favour of first aid training* | 1, 143 vs 119  (2 month follow-up) |  |
|  | Video + demo vs no intervention | | Statistically significant:  35 (23%) vs 12 (10%) §  RR: 2.27, 95%CI [1.23;4.18]  (p=0.009)*  *in favour of first aid training* | 1, 153 vs 119  (2 month follow-up) |  |
| Skills: Compression frequency (rate/min) | Pre vs post | | Statistically significant:  73.1 vs 116.4  MD: 43.3* £†  (p<0.001)  *in favour of first aid training* | CPR/AED + olympics  1, 230 vs 230 §  (within subject) | Vetter, 2016 |
|  |  |  | Statistically significant:  67.7 vs 106.4  MD: 38.7* £†  (p<0.001)  *in favour of first aid training* | CPR/AED  1, 182 vs 182 §  (within subject) |  |
| Skills: Compression depth (mm) |  |  | Statistically significant:  27.3 vs 40  MD: 12.7* £†  (p<0.001)  *in favour of first aid training* | CPR/AED + olympics  1, 230 vs 230 §  (within subject) |  |
|  |  |  | Statistically significant:  26.1 vs 38.5  MD: 12.4* £†  (p<0.001)  *in favour of first aid training* | CPR/AED  1, 182 vs 182 §  (within subject) |  |
| Skills: Hands-off time |  |  | Statistically significant:  17.8 vs 7.7  MD: -10.1* £†  (p<0.001)  *in favour of first aid training* | CPR/AED + olympics  1, 230 vs 230 §  (within subject) |  |
|  |  |  | Statistically significant:  17 vs 9.2  MD: -7.8* £†  (p<0.001)  *in favour of first aid training* | CPR/AED  1, 182 vs 182 §  (within subject) |  |
| ***Resuscitation (CPR) skills - Ventilations*** | | | | | |
| - 10 years | | | | | |
| Skills: Ventilation frequency (rate/min) | Pre vs post | | Statistically significant:  0.00±0.00 vs 4.81±0.66  MD: 4.81* £  p<0.05  *in favour of first aid training* | 1, 90 vs 90 §  (within subject) | Bohn, 2012 |
| Skills: Ventilation volume (ml) |  |  | Statistically significant:  0.00±0.00 vs 579.81±54.50  MD: 579.81* £  p<0.05  *in favour of first aid training* |  |  |
| - 10-11 years | | | | | |
| Skills: Ventilation successful | FA training vs no intervention | | Statistically significant:  112 (21%) vs 17 (3%) §  RR: 7.17, 95%CI [4.36;11.78]  (p<0.00001)*  *in favour of first aid training* | 1, 534 vs 581 §(power analysis) | Frederick, 2002 |
| - 10-14 years | | | | | |
| Skills: Tidal volume (ml) | 10 year vs 14 year | | Statistically significant:  326±308 vs 724±491  MD: -398.00, 95%CI [-608.63;-187.37]  (p=0.0002)*  *in favour of older age* | 1, 27 vs 30 § | Sherif, 2005 |
| Skills: Time until ventilation (seconds) |  |  | Not statistically significant:  32.0±6.9 vs 33.1±6.3  MD: -1.10, 95%CI [-4.54;2.34]  (p=0.53)* |  |  |
| Skills: 1-5^th^ ventilation (seconds) |  |  | Not statistically significant  53.8±9.7 vs 51.4±6.8  MD: 2.40, 95%CI [-1.99;6.79]  (p=0.28)* |  |  |
| - 11-12 years | | | | | |
| Skills: Start ventilation | FA training vs no intervention | | Not statistically significant:  0 (0%) vs 4 (7.8%) §  RR: 0.11, 95%CI [0.01;2.01] ¥  (p=0.14)* | 1, 51 vs 51 § | Moore, 1992 |
| Skills: Time to ventilation (seconds) |  |  | Statistically significant:  24.64±20.56 vs 37.02±24.26  MD: -12.38, 95%CI [-21.11;-3.65]  (p=0.005)*  *in favour of first aid training* |  |  |
| Skills: Adequate ventilations in 10 seconds (>0.8l) |  |  | Statistically significant:  2.30±2.30 vs 0.28±1.40  MD: 2.02, 95%CI [0.44;1.70]  (p=0.007)*  *in favour of first aid training* | 1, 20 vs 25 § (post) | Plotnikoff, 1989 |
|  |  |  | Not statistically significant:  1.05±2.86 vs 0.08±0.04  MD: 0.97, 95%CI -0.28;2.22] ¥  (p=0.13)* | 1, 20 vs 25 § (5 month follow-up) |  |
| Skills: Adequate ventilations in 2 min (>0.8l) |  |  | 5.20±3.58 vs 0±0  MD: 5.20* £†  statistical analysis not possible | 1, 20 vs 25 § (post) |  |
|  |  |  | 9.15±12.33 vs 0±0  MD: 9.15* £†  statistical analysis not possible | 1, 20 vs 25 § (5 month follow-up) |  |
| - 12 years | | | | | |
| Skills: Tidal volume (ml) | FA training vs no intervention | | Statistically significant:  Median [95%CI]: 770 [678;907] vs 0 [0;0]  Median difference: 770* £†  (p<0.05)  *in favour of first aid training* | 1, 52 vs 50 § | Lorem, 2008 |
| - 13 years | | | | | |
| Skills: Ventilation frequency (rate/min) | Pre vs post | | Statistically significant:  0.00±0.00 vs 4.74±0.88  MD: 4.74* £  p<0.05  *in favour of first aid training* | 1, 54 vs 54 §  (within subject) | Bohn, 2012 |
| Skills: Ventilation volume (ml) |  |  | Statistically significant:  0.00±0.00 vs 536.83±72.81  MD: 536.83* £  p<0.05  *in favour of first aid training* |  |  |
| - 13-16 years | | | | | |
| Skills: Ventilation | FA training vs no intervention | | Not statistically significant:  11 (32.4%) vs 8 (32%) §  RR: 1.01, 95%CI [0.48;2.14] ¥  (p=0.98)* | 1, 34 vs 25 | Younas, 2006 |
| - 14-15 years | | | | | |
| Skills: Ventilation | Pre vs post | | Statistically significant:  30 (23%) vs 95 (92%) §  RR: 3.97* £  (p<0.001)  *in favour of first aid training* | 1, 129 vs 103  (within subject) | Meissner, 2012 |
|  | Post vs 4 month follow-up | | Not statistically significant:  95 (92%) vs 90 (91.8%) §  RR: 1.00* £†  (p=0.39) | 1, 103 vs 98  (within subject) |  |
| - 14-18 years | | | | | |
| Skills: Chest rise | Computer vs no intervention | | Statistically significant:  66 (32%) vs 22 (12%) §  RR: 2.71, 95%CI [1.75;4.21]  (p<0.00001)*  *in favour of first aid training* | 1, 207 vs 187  (post) | Reder, 2006 |
|  | Computer + demo vs no intervention | | Statistically significant:  91 (57%) vs 22 (12%) §  RR: 4.83, 95%CI [3.19;7.32]  (p<0.00001)*  *in favour of first aid training* | 1, 160 vs 187  (post) |  |
|  | Video + demo vs no intervention | | Statistically significant:  116 (59%) vs 22 (12%) §  RR: 5.03, 95%CI [3.34;7.58]  (p<0.00001)*  *in favour of first aid training* | 1, 196 vs 187  (post) |  |
|  | Computer vs no intervention | | Statistically significant:  48 (26%) vs 21 (14%) §  RR: 1.89, 95%CI [1.18;3.00]  (p=0.008)*  *in favour of first aid training* | 1, 183 vs 151  (2 month follow-up) |  |
|  | Computer + demo vs no intervention | | Statistically significant:  79 (51%) vs 21 (14%) §  RR: 3.69, 95%CI [2.41;5.65]  (p<0.00001)*  *in favour of first aid training* | 1, 154 vs 151  (2 month follow-up) |  |
|  | Video + demo vs no intervention | | Statistically significant:  92 (58%) vs 21 (14%) §  RR: 4.19, 95%CI [2.76;6.36]  (p<0.00001)*  *in favour of first aid training* | 1, 158 vs 151  (2 month follow-up) |  |
| Skills: % correct ventilations | Computer vs no intervention | | Not statistically significant:  8 (5%) vs 5 (3%) §  RR: 1.48, 95%CI [0.49;4.42] ¥  (p=0.49)* | 1, 169 vs 156  (post) |  |
|  | Computer + demo vs no intervention | | Statistically significant:  19 (14%) vs 5 (3%) §  RR: 4.33, 95%CI [1.66;11.28]  (p=0.003)*  *in favour of first aid training* | 1, 137 vs 156  (post) |  |
|  | Video + demo vs no intervention | | Statistically significant:  24 (15%) vs 5 (3%) §  RR: 4.68, 95%CI [1.83;11.96]  (p=0.001)*  *in favour of first aid training* | 1, 160 vs 156  (post) |  |
|  | Computer vs no intervention | | Not statistically significant:  14 (8%) vs 5 (4%) §  RR: 1.97, 95%CI [0.73;5.33] ¥  (p=0.18)* | 1, 169 vs 119  (2 month follow-up) |  |
|  | Computer + demo vs no intervention | | Statistically significant:  16 (11%) vs 5 (4%) §  RR: 2.66, 95%CI [1.01;7.05]  (p=0.048)*  *in favour of first aid training* | 1, 143 vs 119  (2 month follow-up) |  |
|  | Video + demo vs no intervention | | Statistically significant:  21 (14%) vs 5 (4%) §  RR: 3.27, 95%CI [1.27;8.41]  (p=0.01)*  *in favour of first aid training* | 1, 153 vs 119  (2 month follow-up) |  |
| *Attitudes* | | | | | |
| ***Willingness to perform CPR*** | | | | | |
| - 11-12 years | | | | | |
| Attitude: Willingness to perform CPR | Post training | | Would definitely perform CPR:  On (grand)parents: >80% £†  On a relative: 30 (73%) £†  On a same age friend: 18 (44%) £†  On an adult friend: 10 (24%) £† | 1, 41 §  (case series) | Lester, 1996 |
|  |  |  | Very probably respond: 18 (8%) £†  Probably respond: 87 (38%) £†  Possibly respond: 83 (36%) £†  Unlikely to respond: 40 (18%) £† | 1, 228 §  (case series) | Lester, 1997 |
|  | Boys vs girls | | Statistically significant:  Very probable or probable:  43 (36%) vs 62 (56%)  RR: 0.62, 95%CI [0.47;0.83]*  (p<0.01)  *in favour of girls* | 1, 120 vs 108  (case series) |  |
| - 12-14 years | | | | | |
| Attitude: Willingness to perform CPR | Cross-sectional | | on a family member: 27 (82%)  on a stranger: 24 (73%)  on a stranger if no mouth-to-mouth: 29 (88%) | 1, 33 § £† | Kelley, 2006 |
| - 12-18 years | | | | | |
| Attitude: Willingness to perform CPR | Pre vs post | | On a family member  Girls (n=133)  Not statistically significant:  68 (51%) vs 70 (53%) §  RD: +2%, 95%CI [-10%;+13%] ¥  (p=0.60)  Boys (n=180)  Statistically significant:  88 (49%) vs 104 (58%) §  RD: +9%, 95%CI [-1%;+19%]  (p=0.01)  *In favour of first aid training* | 1, 313  (within subject) | Iserbyt, 2016 |
|  |  |  | On a friend  Girls (n=133)  Not statistically significant:  65 (49%) vs 68 (51%) §  RD: +2%, 95%CI [-9%;+14%] ¥  (p=058)  Boys (n=180)  Not statistically significant:  86(48%) vs 95 (53%) §  RD: +5%, 95%CI [-5%;+15%] ¥  (p=0.15) |  |  |
|  |  |  | On a stranger  Girls (n=133)  Not statistically significant:  15 (11%) vs 17 (13%) §  RD: +2%, 95%CI [-6%;+10%] ¥  (p=0.90)  Boys (n=180)  Not statistically significant:  14 (8%) vs 18(10%) §  RD: +2%, 95%CI [-4%;+8%] ¥  (p=0.82) |  |  |
| - 14-17 years | | | | | |
| Attitude: Willingness to perform CPR | FA training vs no intervention | | Statistically significant:  22.09±4.59 vs 18.72±4.50  MD: 3.37, 95%CI [2.55;4.19]*  (p=0.001)  *In favour of first aid training* | 1, 251 vs 226 | Rahman, 2013 |
| - 14-18 years | | | | | |
| Attitude: Willingness to perform mouth-to-mouth | Cross-sectional | | On a family member:  Male respondent: 67.08%  Female respondent: 66.67%  A stranger:  Male respondent: 41.65%  Female respondent: 23.56% | 1, 575 £† | Alanazi, 2013 |
| Attitude: Willingness to provide hands only CPR (Likert scale: /5) | Pre vs post | | Statistically significant:  Relative: 1.4 vs 1.7 λ£†  Child: 1.55 vs 1.8 λ£†  Old: 1.6 vs 1.95 λ£†  Blood: 2.35 vs 2.55 λ£†  Vomit: 2.55 vs 2.75 λ£†  (p<0.05)  *In favour of first aid training* | 1, 412 vs 412 (within subject | Vetter, 2016 |
| Attitude: Willingness to provide CPR including mouth-to-mouth (Likert scale: /5) |  |  | Statistically significant:  Relative: 2 vs 1.7 λ£†  Child: 1.9 vs 2.25 λ£†  Old: 2.25 vs 2.75 λ£†  Blood: 3.2 vs 3.4 λ£†  Vomit: 3.5 vs 3.75 λ£†  (p<0.05)  *In favour of first aid training* |  |  |
| - 15-16 years | | | | | |
| *Attitude:* Willingness to perform CPR | 3 months post training  (no pre or control) | | 37 (55.2%) | Traditional training  1, 67 § £† | Choi, 2015 |
|  |  |  | 77 (64.7%) | Peer-assisted training  1, 119 § £† |  |
| Attitude: Willingness to perform CPR | Cross-sectional | | 83.3% yes | 1, 383 § £† | Ma, 2015 |
| Attitude: If I have adequate CPR knowledge and skills, I will perform CPR to people in need |  |  | Strongly agree/Agree: 79.9%  Neutral: 14.4%  Strongly disagree/Disagree: 5.7% |  |  |
| Attitude: Even if the victim is a stranger, I will have intention to perform CPR |  |  | Strongly agree/Agree: 72.1%  Neutral: 22.7%  Strongly disagree/Disagree: 5.2% |  |  |
| Attitude: If the victim is a family member, I would be more willing to perform CPR |  |  | Strongly agree/Agree: 54.6%  Neutral: 14.6%  Strongly disagree/Disagree: 30.8% |  |  |
| - 15-17 years | | | | | |
| Attitude: Willingness to perform CPR (chest + mouth to mouth) | Cross-sectional | | Total: 27%  On a stranger: 15%  Trauma: 18%  Child: 37%  Elderly: 25%  Relative: 41% | 1, 3316 £† | Omi, 2008 |
| Attitude: Willingness to perform CPR (chest compressions only or chest + mouth to mouth) |  |  | Total: 59%  On a stranger: 53%  Trauma: 51%  Child: 63%  Elderly: 57%  Relative: 69% |  |  |
| Attitude: Willingness to perform CPR | previous CPR training vs no previous CPR training | | Statistically significant:  35% vs 18%  RR: 0.51*  (p<0.002)  *in favour of first aid training* |  |  |
|  |  |  | Statistically significant:  Perform chest compressions only 33% vs 30%  RR: 0.90*  (p<0.05)  *in favour of first aid training* |  |  |
| - 15-18 years | | | | | |
| Attitude: Willingness to perform chest compressions | Cross-sectional trend in 1998 vs 2006 vs 2010 | | Not statistically significant:   - On a family member:   85 % vs 68% vs 72% £†  (p=0.516)   - On a stranger:   73% vs 53% vs 59% £†  (p=0.664)   - On a child:   80% vs 64% vs 67% £†  (p=0.652)   - On a trauma patient (blood on face):   66% vs 51% vs 57% £†  (p=0.733) | 1, 479 vs 3125 vs 1708 | Taniguchi, 2012 |
| Attitude: Willingness to perform chest compressions + mouth-to-mouth |  |  | Not statistically significant:   - On a family member:   53% vs 41% vs 42% £†  (p=0.414)   - On a stranger:   13% vs 15% vs 16% £†  (p=0.864)   - On a child:   50% vs 37% vs 37% £†  (p=0.364)   - On a trauma patient (blood on face):   18% vs 18% vs 18% £†  (p=0.887) |  |  |
| - 16-17 years | | | | | |
| Attitude: willingness to provide CPR | Cross-sectional | | Family member: 84% £†  Complete stranger: 63% £† | 1, 494 | Parnell, 2006 |
|  | Positive attitude vs negative attitude towards FA and CPR | | Family member:  Not statistically significant:  267 (86%) vs 71 (84%)  RR: 0.98; 95% CI [0.89;1.09]*  (p>0.05) | 1, 311 vs 84 |  |
|  |  |  | Stranger:  Statistically significant:  218 (70%) vs 39 (47%) §  RR: 0.66, 95% CI [0.52;0.84]*(p<0.001)  *In favour of positive attitude* |  |  |
| ***Confidence to perform CPR*** | | | | | |
| - 10-11 years | | | | | |
| Attitude: If you saw someone in need of BLS, do you think you could help that person? | | Post intervention | Yes, I’m confident that I can: 20% λ  Yes, I probably can: 45% λ  I’m not too confident, but I think I will try: 25% λ  I probably can’t help: 8% λ  I can’t help: 2% λ | 1, 392 § £†  (cohort study) | Hori, 2016 |
| - 10-12 years | | | | | |
| Attitude: understand how to perform CPR | Post  (no pre or control) | | Yes or maybe yes:  1874 (97.7%) £† | 1, 1899 vs 1899  (within subject) | Kitamura, 2016 |
| Attitude: If someone collapses, can you do something? | Pre vs post | | Yes or maybe yes:  1529 (80.5%) vs 1783 (93.9%) RR: 1.17* £†  Those who were negative at pre (n=370):  Statistically significant:  72.3% positively changed their willingness after training (p<0.001) £†  *In favour of first aid training*  Those who were positive at pre (n=1529): 99.0% remained positive after training £† |  |  |
| - 10 or 13 years | | | | | |
| Attitude: “I’m good at CPR!” | Pre vs 2 year follow-up | | 63 (43.5%) vs 110 (76.7%)  RR: 1.75* £† | 1, 144 vs 144§  (within subject) | Bohn, 2012 |
|  | Pre vs 4 year follow-up | | 63 (43.5%) vs 123 (85.1%)  RR: 1.95* £† |  |  |
| Attitude: Self-confidence | 4 year follow-up  (no pre or control) | | Feel capable to carry out lay resuscitation: 85.2% £†  Feel capable to intervene in an emergency: 83.1% £†  Too afraid to intervene: 25.3% £† |  |  |
| - 12-13 years | | | | | |
| Attitude: If you saw someone in need of BLS, do you think you could help that person? | Post intervention | | Yes, I’m confident that I can:  7% λ  Yes, I probably can: 40% λ  I’m not too confident, but I think I will try: 38% λ  I probably can’t help: 12% λ  I can’t help: 3% λ | 1, 1798 £†  (cohort study) | Hori, 2016 |
| - 14-15 years | | | | | |
| Attitude: Sufficient knowledge about CPR | Post intervention | | 90.8% | 1, 103 § £† | Meissner, 2012 |
| Attitude: would have dared to apply CPR before training vs would dare to apply CPR following training |  |  | 35 (26.9%) vs 102 (99.2%) §  RR: 3.65* £† | 1, 129 vs 103  (within subject) |  |
| - 15-16 years | | | | | |
| Attitude: If you saw someone in need of BLS, do you think you could help that person? | Post intervention | | Yes, I’m confident that I can: 8% λ  Yes, I probably can: 30% λ  I’m not too confident, but I think I will try: 44% λ  I probably can’t help: 14% λ  I can’t help: 4% λ | 1, 4162 £†  (cohort study) | Hori, 2016 |
| Attitude: CPR confidence before training vs after training | Pre vs post (both recorded post-intervention in exit questionnaire) | | Swedish group:  median (IQR): 4.0 (3.0-4.0) vs 4.2 (4.0-5.0)  US group:  median (IQR): 2.5 (2.0-3.0) vs 4.0 (4.0-4.5) | 1, 12 vs 12 (Swedish group) + 24 vs 24 (US group) §£†  (within subject) | Creutzfeldt, 2013 |
| Attitude: I am satisfied with the level of knowledge to perform CPR | Cross-sectional:  15-16 years | | Strongly agree/Agree: 23.0% £†  Neutral: 28.2% £†  Strongly disagree/Disagree: 48.8% £† | 1, 383 § | Ma, 2015 |
| ***Barriers and facilitators to CPR*** | | | | | |
| - 11-12 years | | | | | |
| Attitude: Barriers to CPR | Post training  (no pre or control) | | Vomit: 160 (69%)  Dirty: 115 (49%)  Unpleasant smell: 120 (52%) | 1, 228 § £†  (case series) | Lester, 1997 |
|  |  |  | Open-ended questions:  Blood in or around mouth: 54(24%)  Serious injuries: 33 (14%)(  Dangerous situations: 33 (14%)  HIV: 16 (7%)  Other known disease: 11 (5%)  Public place: 14 (6%)  Stranger: 16(7%) |  |  |
|  |  |  | Vomit: 24%  Known or visible disease: 17%  Serious injuries or bleeding (no % reported) | 1, 41 § £†  (case series) | Lester, 1996 |
| - 12-14 years | | | | | |
| Attitude: uncomfortable performing CPR because of | Cross-sectional | | Mouth to mouth breathing: 13 (39%)  Fear of infection: 8 (24%)  I might harm the patient: 15 (45%)  I might get sued: 6 (18%)  None: 8 (24%) | 1, 33 § £† | Kelley, 2006 |
| - 12-15 years | | | | | |
| Attitude: Not afraid to apply BLS despite infection | Cross-sectional | | strongly agree: 23.6%  agree: 19.3%  neither agree nor disagree: 31.1%  disagree: 17.6%  strongly disagree: 8.4% | 1, 301 § £† | Petric, 2013 |
| Attitude: Not afraid to apply BLS despite potential harm they might cause |  |  | strongly agree: 15.7%  agree: 15.7%  neither agree nor disagree: 39.9%  disagree: 17.7%  strongly disagree: 10.9% |  |  |
| Attitude: Not afraid to apply BLS despite lack of CPR knowledge |  |  | strongly agree: 37.8%  agree: 24.7%  neither agree nor disagree: 19.2%  disagree: 8.2%  strongly disagree: 10.0% |  |  |
| Attitude: More people would be willing to apply BLS if its training only  involved chest compressions (no mouth to mouth) |  |  | strongly agree: 28.8%  agree: 25.7%  neither agree nor disagree: 27.1%  disagree: 8.9%  strongly disagree: 9.6% |  |  |
| - 12-18 years | | | | | |
| Attitude: Reasons not to perform CPR - Panic | Pre vs post | | Girls (n=133)  Not statistically significant:  25 (19%) vs 32 (24%) §  RD: +5% , 95%CI [-5%;15%] ¥  (p=0.28)  Boys (n=180)  Not statistically significant:  23 (13%) vs 22 (12%) §  RD: -1% , 95%CI [-8%;+6%] ¥  (p=0.56)  Significant gender effect at post-training test (p<0.01) | 1, 313  (within subject) | Iserbyt, 2016 |
| Attitude: Reasons not to perform CPR – Doing something wrong |  |  | Girls (n=133)  Not statistically significant:  51 (38%) vs 51 (38%) §  RD: 0% , 95%CI [-12%;12%] ¥  (p=0.74)  Boys (n=180)  Not statistically significant:  47 (26%) vs 38 (21%) §  RD: -5% , 95%CI [-14%;+4%] ¥  (p=0.08)  Significant gender effect at post-training test (p<0.01)  Significant gender effect at pre-training test (p<0.05) |  |  |
| Attitude: Reasons not to perform CPR – Fear of infections |  |  | Girls (n=133)  Not statistically significant:  13 (10%) vs 5 (4%) §  RD: -6% , 95%CI [-13%;0%] ¥  (p=0.12)  Boys (n=180)  Not statistically significant:  20 (11%) vs 9 (5%) §  RD: -6% , 95%CI [-13%;0%] ¥  (p=0.12) |  |  |
| Attitude: Reasons not to perform CPR – Being unable |  |  | Girls (n=133)  Statistically significant:  41 (31%) vs 17 (13%) §  RD: -18% , 95%CI [-28%;-8%]  (p<0.001)  *in favour of first aid training*  Boys (n=180)  Statistically significant:  41 (23%) vs 14 (8%) §  RD: -15% , 95%CI [-22%;-8%]  (p<0.001)  *in favour of first aid training* |  |  |
| Attitude: Reasons not to perform CPR – Not willing to perform mouth-to-mouth |  |  | Girls (n=133)  Statistically significant:  25 (19%) vs 9 (7%) §  RD: -12% , 95%CI [-20%;-4%]  (p<0.01)  *in favour of first aid training*  Boys (n=180)  Not statistically significant:  18 (10%) vs 11 (6%) §  RD: -4% , 95%CI [-8%;0%] ¥  (p=0.20)  Significant gender effect at pre-training test (p<0.05) |  |  |
| Attitude: Reasons not to perform CPR – Fear of law suit |  |  | Girls (n=133)  Not statistically significant:  5 (4%) vs 4 (3%) §  RD: -1% , 95%CI [-6%;+5%] ¥  (p=0.93)  Boys (n=180)  Not statistically significant:  11 (6%) 5 (3%) §  RD: -3% , 95%CI [-8%;2%] ¥  (p=0.07) |  |  |
| - 15-16 years | | | | | |
| Attitude: barriers to CPR | Cross-sectional | | Fear to make a mistake and hurting the patient: 28.2%  Lack of confidence (no proper training): 26.6%  Lack of confidence due to forgetting the technique: 22.2%  Being afraid of legal disputes: 14.3%  Concerns for infection: 6.2% | 1, 383 § £† | Ma, 2015 |
| Attitude: Facilitators CPR |  |  | Believe that CPR could increase survival rate  Morally the right thing to do |  |  |
| - 15-17 years | | | | | |
| Attitude: Barriers to CPR willingness | Cross-sectional | | Fear of incomplete performance: 55%  Poor knowledge: 33%  Fear of hurting a victim: 23%  Fear of disease transmission: 11% | 1, 3316 £† | Omi, 2008 |
| Attitude: Improve CPR willingness – previous CPR training |  |  | Repeated training:  Perform CPR   - Once 31% - Twice 34% - Three times or more 51% |  |  |
| - 15-18 years | | | | | |
| Attitude: Barriers to chest compressions + mouth-to-mouth | Cross-sectional in 2010 | | On a stranger:  Fear of poor knowledge: 67%  Fear of hurting the victim: 14%  Fear of disease transmission: 7%  On a trauma victim (blood on face):  Fear of poor knowledge: 58%  Fear of hurting the victim: 18%  Fear of disease transmission: 27%  On a child:  Fear of poor knowledge: 77%  Fear of hurting the victim: 20%  Fear of disease transmission: 3%  On a relative:  Fear of poor knowledge: 72%  Fear of hurting the victim: 21%  Fear of disease transmission: 6% | 1, 1708 £† | Taniguchi, 2012 |
| - 16-17 years | | | | | |
| Attitude: Facilitators CPR | Cross-sectional | | Saving lives: 56%  Important: 55%  Helpful: 49%  Essential: 45% | 1, 494 £† | Parnell, 2006 |
| Attitude: Barriers to CPR |  |  | Pain: 43% |  |  |

Mean ± SD (unless otherwise indicated), MD: mean difference, RR: risk ratio, OR: odds ratio, RD: risk difference

* calculations done by the reviewer using Revman, R software, or Excel

£ No raw data available and CI cannot be calculated.

¥ Imprecision (large variability of results)

† Imprecision (lack of data)

§ Imprecision (limited sample size or low number of events)

λ data extracted from graph

Quality of evidence

***Experimental studies***

| **Author, Year** | **Lack of allocation concealment** | **Lack of blinding** | **Incomplete accounting of outcome events** | **Selective outcome reporting** | **Other limitations** |
| --- | --- | --- | --- | --- | --- |
| Beskind, 2016 | Randomization: no, a cluster-randomization was performed  Allocation concealment: no, allocation was concealed | No, statistician was blinded, outcomes were recorded on manikin or simple yes/no action checks | Yes, larger loss to follow-up in brief video compared to others | No | / |
| Bohn, 2012 | Randomization: yes, no randomization  Allocation concealment: not applicable | No, outcomes were recorded on manikin or knowledge questionnaire | Yes, unequal losses to follow-up. No ITT performed | No | Within subject design as no relevant data from control group could be extracted |
| Choi, 2015 | Randomization: yes, no randomization  Allocation concealment: not applicable | No | No | No | No pre-test/baseline assessment + no inclusion of no training control group |
| Connolly, 2007 | Randomization: yes, no randomization  Allocation concealment: not applicable | No | No (loss to follow-up similarly in both groups) | No | Within subject design (no control available for extracted data); possible incorrect statistical analysis (data analyzed using Student’s t-test, incorrect for within subject design) |
| Creutzfeldt, 2013 | Randomization: yes, no randomization  Allocation concealment: not applicable | No | No | Yes, issue with n (%) from the feelings/perceptions scores | No passive control group  Pre and post self-confidence data were measured through one post-intervention exit questionnaire (not independent) |
| Frederick, 2002 | Randomization: yes, no randomization  Allocation concealment: not applicable | Yes, assessors were un-blinded | No | No | No pre-test/baseline assessment |
| Iserbyt, 2016 | Randomization: yes, no randomization  Allocation concealment: not applicable | No, assessment with questionnaire | No | No | No inclusion of control population |
| Kelley, 2006 | Randomization: yes, no randomization  Allocation concealment: not applicable | No, questionnaire used for knowledge and attitude and skills checklist | No or Yes, 9 additional students only did retentions tests, but no tests immediately after training | No | No inclusion of control population, no pre-test/baseline assessment for skills and several students had already received some type of CPR training |
| Moore, 1992 | Randomization: yes, no randomization  Allocation concealment: not applicable | Unclear if those recording and analyzing outcomes were aware of the arm to which participants were allocated | Unclear, not specified in the article | No | No pre-test/baseline assessment |
| Plotnikoff, 1989 | Randomization: yes, no randomization  Allocation concealment: not applicable | Unclear if those recording and analyzing outcomes were aware of the arm to which participants were allocated | No | No | / |
| Rahman, 2013 | Randomization: no, cluster randomization performed  Allocation concealment: no, sealed envelopes were used | Unclear, questionnaire used, no info on blinding of data analyst | No | No | Yes, control population not appropriately selected (pretest scores for willingness to perform CPR are significantly different between control and intervention group). |
| Reder, 2006 | Randomization: no, cluster randomization performed  Allocation concealment: unclear | No | No (loss to follow-up similarly in all groups) | No | No pre-test/baseline assessment |
| Ribeiro, 2013 | Randomization: yes, no randomization  Allocation concealment: not applicable | No, a questionnaire was used (qualitative data not extracted) | No | No | No inclusion of control population |
| Rivera-Tovar, 1990, USA | Randomization: no  Allocation concealment: unclear | Unclear | No | No | / |
| Vetter, 2016 | Randomization: yes, no randomization  Allocation concealment: not applicable | Unclear | No | No | No inclusion of passive control population |
| Younas, 2006 | Randomization: yes, no randomization  Allocation concealment: not applicable | Unclear if those recording and analyzing outcomes were aware of the arm to which participants were allocated | No | No | No pre-test/baseline assessment |

***Observational studies***

| **Author, Year** | **Inappropriate eligibility criteria** | **Inappropriate methods for exposure and outcome variables** | **Not controlled for confounding** | **Incomplete or inadequate follow-up** | **Other limitations** |
| --- | --- | --- | --- | --- | --- |
| Abelairas-Gómez, 2014 | Not applicable | No | Yes, height, BMI and weight were also correlated with performance, but were not corrected for when age was researched | No | Cohort study |
| Alanazi, 2013 | Not applicable | No, a questionnaire was used | Yes | No | Cross-sectional study |
| Fernandes, 2016 | Yes, no inclusion of control population | No | Yes | No | / |
| Hori, 2016 | Not applicable | No, questionnaires were used | Unclear | No | Cohort study |
| Jiménez-Fábrega, 2009 | Yes, no inclusion of control population | No (not applicable for exposure variables; no flawed measurement of outcome variables) | Yes (only univariate analysis one year after intervention, unclear which covariates in multivariate model immediately after) | Yes, considerable drop-out | / |
| Jones, 2007 | Not applicable | No (not applicable for exposure variables; no flawed measurement of outcome variables) | No (corrected for age, height, weight) | No | Cohort study |
| Kitamura, 2016 | Yes, no inclusion of control population | No, a questionnaire was used | Unclear, corrected for age group, elementary school year, sex, previous CPR training | No | / |
| Lester, 1996 | Not applicable | No (not applicable for exposure variables; no flawed measurement of outcome variables) | Yes | No | Case series |
| Lester, 1997 | Yes, no inclusion of passive control population | No (not applicable for exposure variables; no flawed measurement of outcome variables) | Yes | No | No pre-test/baseline assessment |
| Lorem, 2008 | No | No (not applicable for exposure variables; no flawed measurement of outcome variables) | Yes | No | No pre-test/baseline assessment |
| Ma, 2015 | Not applicable | No, a questionnaire was used | Yes | No | Cross-sectional study |
| Meissner, 2012 | Yes, no inclusion of passive control population | No, a questionnaire and checklist were used | No, controlled for age, height, weight, BMI and gender | Yes, considerable drop-out | Yes, questionable number of participants: 72 vs 59 (girls vs. boys) is more than total of 103 tested post intervention |
| Naqvi, 2011 | Yes, no inclusion of control population | Yes, no pre-test assessment of skills | Yes (no control for covariates) | Unclear (no drop-out for 1^st^ post-test, unclear for 2^nd^ post-test) | / |
| Omi, 2008 | Not applicable | No (not applicable for exposure variables; no flawed measurement of outcome variables) | Yes | No | Cross-sectional study |
| Parashar, 2010 | Yes, no inclusion of control population | No (not applicable for exposure variables; no flawed measurement of outcome variables) | Yes (no controlling for covariates) | Yes (drop-out rate unclear, only short-term (8 days) follow-up) | / |
| Parnell, 2006 | Not applicable | No (not applicable for exposure variables; no flawed measurement of outcome variables) | Yes | No | Cross-sectional study; not all aspects of first aid training were investigated (type of course, age at training) |
| Petric, 2013 | Not applicable | No, a questionnaire was used | Yes, not controlled for confounding factors such as experience with cardiac arrest or social and cultural differences | No | Cohort study, sampling was not randomized |
| Sherif, 2005 | Not applicable | No (not applicable for exposure variables; no flawed measurement of outcome variables) | Yes (only sub-analyses for gender, no controlling for age,…) | No | Cohort study |
| Taniguchi, 2012 | Not applicable | No, a questionnaire was used | Yes | No | Cross-sectional study |
| Toner, 2007 | Yes, no inclusion of control population | No (not applicable for exposure variables; no flawed measurement of outcome variables) | Yes (only univariate analyses with covariates gender, family history of cardiac disease and previous BLS training) | No | / |
| Uray, 2003 | Yes, no inclusion of control population | No (not applicable for exposure variables; no flawed measurement of outcome variables) | Yes | No | Incorrect statistical analysis likely (2x2 table next to graph from which we extracted MD values adds up to 92 persons although a 47 person within subject design was used) |
| Wafik, 2014 | Yes, no inclusion of control population | No (not applicable for exposure variables; no flawed measurement of outcome variables) | Yes | No | / |
| Wilks, 2016 | Yes, no inclusion of control population | No, a questionnaire was used | Unclear, statistics not reported | No | / |

**Certainty of the body of evidence**

KNOWLEDGE

|  | **Initial grading Low [C]** | Downgrading due to |
| --- | --- | --- |
| **Limitations of study design** | -1 | See table ‘Quality of evidence’ |
| **Imprecision** | -1 | Limited sample sizes/low number of events + lack of data |
| **Inconsistency** | 0 |  |
| **Indirectness** | 0 |  |
| **Publication bias** | 0 |  |
| **QUALITY (GRADE)** | **Final grading Very low [D]** |  |

SKILLS

|  | **Initial grading Low [C]** | Downgrading due to |
| --- | --- | --- |
| **Limitations of study design** | -1 | See table ‘Quality of evidence’ |
| **Imprecision** | -1 | Limited sample sizes/low number of events + lack of data |
| **Inconsistency** | 0 |  |
| **Indirectness** | -1 | Measured at 1 time-point (Kelley, 2006) |
| **Publication bias** | 0 |  |
| **QUALITY (GRADE)** | **Final grading Very low [D]** |  |

ATTITUDE

|  | **Initial grading Low [C]** | Downgrading due to |
| --- | --- | --- |
| **Limitations of study design** | -1 | See table ‘Quality of evidence’ |
| **Imprecision** | -1 | Limited sample sizes/low number of events + lack of data + large variability of results |
| **Inconsistency** | 0 |  |
| **Indirectness** | -1 | Measured at 1 time-point |
| **Publication bias** | 0 |  |
| **QUALITY (GRADE)** | **Final grading Very low [D]** |  |

| **Conclusion** | *KNOWLEDGE*  There is limited evidence neither in favour of FA training nor the control. A statistically significant increase in resuscitation (CPR) knowledge using FA training could not be demonstrated in   - 11-12 year olds: compared to no intervention (Moore, 1992)   There is limited evidence in favour of FA training. It was shown that FA training resulted in a statistically significant increase in resuscitation (CPR) knowledge in   - 6-7 year olds: pre to post (Uray, 2003) - 10 year olds: pre to post (Bohn, 2012) - 10-12 year olds: pre to post (Toner, 2007; Connolly, 2007) - 11-12 year olds: compared to no intervention (Plotnikoff, 1989), pre to 1w post, and pre to 8w follow-up (Wilks, 2016) - 11-15 year olds: pre to post (Naqvi, 2011; Wafik, 2014), and pre to 2m follow-up (Wafik, 2014) - 13 year olds: pre to post (Bohn, 2012) - 13-15 year olds: pre to post (Ribeiro, 2013) - 14-16 year olds: pre to post (Jimnénez-Fabrega, 2009; Parashar, 2010) - 14-17 year olds: compared to no intervention (Rahman, 2013) - 14-18 year olds: pre to post (Vetter, 2016) - 16-17 year olds: pre to post, and pre to 6m follow-up (Fernandes, 2016)   Note: resuscitation (CPR) knowledge was shown to decline over time in   - 14-18 year olds: following intervention 1 – post to 12m follow-up (Vetter, 2016)   Note: resuscitation (CPR) knowledge was shown not to decline over time in   - 11-12 year olds: 1w post to 8w post (Wilks, 2016) - 11-15 year olds: post to 3m follow-up (Naqvi, 2011) - 14-18 year olds: intervention 2 – post to 12m follow-up (Vetter, 2016)   Evidence is of very low quality and results of these studies are imprecise due to limited sample sizes, low number of events and lack of data.  *SKILLS*  ***Overall resuscitation (CPR) skills***  There is limited evidence in favour of FA training. It was shown that FA training resulted in a statistically significant increase in overall resuscitation (CPR) skills in   - 10-11 year olds: compared to no intervention (Frederick, 2002) - 11-12 year olds: compared to no intervention (Rivera-Tovar, 1990) - 12 year olds: compared to no intervention (Lorem, 2008) - 13-16 year olds: compared to no intervention (Younas, 2006) - 14-15 year olds: pre to post (Meissner, 2012) - 14-16 year olds: pre to post (Jimnénez-Fabrega, 2009; Parashar, 2010) - 14-18 year olds: pre to post (Vetter, 2016)   The majority of participants demonstrated correct resuscitation (CPR) skills in   - 12-14 year olds: at post and 4w follow-up - no p-value reported (Kelley, 2006)   Note: overall resuscitation (CPR) skills were shown to decline over time in   - 14-16 year olds: post to 1 year follow-up (Jiménez-Fabrega, 2009) - 14-18 year olds: intervention 2 – post to 1 year follow-up (Vetter, 2016)   Note: overall resuscitation (CPR) skills were shown not to decline over time in   - 14-15 year olds: post to 4m follow-up (Meissner, 2012) - 14-18 year olds: intervention 1 – post to 1 year follow-up (Vetter, 2016)   Evidence is of very low quality and results of these studies are imprecise due to limited sample sizes, low number of events and lack of data.  ***Resuscitation (CPR) skills – chest compressions***  There is limited evidence neither in favour of FA training nor the control. A statistically significant increase in chest compression skills could not be demonstrated in   - 11-12 year olds: compared to no intervention – hand position (Plotnikoff, 1989) - 14-18 year olds: video compared to no intervention – hands-off time (Beskind, 2016)   There is limited evidence in favour of FA training. It was shown that FA training resulted in a statistically significant increase in chest compression skills in   - 10 year olds: pre to post (Bohn, 2012) - 10-11 year olds: compared to no intervention (Frederick, 2002) - 11-12 year olds: compared to no intervention – effective compressions (Plotnikoff, 1989) - 12 year olds: compared to no intervention (Lorem, 2008) - 13 year olds: pre to post (Bohn, 2012) - 14-15 year olds: pre to post (Meissner, 2012) - 14-18 year olds: compared to no intervention (Reder, 2005); video compared to no intervention – start compression within 2 min, compression depth (only after 2 months) and compression frequency (Beskind, 2016); classroom compared to no intervention – start compression within 2 min, compression frequency (only post), compression depth (only post) and hands-off time (only after 2 months) (Beskind, 2016); and pre to post (Vetter, 2016)   Note: chest compression skills were shown not to decline over time in   - 14-15 year olds: post to 4m follow-up (Meissner, 2012)   There is limited evidence neither in favour of older age nor younger age. A statistically significant increase in chest compression skills could not be demonstrated with increasing age in   - 9-14 year olds - compression rate, and correct hand placement (Jones, 2007)   There is limited evidence in favour of older age. It was shown that older age resulted in a statistically significant increase in chest compression skills in   - 9-14 year olds - compression depth (Jones, 2007) - 10-15 year olds – all outcomes (Abelairas-Gómez, 2014)   There is limited evidence neither in favour of boys nor girls. A statistically significant difference in chest compression skills could not be demonstrated according to gender in   - 14-15 year olds – compression frequency, and time until start of compression (Meissner, 2012)   There is limited evidence in favour of boys as compared to girls. It was shown that male gender resulted in a statistically significant increase in chest compression skills in   - 14-15 year olds – compression depth (Meissner, 2012)   Evidence is of very low quality and results of these studies are imprecise due to limited sample sizes, low number of events and lack of data.  ***Resuscitation (CPR) skills – ventilations***  There is limited evidence neither in favour of FA training nor the control. A statistically significant increase in ventilation skills could not be demonstrated in   - 11-12 year olds: compared to no intervention – start ventilation (Moore, 1992) - 13-16 year olds: compared to no intervention (Younas, 2006) - 14-18 year olds: intervention 1 compared to no intervention - % correct ventilation (Reder, 2006)   There is limited evidence in favour of FA training. It was shown that FA training resulted in a statistically significant increase in ventilation skills in   - 10 year olds: pre to post (Bohn, 2012) - 10-11 year olds: compared to no intervention (Frederick, 2002) - 11-12 year olds: compared to no intervention – time to ventilation (Moore, 1992); compared to no intervention (Plotnikoff, 1989) - 12 year olds: compared to no intervention (Lorem, 2008) - 13 year olds: pre to post (Bohn, 2012) - 14-15 year olds: pre to post (Meissner, 2012) - 14-18 year olds: intervention 1 compared to no intervention – chest rise; intervention 2 compared to no intervention – chest rise and % correct ventilations; intervention 3 compared to no intervention – chest rise and % correct ventilations (Reder, 2006)   Note: ventilation skills were shown not to decline over time in   - 14-15 year olds: post to 4m follow-up (Meissner, 2012)   There is limited evidence neither in favour of older age nor younger age. A statistically significant increase in ventilation skills could not be demonstrated with increasing age in   - 10-14 year olds – time until ventilation, 1-5^th^ ventilation (Sherif, 2005)   There is limited evidence in favour of older age. It was shown that older age resulted in a statistically significant increase in ventilation skills in   - 10-14 year olds – tidal volume (Sherif, 2005)   Evidence is of very low quality and results of these studies are imprecise due to limited sample sizes, low number of events, lack of data and large variability of results.  *ATTITUDES*  ***Willingness to perform resuscitation (CPR)***  The majority of participants is willing to perform CPR   - 15-16 year olds (Choi, 2015; Ma, 2015)   The majority of participants is not willing to perform CPR   - 11-12 year olds (Lester, 1997) – with girls being more willing than boys - 15-17 year olds (Omi, 2008)   There is limited evidence in favour of FA training. It was shown that FA training resulted in a statistically significant increase in willingness to perform CPR in   - 14-17 year olds (Rahman, 2013) - 15-17 year olds (Omi, 2008)   CPR on a FAMILY MEMBER  The majority of participants is willing to perform hands-on CPR on a family member in   - 11-12 year olds (Lester, 1996) - 12-14 year olds (Kelley, 2006) - 12-18 year olds (Iserbyt, 2016) - 14-18 year olds (Vetter, 2016) - 15-16 year olds (Ma, 2015) - 15-17 year olds (Omi, 2008) - 15-18 year olds (Taniguchi, 2012) - 16-17 year olds (Parnell, 2006)   The majority of participants is willing to perform mouth-to-mouth on a family member in   - 11-12 year olds (Lester, 1996) - 12-14 year olds (Kelley, 2006) - 12-18 year olds (Iserbyt, 2016) - 14-18 year olds (Alanazi, 2013; Vetter, 2016) - 15-16 year olds (Ma, 2015) - 16-17 year olds (Parnell, 2006)   The majority of participants is not willing to perform mouth-to-mouth on a family member in   - 15-17 year olds (Omi, 2008) - 15-18 year olds (Taniguchi, 2012)   CPR on a STRANGER  The majority of participants is willing to perform hands-on CPR on a stranger in   - 12-14 year olds (Kelley, 2006) - 15-16 year olds (Ma, 2015) - 15-17 year olds (Omi, 2008) - 15-18 year olds (Taniguchi, 2012) - 16-17 year olds (Parnell, 2006)   The majority of participants is willing to perform mouth-to-mouth on a stranger in   - 12-14 year olds (Kelley, 2006) - 15-16 year olds (Ma, 2015) - 16-17 year olds (Parnell, 2006)   The majority of participants is not willing to perform mouth-to-mouth on a stranger in   - 12-18 year olds (Iserbyt, 2016) - 14-18 year olds (Alanazi, 2013) - 15-17 year olds (Omi, 2008) - 15-18 year olds (Taniguchi, 2012)   CPR on a CHILD  The majority of participants is willing to perform hands-on CPR only on a child in   - 12-18 year olds (Iserbyt, 2016) - 14-18 year olds (Vetter, 2016) - 15-17 year olds (Omi, 2008) - 15-18 year olds (Taniguchi, 2012)   The majority of participants is willing to perform mouth-to-mouth on a child in   - 12-18 year olds (Iserbyt, 2016) - 14-18 year olds (Vetter, 2016)   The majority of participants is not willing to perform mouth-to-mouth on a child in   - 11-12 year olds (Lester, 1996) - 15-17 year olds (Omi, 2008) - 15-18 year olds (Taniguchi, 2012)   CPR on an OLDER PERSON  The majority of participants is willing to perform hands-on CPR only on an older person in   - 14-18 year olds (Vetter, 2016) - 15-17 year olds (Omi, 2008)   The majority of participants is not willing to perform mouth-to-mouth on an older person in   - 11-12 year olds (Lester, 1996) - 14-18 year olds (Vetter, 2016) - 15-17 year olds (Omi, 2008)   CPR on a TRAUMA PATIENT  The majority of participants is willing to perform hands-on CPR only on a trauma patient in   - 15-17 year olds (Omi, 2008) - 15-18 year olds (Taniguchi, 2012)   The majority of participants is not willing to perform hands-on CPR only on a trauma patient in   - 14-18 year olds (Vetter, 2016)   The majority of participants is not willing to perform mouth-to-mouth on a trauma patient in   - 14-18 year olds (Vetter, 2016) - 15-17 year olds (Omi, 2008) - 15-18 year olds (Taniguchi, 2012)   ***Confidence to perform resuscitation (CPR)***  The majority of participants is confident to perform CPR following FA training in   - 10-11 year olds (Hori, 2016) - 10-12 year olds (Kitamura, 2016) - 10-13 year olds (Bohn, 2012) - 14-15 year olds (Meissner, 2012; Creutzfeldt, 2013; Ma, 2015)   The majority of participants is not confident to perform CPR following FA training in   - 12-13 year olds (Hori, 2016) - 15-16 year olds (Hori, 2016)   ***Barriers and facilitators to resuscitation (CPR)***  Barriers to resuscitation (CPR) include   - Fear of infection   - 11-12 year olds (Lester, 1996; Lester, 1997)   - 12-14 year olds (Kelley, 2006)   - 12-15 year olds (Petric, 2013)   - 12-18 year olds (Iserbyt, 2016)   - 15-16 year olds (Ma, 2015)   - 15-17 year olds (Omi, 2008)   - 15-18 year olds (Taniguchi, 2012) - Fear to harm the patient   - 12-14 year olds (Kelley, 2006)   - 12-15 year olds (Petric, 2013)   - 12-18 year olds (Iserbyt, 2016)   - 15-16 year olds (Ma, 2015)   - 15-17 year olds (Omi, 2008)   - 15-18 year olds (Taniguchi, 2012) - Lack of knowledge   - 12-15 year olds (Petric, 2013)   - 12-18 year olds (Iserbyt, 2016)   - 15-16 year olds (Ma, 2015)   - 15-17 year olds (Omi, 2008)   - 15-18 year olds (Taniguchi, 2012) - Fear to get sued   - 12-14 year olds (Kelley, 2006)   - 12-18 year olds (Iserbyt, 2016)   - 15-16 year olds (Ma, 2015) - Serious injuries / unpleasant situations (vomit, blood, dirt, unpleasant smell)   - 11-12 year olds (Lester, 1996; Lester, 1997) - Safety concerns   - 11-12 year olds (Lester, 1997) - Public place   - 11-12 year olds (Lester, 1997)   Facilitators to resuscitation (CPR) include   - Believe that CPR could increase survival rate   - 15-16 year olds (Ma, 2015)   - 16-17 year olds (Parnell, 2006) - Believe that CPR is the morally right thing to do   - 15-16 year olds (Ma, 2015) - Believe that CPR is important, helpful, and essential   - 16-17 year olds (Parnell, 2006)   Evidence is of very low quality and results of these studies are imprecise due to limited sample sizes, low number of events, lack of data and large variability of results. |
| --- | --- |
| **Reference(s)** | **Articles**  Abelairas-Gomez C, Rodriguez-Nunez A, Casillas-Cabana M, Romo-Pérez V, Barcala-Furelos R. *Schoolchildren as life savers: At what age do they become strong enough?* Resuscitation 2014, 85: 814-819.  Alanazi A, Bin H, Alqahtani M, Alhalyabah H, Alanazi A, Al O, Saleh. *Community awareness about cardiopulmonary resuscitation among secondary school students in Riyadh*. World Journal of Medical Sciences 2013, 8**,** 186-189.  Beskind DL, Stolz U, Thiede R, Hoyer R, Burns W, Brown J, Ludgate M, Tiutan T, Shane R, McMorrow D, Pleasants M, Panchal AR. *Viewing a bried chest-compression-only CPR video improves bystander CPR performance and responsiveness in high school students: a cluster randomized trial.* Resuscitation 2016, 104: 28-33.  Bohn A, Van Aken HK, Möllhoff T, Wienzek H, Kimmeyer P, Wild E, Döpker S, Lukas RP, Weber TP. *Teaching resuscitation in schools: annual tuition by trained teachers is effective starting at age 10. A four-year prospective cohort study.* Resuscitation 2012, 83: 619-625.  Choi HS, Lee DH, Kim CW, Kim SE, Oh JH. *Peer-assisted learning to train high-school students to perform basic life-support.* World J Emerg Med 2015, 6(3): 186-190.  Connolly M, Toner P, Connolly D, McCluskey DR. *The ‘ABC for life’ programme – Teaching basic life support in schools.* Resuscitation 2007, 72: 270-279.  Creutzfeldt J, Hedman L, Heinrichs L, Youngblood P, Felländer-Tsai L. *Cardiopulmonary resuscitation training in high school using avatars in virtual worlds: an international feasibility study.* Journal of medical Internet research 2013, 15**,** e9.  Fernandes JMG, Leite ALDS, Auto BDSD, de Lima JEG, Rivera IR, Mendonça MA. *Teaching basic life support to students of public and private high schools.* Arq Bras Cardiol 2014, 102(6): 593-601  Frederick K, Bixby E, Orzel MN, Stewart-Brown S, Willet K. *Will changing the emphasis from ‘pulseless’ to ‘no signs of circulation’ improve the recall scores for effective life support skills in children?* Resuscitation 2002, 55: 255-261.  Hori S, Suzuki M, Yamazaki M, Aikawa N, Yamazaki H. *Cardiopulmonary resuscitation training in schools: A comparison of trainee satisfaction among different age groups.* Keio Journal of Medicine 2016, 65**,** 49-56.  Iserbyt P. *The effect of basic life support education on secondary school students’ willingness to and reasons not to perform BLS in real life.* Acta Cardiol 2016, 71(0).  Jiménez-Fábrega X, Escalada-Roig X, Sánchez M, Culla A, Diaz N, Gómez X, Villena O, Rodriguez E, Gaspar A, Molina JE, Salvador J, Miro O. *Results achieved by emergency physicians in teaching basic cardiopulmonary resuscitation to secondary school students.* European Journal of Emergency Medicine 2009, 16(3): 139-144.  Jones I, Whitfield R, Colquhoun M, Chamberlain D, Vetter N, Newcombe R. *At what age can schoolchildren provide effective chest compressions? An observational study from the Heartstart UK schools training programme.* BMJ 2007.  Kelley J, Richman PB, Ewy GA, Clark L, Bulloch B, Bobrow BJ. *Eighth grade students become proficient at CPR and use of an AED following a condensed training programme.* Resuscitation 2006, 71(2):229-36.  Kitamura T, Nishiyama C, Murakami Y, Yonezawa T, Nakai S, Hamanishi M, Marukawa S, Sakamoto T, Iwami T. *Compression-only CPR training in elementary schools and student attitude toward CPR*. Pediatr Int 2016, 58**,** 698-704.  Lester C, Donnelly P, Weston C, Morgan M. *Teaching schoolchildren cardiomulmonary resuscitation.* Resuscitation 1996, 31: 33-38.  Lester C, Donnelly P, Weston C. *Is peer tutoring beneficial in the context of school resuscitation training?* Health edication research theory & practice 1997, 12(3): 347-354.  Lorem T, Palm A, Wik L. *Impact of a self-instruction CPR kit on 7^th^ graders’ and adults’ skills and CPR performance.* Resuscitation 2008, 79: 103-108.  Lukas RP, Van Aken H, MölhoffT, Weber T, Rammert M, Wild E, Bohn A. *Kids save lives: a six-year longitudinal study of schoolchildren learning cardiopulmonary resuscitation: Who should do the teaching and will the effects last?* Resuscitation 2016, 101: 35-40.  Ma AWW, Vyas L, Wong KL, Tou AYL, Wilks J. *CPR knowledge and attitudes among high school students aged 15-16 in Hong Kong.* Hong Kong Journal of Emergency Medicine 2015, 22**,** 3-13.  Meissner TM, Kloppe C, Hanefeld C. *Basic life support skills of high school students before and after cardiopulmonary resuscitation training: a longitudinal investigation.* Scand J Trauma Resusc Emerg Med 2012, 20**,** 31.  Moore JP, Plotnikoff RC, Preston GD. *A study of school students’ long term retention of expired air resuscitation knowledge and skills.* Resuscitation 1992, 24: 17-25.  Naqvi S, Siddiqi R, Hussain SA, Batool H, Arshad H. *School children training for basic life support.* Journal of the College of Physicians and surgeons Pakistan 2011, 21(10): 611-615.  Omi W, Taniguchi T, Kaburaki T, Okajima M, Takamura M, Noda T, Ohta K, Itoh H, Goto Y, Kaneko S, Inaba H. *The attitudes of Japanese high school students toward cardiopulmonary resuscitation.* Resuscitation 2008, 78: 340-345.  Parashar AK. *Effective planned teaching programme on knowledge & practice of basic life support among students in Mangalore.* Nursing Journal of India 2010, 101(2): 40-43.  Parnell MM, Pearson J, Galletly DC, Larsen PD. *Knowledge of and attitudes towards resuscitation in New Zealand high-school students.* Emerg Med J 2006, 23: 899-902.  Petric J, Malicki M, Markovic D, Mestrovic J. *Students’ and parents’ attitudes toward basic life suppot training in primary schools.* Croat Med J 2013, 54: 376-380.  Plotnikoff R, Moore PJ. *Retention of cardiopulmonary resuscitation knowledge and skills by 11- and 12-year-old children.* The medical Journal of Asutralia 1989, 150: 297-302.  Rahman NH, Keng Sheng C, Kamauzaman TH, Md Noh AY, Wahab SF, Zaini IZ, Fauzi MH, Ab Rahman A, Dzulkifli NS. *A multicenter controlled trial on knowledge and attitude about cardiopulmonary resuscitation among secondary school children in Malaysia.* Int J Emerg Med 2013, 6**,** 37.  Reder S, Cummings P, Quan L. *Comparison of the three instructional methods for teaching cardiopulmonary resuscitation and use of an automatic external defibrillator to high school students.* Resuscitation 2006, 69: 443-453.  Ribeiro LG, Germano R, Menezes PL, Schmidt A, Pazin-Filho A. *Medical students teaching cardiopulmonary resuscitation to middle school Brazilian students.* Arq Bras Cardiol 2013, 101(4): 328-335.  Rivera-Tovar LA, Jones RT. *Effect of elaboration on the acquisition and maintenance of cardiopulmonary resuscitation.* J Pediatr Psychol 1990, 15(1):123-38.  Sherif C, Erdös J, Sohm M, Schönbauer R, Rabitsch W, Schuster E, Frass M. *Effectiveness of mouth-to-mouth resuscitation performed by young adolescents on a mannequin.* American Journal of Emergency Medicine 2005, 23: 51-54.  Taniguchi T, Sato K, Fujita T, Okajima M, Takamura M. *Attitudes to bystander cardiopulmonary resuscitation in Japan in 2010.* Circ J 2012, 76**,** 1130-5.  Toner P, Connolly M, Laverty L, McGrath P, Connolly D, McCluskey DR. *Teaching basic life support to school children using medical students and teachers in a ‘peer-training’ model – results of the ‘ABC for life’ programme.* Resuscitation 2007, 75: 169-175.  Uray T, Lunzer A, Ochsenhofer A, Thanikkel L, Zingerle R, Lillie P, Brandl E, Sterz F, LSFA school study group. *Feasibility of life-supporting first-aid (LSFA) training as a mandatory subject in primary schools.* Resuscitation 2003, 59: 211-220.  Vetter VL, Haley DM, Dugan NP, Iyer VR, Shults J. *Innovative cardiopulmonary resuscitation and automated external defibrillator programs in schools: Results from the Student Program for Olympic Resuscitation Training in Schools (SPORTS) study.* Resuscitation 2016, 104: 46-52.  Wafik W, Tork H. *Effectiveness of a first-aid intervention program applied by undergraduate nursing students to preparatory school children.* Nursing and Health Sciences 2014, 16: 112-118.  Wilks J, Kanasa H, Pendergast D, Clark K. *Emergency response readiness for primary school children.* Aust Health Rev 2016.  Younas S, Raynes A, Morton S, Mackway-Jones K. *An evaluation of the effectiveness of the opportunities for resuscitation and citizen safety (ORCS) defibrillator training programme designed for older school children.* Resuscitation 2006, 71: 222-228.  **Systematic reviews**  De Buck E, Van Remoortel H, Dieltjens T, Verstraeten H, Clarysse M, Moens O, Vandekerckhove P. *Evidence-based educational pathway for the integration of first aid training in school curricula.* Resuscitation 2015, 94**,** 8-22.  Dieltjens T, De Buck E, Verstraeten H, Adriaenssens L, Clarysse M, Moens O, Devreker A, Bastiaen M, Claessens C, Verhelst K. *Evidence-based recommendations on automated external defibrillator training for children and young people in Flanders-Belgium.* Resuscitation 2013, 84**,** 1304-9.  He Z, Wynn P, Kendrick D. *Non-resuscitative first-aid training for children and laypeople: a systematic review.* Emerg Med J 2014, 31(9):763-8.  Lenson S, Mills J. *First aid knowledge retention in school children: A review of the literature.* Australasian Journal of Paramedicine 2016, 13.  Plant N, Taylor K. *How best to teach CPR to schoolchildren: a systematic review.* Resuscitation 2013, 84(4):415-21.  Reveruzzi B, Buckley L, Sheehan M. *School-Based First Aid Training Programs: A Systematic Review.* J Sch Health 2016, 86(4):266-72. |

## Choking

Characteristics of included studies

| **Author, year, Country** | **Study design** | **Population** | **Comparison/Risk factor** | **Remarks** |
| --- | --- | --- | --- | --- |
| Akca, 2016, Turkey | Observational: before after study | *Nr. of participants:*  100 students:   - Gender not reported   *Age range:*  Not reported  Mean ± SD:  17.1 ± 0.73 years | **Intervention:**  *Program:* Foreign body aspiration (ear, nose and respiratory tract) training  *Content:*  Frequency, causes, symptoms and first aid of foreign body aspiration.  Lecture + demonstration + hands-on training of first aid interventions.  *Duration:* 45 min | Outcomes were measured before and 1 month after training.  *Knowledge*: Self-developed questionnaire on foreign body aspiration knowledge |
| Celik, 2013, Turkey | Observational: before after study | *Nr. of participants:*  231 students:   - 170 high school - 61 university - Gender not reported   [Only data from high school students were extracted]  *Age range:*  15-18 years (n=170)  Grade 10-12 | **Intervention:**  *Program:* Foreign body aspiration (ear, nose and airway) training  *Content:* Frequency, causes, symptoms and first aid of foreign body aspiration.  Lecture (power-point) + 3 videos + hands-on dummy training for at least 5 students of each group while others watched.  *Duration:* 60 minute lecture + 5 minute video + 45 minute practice | Outcomes were measured before and 1 month after training.  *Knowledge*: Questionnaire on foreign body aspiration knowledge  *Attitude*: Self-efficacy |
| Connolly, 2007, UK | Experimental: controlled before after study  [extracted data are from experimental before after design without control] | *Nr. of participants:* 79 students:   - 47 males - 32 females   Two arms:  Intervention n=46  Control n=33  *Age range:*  10-12 years  Mean intervention:  11.8 years  Mean control:  12.7 years  (SD not reported) | **Intervention:**  *Program*: CPR training (‘ABC for life’ program)  *Content*: How to approach a patient, CPR and recovery position.  Video + small group lecture + demonstration + hands-on manikin training.  *Duration*: < 2 hours  **Control:**  No intervention. | Outcomes were measured before, immediately after (only intervention group) and 6 months after training.  *Knowledge:* 22 point multiple choice questionnaire (patient approach, basic life support and CPR) |
| Frederick,  2000, UK | Experimental: controlled before after study | *Nr. of participants:* 1096 students   - Gender not reported   Two arms:  Intervention n=542  Control n=554  *Age range:*  10-11 years | **Intervention:**  *Program:* Injury Minimization Program for Schools (IMPS)  *Content:* Road safety, accidents in the home, fire, electricity, poisons, waterways, basic life support and resuscitation skills.  Lecture + video + hands-on manikin training + hospital/emergency department visit.  *Duration:* Not reported. Spread over 1 academic year  **Control:**  No intervention. | Outcomes were measured before and 5 months after training.  *Knowledge:* Self-developed quiz  *Skills:* Performance on a basic life support scenario. [only measured post intervention]  *Attitude:* Record observations of dangerous behavior in a video  [attitude data were not extracted]  [only post-training data were extracted] |
| Wafik,  2014, Egypt | Observational: before after study | *Nr. of participants:* 100 students:   - Gender not reported     *Age range:*  11-16 years  Mean ± SD:  13.2 ± 0.8 years | **Intervention:**  *Program:* First aid training  *Content:* wounds, poisoning, chemicals,  electrocution, hemorrhage, burns, fractures, choking and basic life support.  Lecture + question time + group discussion.  *Duration:* 6 sessions (1 hour/session) | Outcomes were measured before, immediately after and 2 months after training.  *Knowledge*: 32 item questionnaire.  *Skills*: Performance on five first aid scenarios (choking, burns, poisoning, and fractures). |
| Wilks, 2016, Australia | Observational: before after study | *Nr. of participants*: 107 students:   - 51 males - 56 females   *Age range*:  11-12 years  Mean ± SD:  11.4 ± 0.5 years | **Intervention:**  *Program*: First aid, CPR and beach safety training  *Content*:   - Beach safety - Leadership and team bonding exercises - First aid: sprains, bleeding, choking, heart attack, allergic reactions - Hands-on manikin training   *Duration:* 1 day | Outcomes were measured before, 1 week after, and 8 weeks after training.  *Knowledge:* 50-item quiz on emergency services and life-supporting first aid |

**Synthesis of findings**

| **Choking** | | | | | |
| --- | --- | --- | --- | --- | --- |
| **Outcome** | | **Comparison** | **Effect Size** | **#studies, # participants** | **Reference** |
| *Knowledge* | | | | | |
| - 10-11 years | | | | | |
| Knowledge: Choking FA | FA training vs no intervention | | Statistically significant:  1.6±1.0 vs 1.3±1.0  MD: 0.30, 95%CI [0.18;0.42]  (p<0.00001)*  *in favour of first aid training* | 1, 542 vs 554 §(power analysis) | Frederick, 2000 |
| - 10-12 years | | | | | |
| Knowledge: Clear obstruction | Pre vs post | | Not statistically significant:  38 (82.6%) vs 41 (89.1%) §  RR: 1.08* £†  (p>0.05) | 1, 46 vs 46  (within subject) | Connolly, 2007 |
| - 11-12 years | | | | | |
| Knowledge: Choking FA | Pre vs 1 week post | | Statistically significant:  31 (29%) vs 85 (81%) §  RR: 2.88* £  (p<0.001)  *in favour of first aid training* | 1, 107 vs 102  (within subject) | Wilks, 2016 |
|  | Pre vs 8 week follow-up | | Statistically significant:  31 (29%) vs 81 (77%) §  RR: 2.66* £  (p<0.001)  *in favour of first aid training* | 1, 107 vs 105  (within subject) |  |
| - 11-15 years | | | | | |
| Knowledge: Choking FA | Pre vs post | | Statistically significant:  0 (0%) vs 100 (100%) §  RR: 201.00* £  (p<0.001)  *in favour of first aid training* | 1, 100 vs 100  (within subject) | Wafik, 2014 |
|  | Pre vs 2 month follow-up | | Statistically significant:  0 (0%) vs 100 (100%) §  RR: 201.00* £  (p<0.001)  *in favour of first aid training* |  |  |
| - 16-18 years | | | | | |
| *Knowledge:* FA  object in respiratory track | Pre vs post | | Statistically significant:  8 (8%) vs 36 (36%) §  RR: 4.50* £  (p=0.000)  *in favour of first aid training* | 1, 100 vs 100  (within subject) | Akca, 2016 |
| *Knowledge:* FA partial obstruction of respiratory track |  |  | Statistically significant:  57 (57%) vs 80 (80%) §  RR: 1.40* £  (p=0.000)  *in favour of first aid training* |  |  |
| *Knowledge:* FA complete obstruction of respiratory track of <1 year children |  |  | Statistically significant:  2 (2%) vs 35 (35%) §  RR: 17.50* £  (p=0.000)  *in favour of first aid training* |  |  |
| *Knowledge:* FA complete obstruction of respiratory track of >1 year children |  |  | Statistically significant:  2 (2%) vs 35 (35%) §  RR: 17.50* £  (p=0.000)  *in favour of first aid training* |  |  |
| *Knowledge:* FA obstruction of respiratory track in an unconscious child |  |  | Statistically significant:  0 (0%) vs 16 (16%) §  OR: ∞, 95%CI [3.86, ∞]  (p=0.000)*  *in favour of first aid training* |  |  |
| *Skills* | | | | | |
| - 11-15 years | | | | | |
| Skills: Choking FA | Pre vs post | | Statistically significant:  0 (0%) vs 95 (95%) §  RR: 191.00* £  (p<0.001)  *in favour of first aid training* | 1, 100 vs 100  (within subject) | Wafik, 2014 |
|  | Pre vs 2 month follow-up | | Statistically significant:  0 (0%) vs 67 (67%) §  RR: 135.00* £  (p<0.001)  *in favour of first aid training* |  |  |
| *Attitude* | | | | | |
| - 15-18 years | | | | | |
| *Attitude:* Self-efficacy for choking | Pre vs post | | 17 (10%) vs 82 (48.2%) §  RR: 4.82 £†  [p not reported] | 1, 170 vs 170  (within subject) | Celik, 2013 |

Mean ± SD (unless otherwise indicated), MD: mean difference, RR: risk ratio, OR: odds ratio, RD: risk difference

* calculations done by the reviewer using Revman, R software, or Excel

£ No raw data available and CI cannot be calculated.

¥ Imprecision (large variability of results)

† Imprecision (lack of data)

§ Imprecision (limited sample size or low number of events)

λ data extracted from graph

Quality of evidence

***Experimental studies***

| **Author, Year** | **Lack of allocation concealment** | **Lack of blinding** | **Incomplete accounting of outcome events** | **Selective outcome reporting** | **Other limitations** |
| --- | --- | --- | --- | --- | --- |
| Connolly, 2007 | Randomization: yes, no randomization  Allocation concealment: not applicable | No | No (loss to follow-up similarly in both groups) | No | Within subject design (no control available for extracted data); possible incorrect statistical analysis (data analyzed using Student’s t-test, incorrect for within subject design) |
| Frederick, 2000 | Randomization: yes, no randomization  Allocation concealment: not applicable | Yes, assessors were un-blinded | No | No | / |

***Observational studies***

| **Author, Year** | **Inappropriate eligibility criteria** | **Inappropriate methods for exposure and outcome variables** | **Not controlled for confounding** | **Incomplete or inadequate follow-up** | **Other limitations** |
| --- | --- | --- | --- | --- | --- |
| Akca, 2016 | Yes, no inclusion of control population | No | Yes | No | / |
| Celik, 2013 | Yes, no inclusion of control population | No, a questionnaire was used | Yes, not controlled for confounding factors (i.e. previous history with choking infants/children) | No | / |
| Wafik, 2014 | Yes, no inclusion of control population | No (not applicable for exposure variables; no flawed measurement of outcome variables) | Yes | No | / |
| Wilks, 2016 | Yes, no inclusion of control population | No, a questionnaire was used | Unclear, statistics not reported | No | / |

**Certainty of the body of evidence**

KNOWLEDGE

|  | **Initial grading Low [C]** | Downgrading due to |
| --- | --- | --- |
| **Limitations of study design** | -1 | See table ‘Quality of evidence’ |
| **Imprecision** | -1 | Limited sample sizes/low number of events + lack of data |
| **Inconsistency** | 0 |  |
| **Indirectness** | 0 |  |
| **Publication bias** | 0 |  |
| **QUALITY (GRADE)** | **Final grading Very low [D]** |  |

SKILLS

|  | **Initial grading Low [C]** | Downgrading due to |
| --- | --- | --- |
| **Limitations of study design** | -1 | See table ‘Quality of evidence’ |
| **Imprecision** | -1 | Low number of events + lack of data |
| **Inconsistency** | 0 |  |
| **Indirectness** | 0 |  |
| **Publication bias** | 0 |  |
| **QUALITY (GRADE)** | **Final grading Very low [D]** |  |

ATTITUDE

|  | **Initial grading Low [C]** | Downgrading due to |
| --- | --- | --- |
| **Limitations of study design** | -1 | See table ‘Quality of evidence’ |
| **Imprecision** | -1 | Low number of events + lack of data |
| **Inconsistency** | 0 |  |
| **Indirectness** | 0 |  |
| **Publication bias** | 0 |  |
| **QUALITY (GRADE)** | **Final grading Very low [D]** |  |

| **Conclusion** | *KNOWLEDGE*  There is limited evidence neither in favour of FA training nor the control. A statistically significant increase of choking knowledge using FA training could not be demonstrated in   - 10-12 year olds: pre to post (Connolly, 2007)   There is limited evidence in favour of FA training. It was shown that FA training resulted in a statistically significant increase of choking knowledge in   - 10-11 year olds: compared to no intervention (Frederick, 2000) - 11-12 year olds: pre to post, and pre to 8w follow-up (Wilks, 2016) - 11-15 year olds: pre to post, and pre to 2m follow-up (Wafik, 2014) - 16-18 year olds: pre to post (Akca, 2016)   Evidence is of very low quality and results of these studies are imprecise due to limited sample sizes, low number of events and lack of data.  *SKILLS*  There is limited evidence in favour of FA training. It was shown that FA training resulted in a statistically significant increase of choking skills in   - 11-15 year olds: pre to post, and pre to 2m follow-up (Wafik, 2014)   Evidence is of very low quality and results of this study are imprecise due to low number of events and lack of data.  *ATTITUDE*  It was shown that FA training resulted in a significant increase of choking attitudes in   - 15-18 year olds: pre to post – although an exact p-value was not reported (Celik, 2013)   Evidence is of very low quality and results of this study are imprecise due to low number of events and lack of data. |
| --- | --- |
| **Reference(s)** | **Articles**  Akca SO. *The effect of foreign body aspiration training on the knowledge level of pupils.* Braz J Otorhinolaryngology 2016, 82(4): 408-415.  Çelik N, Arikan D. *The effect of the training given to the child development students about foreign body aspiration upon their knowledge levels.* International Journal of Pediatric Otorhinolaryngology 2013, 77: 1811-1817.  Connolly M, Toner P, Connolly D, McCluskey DR. *The ‘ABC for life’ programme – Teaching basic life support in schools.* Resuscitation 2007, 72: 270-279.  Frederick K, Bixby E, Orzel MN, Stewart-Brown S, Willett K. *An evaluation of the effectiveness of the injury minimization programme for schools (IMPS).* Injury prevention 2000, 6: 92-95.  Wafik W, Tork H. *Effectiveness of a first-aid intervention program applied by undergraduate nursing students to preparatory school children.* Nursing and Health Sciences 2014, 16: 112-118.  Wilks J, Kanasa H, Pendergast D, Clark K. *Emergency response readiness for primary school children.* Aust Health Rev 2016.  **Systematic reviews**  De Buck E, Van Remoortel H, Dieltjens T, Verstraeten H, Clarysse M, Moens O, Vandekerckhove P. *Evidence-based educational pathway for the integration of first aid training in school curricula.* Resuscitation 2015, 94**,** 8-22.  Dieltjens T, De Buck E, Verstraeten H, Adriaenssens L, Clarysse M, Moens O, Devreker A, Bastiaen M, Claessens C, Verhelst K. *Evidence-based recommendations on automated external defibrillator training for children and young people in Flanders-Belgium.* Resuscitation 2013, 84**,** 1304-9.  He Z, Wynn P, Kendrick D. *Non-resuscitative first-aid training for children and laypeople: a systematic review.* Emerg Med J 2014, 31(9):763-8.  Lenson S, Mills J. *First aid knowledge retention in school children: A review of the literature.* Australasian Journal of Paramedicine 2016, 13.  Plant N, Taylor K. *How best to teach CPR to schoolchildren: a systematic review.* Resuscitation 2013, 84(4):415-21.  Reveruzzi B, Buckley L, Sheehan M. *School-Based First Aid Training Programs: A Systematic Review.* J Sch Health 2016, 86(4):266-72. |

## Skin wounds

Characteristics of included studies

| **Author, year, Country** | **Study design** | **Population** | **Comparison/Risk factor** | **Remarks** |
| --- | --- | --- | --- | --- |
| Campbell, 2001, USA | Experimental: randomized controlled trial | *Nr. of participants:* 660 students:   - 51% males - 49% females   Two arms:  Intervention n=293 Control n=367  *Age range:*  11-16 years  Mean ± SD:  13 ± 1.11 years | **Intervention:**  *Program:* First aid and home safety training  *Content:* Emergency care, fever and first aid kit, bleeding, burns, fractures, dislocations and sudden illness, sports injury and prevention, poisoning, bites, stings and allergies, first aid review and household safety.  **Control:**  *Program:* Tobacco and alcohol prevention program  *Content:* Health effects of smoking and alcohol, peer pressure, decision making, societal influences and refusal skills.  *Duration (intervention + control):* 8 sessions (2 hours/session) spread over a 7-to 10-week period | Outcomes were measured before, immediately after and 1 year after training.  *Knowledge:*   - Emergency response procedures (check-call-care) - First aid kit   [only knowledge data for FA procedure were extracted]  *Skills:* Responses to two audio-recorded scenarios: scenario 1. severe wound from glass; scenario 2. severe burn injury from a toddler. Type and order of responses were scored:   - Check the scene and victim - Call 911 - Care for the victim   *Attitude:* First aid confidence |
| Wafik,  2014, Egypt | Observational: before after study | *Nr. of participants:* 100 students:   - Gender not reported     *Age range:*  11-16 years  Mean ± SD:  13.2 ± 0.8 years | **Intervention:**  *Program:* First aid training  *Content:* wounds, poisoning, chemicals,  electrocution, hemorrhage, burns, fractures, choking and basic life support.  Lecture + question time + group discussion.  *Duration:* 6 sessions (1 hour/session) | Outcomes were measured before, immediately after and 2 months after training.  *Knowledge*: 32 item questionnaire.  *Skills*: Performance on five first aid scenarios (choking, burns, poisoning, and fractures). |
| Wilks, 2016, Australia | Observational: before after study | *Nr. of participants*: 107 students:   - 51 males - 56 females   *Age range*:  11-12 years  Mean ± SD:  11.4 ± 0.5 years | **Intervention:**  *Program*: First aid, CPR and beach safety training  *Content*:   - Beach safety - Leadership and team bonding exercises - First aid: sprains, bleeding, choking, heart attack, allergic reactions - Hands-on manikin training   *Duration:* 1 day | Outcomes were measured before, 1 week after, and 8 weeks after training.  *Knowledge:* 50-item quiz on emergency services and life-supporting first aid |

**Synthesis of findings**

| **Skin wound** | | | | | |
| --- | --- | --- | --- | --- | --- |
| **Outcome** | | **Comparison** | **Effect Size** | **#studies, # participants** | **Reference** |
| *Knowledge* | | | | | |
| - 11-12 years | | | | | |
| Knowledge: cuts and bleeding FA | | Pre vs 1 week post | Statistically significant:  74 (69%) vs 94 (90%) §  RR: 1.33* £  (p<0.001- overall p)  *in favour of first aid training* | 1, 107 vs 102  (within subject) | Wilks, 2016 |
|  |  | Pre vs 8 week follow-up | Statistically significant:  74 (69%) vs 93 (82%) §  RR: 1.28* £  (p<0.001- overall p)  *in favour of first aid training* | 1, 107 vs 105  (within subject) |  |
| - 11-15 years | | | | | |
| Knowledge: Wounds FA (score >60%) | Pre vs post | | Statistically significant:  47 (47%) vs 96 (96%) §  RR: 2.04* £  (p<0.001)  *in favour of first aid training* | 1, 100  (within subject) | Wafik, 2014 |
|  | Pre vs 2 month follow-up | | Statistically significant:  47 (47%) vs 90 (90%) §  RR: 1.91* £  (p<0.001)  *in favour of first aid training* |  |  |
| *Skills* | | | | | |
| - 11-16 years | | | | | |
| Skills: Order of FA response | FA training vs sham intervention | | Scenario (severe glass wound)  Statistically significant:  21 (14%) vs 10 (5%) §  RR: 3.16, 95%CI [1.53;6.51]*  (p<0.001)  *in favour of first aid training* | 1, 147 vs 221 | Campbell, 2001 |
| Skills: Correct procedures listed |  |  | Scenario (severe glass wound)  Not statistically significant:  75 (52%) vs 125 (57%) §  RR: 0.90, 95%CI [0.74;1.10]* ¥  (p=0.413) |  |  |
| *Attitude – no studies available* | | | | | |

Mean ± SD (unless otherwise indicated), MD: mean difference, RR: risk ratio, OR: odds ratio, RD: risk difference

* calculations done by the reviewer using Revman, R software, or Excel

£ No raw data available and CI cannot be calculated.

¥ Imprecision (large variability of results)

† Imprecision (lack of data)

§ Imprecision (limited sample size or low number of events)

λ data extracted from graph

Quality of evidence

***Experimental studies***

| **Author, Year** | **Lack of allocation concealment** | **Lack of blinding** | **Incomplete accounting of outcome events** | **Selective outcome reporting** | **Other limitations** |
| --- | --- | --- | --- | --- | --- |
| Campbell, 2001 | Randomization and allocation concealment: unclear, not specified in the article | No | Yes, considerable loss to follow-up | Yes, data from some time-points not reported | No pre-test/baseline assessment |

***Observational studies***

| **Author, Year** | **Inappropriate eligibility criteria** | **Inappropriate methods for exposure and outcome variables** | **Not controlled for confounding** | **Incomplete or inadequate follow-up** | **Other limitations** |
| --- | --- | --- | --- | --- | --- |
| Wafik, 2014 | Yes, no inclusion of control population | No (not applicable for exposure variables; no flawed measurement of outcome variables) | Yes | No | / |
| Wilks, 2016 | Yes, no inclusion of control population | No, a questionnaire was used | Unclear, statistics not reported | No | / |

**Certainty of the body of evidence**

KNOWLEDGE

|  | **Initial grading Low [C]** | Downgrading due to |
| --- | --- | --- |
| **Limitations of study design** | -1 | See table ‘Quality of evidence’ |
| **Imprecision** | -1 | Limited sample sizes/low number of events + lack of data |
| **Inconsistency** | 0 |  |
| **Indirectness** | 0 |  |
| **Publication bias** | 0 |  |
| **QUALITY (GRADE)** | **Final grading Very low [D]** |  |

SKILLS

|  | **Initial grading High [A]** | Downgrading due to |
| --- | --- | --- |
| **Limitations of study design** | -1 | See table ‘Quality of evidence’ |
| **Imprecision** | -1 | Low number of events + large variability of the results |
| **Inconsistency** | -1 | Impact of FA training on skin wound skills is not consistent |
| **Indirectness** | 0 |  |
| **Publication bias** | 0 |  |
| **QUALITY (GRADE)** | **Final grading Very low [D]** |  |

ATTITUDE

There is no evidence available.

| **Conclusion** | *KNOWLEDGE*  There is limited evidence in favour of FA training. It was shown that FA training resulted in a statistically significant increase of skin wound knowledge   - 11-12 year olds: pre to post, and pre to 8w follow-up (Wilks, 2016) - 11-15 year olds: pre to post, and pre to 2m follow-up (Wafik, 2014)   Evidence is of very low quality and results of these studies are imprecise due to limited sample sizes, low number of events and lack of data.  *SKILLS*  There is limited evidence neither in favour of FA training nor the control. A statistically significant increase of skin wound skills (correct procedures listed) using FA training compared to a sham intervention could not be demonstrated   - 11-16 year olds (Campbell, 2001)   There is limited evidence in favour of FA training. It was shown that FA training resulted in a statistically significant increase of skin wound skills (order of FA response)   - 11-16 year olds: compared to a sham intervention (Campbell, 2001)   Evidence is of very low quality and results of this study are imprecise due to low number of events and large variability of results.  *ATTITUDE*  There is no evidence available. |
| --- | --- |
| **Reference(s)** | **Articles**  Campbell NR, Ayala GX, Litrownik AJ, Slymen DJ, Zavala F, Elder JP. *Evaluation of a first aid and home safety program for hispanic migrant adolescents.* American Journal of Preventive Medicine 2001, 20(4): 258-265.  Wafik W, Tork H. *Effectiveness of a first-aid intervention program applied by undergraduate nursing students to preparatory school children.* Nursing and Health Sciences 2014, 16: 112-118.  Wilks J, Kanasa H, Pendergast D, Clark K. *Emergency response readiness for primary school children.* Aust Health Rev 2016.  **Systematic reviews**  De Buck E, Van Remoortel H, Dieltjens T, Verstraeten H, Clarysse M, Moens O, Vandekerckhove P. *Evidence-based educational pathway for the integration of first aid training in school curricula.* Resuscitation 2015, 94**,** 8-22.  Dieltjens T, De Buck E, Verstraeten H, Adriaenssens L, Clarysse M, Moens O, Devreker A, Bastiaen M, Claessens C, Verhelst K. *Evidence-based recommendations on automated external defibrillator training for children and young people in Flanders-Belgium.* Resuscitation 2013, 84**,** 1304-9.  He Z, Wynn P, Kendrick D. *Non-resuscitative first-aid training for children and laypeople: a systematic review.* Emerg Med J 2014, 31(9):763-8.  Lenson S, Mills J. *First aid knowledge retention in school children: A review of the literature.* Australasian Journal of Paramedicine 2016, 13.  Plant N, Taylor K. *How best to teach CPR to schoolchildren: a systematic review.* Resuscitation 2013, 84(4):415-21.  Reveruzzi B, Buckley L, Sheehan M. *School-Based First Aid Training Programs: A Systematic Review.* J Sch Health 2016, 86(4):266-72. |

## Burns

**Characteristics of included studies**

| **Author, year, Country** | **Study design** | **Population** | **Comparison/Risk factor** | **Remarks** |
| --- | --- | --- | --- | --- |
| Campbell, 2001, USA | Experimental: randomized controlled trial | *Nr. of participants:* 660 students:   - 51% males - 49% females   Two arms:  Intervention n=293 Control n=367  *Age range:*  11-16 years  Mean ± SD:  13 ± 1.11 years | **Intervention:**  *Program:* First aid and home safety training  *Content:* Emergency care, fever and first aid kit, bleeding, burns, fractures, dislocations and sudden illness, sports injury and prevention, poisoning, bites, stings and allergies, first aid review and household safety.  **Control:**  *Program:* Tobacco and alcohol prevention program  *Content:* Health effects of smoking and alcohol, peer pressure, decision making, societal influences and refusal skills.  *Duration (intervention + control):* 8 sessions (2 hours/session) spread over a 7-to 10-week period | Outcomes were measured before, immediately after and 1 year after training.  *Knowledge:*   - Emergency response procedures (check-call-care) - First aid kit   [only knowledge data for FA procedure were extracted]  *Skills:* Responses to two audio-recorded scenarios: scenario 1. severe wound from glass; scenario 2. severe burn injury from a toddler. Type and order of responses were scored:   - Check the scene and victim - Call 911 - Care for the victim   *Attitude:* First aid confidence |
| Frederick,  2000, UK | Experimental: controlled before after study | *Nr. of participants:* 1096 students   - Gender not reported   Two arms:  Intervention n=542  Control n=554  *Age range:*  10-11 years | **Intervention:**  *Program:* Injury Minimization Program for Schools (IMPS)  *Content:* Road safety, accidents in the home, fire, electricity, poisons, waterways, basic life support and resuscitation skills.  Lecture + video + hands-on manikin training + hospital/emergency department visit.  *Duration:* Not reported. Spread over 1 academic year  **Control:**  No intervention. | Outcomes were measured before and 5 months after training.  *Knowledge:* Self-developed quiz  *Skills:* Performance on a basic life support scenario. [only measured post intervention]  *Attitude:* Record observations of dangerous behavior in a video  [attitude data were not extracted]  [only post-training data were extracted] |
| Heard, 2013, USA | Observational:  cohort study | *Nr. of participants:* 2747 students:   - Gender not reported   Three surveys:  2011 survey n=550  2012 survey n=2197  2012 resurvey n=312 (= based on same sample as from 2011)  *Age range:*  10-15 years | **Intervention:**  *Program:* Burn care and prevention  *Content:* use of cool water on acute burn injury, burn and fire safety presentation and coloring book + crayons.  *Duration:* Not reported | Outcomes were measured before (2012) and 11 months after training (2012 resurvey).  *Knowledge:* 10-item survey on burn first aid.  [data from 2011 survey were not extracted] |
| Uray,  2003, Austria | Observational:  before after study | *Nr. of participants:*  47 students:   - 20 males - 27 females     *Age range:*  6-7 years | **Intervention:**  *Program:* First aid training  *Content*: Emergency call, CPR, AED, recovery position, bleeding and burns.  Demonstration + hands-on manikin training.  *Duration:* 1 week | Outcomes were measured before and immediately after training.  *Knowledge:* Questionnaire in which students had to place three cartoon-like illustrations in the correct sequence |
| Wafik,  2014, Egypt | Observational: before after study | *Nr. of participants:* 100 students:   - Gender not reported     *Age range:*  11-16 years  Mean ± SD:  13.2 ± 0.8 years | **Intervention:**  *Program:* First aid training  *Content:* wounds, poisoning, chemicals,  electrocution, hemorrhage, burns, fractures, choking and basic life support.  Lecture + question time + group discussion.  *Duration:* 6 sessions (1 hour/session) | Outcomes were measured before, immediately after and 2 months after training.  *Knowledge*: 32 item questionnaire.  *Skills*: Performance on five first aid scenarios (choking, burns, poisoning, and fractures). |

**Synthesis of findings**

| **Burns** | | | | | |
| --- | --- | --- | --- | --- | --- |
| **Outcome** | | **Comparison** | **Effect Size** | **#studies, # participants** | **Reference** |
| *Knowledge* | | | | | |
| - 6-7 years | | | | | |
| Knowledge: Burns FA | Pre vs post | | Statistically significant:  MD: 27%, 95%CI [11%;40%] λ  (p<0.05)*  *In favour of first aid training* | 1, 47 vs 47 §  (within subject) | Uray, 2003 |
| - 10-11 years | | | | | |
| Knowledge: Burns FA | FA training vs no intervention | | Statistically significant:  1.5±1.0 vs 1.3±1.0  MD: 0.2, 95%CI [0.08;0.32]  (p=0.0009)*  *in favour of first aid training* | 1, 542 vs 554 §(power analysis) | Frederick, 2000 |
| - 10-15 years | | | | | |
| Knowledge: what to do when clothes catch fire | Pre vs 11 month follow-up | | Statistically significant:  1873 (85%) vs 179 (57%)  OR: 0.41 £†  (p=0.001)  *in favour of first aid training* | 1, 2197 vs 312 | Heard, 2013 |
| Knowledge: Burns FA |  |  | Statistically significant:  1820 (83%) vs 258 (83%)  OR: 1.83 £†  (p=0.026)  *in favour of first aid training* |  |  |
| - 11-15 years | | | | | |
| Knowledge: Burns FA (score >60%) | Pre vs post | | Statistically significant:  5 (5%) vs 99 (99%) §  RR: 19.80* £  (p<0.001)  *in favour of first aid training* | 1, 100  (within subject) | Wafik, 2014 |
|  | Pre vs 2 month follow-up | | Statistically significant:  5 (5%) vs 100 (100%) §  RR: 20.00* £  (p<0.001)  *in favour of first aid training* |  |  |
| *Skills* | | | | | |
| - 11-15 years | | | | | |
| Skills: Burns practice (score >60%) | Pre vs post | | Statistically significant:  0 (0%) vs 94 (94%) §  RR: 189.00* £  (p<0.001)  *in favour of first aid training* | 1, 100  (within subject) | Wafik, 2014 |
|  | Pre vs 2 month follow-up | | Statistically significant:  0 (0%) vs 74 (74%) §  RR: 149.00* £  (p<0.001)  *in favour of first aid training* |  |  |
| - 11-16 years | | | | | |
| Skills: Order of FA response | FA training vs sham intervention | | Scenario (severe burn injury)  Not statistically significant:  5 (3%) vs 1 (0.5%) §  RR: 7.52, 95%CI [0.89;63.69]* ¥  (p=0.064) | 1, 147 vs 221 | Campbell, 2001 |
| Skills: Correct procedures listed |  |  | Scenario (severe burn injury)  Not statistically significant:  104 (73%) vs 149 (69%) §  RR: 1.05, 95%CI [0.91;1.21]* ¥  (p=0.486) |  |  |
| *Attitude – no studies available* | | | | | |

Mean ± SD (unless otherwise indicated), MD: mean difference, RR: risk ratio, OR: odds ratio, RD: risk difference

* calculations done by the reviewer using Revman, R software, or Excel

£ No raw data available and CI cannot be calculated.

¥ Imprecision (large variability of results)

† Imprecision (lack of data)

§ Imprecision (limited sample size or low number of events)

λ data extracted from graph

**Quality of evidence**

***Experimental studies***

| **Author, Year** | **Lack of allocation concealment** | **Lack of blinding** | **Incomplete accounting of outcome events** | **Selective outcome reporting** | **Other limitations** |
| --- | --- | --- | --- | --- | --- |
| Campbell, 2001 | Randomization and allocation concealment: unclear, not specified in the article | No | Yes, considerable loss to follow-up | Yes, data from some time-points not reported | No pre-test/baseline assessment |
| Frederick, 2000 | Randomization: yes, no randomization  Allocation concealment: not applicable | Yes, assessors were un-blinded | No | No | / |

***Observational studies***

| **Author, Year** | **Inappropriate eligibility criteria** | **Inappropriate methods for exposure and outcome variables** | **Not controlled for confounding** | **Incomplete or inadequate follow-up** | **Other limitations** |
| --- | --- | --- | --- | --- | --- |
| Heard, 2013 | Not applicable | No (not applicable for exposure variables; no flawed measurement of outcome variables) | No (corrected for age and school) | Yes (only 11% of participants were assessed after receiving the intervention) | Cohort study; inadequate statistical analysis (comparison between dependent groups as if they were independent) |
| Uray, 2003 | Yes, no inclusion of control population | No (not applicable for exposure variables; no flawed measurement of outcome variables) | Yes | No | Incorrect statistical analysis likely (2x2 table next to graph from which we extracted MD values adds up to 92 persons although a 47 person within subject design was used) |
| Wafik, 2014 | Yes, no inclusion of control population | No (not applicable for exposure variables; no flawed measurement of outcome variables) | Yes | No | / |

**Certainty of the body of evidence**

KNOWLEDGE

|  | **Initial grading Low [C]** | Downgrading due to |
| --- | --- | --- |
| **Limitations of study design** | -1 | See table ‘Quality of evidence’ |
| **Imprecision** | -1 | Limited sample sizes/low number of events + lack of data |
| **Inconsistency** | 0 |  |
| **Indirectness** | 0 |  |
| **Publication bias** | 0 |  |
| **QUALITY (GRADE)** | **Final grading Very low [D]** |  |

SKILLS

|  | **Initial grading Low [C]** | Downgrading due to |
| --- | --- | --- |
| **Limitations of study design** | -1 | See table ‘Quality of evidence’ |
| **Imprecision** | -1 | Low number of events + lack of data + large variability of the results |
| **Inconsistency** | 0 |  |
| **Indirectness** | 0 |  |
| **Publication bias** | 0 |  |
| **QUALITY (GRADE)** | **Final grading Very low [D]** |  |

ATTITUDE

There is no evidence available.

| **Conclusion** | *KNOWLEDGE*  There is limited evidence in favour of FA training. It was shown that FA training resulted in a statistically significant increase of burn knowledge in   - 6-7 year olds: pre to post (Uray, 2003) - 10-11 year olds: compared to no intervention (Frederick, 2000) - 10-15 year olds: pre to 11m follow-up (Heard, 2013) - 11-15 year olds: pre to post, and pre to 2m follow-up (Wafik, 2014)   Evidence is of very low quality and results of these studies are imprecise due to limited sample sizes, low number of events and lack of data.  *SKILLS*  There is limited evidence neither in favour of FA training nor the control. A statistically significant increase of burn FA skills using FA training compared to sham intervention could not be demonstrated in   - 11-16 year olds (Campbell, 2001)   There is limited evidence in favour of FA training. It was shown that FA training resulted in a statistically significant increase of burn skills in   - 11-15 year olds: pre to post, and pre to 2m follow-up (Wafik, 2014)   Evidence is of very low quality and results of these studies are imprecise due to low number of events, lack of data and large variability of results.  *ATTITUDE*  There is no evidence available. |
| --- | --- |
| **Reference(s)** | **Articles**  Campbell NR, Ayala GX, Litrownik AJ, Slymen DJ, Zavala F, Elder JP. *Evaluation of a first aid and home safety program for hispanic migrant adolescents.* American Journal of Preventive Medicine 2001, 20(4): 258-265.  Frederick K, Bixby E, Orzel MN, Stewart-Brown S, Willett K. *An evaluation of the effectiveness of the injury minimization programme for schools (IMPS).* Injury prevention 2000, 6: 92-95.  Heard JP, Latenser BA, Liao J. *Burn prevention in Zambia: a work in progress.* Journal of Burn Care & Research 2013, 34(6): 598-606.  Uray T, Lunzer A, Ochsenhofer A, Thanikkel L, Zingerle R, Lillie P, Brandl E, Sterz F, LSFA school study group. *Feasibility of life-supporting first-aid (LSFA) training as a mandatory subject in primary schools.* Resuscitation 2003, 59: 211-220.  Wafik W, Tork H. *Effectiveness of a first-aid intervention program applied by undergraduate nursing students to preparatory school children.* Nursing and Health Sciences 2014, 16: 112-118.  **Systematic reviews**  De Buck E, Van Remoortel H, Dieltjens T, Verstraeten H, Clarysse M, Moens O, Vandekerckhove P. *Evidence-based educational pathway for the integration of first aid training in school curricula.* Resuscitation 2015, 94**,** 8-22.  Dieltjens T, De Buck E, Verstraeten H, Adriaenssens L, Clarysse M, Moens O, Devreker A, Bastiaen M, Claessens C, Verhelst K. *Evidence-based recommendations on automated external defibrillator training for children and young people in Flanders-Belgium.* Resuscitation 2013, 84**,** 1304-9.  He Z, Wynn P, Kendrick D. *Non-resuscitative first-aid training for children and laypeople: a systematic review.* Emerg Med J 2014, 31(9):763-8.  Lenson S, Mills J. *First aid knowledge retention in school children: A review of the literature.* Australasian Journal of Paramedicine 2016, 13.  Plant N, Taylor K. *How best to teach CPR to schoolchildren: a systematic review.* Resuscitation 2013, 84(4):415-21.  Reveruzzi B, Buckley L, Sheehan M. *School-Based First Aid Training Programs: A Systematic Review.* J Sch Health 2016, 86(4):266-72. |

## Bleeding

Characteristics of included studies

| **Author, year, Country** | **Study design** | **Population** | **Comparison/Risk factor** | **Remarks** |
| --- | --- | --- | --- | --- |
| Campbell, 2001, USA | Experimental: randomized controlled trial | *Nr. of participants:* 660 students:   - 51% males - 49% females   Two arms:  Intervention n=293 Control n=367  *Age range:*  11-16 years  Mean ± SD:  13 ± 1.11 years | **Intervention:**  *Program:* First aid and home safety training  *Content:* Emergency care, fever and first aid kit, bleeding, burns, fractures, dislocations and sudden illness, sports injury and prevention, poisoning, bites, stings and allergies, first aid review and household safety.  **Control:**  *Program:* Tobacco and alcohol prevention program  *Content:* Health effects of smoking and alcohol, peer pressure, decision making, societal influences and refusal skills.  *Duration (intervention + control):* 8 sessions (2 hours/session) spread over a 7-to 10-week period | Outcomes were measured before, immediately after and 1 year after training.  *Knowledge:*   - Emergency response procedures (check-call-care) - First aid kit   [only knowledge data for FA procedure were extracted]  *Skills:* Responses to two audio-recorded scenarios: scenario 1. severe wound from glass; scenario 2. severe burn injury from a toddler. Type and order of responses were scored:   - Check the scene and victim - Call 911 - Care for the victim   *Attitude:* First aid confidence |
| Wafik,  2014, Egypt | Observational: before after study | *Nr. of participants:* 100 students:   - Gender not reported     *Age range:*  11-16 years  Mean ± SD:  13.2 ± 0.8 years | **Intervention:**  *Program:* First aid training  *Content:* wounds, poisoning, chemicals,  electrocution, hemorrhage, burns, fractures, choking and basic life support.  Lecture + question time + group discussion.  *Duration:* 6 sessions (1 hour/session) | Outcomes were measured before, immediately after and 2 months after training.  *Knowledge*: 32 item questionnaire.  *Skills*: Performance on five first aid scenarios (choking, burns, poisoning, and fractures). |
| Wilks, 2016, Australia | Observational: before after study | *Nr. of participants*: 107 students:   - 51 males - 56 females   *Age range*:  11-12 years  Mean ± SD:  11.4 ± 0.5 years | **Intervention:**  *Program*: First aid, CPR and beach safety training  *Content*:   - Beach safety - Leadership and team bonding exercises - First aid: sprains, bleeding, choking, heart attack, allergic reactions - Hands-on manikin training   *Duration:* 1 day | Outcomes were measured before, 1 week after, and 8 weeks after training.  *Knowledge:* 50-item quiz on emergency services and life-supporting first aid |

**Synthesis of findings**

| **Bleeding** | | | | | |
| --- | --- | --- | --- | --- | --- |
| **Outcome** | | **Comparison** | **Effect Size** | **#studies, # participants** | **Reference** |
| *Knowledge* | | | | | |
| - 11-12 years | | | | | |
| Knowledge: cuts and bleeding FA | Pre vs 1 week post | | Statistically significant:  74 (69%) vs 94 (90%) §  RR: 1.33* £  (p<0.001- overall p)  *in favour of first aid training* | 1, 107 vs 102  (within subject) | Wilks, 2016 |
|  | Pre vs 8 week follow-up | | Statistically significant:  74 (69%) vs 93 (82%) §  RR: 1.28* £  (p<0.001- overall p)  *in favour of first aid training* | 1, 107 vs 105  (within subject) |  |
| - 11-15 years | | | | | |
| Knowledge: Haemorrhage FA (score >60%) | Pre vs post | | Statistically significant:  34 (34%) vs 97 (97%) §  RR: 2.85* £  (p<0.001)  *in favour of first aid training* | 1, 100  (within subject) | Wafik, 2014 |
|  | Pre vs 2 month follow-up | | Statistically significant:  34 (34%) vs 92 (92%) §  RR: 2.71* £  (p<0.001)  *in favour of first aid training* |  |  |
| *Skills* | | | | | |
| - 11-16 years | | | | | |
| Skills: Order of FA response | FA training vs sham intervention | | Scenario (severe glass wound)  Statistically significant:  21 (14%) vs 10 (5%) §  RR: 3.16, 95%CI [1.53;6.51]*  (p<0.001)  *in favour of first aid training* | 1, 147 vs 221 | Campbell, 2001 |
| Skills: Correct procedures listed |  |  | Scenario (severe glass wound)  Not statistically significant:  75 (52%) vs 125 (57%) §  RR: 0.90, 95%CI [0.74;1.10]* ¥  (p=0.413) |  |  |
| *Attitude – no studies available* | | | | | |

Mean ± SD (unless otherwise indicated), MD: mean difference, RR: risk ratio, OR: odds ratio, RD: risk difference

* calculations done by the reviewer using Revman, R software, or Excel

£ No raw data available and CI cannot be calculated.

¥ Imprecision (large variability of results)

† Imprecision (lack of data)

§ Imprecision (limited sample size or low number of events)

λ data extracted from graph

Quality of evidence

***Experimental studies***

| **Author, Year** | **Lack of allocation concealment** | **Lack of blinding** | **Incomplete accounting of outcome events** | **Selective outcome reporting** | **Other limitations** |
| --- | --- | --- | --- | --- | --- |
| Campbell, 2001 | Randomization and allocation concealment: unclear, not specified in the article | No | Yes, considerable loss to follow-up | Yes, data from some time-points not reported | No pre-test/baseline assessment |

***Observational studies***

| **Author, Year** | **Inappropriate eligibility criteria** | **Inappropriate methods for exposure and outcome variables** | **Not controlled for confounding** | **Incomplete or inadequate follow-up** | **Other limitations** |
| --- | --- | --- | --- | --- | --- |
| Wafik, 2013 | Yes, no inclusion of control population | No (not applicable for exposure variables; no flawed measurement of outcome variables) | Yes | No | / |
| Wilks, 2016 | Yes, no inclusion of control population | No, a questionnaire was used | Unclear, statistics not reported | No | / |

**Certainty of the body of evidence**

KNOWLEDGE

|  | **Initial grading Low [C]** | Downgrading due to |
| --- | --- | --- |
| **Limitations of study design** | -1 | See table ‘Quality of evidence’ |
| **Imprecision** | -1 | Low number of events + lack of data |
| **Inconsistency** | 0 |  |
| **Indirectness** | 0 |  |
| **Publication bias** | 0 |  |
| **QUALITY (GRADE)** | **Final grading Very low [D]** |  |

SKILLS

|  | **Initial grading High [A]** | Downgrading due to |
| --- | --- | --- |
| **Limitations of study design** | -1 | See table ‘Quality of evidence’ |
| **Imprecision** | -1 | Low number of events + large variability of the results |
| **Inconsistency** | -1 | Impact of FA training on bleeding skills is not consistent |
| **Indirectness** | 0 |  |
| **Publication bias** | 0 |  |
| **QUALITY (GRADE)** | **Final grading Very low [D]** |  |

ATTITUDE

There is no evidence available.

| **Conclusion** | *KNOWLEDGE*  There is limited evidence in favour of FA training. It was shown that FA training resulted in a statistically significant increase of bleeding knowledge in   - 11-12 year olds: pre to post, and pre to 8w follow-up (Wilks, 2016) - 11-15 year olds: pre to post, and pre to 2m follow-up (Wafik, 2014)   Evidence is of very low quality and results of these studies are imprecise due to low number of events and lack of data.  *SKILLS*  There is limited evidence neither in favour of FA training nor the control. A statistically significant increase of bleeding skills (correct procedures listed) using FA training compared to a sham intervention could not be demonstrated in   - 11-16 year olds (Campbell, 2001)   There is limited evidence in favour of FA training. It was shown that FA training resulted in a statistically significant increase of bleeding skills (order of FA response) in   - 11-16 year olds: compared to a sham intervention (Campbell, 2001)   Evidence is of very low quality and results of this study are imprecise due to low number of events and large variability of results.  *ATTITUDE*  There is no evidence available. |
| --- | --- |
| **Reference(s)** | **Articles**  Campbell NR, Ayala GX, Litrownik AJ, Slymen DJ, Zavala F, Elder JP. *Evaluation of a first aid and home safety program for hispanic migrant adolescents.* American Journal of Preventive Medicine 2001, 20(4): 258-265.  Wafik W, Tork H. *Effectiveness of a first-aid intervention program applied by undergraduate nursing students to preparatory school children.* Nursing and Health Sciences 2014, 16: 112-118.  Wilks J, Kanasa H, Pendergast D, Clark K. *Emergency response readiness for primary school children.* Aust Health Rev 2016.  **Systematic reviews**  De Buck E, Van Remoortel H, Dieltjens T, Verstraeten H, Clarysse M, Moens O, Vandekerckhove P. *Evidence-based educational pathway for the integration of first aid training in school curricula.* Resuscitation 2015, 94**,** 8-22.  Dieltjens T, De Buck E, Verstraeten H, Adriaenssens L, Clarysse M, Moens O, Devreker A, Bastiaen M, Claessens C, Verhelst K. *Evidence-based recommendations on automated external defibrillator training for children and young people in Flanders-Belgium.* Resuscitation 2013, 84**,** 1304-9.  He Z, Wynn P, Kendrick D. *Non-resuscitative first-aid training for children and laypeople: a systematic review.* Emerg Med J 2014, 31(9):763-8.  Lenson S, Mills J. *First aid knowledge retention in school children: A review of the literature.* Australasian Journal of Paramedicine 2016, 13.  Plant N, Taylor K. *How best to teach CPR to schoolchildren: a systematic review.* Resuscitation 2013, 84(4):415-21.  Reveruzzi B, Buckley L, Sheehan M. *School-Based First Aid Training Programs: A Systematic Review.* J Sch Health 2016, 86(4):266-72. |

## Injuries to bones, muscles or joints

Characteristics of included studies

| **Author, year, Country** | **Study design** | **Population** | **Comparison/Risk factor** | **Remarks** |
| --- | --- | --- | --- | --- |
| Uray,  2003, Austria | Observational:  before after study | *Nr. of participants:*  47 students:   - 20 males - 27 females     *Age range:*  6-7 years | **Intervention:**  *Program:* First aid training  *Content*: Emergency call, CPR, AED, recovery position, bleeding and burns.  Demonstration + hands-on manikin training.  *Duration:* 1 week | Outcomes were measured before and immediately after training.  *Knowledge:* Questionnaire in which students had to place three cartoon-like illustrations in the correct sequence |
| Wafik,  2014, Egypt | Observational: before after study | *Nr. of participants:* 100 students:   - Gender not reported     *Age range:*  11-16 years  Mean ± SD:  13.2 ± 0.8 years | **Intervention:**  *Program:* First aid training  *Content:* wounds, poisoning, chemicals,  electrocution, hemorrhage, burns, fractures, choking and basic life support.  Lecture + question time + group discussion.  *Duration:* 6 sessions (1 hour/session) | Outcomes were measured before, immediately after and 2 months after training.  *Knowledge*: 32 item questionnaire.  *Skills*: Performance on five first aid scenarios (choking, burns, poisoning, and fractures). |

**Synthesis of findings**

| **Injuries to bones, muscles or joints** | | | | | |
| --- | --- | --- | --- | --- | --- |
| **Outcome** | | **Comparison** | **Effect Size** | **#studies, # participants** | **Reference** |
| *Knowledge* | | | | | |
| - 6-7 years | | | | | |
| Knowledge: Trauma FA | Pre vs post | | Statistically significant:  MD: 32%, 95%CI [21%;45%]  (p<0.05)*  *In favour of first aid training* | 1, 47 vs 47 §  (within subject) | Uray, 2003 |
| - 11-15 years | | | | | |
| Knowledge: Fractures FA (score >60%) | Pre vs post | | Statistically significant:  5 (5%) vs 99 (99%) §  RR: 19.80* £  (p<0.001)  *in favour of first aid training* | 1, 100  (within subject) | Wafik, 2014 |
|  | Pre vs 2 month follow-up | | Statistically significant:  5 (5%) vs 97 (97%) §  RR: 19.40* £  (p<0.001)  *in favour of first aid training* |  |  |
| *Skills* | | | | | |
| - 11-15 years | | | | | |
| Skills: Fractures practice (score >60%) | Pre vs post | | Statistically significant:  0 (0%) vs 88 (88%) §  RR: 177.00* £  (p<0.001)  *in favour of first aid training* | 1, 100  (within subject) | Wafik, 2014 |
|  | Pre vs 2 month follow-up | | Statistically significant:  0 (0%) vs 67 (67%) §  RR: 135.00* £  (p<0.001)  *in favour of first aid training* |  |  |
| *Attitude – no studies available* | | | | | |

Mean ± SD (unless otherwise indicated), MD: mean difference, RR: risk ratio, OR: odds ratio, RD: risk difference

* calculations done by the reviewer using Revman, R software, or Excel

£ No raw data available and CI cannot be calculated.

¥ Imprecision (large variability of results)

† Imprecision (lack of data)

§ Imprecision (limited sample size or low number of events)

λ data extracted from graph

Quality of evidence

***Observational studies***

| **Author, Year** | **Inappropriate eligibility criteria** | **Inappropriate methods for exposure and outcome variables** | **Not controlled for confounding** | **Incomplete or inadequate follow-up** | **Other limitations** |
| --- | --- | --- | --- | --- | --- |
| Uray, 2003 | Yes, no inclusion of control population | No (not applicable for exposure variables; no flawed measurement of outcome variables) | Yes | No | Incorrect statistical analysis likely (2x2 table next to graph from which we extracted MD values adds up to 92 persons although a 47 person within subject design was used) |
| Wafik, 2014 | Yes, no inclusion of control population | No (not applicable for exposure variables; no flawed measurement of outcome variables) | Yes | No | / |

**Certainty of the body of evidence: Injuries to bones, muscles or joints**

KNOWLEDGE

|  | **Initial grading Low [C]** | Downgrading due to |
| --- | --- | --- |
| **Limitations of study design** | -1 | See table ‘Quality of evidence’ |
| **Imprecision** | -1 | Limited sample sizes/low number of events + lack of data |
| **Inconsistency** | 0 |  |
| **Indirectness** | 0 |  |
| **Publication bias** | 0 |  |
| **QUALITY (GRADE)** | **Final grading Very low [D]** |  |

SKILLS

|  | **Initial grading Low [C]** | Downgrading due to |
| --- | --- | --- |
| **Limitations of study design** | -1 | See table ‘Quality of evidence’ |
| **Imprecision** | -1 | Low number of events + lack of data |
| **Inconsistency** | 0 |  |
| **Indirectness** | 0 |  |
| **Publication bias** | 0 |  |
| **QUALITY (GRADE)** | **Final grading Very low [D]** |  |

ATTITUDE

There is no evidence available.

| **Conclusion** | *KNOWLEDGE*  There is limited evidence in favour of FA training. It was shown that FA training resulted in a statistically significant increase of knowledge related to bones, muscles or joints injuries in   - 6-7 year olds: pre to post (Uray, 2003) - 11-15 year olds: pre to post, and pre to 2m follow-up (Wafik, 2014)   Evidence is of very low quality and results of these studies are imprecise due to limited sample sizes, low number of events and lack of data.  *SKILLS*  There is limited evidence in favour of FA training. It was shown that FA training resulted in a statistically significant increase of skills related to bones, muscles or joints injuries in   - 11-15 year olds: pre to post, and pre to 2m follow-up (Wafik, 2014)   Evidence is of very low quality and results of this study are imprecise due to low number of events and lack of data.  *ATTITUDE*  There is no evidence available. |
| --- | --- |
| **Reference(s)** | **Articles**  Uray T, Lunzer A, Ochsenhofer A, Thanikkel L, Zingerle R, Lillie P, Brandl E, Sterz F, LSFA school study group. *Feasibility of life-supporting first-aid (LSFA) training as a mandatory subject in primary schools.* Resuscitation 2003, 59: 211-220.  Wafik W, Tork H. *Effectiveness of a first-aid intervention program applied by undergraduate nursing students to preparatory school children.* Nursing and Health Sciences 2014, 16: 112-118.  **Systematic reviews**  De Buck E, Van Remoortel H, Dieltjens T, Verstraeten H, Clarysse M, Moens O, Vandekerckhove P. *Evidence-based educational pathway for the integration of first aid training in school curricula.* Resuscitation 2015, 94**,** 8-22.  Dieltjens T, De Buck E, Verstraeten H, Adriaenssens L, Clarysse M, Moens O, Devreker A, Bastiaen M, Claessens C, Verhelst K. *Evidence-based recommendations on automated external defibrillator training for children and young people in Flanders-Belgium.* Resuscitation 2013, 84**,** 1304-9.  He Z, Wynn P, Kendrick D. *Non-resuscitative first-aid training for children and laypeople: a systematic review.* Emerg Med J 2014, 31(9):763-8.  Lenson S, Mills J. *First aid knowledge retention in school children: A review of the literature.* Australasian Journal of Paramedicine 2016, 13.  Plant N, Taylor K. *How best to teach CPR to schoolchildren: a systematic review.* Resuscitation 2013, 84(4):415-21.  Reveruzzi B, Buckley L, Sheehan M. *School-Based First Aid Training Programs: A Systematic Review.* J Sch Health 2016, 86(4):266-72. |

## Poisoning

Characteristics of included studies

| **Author, year, Country** | **Study design** | **Population** | **Comparison/Risk factor** | **Remarks** |
| --- | --- | --- | --- | --- |
| Frederick,  2000, UK | Experimental: controlled before after study | *Nr. of participants:* 1096 students   - Gender not reported   Two arms:  Intervention n=542  Control n=554  *Age range:*  10-11 years | **Intervention:**  *Program:* Injury Minimization Program for Schools (IMPS)  *Content:* Road safety, accidents in the home, fire, electricity, poisons, waterways, basic life support and resuscitation skills.  Lecture + video + hands-on manikin training + hospital/emergency department visit.  *Duration:* Not reported. Spread over 1 academic year  **Control:**  No intervention. | Outcomes were measured before and 5 months after training.  *Knowledge:* Self-developed quiz  *Skills:* Performance on a basic life support scenario. [only measured post intervention]  *Attitude:* Record observations of dangerous behavior in a video  [attitude data were not extracted]  [only post-training data were extracted] |
| Wafik,  2014, Egypt | Observational: before after study | *Nr. of participants:* 100 students:   - Gender not reported     *Age range:*  11-16 years  Mean ± SD:  13.2 ± 0.8 years | **Intervention:**  *Program:* First aid training  *Content:* wounds, poisoning, chemicals,  electrocution, hemorrhage, burns, fractures, choking and basic life support.  Lecture + question time + group discussion.  *Duration:* 6 sessions (1 hour/session) | Outcomes were measured before, immediately after and 2 months after training.  *Knowledge*: 32 item questionnaire.  *Skills*: Performance on five first aid scenarios (choking, burns, poisoning, and fractures). |

**Synthesis of findings**

| **Poisoning** | | | | | |
| --- | --- | --- | --- | --- | --- |
| **Outcome** | | **Comparison** | **Effect Size** | **#studies, # participants** | **Reference** |
| *Knowledge* | | | | | |
| - 10-11 years | | | | | |
| Knowledge: Recognize poisoning | FA training vs no intervention | | Not statistically significant:  2.2±1.0 vs 2.2±1.0  MD: 0.0, 95%CI [-0.12;0.12]  (p=1.00)* | 1, 542 vs 554 §(power analysis) | Frederick, 2000 |
| - 11-15 year | | | | | |
| Knowledge: Poisoning (score >60%) | Pre vs post | | Statistically significant:  5 (5%) vs 98 (98%) §  RR: 19.60* £  (p<0.001)  *in favour of first aid training* | 1, 100  (within subject) | Wafik, 2014 |
|  | Pre vs 2 month follow-up | | Statistically significant:  5 (5%) vs 99 (99%) §  RR: 19.80* £  (p<0.001)  *in favour of first aid training* |  |  |
| *Skills* | | | | | |
| - 11-15 years | | | | | |
| Skills: Poisoning practice (score >60%) | Pre vs post | | Statistically significant:  0 (0%) vs 91 (91%) §  RR: 183.00* £  (p<0.001)  *in favour of first aid training* | 1, 100  (within subject) | Wafik, 2014 |
|  | Pre vs 2 month follow-up | | Statistically significant:  0 (0%) vs 59 (59%) §  RR: 119.00* £  (p<0.001)  *in favour of first aid training* |  |  |
| *Attitude – no studies available* | | | | | |

Mean ± SD (unless otherwise indicated), MD: mean difference, RR: risk ratio, OR: odds ratio, RD: risk difference

* calculations done by the reviewer using Revman, R software, or Excel

£ No raw data available and CI cannot be calculated.

¥ Imprecision (large variability of results)

† Imprecision (lack of data)

§ Imprecision (limited sample size or low number of events)

λ data extracted from graph

Quality of evidence

***Experimental studies***

| **Author, Year** | **Lack of allocation concealment** | **Lack of blinding** | **Incomplete accounting of outcome events** | **Selective outcome reporting** | **Other limitations** |
| --- | --- | --- | --- | --- | --- |
| Frederick, 2000 | Randomization: yes, no randomization  Allocation concealment: not applicable | Yes, assessors were un-blinded | No | No | / |

***Observational studies***

| **Author, Year** | **Inappropriate eligibility criteria** | **Inappropriate methods for exposure and outcome variables** | **Not controlled for confounding** | **Incomplete or inadequate follow-up** | **Other limitations** |
| --- | --- | --- | --- | --- | --- |
| Wafik, 2014 | Yes, no inclusion of control population | No (not applicable for exposure variables; no flawed measurement of outcome variables) | Yes | No | / |

**Certainty of the body of evidence: Poisoning**

KNOWLEDGE

|  | **Initial grading Low [C]** | Downgrading due to |
| --- | --- | --- |
| **Limitations of study design** | -1 | See table ‘Quality of evidence’ |
| **Imprecision** | -1 | Low number of events + lack of data |
| **Inconsistency** | 0 |  |
| **Indirectness** | 0 |  |
| **Publication bias** | 0 |  |
| **QUALITY (GRADE)** | **Final grading Very low [D]** |  |

SKILLS

|  | **Initial grading Low [C]** | Downgrading due to |
| --- | --- | --- |
| **Limitations of study design** | -1 | See table ‘Quality of evidence’ |
| **Imprecision** | -1 | Low number of events + lack of data |
| **Inconsistency** | 0 |  |
| **Indirectness** | 0 |  |
| **Publication bias** | 0 |  |
| **QUALITY (GRADE)** | **Final grading Very low [D]** |  |

ATTITUDE

There is no evidence available.

| **Conclusion** | *KNOWLEDGE*  There is limited evidence neither in favour of FA training nor the control. A statistically significant increase of poisoning knowledge using FA training compared to no intervention could not be demonstrated in   - 10-11 year olds (Frederick, 2000)   There is limited evidence in favour of FA training. It was shown that FA training resulted in a statistically significant increase of poisoning knowledge in   - 11-15 year olds: pre to post, and pre to 2m follow-up (Wafik, 2014)   Evidence is of very low quality and results of these studies are imprecise due to low number of events and lack of data.  *SKILLS*  There is limited evidence in favour of FA training. It was shown that FA training resulted in a statistically significant increase of poisoning skills in   - 11-15 year olds: pre to post, and pre to 2m follow-up (Wafik, 2014)   Evidence is of very low quality and results of these studies are imprecise due to low number of events, and lack of data.  *ATTITUDE*  There is no evidence available. |
| --- | --- |
| **Reference(s)** | **Articles**  Frederick K, Bixby E, Orzel MN, Stewart-Brown S, Willett K. *An evaluation of the effectiveness of the injury minimization programme for schools (IMPS).* Injury prevention 2000, 6: 92-95.  Wafik W, Tork H. *Effectiveness of a first-aid intervention program applied by undergraduate nursing students to preparatory school children.* Nursing and Health Sciences 2014, 16: 112-118.  **Systematic reviews**  De Buck E, Van Remoortel H, Dieltjens T, Verstraeten H, Clarysse M, Moens O, Vandekerckhove P. *Evidence-based educational pathway for the integration of first aid training in school curricula.* Resuscitation 2015, 94**,** 8-22.  Dieltjens T, De Buck E, Verstraeten H, Adriaenssens L, Clarysse M, Moens O, Devreker A, Bastiaen M, Claessens C, Verhelst K. *Evidence-based recommendations on automated external defibrillator training for children and young people in Flanders-Belgium.* Resuscitation 2013, 84**,** 1304-9.  He Z, Wynn P, Kendrick D. *Non-resuscitative first-aid training for children and laypeople: a systematic review.* Emerg Med J 2014, 31(9):763-8.  Lenson S, Mills J. *First aid knowledge retention in school children: A review of the literature.* Australasian Journal of Paramedicine 2016, 13.  Plant N, Taylor K. *How best to teach CPR to schoolchildren: a systematic review.* Resuscitation 2013, 84(4):415-21.  Reveruzzi B, Buckley L, Sheehan M. *School-Based First Aid Training Programs: A Systematic Review.* J Sch Health 2016, 86(4):266-72. |

## Bee and wasp stings

Characteristics of included studies

Not applicable

**Synthesis of findings**

Not applicable

Quality of evidence

Not applicable

| **Conclusion** | No evidence was found using the above mentioned search strategy and criteria. |
| --- | --- |
| **Reference(s)** | / |

## Snake bites

Characteristics of included studies

Not applicable

**Synthesis of findings**

Not applicable

Quality of evidence

Not applicable

| **Conclusion** | No evidence was found using the above mentioned search strategy and criteria. |
| --- | --- |
| **Reference(s)** | / |

## Fever

Characteristics of included studies

Not applicable

**Synthesis of findings**

Not applicable

Quality of evidence

Not applicable

| **Conclusion** | No evidence was found using the above mentioned search strategy and criteria. |
| --- | --- |
| **Reference(s)** | / |

## Diarrhoea

Characteristics of included studies

| **Author, year, Country** | **Study design** | **Population** | **Comparison/Risk factor** | **Remarks** |
| --- | --- | --- | --- | --- |
| Galiani, 2012, Peru | Experimental: cluster randomized controlled trial | *Nr. of participants:* 880 households (intervention) vs 1640 households (control); exact number of children going to the local primary schools not available  *Age range:* 5-12 years | **Intervention:**  *Program:* Global Scaling Up Hand­washing Project  *Content:* The activities  in schools included designating a place in the classroom for soap, performing regular handwashing practices in groups each day, weekly handwashing promotion classes, and other children’s activities such as  singing songs and drawing posters. As part of the programme, training of the community and capacity building for mothers was also foreseen.  *Duration:* activities spread over 3 years  **Control:** no training | Included study in systematic review (De Buck, 2017).  Outcomes were measured before and immediately after the implementation period of 3 years.  *Knowledge*: knowledge about not washing hands as the cause of diarrhoea and about handwashing was measured with a questionnaire. |
| Lansdown, 2002, Tanzania | Experimental: cluster randomized controlled trial | *Nr. of participants:*  560  - Gender not reported  Two arms:  Intervention n=336  Control n=224  *Age range:* 7-15 years | **Intervention:**  *Program:* Lushoto Enhanced Health Education Project  *Content:* School teachers were introduced to active teaching methods as well as being given some knowledge on  parasitology and ways of preventing infection. After returning to their  schools, teachers widened their work to include the importance of clean drinking water and good nutrition. In some schools the prevention of locally common diseases was taught. Songs, poetic dramas, short plays,  visits and discussions were commonly used. All but one of the schools had motto boards or daily message boards.  *Duration:* activities spread over 11 months  **Control:** no training | Included study in systematic review (De Buck, 2017).  Outcomes were measured before and immediately after the implementation period of 11 months.  *Knowledge*: general health knowledge (including preventing parasitic infections) was measured with a questionnaire. |
| Maiti, 2012, India | Experimental: before-after study | *Nr. of participants:* 827  - 433 males  - 394 females  *Age range:* 10-15 years | **Intervention:**  *Program:* health awareness package  *Content:* visual presentation,movie, leaflets and posters on malaria,tuberculosis, diarrhoea, dysentery and cholera, followed by an interactive session on misconceptions and myths about the diseases  *Duration:* 1 hour for each disease in each class  [only data on knowledge about diarrhoea were extracted] | Outcomes were measured before and 6 months following the intervention  *Knowledge*: measured with a questionnaire on causes, signs, symp­toms, complications, mode of transmission, method of prevention and role of children to control the diseases. |

**Synthesis of findings**

| **Diarrhoea** | | | | | |
| --- | --- | --- | --- | --- | --- |
| **Outcome** | | **Comparison** | **Effect Size** | **#studies, # participants** | **Reference** |
| *Knowledge* | | | | | |
| - 5-12 years | | | | | |
| Knowledge: not washing hands with water and soap is the main cause of diarrhoea | Education vs no education | | Not statistically significant:  0.9510±0.3350 vs 0.940±0.3350*  MD: 0.011, 95%CI  [-0.0164;0.0384]*  (p=0.43) | 1, 880 vs 1640 | Galiani, 2012 |
| - 7-15 years | | | | | |
| General health knowledge | Education vs no education | | Statistically significant:  14.85±7.79 vs 12.14±4.65  MD: 2.71, 95%CI [2.31;3.11]* (p<0.00001)  *In favour of education* | 1, 168 vs 112 § | Lansdown, 2002 |
| - 10-14 years | | | | | |
| Knowledge: cause of diarrhoea, modes of infection, signs and symptoms, modes of prevention, role of children | Pre vs post | | Statistically significant:  1.18 vs 3.08  MD: 1.9* £†  (p<0.05)  *In favour of education* | 1, 827 vs 827 (within subjects) | Maiti, 2012 |

Mean ± SD (unless otherwise indicated), MD: mean difference, RR: risk ratio

* calculations done by the reviewer using Revman

£ No raw data available, CI cannot be calculated

§ Imprecision (limited sample size)

† Imprecision (lack of data)

Quality of evidence

| **Author, Year** | **Lack of allocation concealment** | **Lack of blinding** | **Incomplete accounting of outcome events** | **Selective outcome reporting** | **Other limitations** |
| --- | --- | --- | --- | --- | --- |
| Galiani, 2012 | Randomization: unclear, not specified in the article  Allocation concealment: unclear, not specified in the article | participants: unclear, not specified in the article  outcome assessors: no | unclear, not specified in the article | No | / |
| Lansdown, 2002 | Randomization: unclear, not specified in the article  Allocation concealment: unclear, not specified in the article | participants: no  outcome assessors: yes | no | yes | / |
| Maiti, 2012 | Randomization: yes, no randomization  Allocation concealment: not applicable (not randomized) | participants: not applicable (within subjects)  outcome assessors: unclear, not specified in the article | no | yes, dysentery knowledge questionnaire data seems to not have been reported | / |

**Certainty of the body of evidence**

KNOWLEDGE

|  | **Initial grading high [A]** | Downgrading due to |
| --- | --- | --- |
| **Limitations of study design** | -1 | See table ‘Quality of evidence’ |
| **Imprecision** | 0 |  |
| **Inconsistency** | 0 |  |
| **Indirectness** | 0 |  |
| **Publication bias** | 0 |  |
| **QUALITY (GRADE)** | **Final grading moderate [B]** |  |

| **Conclusion** | *KNOWLEDGE*  There is evidence in favour of education on diarrhoea knowledge items. It was shown that school programmes aimed at children of 7-15 years and 10-14 years, focussing on diarrhoea and general health, and lasting about 1 year, resulted in a statistically significant increase of general health knowledge, causes and signs and symptoms of diarrhoea and prevention of diarrhoea, compared to not providing such a programme (Lansdown, 2002; Maiti, 2012). However, knowledge about the cause of diarrhoea could not be demonstrated in case of 3 year-long programme with children of 5 to 12 years old (Galiani, 2015)  Evidence is of moderate quality. |
| --- | --- |
| **Reference(s)** | **Articles**  Galiani S, Gertler P, Orsola-Vidal A. *Promoting Handwashing Behavior in Peru: The Effects of Large-scale Mass media and Community Level Interventions*. The World Bank Sustainable Development Network Water and Sanitation Program 2012, Policy Research Working Paper 6257  Lansdown R, Ledward A, Hall A, Issae W, Yona E, Matulu J, Mweta M, Kihamia C, Nyandindi U, Bundy D. Schistosomiasis, helminth infection and health education in Tanzania: achieving behavior change in primary schools. Health Educ Res 2002, 17(4):425-33  Maiti S, Chatterjee K, Monjur Ali K, Jana K, Kanti Bera T, Ghosh D. *Evaluation of the health awareness package for the improvement of knowledge, Attitudes and practices (KAP) of secondary school students at rural areas of Paschim Medinipur,*  *West Bengal*. Indian Journal of Public Health Research and Development 2012, 3(4):41-46  **Systematic reviews**  De Buck E, Van Remoortel H, Hannes K, Govender T, Naidoo S, Avau B,  Vande veegaete A, Musekiwa A, Vittoria L, Cargo M, Mosler H-J, Vandekerckhove P, Young T. *Approaches to promote handwashing and sanitation behaviour change in low- and middle-income countries: a mixed method systematic review.* Campbell Systematic Reviews 2017:7 |

## Epilepsy

Characteristics of included studies

| **Author, year, Country** | **Study design** | **Population** | **Comparison/Risk factor** | **Remarks** |
| --- | --- | --- | --- | --- |
| Brabcova, 2013, Czech Republic | Experimental: non-randomized controlled trial | *Nr. of participants:*  1342   - 701 males - 641 females   Three arms:  Intervention 1  n=762   - 404 males - 358 females   Intervention 2  n=400   - 201 males - 199 females   Control  n=180   - 96 males - 84 females   *Age range:* 9-11 years | **Intervention 1:**  *Program:* Turen der Gik I Fisk (Adventures While  Fishing)  *Content:* video with a variety of animated images depicting 3 different types of seizures and basic information about first aid for epilepsy and epilepsy treatment. Main emphasis that children with epilepsy can be just as happy as their peers.  *Duration:* 20 min  **Intervention 2:**  *Program:* Island Adventure  *Content:* educational drama with children actively participating in acting a similar story to the video above including imitating the types of seizures. Children communicated their feelings and were allowed to invite an expert (doctor) into the game who answered their question on epilepsy and its health and social aspects  *Duration:* not provided  **Control:**  No intervention | Outcomes were measured immediately after and 6 months after the intervention.  *Knowledge*:  Questionnaire with 4 out of 7 questions on knowledge of epilepsy  [attitude data (3 out of 7 questions in questionnaire) not extracted]  [data from immediately after intervention not extracted since there was no control or baseline to compare to] |
| Brabcova, 2017, Czech Republic | Experimental:  before after study | *Nr. of participants:*  182   - 85 males - 97 females   Two arms:  Intervention 1  n=89   - 52 males - 37 females   Intervention 2  n=93   - 33 males - 60 females   *Age range:* 9-12 years  Mean±SD Intervention 1: 9.6±0.6 years  Mean±SD Intervention 2: 10.6±0.7 years | **Intervention 1:**  *Program:* Turen der Gik I Fisk (Adventures While  Fishing) – educational video  *Content:* video with a variety of animated images depicting 3 different types of seizures and basic information about first aid for epilepsy and epilepsy treatment. Main emphasis that children with epilepsy can be just as happy as their peers.  *Duration:* 20 min  **Intervention 2:**  *Program:* Turen der Gik I Fisk (Adventures While  Fishing) – educational story  *Content:* video described in Intervention 1 was transcribed preserving language and content. The story was the slowly and carefully read to the children  *Duration:* not provided | Outcomes were measured before, immediately after and and 6 months after the intervention.  *Knowledge*:  11-item questionnaire  [data from immediately after intervention not extracted since there was no control and analysis for comparison to baseline could not be performed due to within-subject design] |
| Kolar Sridara Murthy, 2016, India | Experimental:  before after study | *Nr. of participants:*  70  *Age range:* 12-16 years  Mean ± SD:  14.55 ± 1.33 | **Intervention:**  *Program:* Intervention package on epilepsy awareness for knowledge, attitude and first aid practices  *Content:*  epilepsy awareness for the students with respect to their existing knowledge, attitude, and myths and misconceptions of epilepsy; first aid in epilepsy; belief and practices about epilepsy; and role of students in school.  posters + brochures + focus group discussions + 12-minute video on first aid practices  *Duration:* 12 minute video on first aid + session with discussion (no duration reported) | Outcomes were measured before and 1 week after the intervention.  *Knowledge*:  Knowledge on epilepsy: 8-item questionnaire  Knowledge on first aid practices: 9-item questionnaire  [attitude data not extracted] |
| Martiniuk, 2007, Australia | Experimental: cluster randomized controlled trial | *Nr. of participants:*  783 individuals from 24 schools in Canada  Two arms:  Intervention n=403 from 12 schools  Control n=380 from 12 schools  *Age range:* 9-11 years | **Intervention:**  *Program:* Thinking about Epilepsy education program  *Content:*  knowledge: the role of the brain (location, parts, and function), epilepsy (what epilepsy is, types, and manifestations), diagnosis (EEGs and treatment), first-aid (for tonic–clonic seizures), and causes. attitudes: contagiousness (sharing a drink, sitting beside), friendship (no one’s fault, play with, can tell who has epilepsy by looks, hard to make friends), and that epilepsy should not limit achievement (intelligence, academic performance, participation in sports).  *Duration:* 30 minutes  **Control:**  Delayed intervention (wait-list control) | Outcomes were measured before and 1 month after the intervention  *Knowledge*: 18-item questionnaire  [attitude data not extracted] |
| Simon, 2016, Austria | Experimental: controlled before-after study | *Nr. of participants:*  168  Two arms:  Intervention n=105  Control n=63  *Age range:* 14-17 years | **Intervention:**  *Program:* Teaching unit on epilepsy to incorporate in biology curriculum  *Content:* 3 lessons incorporating several different teaching methods (among others video, discussion, worksheet) on seizures (and types), epilepsy diagnosis, people with epilepsy and their daily lives, therapies for epilepsy and first aid for seizures  *Duration:* 3 consecutive biology lessons with 45 min each  **Control:**  no intervention | Outcomes were measured one week before the intervention (pre-test), one week after the final lesson in each class (post-test) and another two months later (follow-up test)  *Knowledge*: Questionnaire with 37 out of 44 questions on knowledge about concerning epilepsy  [attitude data (7 out of 44 questions in questionnaire) not extracted] |

**Synthesis of findings**

| **Epilepsy** | | | | | |
| --- | --- | --- | --- | --- | --- |
| **Outcome** | | **Comparison** | **Effect Size** | **#studies, # participants** | **Reference** |
| *Knowledge* | | | | | |
| - 9-11 years | | | | | |
| Knowledge: | Education vs no education | | Statistically significant:  Regression: -9.12, 95%CI [-10.05;-8.18]  (p<0.0001)  *in favour of education* | 1, 403 vs 380 | Martiniuk, 2007 |
| Knowledge: how do you know someone has epilepsy? (seizure) | Video vs no intervention | | Not statistically significant:  743 (97.5%) vs 171 (95.0%)  RR: 1.03, 95%CI [0.99;1.06]*  (p=0.146) | 1, 762 vs 180 | Brabcova, 2013 |
|  | Educational drama vs no intervention | | Not statistically significant:  385 (96.3%) vs 171 (95.0%)  RR: 1.01, 95%CI [0.97;1.05]*  (p=0.508) | 1, 400 vs 180 |  |
| Knowledge: what happens when someone has an epileptic seizure? (different symptoms) | Video vs no intervention | | Statistically significant:  727 (95.4%) vs 151 (83.9%)  RR: 1.14, 95%CI [1.06;1.21]*  (p<0.001)  *in favour of education* | 1, 762 vs 180 |  |
|  | Educational drama vs no intervention | | Statistically significant:  373 (93.3%) vs 151 (83.9%)  RR: 1.11, 95%CI [1.04;1.19]*  (p=0.002)  *in favour of education* | 1, 400 vs 180 |  |
| Knowledge: What would you do during seizure? (get help) | Video vs no intervention | | Statistically significant:  675 (88.6%) vs 27 (15.0%)  RR: 5.91, 95%CI [4.17;8.37]*  (p<0.001)  *in favour of education* | 1, 762 vs 180 |  |
|  | Educational drama vs no intervention | | Statistically significant:  328 (82.0%) vs 27(15.0%)  RR: 5.47, 95%CI [3.85;7.76]*  (p<0.001)  *in favour of education* | 1, 400 vs 180 |  |
| Knowledge: what do children with epilepsy have to do? (medication) | Video vs no intervention | | Statistically significant:  740 (97.1%) vs 158 (87.8%)  RR:1.11, 95%CI [1.05;1.17]*  (p<0.001)  *in favour of education* | 1, 762 vs 180 |  |
|  | Educational drama vs no intervention | | Not statistically significant:  368 (92.0%) vs 158 (87.8%)  RR: 1.05, 95%CI [0.99;1.11]*  (p=0.133) | 1, 400 vs 180 |  |
| Knowledge: what would you have to change if you had epilepsy? (medication) | Video vs no intervention | | Not statistically significant:  661 (86.8%) vs 150 (83.3%)  RR: 1.04, 95%CI [0.97;1.12]*  (p=0.27) | 1, 762 vs 180 |  |
|  | Educational drama vs no intervention | | Not statistically significant:  328 (82.0%) vs 150 (83.3%)  RR: 0.98, 95%CI [0.91;1.07]*  (p=0.694) | 1, 400 vs 180 |  |
| - 9-12 years | | | | | |
| Overall score: Knowledge test | Video: pre vs 6-month follow-up | | Statistically significant:  6.58±1.98 vs 9.09±1.56  MD: 2.51* £  (p<0.001)  *in favour of education* | 1, 89 vs 89 (within subject) § | Brabcova 2017 |
|  | Story: pre vs 6-month follow-up | | Statistically significant:  6.88±2.24 vs 8.99±1.58  MD: 2.11* £  (p<0.001)  *in favour of education* | 1, 93 vs 93 (within subject) § |  |
| Knowledge: first aid during seizure (recovery position) | Video: pre vs 6-month follow-up | | Statistically significant:  51 (57.3%) vs 87 (97.8%) §  RR:1.71* £  (p<0.001)  *in favour of education* | 1, 89 vs 89 (within subject) |  |
|  | Story: pre vs 6-month follow-up | | Statistically significant:  66 (71%) vs 86 (92.5%) §  RR: 1.30* £  (p<0.001)  *in favour of education* | 1, 93 vs 93 (within subject) |  |
| Knowledge: why should you put person on his side (first aid)? | Video: pre vs 6-month follow-up | | Statistically significant:  39 (43.8%) vs 72 (80.9%) §  RR: 1.85 £  (p<0.001)  *in favour of education* | 1, 89 vs 89 (within subject) |  |
|  | Story: pre vs 6-month follow-up | | Statistically significant:  60 (64.5%) vs 75 (80.6%) §  RR: 1.25* £  (p=0.007)  *in favour of education* | 1, 93 vs 93 (within subject) |  |
| Knowledge: how do you know someone has epilepsy? (seizure) | Video: pre vs 6-month follow-up | | Statistically significant:  72 (80.9%) vs 87 (97.8%) §  RR: 1.21* £  (p<0.001)  *in favour of education* | 1, 89 vs 89 (within subject) |  |
|  | Story: pre vs 6-month follow-up | | Not statistically significant:  81 (87.1%) vs 86 (92.5%) §  RR: 1.06* £†  (p=0.113) | 1, 93 vs 93 (within subject) |  |
| Knowledge: does epilepsy look the same with everyone (different symptoms) | Video: pre vs 6-month follow-up | | Not statistically significant:  50 (56.2%) vs 59 (66.3%) §  RR: 1.18* £†  (p=0168) | 1, 89 vs 89 (within subject) |  |
|  | Story: pre vs 6-month follow-up | | Not statistically significant:  58 (62.4%) vs 60 (64.5%) §  RR: 1.03* £†  (p=0.382) | 1, 93 vs 93 (within subject) |  |
| Knowledge: what do children with epilepsy have to do (medication) | Video: pre vs 6-month follow-up | | Statistically significant:  68 (76.4%) vs 86 (96.6%) §  RR: 1.26* £  (p<0.001)  *in favour of education* | 1, 89 vs 89 (within subject) |  |
|  | Story: pre vs 6-month follow-up | | Statistically significant:  78 (83.9%) vs 93 (100%) §  RR: 1.19* £  (p<0.001)  *in favour of education* | 1, 93 vs 93 (within subject) |  |
| Knowledge: what would you have to change if you had epilepsy? (medication) | Video: pre vs 6-month follow-up | | Statistically significant:  63 (70.8%) vs 77 (86.5%) §  RR: 1.22* £  (p=0.005)  *in favour of education* | 1, 89 vs 89 (within subject) |  |
|  | Story: pre vs 6-month follow-up | | Statistically significant:  73 (78.5%) vs 86 (92.5%) §  RR: 1.18* £  (p=0.003)  *in favour of education* | 1, 93 vs 93 (within subject) |  |
| Knowledge: can you recognize person with epilepsy at first sight | Video: pre vs 6-month follow-up | | Statistically significant:  53 (59.6%) vs 79 (88.8%) §  RR: 1.49* £  (p<0.001)  *in favour of education* | 1, 89 vs 89 (within subject) |  |
|  | Story: pre vs 6-month follow-up | | Statistically significant:  52 (55.9%) vs 81 (87.1%) §  RR: 1.56* £  (p<0.001)  *in favour of education* | 1, 93 vs 93 (within subject) |  |
| Knowledge: how does a person contract epilepsy? | Video: pre vs 6-month follow-up | | Not statistically significant:  55 (61.8%) vs 55 (61.8%) §  RR: 1.00* £  (p=1.000) | 1, 89 vs 89 (within subject) |  |
|  | Story: pre vs 6-month follow-up | | Statistically significant:  55 (59.1%) vs 71 (76.3%) §  RR: 1.29* £  (p=0.006)  *in favour of education* | 1, 93 vs 93 (within subject) |  |
| Knowledge: which organ is affected by epilepsy? | Video: pre vs 6-month follow-up | | Not statistically significant:  59 (66.3%) vs 65 (73.0%) §  RR: 1.10* £†  (p=0.327) | 1, 89 vs 89 (within subject) |  |
|  | Story: pre vs 6-month follow-up | | Statistically significant:  32 (34.4%) vs 58 (62.4%) §  RR: 1.81* £  (p<0.001)  *in favour of education* | 1, 93 vs 93 (within subject) |  |
| Knowledge: how do doctors diagnose someone with epilepsy from brain waves? | Video: pre vs 6-month follow-up | | Statistically significant:  38 (42.7%) vs 74 (83.1%) §  RR: 1.95* £  (p<0.001)  *in favour of education* | 1, 89 vs 89 (within subject) |  |
|  | Story: pre vs 6-month follow-up | | Statistically significant:  40 (43%) vs 62 (66.7%) §  RR: 1.55* £  (p<0.001)  *in favour of education* | 1, 93 vs 93 (within subject) |  |
| Knowledge: do you think epilepsy can be treated? | Video: pre vs 6-month follow-up | | Statistically significant:  38 (42.7%) vs 68 (76.4%) §  RR:1.79* £  (p<0.001)  *in favour of education* | 1, 89 vs 89 (within subject) |  |
|  | Story: pre vs 6-month follow-up | | Statistically significant:  45 (48.4%) vs 78 (83.9%) §  RR: 1.73 £  (p<0.001)  *in favour of education* | 1, 93 vs 93 (within subject) |  |
| - 12-16 years | | | | | |
| Knowledge: knowledge on epilepsy | Pre vs post | | Statistically significant:  3.47±1.13 vs 7.60±0.52  MD: 4.13 £  (p<0.001)  *in favour of education* | 1, 70 vs 70 (within subject) § | Kolar Sridara Murthy, 2016 |
| Knowledge: first aid practices for epilepsy |  |  | Statistically significant:  1.36±0.96 vs 7.67±0.50  MD: 6.31 £  (p<0.001)  *in favour of education* |  |  |
| - 14-17 years | | | | | |
| Knowledge: overall | Education vs no education | | Statistically significant:  GLM: (p<0.01) £  *in favour of education* | 1, 105 vs 63 § | Simon, 2016 |
| Knowledge: seizures (%) | Pre vs post | | Statistically significant:  43.0%±19.5% vs 81.3%±16.4%  MD: 38.3 £  Post-hoc test of GLM  (p<0.05)  *in favour of education* | 1, 105 vs 105 (within subject) § |  |
| Knowledge: daily life aspects of epilepsy (%) |  |  | Statistically significant:  58.8%±21.5% vs 80.7%±18.4%  MD: 21.9 £  Post-hoc test of GLM  (p<0.05)  *in favour of education* |  |  |
| Knowledge: medical knowledge about epilepsy (%) |  |  | Statistically significant:  24.0%±21.5% vs 89.8%±17.4%  MD: 65.8 £  Post-hoc test of GLM  (p<0.05)  *in favour of education* |  |  |
| Knowledge: first-aid concerning epilepsy (%) |  |  | Statistically significant:  40.2±20.5 vs 89.3±16.4  MD: 49.1 £  Post-hoc test of GLM  (p<0.05)  *in favour of education* |  |  |

Mean ± SD (unless otherwise indicated), MD: mean difference, RR: risk ratio

* Calculations done by the reviewer using Revman

£ No raw data available and CI cannot be calculated.

§ Imprecision (limited sample size or low number of events)

† Imprecision (lack of data)

Quality of evidence

***Experimental studies***

| **Author, Year** | **Lack of allocation concealment** | **Lack of blinding** | **Incomplete accounting of outcome events** | **Selective outcome reporting** | **Other limitations** |
| --- | --- | --- | --- | --- | --- |
| Brabcova, 2013 | Randomization:  Yes, no randomization  Allocation concealment: Not applicable (not randomized) | Participants:  No, participants were from different schools to avoid contamination effects  Outcome assessors:  No, no information provided but a questionnaire was used | No | No | Yes, method of execution of the educational drama probably varied to some extent between groups and this might affect the effectiveness of that intervention |
| Brabcova, 2017 | Randomization:  Yes, no randomization  Allocation concealment: Not applicable (not randomized) | Participants:  Not applicable (within subjects design)  Outcome assessors:  No, no information provided but a questionnaire was used | No, only 18 respondents (9%) were not included in the final analysis of which 16 due to illness and 2 due to mostly unfilled questionnaires | No | Yes, no passive control group included, base characteristics of the two intervention groups were quite different and teacher enthusiasm in delivery of the story might affect intervention |
| Kolar Sridara Murthy, 2016 | Randomization:  Yes, no randomization  Allocation concealment:  Not applicable (not randomized) | Participants:  Not applicable (within subjects design)  Outcome assessors:  No, no information provided but a questionnaire was used | No | No | Yes, reported data for individual questions regarding practices seems contradictory |
| Martiniuk, 2007 | Randomization:  No, blocked stratified randomization generated with Minitab software randomization function  Allocation concealment:  Unclear, not specified in the article | Participants: No, neither teachers nor students were informed about their study status  Outcome assessors:  No, not specified in the article but self-administered questionnaire used for assessment minimizing impact | No, 78 (10%) lost to follow-up, but sensitivity analyses showed that loss did not account for difference between intervention and control group | No |  |
| Simon, 2016 | Randomization:  Yes, no randomization  Allocation concealment:  Not applicable (not randomized) | Participants:  Unclear, not specified in the article  Outcome assessors:  No, no information provided but a questionnaire was used | No | No |  |

**Certainty of the body of evidence**

KNOWLEDGE

|  | **Initial grading High [A]** | Downgrading due to |
| --- | --- | --- |
| **Limitations of study design** | -1 | See table ‘Quality of evidence’ |
| **Imprecision** | -1 |  |
| **Inconsistency** | 0 |  |
| **Indirectness** | 0 |  |
| **Publication bias** | 0 |  |
| **QUALITY (GRADE)** | **Final grading Low [C]** |  |

| **Conclusion** | *KNOWLEDGE*  There is limited evidence in favour of education on epilepsy knowledge items. In making this evidence conclusion we place a higher value on significant results over non-significant results.  It was shown that an educational intervention resulted in a significant increase in overall knowledge in   - 9-11 year olds: intervention (education programme) vs no intervention (Martiniuk, 2007) - 9-11 year olds: intervention (video or educational drama) vs no intervention (Brabcova 2013) - 9-12 year olds: story or video, pre vs 6 month follow-up (Brabcova 2017) - 12-16 year olds: pre vs post (Kolar Sridara Murthy 2016) - 14-17 year olds: education vs no intervention and pre vs post (Simon 2016)   Evidence is of low quality and results cannot be considered precise due to limited sample size, low number of events and/or lack of data. |
| --- | --- |
| **Reference(s)** | **Articles**  Brabcova D, Lovasova V, Kohout J, Zarubova J, Komarek V. *Improving the knowledge of epilepsy and reducing epilepsy-related stigma among children using educational video and educational drama--a comparison of the effectiveness of both interventions.* Seizure 2013, 22(3):179-84.  Brabcová D, Kohout J, Weberová V, Komárek V. Educational video and story as effective interventions reducing epilepsy-related stigma among children. Epilepsy Behav 2017, 69:12-17.  Kolar Sridara Murthy M, Govindappa L, Sinha S. Outcome of a school-based health education program for epilepsy awareness among schoolchildren. Epilepsy Behav 2016, 57(Pt A):77-81.  Martiniuk AL, Speechley KN, Secco M, Campbell MK, Donner A. Evaluation of an epilepsy education program for Grade 5 students: a cluster randomized trial. Epilepsy Behav 2007, 10(4):604-10.  Simon UK, Gesslbauer L, Fink A. *A Three-Lesson Teaching Unit Significantly Increases High School Students' Knowledge about Epilepsy and Positively Influences Their Attitude towards This Disease.* PLoS One. 2016 Feb 26;11(2):e0150014. |
