# Supplementary material for: An educational pathway and teaching materials for first aid training of children in sub-Saharan Africa based on the best available evidence
Source: BMC Public Health. 2020 Jun 3;20:836. doi: 10.1186/s12889-020-08857-5 (PMC7268765; doi:10.1186/s12889-020-08857-5)
Supplement: Supplementary file 8 — Additional file 8. Characteristics of included studies, synthesis of findings, risk of bias and certainty of evidence for research question 2 [file 12889_2020_8857_MOESM8_ESM.docx]

# Additional file 8: Characteristics of included studies, synthesis of findings, risk of bias and certainty of evidence for research question 2

## Characteristics of included studies

We identified 2 systematic reviews that answer our PICO question and fulfil our in- and exclusion criteria (Snilstveit, 2015 and Conn, 2014). Below, the characteristics of these 2 included systematic reviews are provided, including their selection criteria. Thereafter, the characteristics of the individual studies included in these systematic reviews are listed, since detailed information on the intervention could be useful to interpret the results.

| **Author, year, Country** | **Study design** | **Population** | **Comparison** | **Remarks** |
| --- | --- | --- | --- | --- |
| Conn, 2014, USA | Systematic review and meta-analysis  Selection criteria used for study design:  Experimental or quasi-experimental studies were included:  - Randomized controlled trials  - Difference-in-difference specifications  - Instrumental variables methods  - Matching methods (propensity score, non-parametric or simple covariate matching)  - Regression discontinuity designs  - Time series models with fixed effects  Excluded:  Randomized controlled trials that only randomize 2 classrooms or schools, even if a student-level randomization between groups is conducted | 56 papers, corresponding to 66 studies with 83 treatment arms and 420 effect size estimates  Selection criteria used for population:  Students in any formal level of education in Sub-Saharan Africa were included | Twelve different types of education interventions or programs that aim to improve learning outcomes, repetition, drop-out, completion, retention, enrolment and attendance rates in Sub-Saharan Africa  vs  no intervention, business as usual  Selection criteria used for intervention:  The following intervention areas were included:  (1) Quality of instruction:  - After-school tutoring  - Class size  - Technology-assisted learning  - Instructional time  - Pedagogical interventions/teacher training  - Language of instruction  - School supplies provision  - Tracking & peer effects  (2) Student or community financial limitations:  - Abolishment of school fees  - Cash transfers  - School infrastructure  - School choice  (3) School or system accountability:  - High stakes testing & accountability systems  - Information for accountability  -School-based management/ decentralization  - School type  (4) Student cognitive processing abilities:  - School meals  - Health treatments  (5) Student or teacher motivation:  - Student incentives  - Teacher incentives  Selection criteria used for comparison:  Control/”status quo” comparisons were included | From this systematic review,  we only extracted information on the effect of one category of interventions,  *i.e.* “Pedagogical interventions/ teacher training”.  For this evidence summary, we altered the name of this category to  “Use of alternative pedagogical methods”.  Selection criteria used for outcomes:  One or more of the following outcome measures:  - Learning outcomes  - Repetition  - Drop-out  - Completion  - Retention  - Enrolment  - Attendance  Selection criteria used for publication type:  - Peer-reviewed journals  - Academic working papers  - Reports published through academic institution or research organisations  Selection criteria used for publication date:  1980-“present”  (*i.e.* April 2013) |
| Snilstveit, 2015, New Delhi | Systematic review and meta-analysis  Selection criteria used for study design of quantitative studies:  Experimental or quasi-experimental studies that allow for causal inference were included:  - Experimental study designs  - Regression discontinuity designs  - Natural experiments  - Studies using statistical matching or regression adjustment  - Quasi-experimental studies that include a baseline measure of the outcome variables and control for appropriate covariates  Excluded:  - Studies with less than 2 teachers or schools in each group, to avoid confounding treatment effects - Efficacy trials, because these trials test an intervention under ideal and controlled conditions, and not under ‘real-world’ circumstances | 420 papers, corresponding to 238 studies and 216 unique interventions  Selection criteria used for population:  Included were primary and secondary school age children (age depending on the country, in most countries 4/5+ years) in mainstream education in low- and middle-income countries, as defined by the World Bank at the point in time that an intervention was carried out.  Excluded:  - Studies in refugees, migrants and orphans only  - Studies focussing on children with special educational needs  - Adult education interventions  - Studies from high-income countries | A range of interventions designed to improve the access and/or quality of primary and secondary school education in low- and middle-income countries  vs  no intervention, business as usual, or a different form of educational intervention  Selection criteria used for intervention:  The following interventions were included:  (1) Child level:  - School feeding programmes  - School-based health programmes  - Providing information to children  - Merit-based scholarships  (2) Household level:  - Cash transfers  - Scholarships and allowances  - Reducing or eliminating school user fees  - Providing information to parents  (3) School level:  - Instructional approach, content, time and organisation interventions (divided into more specific sub-categories: computer-assisted learning, structured pedagogy, extra time, remedial education, grade retention, tracking)  - New schools & infrastructure  - Providing materials  (4) Teacher level:  - Teacher incentives  - Teacher training  - Hiring additional teachers  (5) System level:  - School-based management  - Community-based monitoring  - Public private partnerships and private provision of schooling  Selection criteria used for comparison:  Studies using any type of comparison group (no intervention including wait-list comparisons as part of pipe-line designs, business as usual, different form of educational intervention) at the school, student or area level (*e.g.* school districts) were included. | From this systematic review, we only extracted information on the effect of two categories of school level interventions,  *i.e.* “Providing materials” and “Structured pedagogy”.  For this evidence summary, we altered the name of the first category to  “Provision of instructional materials”.  Selection criteria used for outcomes:  To be included, studies had to assess at least one of the following education- related primary or secondary outcomes:  (1) Primary outcomes:  - Enrolment  - Attendance  - Drop-out  - Completion  - Learning (focused on the key domains of maths & language arts, cognitive and problem solving skills, or composite assessment scores from test scores in different subjects or other measures of skills and learning)  (2) Secondary outcomes:  - Teacher attendance  - Teacher performance  - Other secondary and intermediate outcomes if they were reported in studies that met all other inclusion criteria.  Selection criteria used for publication date:  Any studies published before 1990 were excluded.  This systematic review is a mixed-methods systematic review. This means that, in addition to the quantitative studies mentioned here, the authors also included qualitative studies, process evaluations and project documents related to the programmes studied in the included (quantitative) impact evaluations. From these documents, descriptive information on programme design and implementation was extracted by the authors, as well as information on context and resources and findings on barriers and facilitators of intervention success or failure. |
| **PROVISION OF INSTRUCTIONAL MATERIALS = textbooks, flip-charts or grants used for materials** | | | | |
| Das, 2013, USA | Experimental: Cluster randomized controlled trial | 200 public primary schools in the rural Indian state of Andhra Pradesh were randomly assigned to the intervention (100 schools) or control group (100 schools).  Students attended grade 2-5 (total number of students and their age is not reported). | Intervention: schools received a school grant worth around $3 per pupil. The funds were to be spent on inputs used directly by students and not on infrastructure or construction projects. The majority of the grant was typically spent on notebooks, writing materials, workbooks, and stationery material (not on textbooks because they are provided freely by the government).  Control: no intervention.  [It is unclear which effect sizes (year 1 and/or year 2) were used by Snilstveit, 2015 in their meta-analyses.] | Identified from the systematic review of Snilstveit, 2015.  In the first year, the grant was a surprise for recipient  schools. In the second year, it was anticipated by parents  and teachers.  Maths and language assessment was conducted at baseline and at the end of each of the 2 school years in the intervention period. |
| Glewwe, 2004, USA | Experimental: Cluster randomized controlled trial | 178 primary schools in the rural Kenyan districts of Busia and Teso were randomly assigned to the intervention (89 schools, 9984 students) or control group (89 schools, 10462 students).  Students attended grade 6-8 (their age is not reported). | Intervention: each school received two sets of science flip-charts (one on agriculture and one on general science), as well as a teacher’s guide for science, one set of charts for health, one set of charts for mathematics, and a wall map of East Africa for geography.  Each set of flip-charts contains about 12 individual charts spiral bound together. Each individual chart covers different aspects of the topic.  The charts were not kept in the classroom, but rather brought in when they were relevant to the day’s lesson, and could therefore be used in more than one classroom on any given day.  Control: no intervention. | Identified from the systematic review of Snilstveit, 2015.  Pre-intervention test scores data are averaged across all subjects from practice exams in 1996. Post-intervention test scores are individual scores from KCPE or practice exams in 1997 and 1998.  An example of a science flip-chart is included in the paper. |
| Glewwe, 2009, USA | Experimental: Cluster randomized controlled trial | 100 primary schools in the rural Kenyan districts of Busia and Teso were randomly divided in 4 treatment groups of 25 schools each:  1) Textbook schools  2) Grant schools 1997  3) Grant schools 1998  4) Grant schools 2000.  Students attended grade 3-8 (total number of students and their age is not specified). | Textbook schools: in early 1996, students received official government textbooks on English (grade 3-7), Math (grades 3-7), Science (grade 8) or Agriculture (grade 8).  A 60 % textbook per student ratio was used for English and science, and a 50 % ratio for math.  Students in grades 3-5 could not take textbooks  home, but students in grade 6-8 were put in pairs to share textbooks and were could take the textbook home on alternate days.  Grant schools: in early 1997, group 2 received grants equal to US$2.65 per student or US$727 per school. Group 3 and 4 received similar grants in early 1998 and 2000 respectively.  [Snilstveit, 2015 extracted the data comparing textbook schools with grant schools that had not yet received their grants (no intervention).  It is unclear which effect sizes (year 1 and/or year 2) they used in their meta-analyses.] | Identified from the systematic review of Snilstveit, 2015.  District-wide exams and KCPE exams (only for grade 8 students), as well as additional tests were conducted at baseline and after 1 and 2 years.  This article also reports data on drop-out and completion rates. |
| Sabarwal, 2014, USA | Experimental: Cluster randomized controlled trial | In each of Sierra Leone’s 4 regions of Kambia, Kailahun, Pujehun and Western Urban/Western Rural, 90 community primary schools were randomly assigned into 3 groups of 30 schools:  1) Receiving textbooks  2) Receiving textbooks and teacher training  3) Control group.  The teacher training component (group 2) was not undertaken due to multiple implementation challenges. Hence, schools either received textbooks (intervention) or did not (control).  Students attended grade 4-5 (total number of students and their age is not reported). | Intervention: schools were provided by the government of Sierra Leone with a set of core textbooks (English, Mathematics, Integrated Science and Social Studies textbooks) for every child in the school.  Control: no intervention. | Identified from the systematic review of Snilstveit, 2015.  Data (head-teacher survey, a classroom teacher survey, a student survey, and student exams) were collected at baseline and after ± 19 months.  This article also reports data on enrolment and attendance rates, and teacher attendance and performance rates. |

| **USE OF ALTERNATIVE PEDAGOGICAL METHODS = problem-solving instruction, constructivist instruction, guided-inquiry instruction, cooperative instruction, small-group instruction** | | | | | | | | |
| --- | --- | --- | --- | --- | --- | --- | --- | --- |
| Abdu-Raheem, 2012, Nigeria | Experimental:  Cluster randomized controlled trial | | 240 junior secondary school students were randomly selected from 6 secondary schools (40 per school) in the Nigerian state of Ekiti and randomly assigned to the intervention or control group.  Students attended grade 8 (their age is not reported). | | Intervention: problem-solving method of teaching during a period of 6 weeks, including techniques such as questioning, sorting, field trips, interviewing, brainstorming, role-playing, use of projects, use of resource persons, library search and other creative activities.  Control: conventional lecturing method. | | | Identified from the systematic review of Conn, 2014.  Social studies achievement test (SSAT) was conducted before, immediately after the intervention (6 weeks) and 6 weeks after the end of the intervention. |
| Ajaja, 2010, Nigeria | Experimental: Cluster randomized controlled trial | | 120 junior secondary 3 students, attending grade 9, in the Abavo Mixed Secondary school in the Nigerian city of Abavo, were stratified according to their scholastic ability and then randomly and proportionally assigned to 4 classes of 30 students each. Two classes formed the intervention group, the other two the control group.  2 experienced teachers were randomly assigned to teach the intervention and control groups. | | Intervention: cooperative teaching method for integrated science during a period of 6 weeks. In this group, students discussed the learning material, helped each other, or developed projects in groups.  Control: traditional classroom teaching method for a period of 6 weeks: students read the assigned reading material silently, completed assignments independently at their seats, engaged in discussions with the teacher in response to the teacher’s questions.  [Conn, 2014 only used the effect size of the assessment data collected after the end of the intervention in her meta-analysis.] | | | Identified from the systematic review of Conn, 2014.  Scholastic Ability Test in Integrated Science (SATIS), Students’ Attitude Scale (SAS), and Integrated Science Achievement Test (ISAT) were conducted before, during (at the end of every of the 6 weeks) and after the 6-week intervention. |
| Bimbola, 2010, Nigeria | Experimental: Cluster randomized controlled trial | | 4 public junior secondary schools in the South-West Nigerian state of Ogun were randomly selected and randomly assigned to the intervention group (2 schools) or control group (2 schools).  Students (120 in total) attended grade 9 (their age is not reported). | | Intervention: constructivist instruction by the researchers and their assistants for a period of 3 weeks, using the following materials:  (1) A work scheme consisting of selected integrated science topics (writing chemical equation, work and energy). (2) An instructional package with the use of constructivist instruction. In constructivism, the learner is actively involved, because he must actively construct new information onto the foundation of previous learning for meaningful learning to occur.  Control: conventional lecturing instruction by the researchers and their assistants for a period of 3 weeks, using the following materials:  (1) The same work scheme as the intervention group.  (2) An instructional package with the use of conventional lecture instruction. | | | Identified from the systematic review of Conn, 2014.  A test with 45 multiple-choice questions on integrated science was conducted before, immediately after and two weeks after the intervention had ended. |
| Brooker, 2013, India | Experimental: Cluster randomised controlled trial | | 101 government primary schools in the rural Kenyan districts of Kwale and Msambweni were randomly selected and randomly assigned to either of 4 groups:  (1) Malaria intervention (25 schools, 1571 students)  (2) Literacy intervention (25 schools, 1428 students)  (3) Malaria + literacy intervention (26 schools, 1660 students)  (4) Control (25 schools, 1518 students).  The total sample consisted of 5223 students attending grades 1 and 5. The literacy intervention was targeted at grade 1 students, aged 5-15 years (average age 8 years). | | Literacy intervention: consisting of the following 3 components:  1) Teachers received a teaching manual containing 140 sequential lessons for teachers in English and Swahili. The lessons were structured to guide the teacher in what to say, what to do (i.e. with their hands or materials), which instructional materials to use and the estimated time of the lesson. The plans build from existing teaching methods (e.g. choral repetition, use of song) and show teachers how these methods can be modified slightly to promote successful  beginning reading instruction.  2) Teacher training, including a 3-day initial workshop providing background information about how children learn to read, explaining how to use the teaching manual and giving the opportunity to customise materials for use in their classroom. Also, follow-up workshops were conducted.  3) Ongoing support for teachers through weekly text messages providing  brief instructional tips and motivation to implement lesson plans. Teachers also received credit of $0.50 each week for their mobile phones.  Control: no intervention.  [Conn, 2014 only extracted data on the literacy intervention group and the control group.] | | | Identified from the systematic review of Conn, 2014.  Literacy, numeracy, classroom attention and cognitive function were assessed at baseline, after 9 months and after 24 months. |
| Githua, 2008, Kenya | Experimental:  Cluster randomized controlled trial | | 4 mixed-sex secondary schools from the Kenyan district of Nakuru were randomly selected and randomly assigned to the intervention (2 schools) or control group (2 schools).  Students (142 in total) attended grade 11 (their age is not reported). | | Intervention: maths teachers were trained for 5 days on the  creation and use of advance organisers. These are tools used to introduce the lesson topic and illustrate the relationship between what the students are about to learn and the information they have already learned. In this study, analogies were used at the beginning of each maths lesson, which were presented explicitly and involved real life situations of business transactions, well adapted to the students and the social context in Kenya.  Control: maths teachers were not trained, so were expected to use conventional or traditional instructional methods. | | | Identified from the systematic review of Conn, 2014.  The Mathematics Achievement Test (MAT) was conducted before (for one control and one intervention school) and after the intervention (for all 4 schools). |
| Kiboss, 2012, Kenya | Experimental: Cluster randomized controlled trial | | 4 special education primary schools in the Kenyan province of Rift Valley were randomly assigned to the intervention (2 schools) or control group (2 schools).  Students (66 in total) attended grade 3 and were aged between 9 and 14 years. | | Intervention: Special Electronic Learning Program (SELP) course on geometry shapes and pattern making, consisting of 8 lessons of 35 minutes. The software makes maximum use of visual perception while transmitting information in both text format and sign language format. It not only provides information, but also remediates, asks questions and judges responses of students.  A 20-hour orientation session was used to familiarize students and their teachers to the software.  Instructional manuals on the use of equipment were provided to the students and their teachers.  Control: conventional method of teaching. | | | Identified from the systematic review of Conn, 2014.  This computer-based intervention and its targeted hearing-impaired population are out of scope for this evidence summary. However, we report this study here as it is included in the meta-analysis by Conn on the effect of pedagogical methods.  The Geometry Achievement Test (GAT) and the Special Learners’ Classroom Environment Questionnaire (SLCEQ) were conducted before (for one control and one intervention school) and after the intervention (for all 4 schools). |
| Korsah, 2010, USA | Experimental:  Randomized controlled trial (within subjects design) | | 3 schools in the Ghanaian capital of Accra were recruited:  - 1 private school in a middle-income community - 1 public school in a low-income community - 1 informal educational program for highly disadvantaged children who have never attended formal school.  89 students attending grade 2-4 were randomly assigned (across school, grade and gender boundaries) to the intervention-first (46 children, 50% male) or control-first group (43 children, 51% male). Their age is not reported.  A cross-over study design was used: the intervention-first group received the intervention during the first 9 weeks and subsequently had no intervention during the next 9 weeks. In the control-first group, this was the other way around. | | Intervention: computer-assisted learning intervention using an automated computer-based reading tutor.  The tutor is a tool that displays stories on screen and “listens” to a child read aloud. Using automated speech recognition to analyze the child’s reading, it  is able to give graphical and spoken feedback, as well as provide help when needed.  The tutor in a computer lab was used daily by every child for ±30 minutes, during a period of 9 weeks.  Control: no intervention.  [Conn, 2014 only used the effect sizes of the endline assessment data in her meta-analysis.] | | | Identified from the systematic review of Conn, 2014.  This computer-based intervention is out of scope for this evidence summary. However, we report this study here as it is included in the meta-analysis by Conn on the effect of pedagogical methods.  An oral reading fluency test and Test of Written Spelling were conducted before the start of the study, mid-way and at the end of the study.  In the intervention-first group, carryover treatment effect may have affected the results from the second half of the study. |
| Louw, 2008, South Africa | Experimental:  Controlled before-after study | | 146 of the poorest secondary schools in the South African province of Western Cape received the intervention.  5 of these schools were randomly selected as the intervention group in this evaluation study.  5 other schools, which did not yet receive the intervention before the completion of the study, were identified and matched to the 5 intervention schools in terms of geographical location and poverty index.  The 580 sampled students (271 in the control group, 309 in the intervention group) attended grade 11 at the start of the study, 51% female (their age is not reported). | | Intervention: 40 computers per school with MasterMaths software were used to deliver the maths curriculum. This tutoring system includes 293 online teaching modules, as well as module notes and worksheets that are intended to reinforce teaching and learning. A tutor from MasterMaths oversees and coordinates students’ engagement with the system.  Control: no intervention. | | | Identified from the systematic review of Conn, 2014.  This computer-based intervention is out of scope for this evidence summary. However, we report this study here as it is included in the meta-analysis by Conn on the effect of pedagogical methods.  Marks in mathematics were obtained directly from the schools (before the intervention) or were downloaded  from the WCED database (after the intervention). |
| Lucas, 2014, USA | Experimental: Cluster randomized controlled trial | | Kenya: 112 public primary schools were randomly assigned to the intervention (57 schools, 3574 students) or control group (55 schools, 3441 students).  Uganda: 109 public primary schools were randomly assigned to the intervention (52 schools, 3275 students) or control group (57 schools, 3576 students).  All students in both Kenya and Uganda attended grade 1-3 (their age is not reported). | | Intervention: Reading to Learn (RTL) intervention, consisting of different elements:  1) 12-day teacher and head-teacher training in the use of a specific 5-step instructional method for reading instruction, and local-language materials, management of large classes and learning assessment.  2) Provision of reading and numeracy learning materials, including mini-libraries, book corners and lockable storage facilities.  3) Encouragement of school management committees to prioritize lower primary literacy.  4) Regular in-class monitoring and mentoring of teachers during school visits and periodic meetings with peer teachers.  Control: no intervention. | | | Identified from the systematic review of Conn, 2014.  To reduce data collection costs, subsampling was performed.  Exams in numeracy, written literacy and oral literacy and surveys were conducted at baseline and after  ± 22 months.  This article also reports data on attendance rates. |
| Nwagbo, 2006, Nigeria | Experimental:  Non-randomized controlled trial | | 8 classes, randomly selected from 4 senior secondary schools in the Nigerian area of Nsukka, were assigned to the intervention (4 classes) or control group (4 classes).  Students (147 in total) were senior secondary 2 biology students, attending grade 10 (their age is not reported). | | Intervention: guided inquiry method, a student-centered, activity-oriented teaching strategy, in which the teacher uses varieties of instructional materials and probing questions, to enable students discover answers to the problems at hand.  Students were taught by their regular biology teachers, who first received 8 hours of training (2 hours, 4 weeks), including going over the lesson plans and illustration of various instructional materials.  More specifically, students were grouped per 5 or 6. Each group was provided with instructional materials (charts, real specimens, models etc.) needed for the lesson. The teaching featured introduction of  the topic, drawing attention to the instructional materials, use of probing questions, student questioning, and drawing of conclusions, and the teacher directing students’ inconsistencies. Each activity was followed by a class discussion.  Control: expository method, a teacher-centered method of teaching in which the teacher delivers pre-planned lesson to the students with little or no instructional aids.  Students were taught by their regular biology teachers, who first received 8 hours of training (2 hours, 4 weeks), including going over the lesson plans.  Interaction between the students and the teacher was minimal. The students listened and assimilated principles and procedures for the correct solutions to the problems. | | | Identified from the systematic review of Conn, 2014.  Scientific Literacy Test (SLT), Biology Achievement Test (BAT) and  Attitude to Biology Scale (ABS) were conducted before and after the intervention. |
| Piper, 2009, USA | Experimental:  Cluster randomized controlled trial | | 10 intervention and 5 control schools were sampled from 3 South African provinces of North West, Limpopo and Mpumalanga.  In each school, all the grade 1 classes were included. Assignment to the intervention or control group was done randomly.  Students (650 in total) attended grade 1 and were 6 years old on average. | | Intervention: Systematic Method for Reading Success (SMRS) program, consisting of 45 lessons over a period of 6 months, in which learners are systematically introduced to letter sounds, blending sounds into words, recognizing sight words, learning vocabulary and  comprehension skills through teacher read-alouds, then reading words in decodable  and predictable stories. Because of several issues, most teachers only finished 21 of the 45 lessons.  Control: no intervention. | | | Identified from the systematic review of Conn, 2014.  4 sub-tasks of the Early Grade Reading Assessment (EGRA) were assessed (letter sound recognition,  word recognition, reading a simple passage and answering comprehension questions  about the passage) before and after the intervention  (± 6 months). |
| Piper, 2011, USA | Experimental:  Cluster randomized controlled trial | | 180 primary schools in Liberia were randomly assigned to either of 3 groups:  1) Full intervention (60 schools)  2) Light intervention  (60 schools)  3) Control  (60 schools)  Students (2988 at baseline) attended grade 2-3 (their age is not reported). | | Full intervention: Early Grade Reading Assessment (EGRA) Plus full intervention, consisting of the following components:  1) Teacher training on reading instructional strategies and continuous assessment of student performance.  2) Frequent school-based support: continuous assessment and coaching of teachers by coaches.  3) Provision of resource materials (pocket charts, teacher manual, including tightly scripted daily lesson plans in the second year of the intervention) and books.  4) Informing parents and communities about student performance.  Light intervention:  1) Teacher training on the development of student reading report cards, which they issued 4 times per year.  2) Informing parents and communities about student performance.  Control: no intervention.  [Conn 2014 only used the effect size of the endline assessment data in her meta-analysis.] | | | Identified from the systematic review of Conn, 2014.  The assessment was done on 10-20 randomly selected students in each school.  Essential early grade reading tasks were assessed at baseline, midterm (after ± 6 months) and endline (after ± 15-17 months).  This report contains tips for effective implementation of learning resources and teacher training (p. 15), including:  - Choose a realistic number of lesson plans.  - Lessons need to be tightly scripted.  - The teacher manual needs to be in one book and needs to be durable. If too large, split it into two volumes, one for each semester.  - Providing teachers with lots of options often seems good to donors, but can actually be crippling.  - One week of teacher training really is not enough, especially when teachers completely lack skills. |
| Sailors, 2010, USA | Experimental:  Non-randomized controlled trial | | Rural schools with the sparsest of resources and educational materials in South Africa were selected for the intervention group.  Officials selected control schools that closely mirrored the demographics of the intervention schools.  Students attended grade 1-2.  Students in grade 1, 793 in total, were aged on average 7 years.  Students in grade 2, 809 in total, were aged on average 8 years. | | Intervention: The Home Language Initiative, consisting of the following components:  (1) Teachers received books:  - 10 high-quality picture books that represented the lives and experiences of South African children, either in the home languages (grade 1) or English (grade 2).  - 8 wordless oversized picture books, for instruction in either language.  - 10 developmentally appropriate mother-tongue leveled readers to be placed in the hands of the children in the classroom.  - Grade 1: 20 Sunshine Starter home-language books (based on language of school), 20 Sunshine Big Books (written in English), and 120 “little books” (6 of each of the Big Book titles). Grade 2: 40 Sunshine Starter English books, 40 Big Books (English), and 240 “little books” (6 of each of the Big Book  titles, all in English).  (2) Teachers received a teacher’s guide to support their use of the various books.  (3) Each student received workbooks that provided them with opportunities to practice the reading skills taught in each of the leveled readers.  (4) Intensive and systematic professional development for teachers:  - Teachers’ curriculum, framed in a 10-day teaching cycle, was written to closely align with the national curriculum.  - Monthly coaching visits by mentors to the teachers to ensure maximum teachers support.  - Demonstration lessons by the mentors, after-school workshops, and one-on-one teacher reflections in response to the monitoring visits by the mentors.  - Workshops and conversations with principals and school administrators.  Control: no intervention. | | | Identified from the systematic review of Conn, 2014.  No information on the number of schools or on the assignment procedure to the intervention and control groups.  A random sample of 20 first and second graders was selected and tested at each of the school sites for assessment.  The Home-Language/English-as-a–Second-Language Assessment was conducted after the intervention (no pre-test data). |
| Spratt, 2013, USA | Experimental: Cluster randomized controlled trial | | 100 public or community urban or rural primary schools in 4 different language regions of Mali were stratified into 2 language groups: Bamanankan and “Other” (Bomu, Fulfulde and Songhai languages).  Schools within each language group were then randomly assigned to the intervention or control group.  Students attended grade 1-2 (their age is not reported). | | Intervention: Read-Learn-Lead program, consisting of different components:  1) Provision of student workbooks and readers, flash cards and posters, as well as teacher’s guides containing structured and systematic lessons and activities.  2) Pre-service and in-service professional development for teachers, as well as support and monitoring visits.  3) Formative assessment of children’s reading performance over time.  Control: no intervention.  [Conn, 2014 only used the effect sizes of the 3 year assessment data in her meta-analysis.] | | | Identified from the systematic review of Conn, 2014.  While the sampled schools in the study remained constant across the years of the study, the  individual staff surveyed and students assessed were not traced longitudinally.  Teacher and student surveys, as well as reading and language assessments were done at baseline, after 1 year, 2 years and 3 years. |
| Van Staden, 2011, South Africa | Experimental: Randomized controlled trial | | 24 urban and rural schools from the South African Free State Province were randomly selected.  A total of 24 postgraduates (92% female) in support teaching were recruited to participate as support teachers and thoroughly trained. Each postgraduate identified and assessed 12 low-performing readers from his/her school.  Students were randomly assigned to the intervention or control group.  Students (288 in total) were English second-language learners who were low-performing readers, attended grade 4-6, were aged between 10 and 14 years, 54% male. | | Intervention: small-group instruction, with 2-6 learners in a group, twice a week for 45-minute sessions for six months. The students received direct instruction based on mastering a sequence of essential reading skills, and using a variety of instructional materials and methods, including word-wall exercises, reciprocal questioning, print/picture mapping exercises, fast word-recognition games etc.  Control: continued with the specific schools’ reading curriculum. This followed a balanced literacy approach and consisted of several common features, including word study, group reading of stories, and writing activities, without explicit instruction. | | | Identified from the systematic review of Conn, 2014.  UCT reading tests (standardized instrument to assess sight words, one-minute speed reading, word identification and spelling) and diagnostic tests evaluating children’s level of syntactic awareness and reading comprehension were performed before and after the intervention. |
| Wachanga, 2004, Kenya | Experimental: Cluster randomized controlled trial | | A stratified random sample of 12 secondary schools (4 boys’, 4 girls’ and 4 co-educational schools) was drawn from the Kenyan Nakuru district.  The 4 schools per category were randomly assigned to the intervention (2 schools) or control group (2 schools).  Students (521 in total) attended grade 9 (“were of approximately the same age”; their age is not reported). | | Intervention: cooperative teaching method for chemistry during a period of 5 weeks.  Before the start of the intervention, students were trained by their own teachers on cooperative learning for 2 months. Next, each week during the five-week intervention period had one lesson of 80 minutes in which students performed experiments and one of 40 minutes in which they discussed the topic or performed additional experiments.  Control: students were taught through regular methods, with the teacher demonstrating the experiments. | | | Identified from the systematic review of Conn, 2014.  The Chemistry Achievement Test (CAT) was conducted before (for one control and one intervention school) and after the intervention (for all 4 schools). |
| **STRUCTURED PEDAGOGY INTERVENTIONS = development of structured lesson content and providing teacher training in delivering this, often in combination with instructional materials for students and teachers** | | | | | | | | |
| Abeberese, 2011, USA | | Experimental: Cluster randomized controlled trial | | 100 public primary schools in the Tarlac province of the Philippines were randomly assigned to the intervention or control group.  Students (5510 at baseline) attended grade 4 and were 9 years on average. | | Intervention: the Sa Aklat Sisikat (“Books make you cool”) program, consisting of the following components:  1) Provision of a set of 60 age-appropriate storybooks for every 4^th^ grade classroom at participating schools. Books were provided in both English and Filipino. Schools were allowed to keep the books after the intervention period.  2) A 2-day training session for teachers on how to implement the 31-day reading marathon, also providing ideas for reading lessons that are interactive and engaging.  3) After the 2-day training, the teachers start the 31-day reading marathon, during which students and teachers use the donated books as much as possible. Students are encouraged to read as many of the 60 storybooks as possible.  4) Teachers are monitored to ensure compliance and to answer any of their questions.  Control: no intervention.  [Snilstveit, 2015 only used the effect size of the 7 months assessment data in their meta-analyses, because the majority of the effect sizes applied a follow-up period of ±12 months.] | Identified from the systematic review of Snilstveit, 2015.  A survey to assess children’s reading skills and math skills was conducted at baseline and after 4 and 7 months. | |
| Berlinski, 2013, USA | | Experimental: Cluster randomized controlled trial | | 85 public secondary schools in the urban and semi-rural areas of Costa Rica were randomized into 10 bins of 5 schools and 5 bins of 7 schools. In every bin, schools were assigned to either one of the following conditions:  (1) New curriculum (20 schools)  (2) New curriculum + interactive whiteboard (15 schools)  (3) New curriculum + computer lab (15 schools)  (4) New curriculum + 1 computer per student (15 schools)  (5) Control (20 schools).  The total sample consisted of nearly 18 000 students attending grade 7, aged on average 13 years, 48% female. | | New curriculum intervention: intervention aimed at changing the pedagogical approach in mathematics teaching, consisting of the following components:  (1) Design of pedagogical materials for teaching geometry (worth 3 months of teaching), with an emphasis of moving away from lecture-teaching styles towards exploration, formalization and practice of concepts:  - Teachers received a structured manual and CD with 19 thematic sessions covering all the materials in the 7^th^ grade curriculum of Costa Rica, including advice on how to proceed and motivate students at different points in the class. - Students received a workbook with hands-on paper based activities, identical in knowledge content to the teacher manual.  (2) Teachers were trained for 40 hours (5 hours on-site and 5 hours in a virtual classroom, for 4 weeks) with the following objectives in mind: - immersing them into the new pedagogical approach - familiarizing them with how to use the teachers’ manual and student workbook.  At the beginning of the training, every teacher received a laptop.  New curriculum + interactive whiteboard intervention: same intervention as above, but in addition, classrooms were equipped with one interactive whiteboard, one desktop, one router and open-source mathematics software (GeoGebra). During teacher training, teachers were taught how to use the technology.  Control: no intervention.  [Snilstveit, 2015 only extracted data on the new curriculum group, the new curriculum + interactive whiteboard group and the control group.] | Identified from the systematic review of Snilstveit, 2015.  Because of cost considerations, it was unfeasible to collect data on all students. On average, each teacher was in charge of 3 classrooms. One classroom per teacher was randomly selected, rendering a total sample population of 4830 students.  At baseline, a student survey and a standardized achievement test was conducted.  After the intervention, a geometry test was conducted.  During the intervention, teacher logs and class observations were also collected.  This article also reports data on teacher performance rates. | |
| Dixon, 2011, UK | | Experimental: Cluster randomized controlled trial | | 20 private unaided English medium schools in notified slum areas of the Indian city of Hyderabad were randomly assigned to the intervention group (14 schools, 265 students) or control group, which would receive the intervention after completion of the trial (6 schools, 241 students).  Students attended grade 1 and were 7 years on average. | | Intervention: synthetic phonics intervention aimed to improve reading and spelling, consisting of the following components:  1) Provision of “Jolly Phonics” materials, including worksheets, flash cards, blending cards, storybooks and reading books.  2) Lesson plans and a sequential lesson pattern, including instructions on how to use the set of materials.  A 1-hr long synthetics phonics class designed around the materials was given every weekday for 6 months by a peripatetic teacher trained by the  researchers.  Control: no intervention. | Identified from the systematic review of Snilstveit, 2015.  Where the class was large in the intervention group, 30 children were randomly selected from the class list to experience the Jolly Phonics lessons.  Reading, spelling, letter recognition, sound values of letters and dictation were assessed before and after the intervention (6 months after the start of the intervention). | |
| He, 2007, USA | | Experimental: Cluster randomized controlled trial | | Year 1:  97 urban primary schools in the Indian district of Thane were randomly assigned to one of 2 groups:  (1) PicTalk class in grade 2, but not in grade 3.  (2) PicTalk class in grade 3, but not in grade 2.  Students (5317 in total) attended grade 2-3 and were aged about 6-9 years (7 years on average), 51% male. | | Intervention: Pratham PicTalk program, consisting of the following components:  1) Provision of 10 PicTalk Machines per school, 200 books for the machines, 50 rechargeable batteries, 2 battery chargers and 80 cartridges.  The PicTalk machine allows children to point to pictures with a stylus and hear the word pronounced aloud. Additionally, there are touch points to receive instructions and quiz questions that ask the children to identify words by pointing (giving auditory feedback).  2) Provision of a set of interactive activities designed around 440 flashcards, including chants, poems, audio tapes, games.  3) Pratham hired and trained 68 teaching assistants to implement the program. The assistants attended weekly training sessions for feedback and to prepare the materials for the week ahead. Ten monitors were assigned to supervise their  attendance.  The assistants rotated assignments, attending a new school each week. Each school received 3 days of Machine instruction and 3 days of activities instruction per week. The 2 components were alternated each day.  Control: no intervention in that same grade. | Identified from the systematic review of Snilstveit, 2015.  English knowledge was assessed at baseline and follow-up (± 12 months after the start of the intervention).  This article also reports data on attendance rates. | |
|  |  |  |  | Year 2:  242 rural government primary schools in the Indian Mangaon sub-district were randomly assigned to either of 4 groups:  (1) PicTalk Machine classes only  (2) Activities classes only  (3) PicTalk Machine + activities classes  (4) Control.  Students (9944 in total) attended grade 1-5 and were 8 years on average, 52% male. | | PicTalk Machine classes only:  1) School teacher training for 5 days on how to implement the PicTalk program.  2) Provision of PicTalk Machines.  3) Regular access to  Pratham monitors who circulated amongst the schools on a regular basis to assist teachers  as questions arose.  Activities classes only:  1) School teacher training for 5 days on how to implement the PicTalk program.  2) Provision of a set of interactive activities designed around 440 flashcards.  3) Regular access to  Pratham monitors who circulated amongst the schools on a regular basis to assist teachers  as questions arose.  PicTalk Machine + activities classes:  1) School teacher training for 5 days on how to implement the PicTalk program (both Machines and activities).  2) Provision of PicTalk Machines and a set of interactive activities.  3) Regular access to  Pratham monitors who circulated amongst the schools on a regular basis to assist teachers  as questions arose.  Control: no intervention.  [Snilstveit, 2015 only used the effect size of the PicTalk Machine+activities classes group in their meta-analysis on composite test scores, because this treatment arm was most comparable to the other studies. For their meta-analyses on language arts and maths test scores, all treatment arms were included and robust variance estimation was used.] |  |  |
| He, 2009, USA | | Experimental:  Cluster randomized controlled trial | | 67 public urban primary schools in the Indian city of Mumbai were randomly assigned to either of 3 groups:  (1) In-school treatment group (23 schools)  (2) Out-of-school treatment group (24 schools)  (3) Control group (20 schools)  Students (2679 in total) attended grade 1 (their age is not reported). | | In-school treatment:  Pratham Shishuvachan reading program over a period of 6 weeks, consisting of the following components:  1) Provision of a child library.  2) Provision of a precisely scheduled curriculum with specific activities (pre-reading, story-telling, story-reading, word recognition, letter recognition, using charts, unfamiliar text reading) conducted at specified periods.  3) Teachers were trained and supervised twice a week, whereas the supervisors met with four zonal heads every 10 days, to ensure consistency in training and implementation.  Out-of-school treatment:  Shishuvachan program was implemented on an out-of-school basis in the community.  Control: no intervention.  [Snilstveit, 2015 only extracted data on the in-school treatment group and the control group.] | Identified from the systematic review of Snilstveit, 2015.  Reading skills were assessed before and after the intervention (6 weeks later). | |
| Irwing, 2008, UK | | Experimental: Controlled before-after study | | 58 classes from 16 primary schools in the rural state of Khartoum in Sudan were divided into the intervention (31 classes) or control group (27 classes).  Students (3185 in total) were aged 7-11 years (average age 9 years), 51% male. | | Intervention: intensive Abacus program training (training in mental arithmetic using the abacus instrument) by trained teachers during 2 hours per week for 34 weeks.  Control: no intervention. | Identified from the systematic review of Snilstveit, 2015.  Cognitive skills (IQ) were tested using the standard progressive matrices (SPM) test before and after the intervention (after 34 weeks, thus ± 8 months). | |
| Jukes, 2015, USA | | Experimental: Cluster randomized controlled trial | | 101 public primary schools in the rural Kenyan districts of Kwale and Msambweni were randomly assigned to the intervention (51 schools) or control group (50 schools).  Students (2539 in total, 30 students per school) attended grade 1 and were aged 5-15 years. | | Intervention: literacy intervention, consisting of the following 3 components:  1) 140 sequential, semiscripted lesson plans for literacy sessions, each one in either Swahili or English, which were given to all participating teachers.  2) Teacher training, including a 3-day initial workshop that included guided opportunities to  create new instructional materials, a problem-solving workshop four months after the  commencement of the school year, and a refresher training the following school year.  3) Ongoing support for teachers for two years through weekly text messages providing  brief instructional tips and motivation to implement lesson plans. Teachers also received credit of $0.50 each week for their mobile phones.  Control: no intervention.  [It is unclear which effect sizes (9 months or 24 months) were used by Snilstveit, 2015 in their meta-analyses.] | Identified from the systematic review of Snilstveit, 2015.  This literacy intervention was evaluated together with a program of screening and treatment of malaria. This article only discusses the effect of the literacy intervention.  Children’s literacy, numeracy and cognitive skills (sustained attention and reasoning) were assessed at baseline, after 9 months and after 24 months. | |
| Kerwin, 2015, USA | | Experimental: Cluster randomized controlled trial | | 38 public primary schools in Uganda were randomly assigned to either of 3 groups:  1) Mango Tree-administered program schools (12 schools)  2) Government-administered program schools (14 schools)  3) Control (12 schools).  Students (1900 in total) attended grades 1-2 and were aged 7 years on average. | | Mango Tree-administered program: primary literacy-promotion program called the Northern Uganda Literacy Project (NULP), developed by Mango Tree Educational Enterprises Uganda.  In the program:  1) Children are instructed in their native language.  2) Teachers are extensively trained and supported. The first training involves a 5-day workshop on Leblango orthography. They also undergo 3 additional intensive trainings on literacy methods during school holidays. They also participate in 6 Saturday in-service training workshops throughout the school year.  3) Teaching materials are provided: primers and readers, slates that allow students to practice writing individually.  4) Content is introduced slowly, providing time for repetition and revision.  5) Engaging with parents and the local community is stimulated through parent meetings and trainings.  Government-administered program: reduced-cost version of the program that was implemented through the government and designed to simulate how the program could be implemented at scale.  In this program, there is no provision of slates. Moreover, the intensity and cost of the teacher training and support is reduced.  Control: no intervention. | Identified from the systematic review of Snilstveit, 2015.  Exams (Early Grade Reading Assessment, Oral English Test and Writing Test) were conducted at baseline and at endline (after ± 11 months).  This article also reports data on attendance, enrolment, teacher attendance and teacher performance rates. | |
| Leme, 2012, Brazil | | Experimental: Controlled before-after study | | 393 municipalities with municipal primary schools in the Brazilian state of São Paolo:  59 intervention municipalities and 332 control municipalities.  Students attended grade 4 and 8 (their age is not reported). | | Intervention: in the late 1990s, a variety of “Structured methods” were started, typically encompassing:  1) Curricular design.  2) Provision of learning materials (textbooks,  instructor material, test banks, etc.) intended for students and teachers.  3) Regular teachers instruction and pedagogic advice services (bimonthly or 6-monthly meetings) and monitoring.  4) Teacher (and sometimes student) access to interactive websites with supplemental activities, texts, documents and education-related articles and test-question banks.  Control: no intervention.  [It is unclear which effect sizes (5 or 7 years) were used by Snilstveit, 2015 in their meta-analysis.] | Identified from the systematic review of Snilstveit, 2015.  Mathematics and Portuguese language scores were assessed in 2005 and 2007 (at least 5 and 7 years).  This article also reports data on completion rates. | |
| Lucas, 2014, USA | | Characteristics: see above (“Use of alternative pedagogical methods”). | | | | | Identified from the systematic review of Snilstveit, 2015. | |
| Mouton, 1995, South Africa | | Experimental: Cluster randomized controlled trial | | 54 teachers were randomly allocated to intervention or control conditions in 48 public primary schools (32 intervention schools, 16 control schools) in 2 urban and 2 rural circuits of the Kwazulu region of South Africa.  Students (2200 in total) attended grade 3 (their age is not reported). | | Intervention: the English and Operacy Program (EOP), consisting of the following components:  1) Teacher training of 3 weeks in the English language, thinking skills and the methodology of Suggestopedia, which is based on the following principles: - learning should be a fun and fearless activity; - both the conscious and the subconscious should be used simultaneously; - a suggestive link between the teacher and the students (de-emphasis of errors, absence of destructive criticism, positive and supportive attitude…). A typical training cycle consists of the following steps: (1) Physical and mental relaxation (2) Activation of previously taught material, using drama, poems, songs, games and stories (3) Presenting of new language material (4) No homework 2) Half of the teachers received visits to provide support and motivate them.  3) Monitoring occurred through interviews with teachers and principals as well as classroom observation.  Control: teachers received training in how to use interactive group techniques when giving classes (small intervention designed to negate any Hawthorne-like effect).  [Snilstveit, 2015 only used the effect size of the November assessment data in their meta-analyses, because the majority of the effect sizes applied a follow-up period of ±12 months.] | Identified from the systematic review of Snilstveit, 2015.  Students were pretested at the beginning of the academic year in January on 4 psychometric test (English, Mathematics, Pattern completion and Number series) and a self-concept questionnaire. These were repeated in June (+ biographical questionnaire) and November (+ exam scores on English, Mathematics and Social Studies).  This article also reports data on teacher performance rates. | |
| Nonoyama-Tarumi, 2009, USA | | Experimental: Controlled before-after study | | 10 rural and urban primary schools in 3 provinces of Cambodia that participated in the School Readiness Program (SRP) were randomly chosen.  Another 10 schools, where the intervention had not occurred, were assigned to the control group and matched to SRP schools according to demography and poverty ratings.  Students (931 in total at baseline, 473 in intervention group and 458 in control group) attended grade 1 (their age is not reported). | | Intervention: the School Readiness Program (SRP) of the government was implemented to tackle the high grade repetition in the first grades of primary school. It consisted of the following components:  1) Development of a specialized bridging curriculum that prepares children for eventual exposure to the formal curriculum. This includes the areas of basic language skills, concept of number, time and space, working in groups, hygiene… and focuses on fine and gross motor skills (instead of the traditional academic curriculum, which focuses on verbal and visual abstractions).  2) A 14-day teacher training program concerning the use of the special curriculum, as well as the need for changes in classroom practice. For example, teachers acquired a repertoire of  numerous activities involving songs, role plays, drawing, games,  and other activities for teaching designated lessons in a more engaging way and to rely less on textbooks.  3) Regular monitoring to support implementation by teachers.  4) Physical upgrading of classrooms. This usually included the provision of  copious amounts of stationery and raw materials for the production of teaching aids as well as decorations to make classroom environments more interesting.  5) Formalized student assessment for monitoring purposes.  Control: no intervention.  [Snilstveit, 2015 only used the effect size of the 10 months assessment data in their meta-analyses, because the majority of the effect sizes applied a follow-up period of ±12 months.] | Identified from the systematic review of Snilstveit, 2015.  Khmer language skills were assessed through interviewing at baseline, at the end of the intervention (after 8 weeks) and at the end of the school year (± 10 months after the start of the intervention). | |
| Pallante, 2013, USA | | Experimental: Controlled before-after study | | Teachers were randomly assigned to either intervention or control conditions in 10 classes of 5 private primary schools in the urban area of Santiago in Chile (5 control classes, 5 intervention classes).  Students (305 in total) attended grade 1, were aged on average 7 years and 55% female.  [This article also reports the effect of the same intervention in kindergarteners (n=312, average age 6 years). These data were not extracted by Snilstveit, 2015.] | | Intervention: Collaborative Language and Literacy Instruction Project (CLLIP), consisting of 2 different components:  1) Teachers attended 4 professional development training modules throughout the year. Each training included the following elements: theoretical basis for content being learned, materials needed to provide students with best practice instruction,  and graphic organizers to help participants plan  for instruction to be taught immediately.  In addition, they were trained to identify children at risk for reading and writing difficulties, and to  provide small group instruction interventions.  2) Teachers were coached on-site after every training module at the local school.  Control: no intervention.  [Snilstveit, 2015 only used the effect size of the 8 months assessment data in their meta-analyses, because the majority of the effect sizes applied a follow-up period of ±12 months.] | Identified from the systematic review of Snilstveit, 2015.  Language skills were assessed at the beginning (late March), middle (late June) and end of the academic year (October to mid-November; ± 8 months after the start of the intervention). | |
| Piper, 2011, USA | | Characteristics: see above (“Use of alternative pedagogical methods”).  [Snilstveit 2015 only extracted data on the full intervention group and control group. Like Conn 2014, they only used the effect size of the endline assessment data in their meta-analyses, because the majority of the effect sizes applied a follow-up period of ±12 months.] | | | | | Identified from the systematic review of Snilstveit, 2015. | |
| Piper, 2014, USA | | Experimental: Cluster randomized controlled trial | | 411 public and low-cost private primary schools in the Kenyan counties of Nairobi, Murang’a, Kiambu and Nakuru were randomly selected and randomly assigned to the intervention (310 schools) or control group (101 schools, which received the intervention after the endline assessment of the program).  Students attended grades 1-2 (their age is not reported). | | Intervention: Primary Mathematics and Reading (PRIMR) Initiative, consisting of the following components:  (1) Provision of inexpensive books for pupils at a 1:1 ratio (for English and Kiswahili: attractive illustrations in full colour, phonics activities and decodable stories).  (2) Provision of simple instructional aids, including an A3-sized pocket chart and a set of letter and numeral flashcards.  (3) Provision of a teachers’ guide for Kiswahili, English and mathematics and an assessment manual, supplementary readers, a sheet of training tips and a two-page document to track pupil progress.  (4) 10 days of teacher training in the subjects of English, Kiswahili and maths.  (5) Regular follow-up and observation of teachers by tutors and instructional coaches.  (6) Open-to-the-public reading and maths contests.  (7) Teachers’ monthly reflection meetings.  Control: no intervention.  [Snilstveit, 2015 did not report effect sizes relating to language arts, because of extreme variation in the effects, which could not be clarified by the authors of the study.  For maths, it is unclear which effect sizes (9 months or 21 months) they used in their meta-analyses.] | Identified from the systematic review of Snilstveit, 2015.  A total of 4,385 pupils randomly  selected from 230 schools were assessed.  Early Grade Reading Assessment (EGRA) and the Early Grade  Mathematics Assessment (EGMA) were conducted at baseline, midterm (after 9 months) and at endline (after 21 months). | |
| RTI International, 2015, USA | | Experimental: Cluster randomized controlled trial | | 834 public primary schools in the rural Bungoma and Machakos counties of Kenya were randomly assigned to either of 5 groups:  1) Full Primary Math and Reading treatment (PRIMR) (162 schools)  2) Books & training treatment (156 schools)  3) Training only treatment (173 schools)  4) Mother tongue + Full PRIMR treatment (166 schools)  5) Control group (177 schools), which received the full PRIMR intervention after the endline assessment of the program.  Students (up to 4566 in the endline sample) attended grade 1 and 2 (their age is not reported). | | Books & training intervention: consisted of different components:  1) Provision of low-cost books for students, in a 1:1 student-book ratio, covering English, Kiswahili, maths and mother tongue.  The books had attractive illustrations in full colour.  2) Provision of basic instructional aids, including an A3-sized pocket chart with 3 pockets, a set of laminated letter flashcards in business-card size, and a set of number flashcards laminated in business-card size.  3) 10 days of teacher training during the entire year, where they learned to implement lessons and plan lessons.  4) Regular supervision and classroom monitoring of teachers.  5) Teacher training to ensure continuous assessment.  6) Training, monitoring and coaching of teachers by instructional coaches.  7) Termly reading and maths exhibitions, inviting parents and community members to visit the schools.  8) Monthly reflection meetings for teachers.  However, there was no provision of teachers’ guides.  Control: no intervention.  [Snilstveit, 2015 only extracted data on the books & training intervention group and control group.  It is unclear which effect sizes (7 months or 18 months) they used in their meta-analyses.] | Identified from the systematic review of Snilstveit, 2015.  171 (± 40%) of schools were sampled at baseline. To reduce the cost of data collection at midterm and endline, 230 schools were sampled.  Language and maths were assessed after 7 (midterm) and 18 months (endline). | |
| San Antonio, 2011 | | Experimental: Cluster randomized controlled trial | | 50 mathematics teachers from 49 urban public primary schools in the Bicol Region of the Philippines were matched based on their commitment-level scores and these pairs were randomly assigned to the intervention (25 teachers) or control group (25 teachers).  Students attended grade 6 (their age is not reported). | | Intervention:  (1) Teachers attended an orientation workshop on the proper way of using printed ‘modules’ (self-contained instructional packages about a single unit of mathematics) to enhance their professional competence.  (2) Next, they proceeded with their usual classroom teaching duties, while also studying the modules distributed to them every week.  (3) Teachers received follow-ups from the school heads and supervisors.  Control: teachers attended a separate seminar on the topics covered in the modules used by the intervention group (small intervention designed to negate any Hawthorne-like effect). Next, they proceeded with their usual classroom teaching duties. | Identified from the systematic review of Snilstveit, 2015.  Mathematics proficiency was tested after 5 weeks of implementing the intervention (no pre-test was conducted).  This article also reports data on teacher performance rates. | |
| Spratt, 2013, USA | | Characteristics: see above (“Use of alternative pedagogical methods”).  [Unlike Conn 2014, Snilstveit 2015 only used the effect size of the 1 year assessment data in their meta-analyses, because the majority of the effect sizes applied a follow-up period of ±12 months.] | | | | | Identified from the systematic review of Snilstveit, 2015. | |
| Tan, 1999, USA | | Experimental:  Cluster randomized controlled trial | | 29 primary schools located in 10 rural provinces of the Philippines were randomly assigned to either of 5 groups:  1) School feeding alone (5 schools, 751 students)  2) School feeding with parent-teacher partnership (5 schools, 858 students)  3) Multi-level learning materials alone (5 schools, 673 students)  4) Multi-level learning materials with parent-teacher partnership (5 schools, 629 students)  5) Control (9 schools, 1356 students).  Students attended grade 1-5 (their age is not reported). | | Multi-level learning materials intervention: teachers received pedagogical materials designed to help them pace their teaching according to the differing abilities of their students. Prior to implementation, teachers attended a week-long training course on the use of the materials.  Control: no intervention.  [Snilstveit, 2015 only extracted data on the multi-level learning materials alone group and control group.] | Identified from the systematic review of Snilstveit, 2015.  Achievement tests (grade-specific tests in mathematics, English and Filippino) were conducted at the beginning and the end of the school year.  This article also reports data on drop-out rates. | |

## Synthesis of findings

| **Outcome** | **Comparison** | **Effect Size** | **#effect sizes, # studies** | | **Reference** |
| --- | --- | --- | --- | --- | --- |
| **PROVISION OF INSTRUCTIONAL MATERIALS** | | | | | |
| Composite test scores (standardised mean difference (SMD)±standard error (SE)) | Provision of instructional materials  vs no intervention (business as usual) | Not statistically significant:  SMD: 0.01±0.01, 95%CI  [-0.01;0.02] (p=0.23) † | 5 effect sizes from  3 studies ††  (Das 2013,  Glewwe 2004,  Glewwe 2009) | | Snilstveit, 2015 |
| Language arts test scores (SMD±SE) |  | Not statistically significant:  SMD: 0.00±0.01, 95%CI  [-0.02;0.02] (p=0.78) † | 5 effect sizes from  4 studies ††  (Das 2013,  Glewwe 2004,  Glewwe 2009,  Sabarwal 2009) | |  |
| Maths test scores (SMD±SE) |  | Not statistically significant:  SMD: -0.02±0.02, 95%CI  [-0.06;0.02] (p=0.26) † | 5 effect sizes from  4 studies ††  (Das 2013,  Glewwe 2004,  Glewwe 2009,  Sabarwal 2009) | |  |
| **USE OF ALTERNATIVE PEDAGOGICAL METHODS** | | | | | |
| Learning/testing outcomes (Cohen’s *d* ± SE) | Use of alternative pedagogical methods  vs  conventional teaching methods | Statistically significant:  Cohen’s *d*: 0.918±0.314, 95%CI [0.25;1.59] (p<0.05) | 41 effect sizes from  17 studies ††  (Abdu-Raheem 2012,  Ajaja 2010,  Bimbola 2010,  Brooker 2013,  Githua 2008,  Kiboss 2012,  Korsah 2010,  Louw 2008,  Lucas 2014 Kenya,  Lucas 2014 Uganda,  Nwagbo 2006,  Piper 2009,  Piper 2011,  Sailors 2010,  Spratt 2013,  Van Staden 2011, Wachanga 2004) | | Conn, 2014 |
| **STRUCTURED PEDAGOGY INTERVENTIONS** | | | | | |
| Cognitive test scores (SMD±SE) | Structured pedagogy interventions vs no intervention or other small educational intervention | Not statistically significant:  SMD: 0.01±0.03, 95%CI  [-0.04;0.07] (p=0.66) † | 2 effect sizes from  2 studies ††  (Irwing 2008,  Jukes 2015) | Snilstveit, 2015 | |
| Composite test scores (SMD±SE) |  | Statistically significant:  SMD: 0.06±0.01, 95%CI [0.03;0.08] (p<0.0001) | 3 effect sizes from  3 studies ††  (He 2007 year 1,  He 2007 year 2,  Tan 1999) |  |  |
|  |  | *Grades 1-3 sub-group:*  Statistically significant:  SMD: 0.09±0.02, 95%CI [0.05;0.13] (p<0.0001) | 2 effect sizes from  2 studies ††  (He 2007 year 1,  He 2007 year 2) |  |  |
|  |  | *Grades 4-5 sub-group:*  Statistically significant:  SMD: 0.08±0.02, 95%CI [0.04;0.12] (p<0.0001) | 2 effect sizes from  2 studies ††  (He 2007 year 1,  He 2007 year 2) |  |  |
| Language arts test scores (SMD±SE) |  | Statistically significant:  SMD: 0.23±0.05, 95%CI [0.13;0.34] (p<0.001) | 67 effect sizes from  17 studies ††  (Abeberese 2011,  Dixon 2011,  He 2007 year 1,  He 2007 year 2,  He 2009,  Jukes 2015,  Kerwin 2015,  Leme 2010,  Lucas 2014 Kenya,  Lucas 2014 Uganda, Mouton 1995, Nonoyama-Tarumi 2009,  Pallante 2013,  Piper 2011,  RTI International 2015,  Spratt 2013,  Tan 1999) |  |  |
|  |  | *Grades 1-3 sub-group:*  Statistically significant:  SMD: 0.23±0.06, 95%CI [0.11;0.35] (p<0.01) | 63 effect sizes from  14 studies ††  (Dixon 2011,  He 2007 year 1,  He 2007 year 2,  He 2009,  Jukes 2015,  Kerwin 2015,  Lucas 2014 Kenya,  Lucas 2014 Uganda, Nonoyama-Tarumi 2009,  Pallante 2013,  Piper 2011,  RTI International 2015,  Spratt 2013,  Tan 1999) |  |  |
|  |  | *Grades 4-6 sub-group:*  Not statistically significant:  SMD: 0.21±0.13, 95%CI  [-0.04;0.47] (p=0.10) † | 4 effect sizes from  4 studies ††  (Abeberese 2011,  He 2007 year 2,  Leme 2010,  Mouton 1995) |  |  |
| Maths test scores (SMD±SE) |  | Statistically significant:  SMD: 0.14±0.03, 95%CI [0.08;0.20] (p<0.001) | 24 effect sizes from  14 studies ††  (Abeberese 2011, Berlinski 2013,  He 2007 year 1,  He 2007 year 2,  Jukes 2015,  Lucas 2014 Kenya,  Lucas 2014 Uganda, Leme 2010,  Mouton 1995,  Piper 2011,  Piper 2014,  RTI International 2015,  San Antonio 2011,  Tan 1999) |  |  |
|  |  | *Grades 1-3 sub-group:*  Statistically significant:  SMD: 0.08±0.03, 95%CI [0.03;0.13] (p<0.01) | 9 effect sizes from  9 studies ††  (He 2007 year 1,  He 2007 year 2,  Jukes 2015,  Lucas 2014 Kenya,  Lucas 2014 Uganda, Piper 2011,  Piper 2014,  RTI International 2015,  Tan 1999) |  |  |
|  |  | *Grades 4-6 sub-group:*  Statistically significant:  SMD: 0.21±0.08, 95%CI [0.04;0.37] (p<0.05) | 4 effect sizes from  4 studies ††  (Abeberese 2011,  He 2007 year 2,  Leme 2010,  Mouton 1995) |  | |

|  |  | *Grades 7-11 sub-group:*  Not statistically significant:  SMD: 0.13±0.12, 95%CI  [-0.10;0.35] (p=0.29) † | 3 effect sizes from  3 studies ††  (Berlinski 2013,  Leme 2010,  San Antonio 2011) |  |
| --- | --- | --- | --- | --- |

SMD: standardized mean difference, SE: standard error

† Imprecision (lack of data): mean of the control group is not reported.

†† Imprecision (lack of data): total sample size is not reported.

## Certainty of the body of evidence

| **Provision of instructional materials** | **Initial grading High [A]** | Downgrading due to |
| --- | --- | --- |
| **Limitations of study design** | -1 | See SR Snilstveit 2015:  1) High risk of analysis reporting bias in Glewwe 2009.  2) High risk of performance bias in Glewwe 2009 and Sabarwal 2014.  3) 3 of the 4 included studies report issues with program implementation: - some schools did not actually receive the text books (Sabarwal 2014, Das 2013); - some schools did not spend the grants on purchasing materials, but used them for classroom construction (Glewwe 2009);  - books were kept in storage instead of being provided to the students (Sabarwal 2014);  - English text books might have been too difficult for most students (Glewwe 2009). |
| **Imprecision** | -1 | Lack of data |
| **Inconsistency** | 0 |  |
| **Indirectness** | 0 |  |
| **Publication bias** | 0 |  |
| **QUALITY (GRADE)** | **Final grading Low [C]** |  |

| **Use of alternative pedagogical methods** | **Initial grading High [A]** | Downgrading due to |
| --- | --- | --- |
| **Limitations of study design** | -1 | No clear reporting of results of quality assessment of the included studies in the SR of Conn 2014; 9 of the 17 included studies are categorized as high-quality studies (quality index ≥ 3), indicating that the other 8 studies have some methodological shortcomings. Conn was contacted to obtain more information on this matter, but did not respond to our email.  In addition, it seems as though the systematic searching and abstract/full-text screening for this SR was performed by a single reviewer (Conn herself). |
| **Imprecision** | -1 | Lack of data |
| **Inconsistency** | 0 |  |
| **Indirectness** | 0 | 3 of the 17 included studies report on the effect of computer-based interventions, which go beyond the scope of this evidence summary (Kiboss 2012, Korsah 2010, Louw 2008). |
| **Publication bias** | 0 |  |
| **QUALITY (GRADE)** | **Final grading Low [C]** |  |

| **Structured pedagogy interventions** | **Initial grading High [A]** | Downgrading due to |
| --- | --- | --- |
| **Limitations of study design** | -1 | See SR Snilstveit 2015:  1) High risk of selection bias and confounding in 4 studies (Dixon 2011, Irwing 2008, Nonoyama-Tarumi 2009, Piper 2014).  2) High risk of performance bias in 15 studies.  3) High risk of other bias in 5 studies (Tan 1999, Abeberese 2011, San Antonio 2011, Piper 2011, Piper 2014).  4) Issues with program implementation:  - teachers opposed the proposed changes, leading to teacher unions taking strike action (Piper 2011, Piper 2014 and Spratt 2013);  - failure to deliver tools and supplies (Lucas 2014, Spratt 2013, Piper 2014);  - failure to provide on-time and high-quality teacher training (RTI 2015, Mouton 1995, Spratt 2013);  - teachers were not knowledgeable or experienced enough to fully understand their training (Berlinski 2013, Dixon 2011, He 2007, He 2009, Mouton 1995);  - teachers did not implement lessons as intended (Piper 2014, Lucas 2014);  - teachers tended to keep books in school rather than let children take them home (Abeberese 2011).  5) Issues related to failure to fully take into account key contextual factors, including: - limited resources of the education systems (Dixon 2011, Tan 1999, Lucas 2014, Nonoyama-Tarumi 2009, Piper 2014, Mouton 1995); - high rates of enrolment (Lucas 2014, Nonoyama-Tarumi 2009, Piper 2014, Spratt 2013). |
| **Imprecision** | -1 | Lack of data |
| **Inconsistency** | 0 |  |
| **Indirectness** | 0 |  |
| **Publication bias** | 0 |  |
| **QUALITY (GRADE)** | **Final grading Low [C]** |  |

| **Conclusion** | **Provision of instructional materials**  There is limited evidence neither in favour of providing instructional materials nor not providing these materials:  It could not be demonstrated that provision of instructional materials (textbooks, flip-charts or grants used directly for materials) results in a statistically significant increase in composite test scores, language arts test scores or maths test scores (Snilstveit 2015).  Evidence is of low quality, mainly due to issues with implementation in the existing studies, and results are imprecise due to lack of data.  Nevertheless, structured pedagogy interventions (see below) do suggest beneficial effects of programs that include the provision of materials together with other components.  **Use of alternative pedagogical methods** There is limited evidence in favour of the use of alternative pedagogical methods:  It was shown that the use of alternative pedagogical methods (including problem-solving instruction, constructivist instruction, guided-inquiry instruction, cooperative instruction, small-group instruction) in schools of Sub-Saharan Africa resulted in a statistically significant increase in learning/testing outcomes, compared to conventional (often lecturing) teaching methods (Conn 2014).  Evidence is of low quality and results cannot be considered precise due to lack of data.  **Structured pedagogy interventions** There is limited evidence in favour of structured pedagogy interventions:  It was shown that structured pedagogy interventions (development of structured lesson content and providing teacher training in delivering this, often in combination with instructional materials for students and teachers) resulted in a statistically significant increase in composite test scores, language arts test scores and maths test scores. However, a significant change in cognitive test scores could not be demonstrated (Snilstveit 2015).  Evidence is of low quality and results cannot be considered precise due to lack of data.  Structured pedagogy interventions show a large range in effects, suggesting that intervention design, implementation factors and contextual factors can play an important role in determining how effective an intervention is. |
| --- | --- |
| **Reference(s)** | **Articles**  Abdu-Raheem BO. *Effects of problem-solving method on secondary school students’ achievement and retention in social studies, in Ekiti State, Nigeria.* Journal of International Education Research 2012, 8(1):19-26  Abeberese AB, Kumler TJ, Linden LL. *Improving reading skills by encouraging children to read: a randomized evaluation of the Sa Aklat Sisikat reading program in the Philippines.* 2011, Institute for the Study of Labor (IZA) Discussion paper No. 5812  Ajaja OP, Eravwoke OU. *Effects of cooperative learning strategy on junior secondary school students achievement in integrated science.* Electronic Journal of Science Education 2010, 14(1):1-18  Berlinski S, Busso M. *Pedagogical change in mathematics teaching: evidence from a randomized controlled trial.* 2013, unpublished working paper.  Bimbola O, Daniel OI. *Effect of constructivist-based teaching strategy on academic performance of students in integrated science at the junior secondary school level.* Educ Res Rev 2010, 5(7):347-353  Brooker S, Halliday K. *Impact of malaria control and enhanced literacy instruction on educational outcomes among school children in Kenya: a multi-sectoral, prospective, randomised evaluation.* 2013, 3ie Impact Evaluation Report 18  Das J, Dercon S, Habyarimana J, Krishnan P, Muralidharan K, Sundararaman V. *School inputs, household substitution, and test scores.* Am Econ J Appl Econ 2013, 5(2):29-57  Dixon P, Schagen I, Seedhouse P. *The impact of an intervention on children’s reading and spelling ability in low-income schools in India.* School Effectiveness and School Improvement 2011, 22(4):461-482  Githua BN, Nyabwa RA. *Effects of advance organiser strategy during instruction on secondary school students’ mathematics achievement in Kenya’s Nakuru district.* Int J Sci Math Educ 2008, 6:439-457  Glewwe P, Kremer M, Moulin S, Zitzewitz E. *Retrospective vs. prospective analyses of school inputs: the case of flip charts in Kenya*. J Dev Econ 2004, 74:251-268  Glewwe P, Kremer M, Moulin S. *Many children left behind? Textbooks and test scores in Kenya.* Am Econ J Appl Econ 2009, 1(1):112-135  He F, Linden LL, MacLeod M. *Helping teach what teachers don’t know: an assessment of the Pratham English Language Program.* 2007, unpublished working paper.  He F, Linden LL, MacLeod M. *A better way to teach children to read? Evidence from a randomized controlled trial.* 2009, unpublished manuscript.  Irwing P, Hamza A, Khaleefa O, Lynn R. *Effects of Abacus training on the intelligence of Sudanese children.* Pers Individ Dif 2008, 45:694-696  Jukes MCH, Dubeck MM. *Teacher professional development and text messages support for improved literacy instruction in Kenya: an experimental evaluation.* 2015, working paper.  Kerwin JT, Thornton R. *Making the grade: understanding what works for teaching literacy in rural Uganda.* 2015, Population Studies Center Research Report 15-842  Kiboss JK. *Effects of special e-learning program on hearing-impaired learners’ achievement and perceptions of basic geometry in lower primary mathematics.* J Educational Computing Research 2012, 46(1):31-59  Korsah GA, Mostow J, Dias MB, Sweet TM, Belousov SM, Dias MF, Gong J. *Improving child literacy in Africa: experiments with an automated reading tutor.* Information Technologies & International Development 2010, 6(2):1-19  Leme MC, Louzano P, Ponczek V, Souza AP. *The impact of structured teaching methods on the quality of education in Brazil.* Econ Edu Rev 2012, 31:850-860  Louw J, Muller J, Tredoux C. *Time-on-task, technology and mathematics achievement.* Eval Prog Plan 2008, 31:41-50  Lucas AM, McEwan PJ, Ngware M, Oketch M. *Improving early-grade literacy in East Africa: Experimental evidence from Kenya and Uganda.* J Policy Anal Manag 2014, 33(4):950-976  Mouton J. *Second language teaching for primary school students: an evaluation of a new teaching method.* Eval Program Plann 1995, 18(4):391-408  Nonoyama-Tarumi Y, Bredenberg K. *Impact of school readiness program interventions on children’s learning in Cambodia.* Int J Edu Dev 2009, 29:39-45  Nwagbo C. *Effects of two teaching methods on the achievement in and attitude to biology of students of different levels of scientific literacy.* Int J Edu Res 2006, 45:216-229  Pallante DH, Kim YS. *The effect of a multicomponent literacy instruction model on literacy growth for kindergartners and first-grade students in Chile.* Int J Psychol 2013, 48(5):747-761  Piper B. *Integrated Education Program: Impact study of SMRS using Early Grade Reading Assessment in three provinces in South Africa.* 2009, RTI International.  Piper B, Korda M. *EGRA Plus: Liberia. Program evaluation report.* 2011, RTI International.  Piper B. *The Primary Math and Reading (PRIMR) initiative: Endline impact evaluation – Revised edition.* 2014, RTI International.  RTI International. *The Primary Math and Reading (PRIMR) initiative: DFID/Kenya rural expansion programme. Bungoma and Machakos endline study.* 2015, RTI International.  Sabarwal S, Evans DK, Marshak A. *The permanent input hypothesis: the case of textbooks and (no) student learning in Sierra Leone.* 2014 World Bank Group Education Global Practice Group & Africa Region, policy research working paper 7021.  Sailors M, Hoffman JV, Pearson PD, Beretvas SN, Matthee B. *The effects of first- and second-language instruction in rural South African schools.* Biling Res J 2010, 33(1):21-41  San Antonio DM, Morales NS, Moral LS. *Module-based professional development for teachers: a cost-effective Philippine experiment.* Teacher Development 2011, 15(2):157-169  Spratt J, King S, Bulat J. *Independent evaluation of the effectiveness of Institut pour l’Education Populaire’s “Read-Learn-Lead” (RLL) program in Mali: endline report.* 2013, RTI International.  Tan JP, Lane J, Lassibille G. *Student outcomes in Philippine elementary schools: an evaluation of four experiments.* World Bank Econ Rev 1999, 13(3):493-508  Van Staden A. *Put reading first: positive effects of direct instruction and scaffolding for ESL learners struggling with reading.* Perspectives in Education 2011, 29(4):10-21  Wachanga SW, Mwangi JG. *Effects of the cooperative class experiment teaching method on secondary school students’ chemistry achievement in Kenya’s Nakuru district.* Int Edu J 2004, 5(1):26-36  **Systematic reviews**  Conn K. *Identifying effective education interventions in Sub-Saharan Africa: a meta-analysis of rigorous impact evaluations.* 2014, Columbia University PhD dissertation  Snilstveit B, Stevenson J, Phillips D, Vojtkova M, Gallagher E, Schmidt T, Jobse H, Geelen M, Pastorello MG et al. *Interventions for improving learning outcomes and access to education in low-and middle-income countries: a systematic review.* 2015, 3ie Systematic Review 24. |
