# Supplementary material for: An educational pathway and teaching materials for first aid training of children in sub-Saharan Africa based on the best available evidence
Source: BMC Public Health. 2020 Jun 3;20:836. doi: 10.1186/s12889-020-08857-5 (PMC7268765; doi:10.1186/s12889-020-08857-5)
Supplement: Supplementary file 9 — Additional file 9. Evidence-based educational pathway on first aid for sub-Saharan Africa 221 [file 12889_2020_8857_MOESM9_ESM.docx]

# Additional file 9: Educational pathway on first aid for sub-Saharan Africa based on the best available evidence

| E: Encourage  K: Know/Know How  R: Repeat | 5-6 yrs | 7-8 yrs | 9-10 yrs | 11-12 yrs | 13-14 yrs | 15-16 yrs | 17-18 yrs |
| --- | --- | --- | --- | --- | --- | --- | --- |
| General |  |  |  |  |  |  |  |
| **Knowledge** |  |  |  |  |  |  |  |
| The children know:   - why/that they must find help from an adult as quickly as possible in an emergency situation; | E | K | R | R |  |  |  |
| - how to seek help from a medical care provider; | E | E | K | R | R | R | R |
| - the six principles of first aid (but not psychosocial first aid, only ensuring comfort in a simple way). |  |  | E | K | R | R |  |
| **Skills** |  |  |  |  |  |  |  |
| The children can:  wash their hands; | K | R | R | R | R | R | R |
| - put on disposable rubber gloves or plastic bags; | E | K | R | R | R | R | R |
| - use the six principles of first aid when looking after an ill or injured person (not psychosocial first aid). |  |  | E | K | R | R | R |
| **Attitudes** |  |  |  |  |  |  |  |
| The children are prepared:   - to help; | E | K | R | R | R | R | R |
| - to comfort the ill or injured person (by covering him with a blanket, by protecting him against the sun); | E | K | R | R | R | R | R |
| - to ensure their own safety; | E | E | K | R | R |  |  |
| - to fetch an adult. | E | K | R | R | R | R | R |
| The children recognise the importance of:   - fetching an adult; | E | K | R | R | R | R | R |
| - seeking help from a medical care provider. | E | K | R | R |  |  |  |
| The children recognise the importance of:   - avoiding infection; | E | E | K | R | R | R | R |
| - ensuring the comfort of the ill or injured person. |  |  | E | K | R | R | R |
| Four main steps in first aid |  |  |  |  |  |  |  |
| **Knowledge** |  |  |  |  |  |  |  |
| The children know:   - the four main steps in first aid; |  | E | E | K | R | R | R |
| - which wound must be treated first when providing first aid; |  |  |  | K | R | R |  |
| - the importance of an open airway; |  |  | E | K | R | R | R |
| - when they should place a person in the recovery position; |  |  |  | E | K | R | R |
| - when someone is fainting or has fainted; | E | K | R | R | R | R | R |
| - what to do when someone is fainting or has fainted. | E | K | R | R | R | R | R |
| **Skills** |  |  |  |  |  |  |  |
| The children ensure the safety of:   - themselves; | E | E | K | R |  |  |  |
| - the ill or injured person and bystanders. |  | E | K | R | R |  |  |
| The children can:   - seek help from an adult in an emergency situation; | E | K | R | R |  |  |  |
| - seek help from a medical care provider correctly; |  | E | K | R | R |  |  |
| - establish whether a person is conscious or not; | E | K | R | R | R | R | R |
| - establish whether an unconscious person is breathing; |  |  | E | K | R | R | R |
| - tilt the head back and lift the chin up correctly [technique]; |  |  | E | K | R | R | R |
| - can place a person in the recovery position [technique]; |  |  | E | K | R | R | R |
| - provide further first aid. |  | E | E | E | K | R | R |
| **Attitudes** |  |  |  |  |  |  |  |
| *See educational pathway General > Attitudes* |  |  |  |  |  |  |  |
| The children:   - appreciate the importance of ensuring their own safety; | E | E | K | R | R |  |  |
| - appreciate that it is also important to ensure the safety of the ill or injured person and the bystanders; |  | E | K | R | R |  |  |
| - recognise the importance of fetching an adult; | E | K | R | R |  |  |  |
| - recognise the importance of the correct and complete application of the four main steps in first aid; |  | E | E | K | R |  |  |
| - are ready to provide further first aid where able. |  | E | E | E | K | R | R |
| Resuscitation |  |  |  |  |  |  |  |
| **Knowledge** |  |  |  |  |  |  |  |
| The children know:   - that they should perform cardiopulmonary resuscitation (CPR) on an unconscious person who is not breathing normally; |  |  | E | K | R | R |  |
| - the different aspects of CPR and know in which order they should be administered; |  |  | E | K | R | R |  |
| - how many chest compressions should be given; |  |  | E | K | R | R |  |
| - the correct chest compression depth; |  |  | E | K | R | R |  |
| - the frequency at which chest compressions should be given; |  |  | E | K | R | R |  |
| - how many rescue breaths should be administered; |  |  | E | K | R | R |  |
| - that they must always seek help from a medical care provider if a person is unconscious. |  |  | E | K | R | R |  |
| **Skills** |  |  |  |  |  |  |  |
| The children can:   - correctly perform CPR on an unconscious person who is not breathing normally; |  |  |  | E | K | R | R |
| - correctly carry out chest compressions [technique]; |  |  |  | E | K | R | R |
| - correctly administer rescue breaths [technique]. |  |  |  | E | K | R | R |
| **Attitudes** |  |  |  |  |  |  |  |
| *See educational pathway General > Attitudes* |  |  |  |  |  |  |  |
| The children:   - recognise the importance of performing CPR; |  |  |  | E | K | R | R |
| - are prepared to perform CPR. |  |  |  | E | K | R | R |
| Choking |  |  |  |  |  |  |  |
| **Knowledge** |  |  |  |  |  |  |  |
| The children know:   - the difference between mild and severe choking. |  |  |  | K | R | R |  |
| **Skills** |  |  |  |  |  |  |  |
| The children can:   - administer first aid correctly in the event of a choking incident; |  |  |  | K | R | R |  |
| - correctly give blows to the back [technique]; |  |  |  | K | R | R |  |
| - correctly give abdominal thrusts [technique]. |  |  |  | E | K | R | R |
| **Attitudes** |  |  |  |  |  |  |  |
| *See educational pathway General > Attitudes* |  |  |  |  |  |  |  |
| Skin wound |  |  |  |  |  |  |  |
| **Knowledge** | | | | | | | |
| The children:   - recognise a skin wound; | E | K | R | R |  |  |  |
| - know which equipment is needed to provide first aid for a skin wound; | E | K | R | R |  |  |  |
| - know when the injured person should seek medical help for a skin wound; |  |  |  | K | R | R | R |
| - know the importance of tetanus vaccinations, and know why tetanus is dangerous and linked with skin wounds; |  |  |  | K | R | R | R |
| - know that an injured person with a skin wound in which a foreign object is embedded should always seek medical help. |  |  |  | K | R | R | R |
| **Skills** |  |  |  |  |  |  |  |
| The children can:   - correctly provide first aid for a skin wound if clean water is available; | E | K | R | R | R |  |  |
| - stop the bleeding of a wound that does not stop bleeding by itself; |  |  | E | K | R | R | R |
| - correctly provide first aid for a skin wound in which a foreign object is embedded. |  |  |  |  | K | R | R |
| **Attitudes** |  |  |  |  |  |  |  |
| *See educational pathway General > Attitudes* |  |  |  |  |  |  |  |
| The children recognise the importance of:   - correctly providing first aid for a skin wound in which a foreign object is embedded. |  |  |  | E | K | R | R |
| Burns |  |  |  |  |  |  |  |
| **Knowledge** |  |  |  |  |  |  |  |
| The children:   - recognise a burn; | E | K | R | R | R |  |  |
| - know how to provide first aid for a burn (regardless of the degree of the burn); | E | K | R | R | R |  |  |
| - know when to seek medical help for a burn; |  |  | E | K | R | R | R |
| - know the difference between a superficial, intermediate and deep burn; |  |  |  | E | K | R | R |
| - know what commonly causes burns (hot water, flames, fire); | K | R | R | R |  |  |  |
| - know what can cause a burn (heat, chemicals, radiation...). | E | E | E | E | K | R | R |
| **Skills** | | | | | | | |
| The children can:   - correctly provide first aid for a burn; | E | K | R | R | R |  |  |
| - seek medical help if the burn is serious. |  |  |  | K | R | R | R |
| **Attitudes** |  |  |  |  |  |  |  |
| *See educational pathway General > Attitudes* |  |  |  |  |  |  |  |
| The children recognise the importance of:   - continuously applying water to a burn. | E | K | R | R | R |  |  |
| Bleeding |  |  |  |  |  |  |  |
| **Knowledge** |  |  |  |  |  |  |  |
| The children know:   - what they have to do in the event of a nose bleed; | E | K | R | R | R |  |  |
| - when to seek medical help for a nose bleed; |  | E | K | R | R |  |  |
| - how to correctly stop (severe) bleeding; |  |  | E | K | R | R |  |
| - that medical help must always be sought in the event of severe bleeding. |  |  | E | K | R | R |  |
| **Skills** |  |  |  |  |  |  |  |
| The children can:   - correctly stop a nose bleed; | E | K | R | R | R |  |  |
| - apply a bandage to stop (severe) bleeding [technique]. |  |  |  | K | R | R |  |
| **Attitudes** |  |  |  |  |  |  |  |
| *See educational pathway General > Attitudes* |  |  |  |  |  |  |  |
| The children recognise the importance of   - stopping a bleeding as quickly as possible. |  | E | K | R | R |  |  |
| Injuries to bones, muscles or joints |  |  |  |  |  |  |  |
| **Knowledge** |  |  |  |  |  |  |  |
| The children:   - know that they must not move a part of the body that is seriously painful; |  | E | K | R |  |  |  |
| - know three possible injuries to the motor system (dislocation, fracture, muscle or joint injury); |  |  | E | K | R | R |  |
| - recognise an injury to bones, muscles or joints; |  |  | E | K | R | R |  |
| - know the difference between an open and closed dislocation or fracture. |  |  |  |  | K | R | R |
| **Skills** |  |  |  |  |  |  |  |
| The children can:   - correctly provide first aid for a minor injury to bones, muscles or joints; |  |  |  | E | K | R | R |
| - can splint a broken bone; |  |  |  | E | K | R | R |
| - provide first aid to a person with an open fracture. |  |  |  |  | K | R | R |
| **Attitudes** |  |  |  |  |  |  |  |
| *See educational pathway General > Attitudes* |  |  |  |  |  |  |  |
| Poisoning |  |  |  |  |  |  |  |
| **Knowledge** |  |  |  |  |  |  |  |
| The children know:   - the signs of poisoning; |  |  |  | E | K | R | R |
| - some of the causes of poisoning (alcohol, drugs, toxic substances...) and how poisoning can be prevented; |  |  |  | E | K | R | R |
| - the dangers of carbon monoxide (CO) poisoning; |  |  |  | E | K | R | R |
| - some of the causes of CO poisoning and how it can be prevented; |  |  |  | E | K | R | R |
| - that urgent transportation to medical care is necessary; |  |  |  | E | K | R | R |
| - toxic substances that are poisonous when swallowed. | E | E | K | R | R |  |  |
| **Skills** |  |  |  |  |  |  |  |
| The children can:   - gather information on the nature and severity of the poisoning incident; |  |  |  | E | K | R | R |
| - seek medical help. |  |  |  | E | K | R | R |
| **Attitudes** |  |  |  |  |  |  |  |
| *See educational pathway General > Attitudes* |  |  |  |  |  |  |  |
| The children are aware of:   - the importance of assessing their own safety, that of the ill person and the bystanders in the case of CO poisoning. |  |  |  | E | K | R | R |
| Stings and bites | | | | | | | |
| **Knowledge** | | | | | | | |
| The children know:   - that a bee or wasp sting can be life-threatening because of allergic reactions; |  |  |  | E | K | R | R |
| - when to seek medical help in case of bee or wasp stings; |  |  |  | E | K | R | R |
| - know that they must always seek help from a medical care provider in case of a snake bite; |  | E | K | R | R | R | R |
| - that many snakes are poisonous, and that some types of snake venom can cause death; |  |  |  | E | K | R | R |
| - that it is not safe to touch or catch the snake; |  | E | K | R | R | R | R |
| - that sucking or cutting the venom out will not help and harm the person even more. |  |  |  | E | K | R | R |
| **Skills** | | | | | | | |
| The children can:   - remove a bee or wasp stinger; |  |  |  | E | K | R | R |
| - provide further first aid in case of a bee or wasp sting; |  |  |  | E | K | R | R |
| - splint a leg in case of a snake bite in the leg; |  |  |  | E | K | R | R |
| - provide further first aid in case of a snake bite. |  |  |  | E | K | R | R |
| **Attitude** | | | | | | | |
| *See educational pathway General > Attitudes* |  |  |  |  |  |  |  |
| The children recognise the importance of:   - correctly providing first aid in case of a bee or wasp sting; |  |  |  | E | K | R | R |
| - correctly providing first aid in case of a snake bite. |  |  |  | E | K | R | R |
| Fever | | | | | | | |
| **Knowledge** | | | | | | | |
| The children know:   - that fever can be a sign of serious illness; |  |  | K | R | R | R | R |
| - that a person with fever needs medical attention to determine the cause of the fever; |  |  | K | R | R | R | R |
| - that fever can be very dangerous and lead to dead if left untreated; |  |  | K | R | R | R | R |
| - that a person with fever needs to rest and drink lots of fluids. |  |  | K | R | R | R | R |
| Diarrhoea | | | | | | | |
| **Knowledge** | | | | | | | |
| The children know:   - that diarrhoea is usually caused by an infection; |  |  | K | R | R | R | R |
| - that poor hygiene (not washing hands, touching stools, eating unsafe food, drinking unsafe water) can lead to diarrhoea; |  |  | K | R | R | R | R |
| - that diarrhoea causes dehydration; |  |  | K | R | R | R | R |
| - that diarrhoea can be very dangerous and lead to dead if left untreated; |  |  | K | R | R | R | R |
| - when to seek medical help in case of diarrhoea; |  |  |  | K | R | R | R |
| - someone with diarrhoea needs to drink lots of fluids. |  |  | K | R | R | R | R |
| Fits | | | | | | | |
| **Knowledge** | | | | | | | |
| The children know:   - that fits are not caused by demons or bad spirits, but are the result of physical illnesses or injuries; |  | E | K | R | R | R | R |
| - that someone having fits is not contagious; |  | E | K | R | R | R | R |
| - that fits can be accompanied by other symptoms such as loss of consciousness, discolouration of nails and lips, eyes turning away,…; |  |  |  | K | R | R | R |
| - they should not hold the fitting person down or put objects into his mouth; |  |  | K | R | R | R | R |
| - how to provide first aid in case of fits; |  |  | E | K | R | R | R |
| - when to seek medical help in case of fits. |  |  |  | K | R | R | R |
| Disaster principles | | | | | | | |
| **Knowledge** | | | | | | | |
| The children know:   - the concept of risk awareness; |  |  |  | E | K | R | R |
| - the concept of which disaster victims are more likely to survive and different categories of victims to treat (triage) |  |  |  | E | K | R | R |
